# The Discovery of Potential SARS-CoV-2 Natural Inhibitors among 4924 African Metabolites Targeting the Papain-like Protease: A Multi-Phase In Silico Approach

**DOI:** 10.3390/metabo12111122

**Published:** 2022-11-16

**Authors:** Eslam B. Elkaeed, Mohamed M. Khalifa, Bshra A. Alsfouk, Aisha A. Alsfouk, Abdul-Aziz M. M. El-Attar, Ibrahim H. Eissa, Ahmed M. Metwaly

**Affiliations:** 1 Department of Pharmaceutical Sciences, College of Pharmacy, AlMaarefa University, Riyadh 13713, Saudi Arabia; 2 Pharmaceutical Medicinal Chemistry & Drug Design Department, Faculty of Pharmacy (Boys), Al-Azhar University, Cairo 11884, Egypt; 3 Department of Pharmaceutical Sciences, College of Pharmacy, Princess Nourah bint Abdulrahman University, P.O. Box 84428, Riyadh 11671, Saudi Arabia; 4 Pharmaceutical Analytical Chemistry Department, Faculty of Pharmacy, Al-Azhar University, Nasr City, Cairo 11884, Egypt; 5 Pharmacognosy and Medicinal Plants Department, Faculty of Pharmacy (Boys), Al-Azhar University, Cairo 11884, Egypt; 6 Biopharmaceutical Products Research Department, Genetic Engineering and Biotechnology Research Institute, City of Scientific Research and Technological Applications (SRTA-City), Alexandria 21934, Egypt

**Keywords:** African natural products, SARS- papain-like protease, molecular fingerprints, structural similarity, docking, ADMET, toxicity, molecular dynamics simulations

## Abstract

Four compounds, hippacine, 4,2′-dihydroxy-4′-methoxychalcone, 2′,5′-dihydroxy-4-methoxychalcone, and wighteone, were selected from 4924 African natural metabolites as potential inhibitors against SARS-CoV-2 papain-like protease (PLpro, PDB ID: 3E9S). A multi-phased in silico approach was employed to select the most similar metabolites to the co-crystallized ligand (**TTT**) of the PLpro through molecular fingerprints and structural similarity studies. Followingly, to examine the binding of the selected metabolites with the PLpro (molecular docking. Further, to confirm this binding through molecular dynamics simulations. Finally, in silico ADMET and toxicity studies were carried out to prefer the most convenient compounds and their drug-likeness. The obtained results could be a weapon in the battle against COVID-19 via more in vitro and in vivo studies.

## 1. Introduction

On 11 November 2022, the WHO stated that the confirmed global infections of COVID-19 were 630,832,131, with 6,584,104 people dead [1]. Conforming to these gigantic numbers, massive work is demanded from scientists worldwide to find a cure.

The evolution of computational chemistry methods as a successful tool to conclude the physical and chemical properties of a molecule and the molecular reactions allowed deep identification of the molecular properties of compounds in addition to their interactions with different proteins [2]. Consequently, the in silico prediction of the activity of large libraries of compounds against a specific molecular target became available [3]. The computational (in silico) chemistry methods have been employed in drug discovery [4,5,6], molecular modeling [7], and design [8,9]. Additionally, it has been used to predict ADMET [10,11,12], toxicity [13,14,15] as well as DFT [16] properties.

The interest of humans in the use of natural products can be traced back hundreds of years and continues to the present [17,18].

The papain-like protease, PLpro, is a vital enzyme in the coronavirus. PLpro has an important role in the processing mechanism of viral polyproteins. This process leads to the formation of an active replicase complex [19]. Besides, PLpro has an additional vital role in deactivating human immunity. PLpro acts on human enzymes via cleaving proteinaceous post-translational modifications [20].

Our teamwork employed the in silico approaches to explore the potentialities of natural products against COVID-19 several times before. The determination of the most convenient inhibitors between fifty-nine isoflavonoids against hACE2 and main viral protease has been reported [21]. Likely, the activities of a set of fifteen alkaloids against COVID-19 five enzymes have been published [22].

In the presented work, a set of 4924 African natural products (compounds isolated from African natural sources) has been selected. The experiment set was obtained from the African Natural Products Database (ANPDB), a collection of several natural product databases in different African regions. The selected data set covered the period of 1962–2019 and was derived from international and local African journals in addition to MSc and Ph.D. theses in African university libraries [23].

The selected compounds were screened using multistage computational methods to detect the most potent SARS-CoV-2 PLpro inhibitors. The applied methods included molecular structures similarity study against the co-crystallized ligand (**TTT**) of PLpro (PDB ID: 3E9S) [24], fingerprint study against the same ligand, molecular docking against PLpro, ADMET, toxicity and molecular dynamics (MD) simulation experiments.

## 2. Method

### 2.1. Molecular Similarity Detection

Discovery Studio 4.0 software, 2016, Vélizy-Villacoublay, France, was used to investigate the similarities between 4924 African natural metabolites and **TTT**, the co-crystallized ligand of PLpro (Supplementary Data provides comprehensive details).

### 2.2. Fingerprint Studies 

Discovery Studio 4.0 software was used to investigate the similarities between 100 African natural metabolites and **TTT**, the co-crystallized ligand of PLpro (Supplementary Data provides comprehensive details).

### 2.3. Docking Studies 

Docking studies were done for the most similar 40 metabolites against the PLpro protease (PDB ID: 3E9S) using Discovery studio software [25] to investigate the binding energies as well as binding modes (Supplementary Data provides comprehensive details).

### 2.4. ADMET Analysis

Discovery Studio 4.0 was used [26] to examine 5 different ADMET parameters for 17 metabolites of correct binding scores (Supplementary Data provides comprehensive details).

### 2.5. Toxicity Studies 

Discovery Studio 4.0 software was used [27,28,29] to examine 7 different toxicity parameters for 7 metabolites of good ADMET profile (Supplementary Data provides comprehensive details).

### 2.6. Molecular Dynamics Simulation

The PLpro-wighteone system was prepared using the web-based CHARMM-GUI [30,31,32] interface utilizing the CHARMM36 force field [33] and NAMD 2.13 [34] package. The TIP3P explicit solvation model was used (Supporting Data provides comprehensive details).

## 3. Results and Discussion 

### 3.1. Structure Fingerprints Study 

The basic assumption of structure-activity relationship studies is that “Chemical compounds with similar structures may have similar activities” [35]. This assumption was very useful in discovering several bioactive ligands [36]. The high affinity of the co-crystallized ligand to bind with the targeted protein was our main motive in this work. We utilized some ligand-based computational techniques such as structure fingerprints and similarity to select the natural compounds (through the examined library) that have high degrees of similarities and hence could bind with PLpro effectively. The fingerprint study is a molecular descriptor technique widely used to figure out the similarity or dissimilarity between the chemical structures of two molecules or more [37,38]. In fingerprint study, the software converts the basic chemical molecular descriptors into mathematical symbols. The resulting data is displayed as bit strings that identify the presence (1) or absence (0) of a specific 2D atomic or fragment descriptor in both test and reference compounds [39,40]. In this study, Discovery Studio software examined the molecular fingerprints of 4924 compounds against **TTT**. This study aims to extract the most similar natural compounds to the ligand. The employed descriptors are H-bond acceptor [41] and donor [42], charge [43], hybridization [44], positive [45] and negative ionizable atoms [46], halogens [47], aromatic [48], or none of the above besides the ALogP [49] category of fragments and atoms. The study was adjusted to choose the most structurally similar 200 compounds to **TTT** (Table 1).

### 3.2. Molecular Similarity

The molecular similarity study differs from the fingerprints study in that the first computes certain descriptors regarding the whole chemical structure of a molecule. The computed descriptors are topological, steric, electronic, and/or physical properties [50]. On the other hand, the fingerprints study compares the absence or presence of certain 2D atom paths, fragments, or substructures in the chemical structures of reference and test molecules [51].

**Figure 1:**
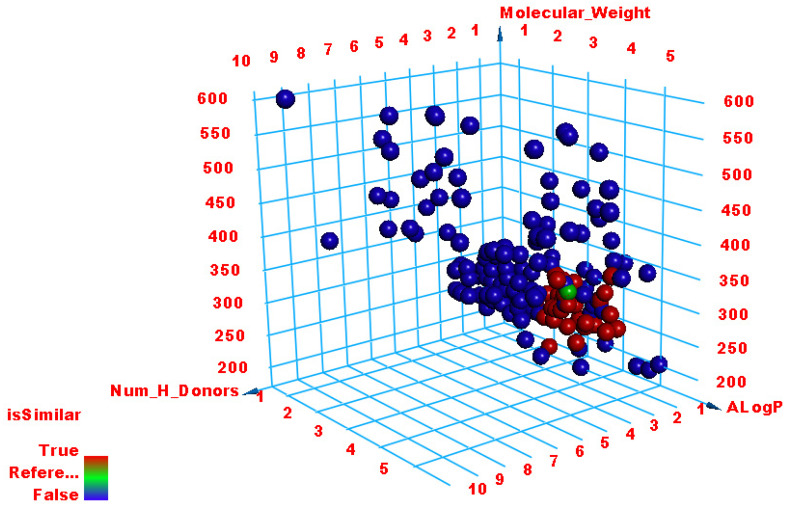
Molecular similarity analysis of the African metabolites and **TTT**.

**Figure 2:**
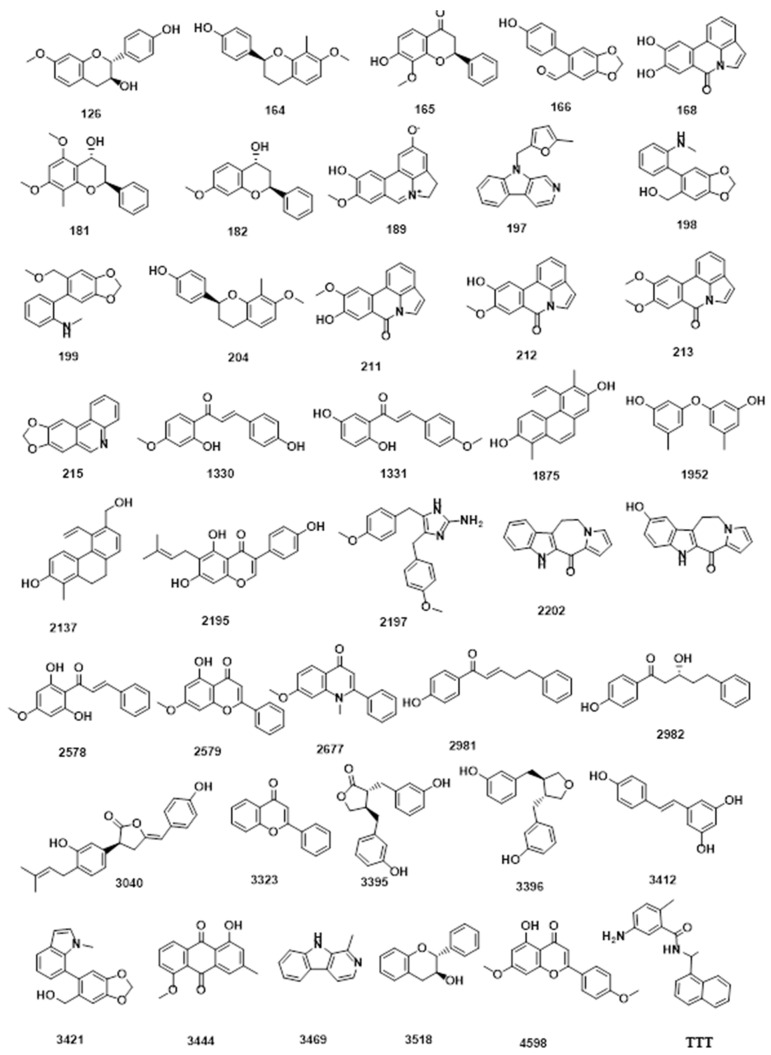
The most similar African metabolites to **TTT**.

Employing Discovery studio software, the molecular similarities of the selected 100 natural metabolites were investigated correlating **TTT**. The employed properties in this study (Figure 1 and Table 2) were partition coefficient (ALog p) [52], molecular weight (M. Wt) [53], H- bond donors (HBA) [54], H- bond acceptors (HBD) [55], rotatable bonds number [56], number of rings along with aromatic rings [57], and minimum distance [58] as well as the molecular fractional polar surface area (MFPSA) [59]. The experiment was adjusted to extract the most similar 40 compounds (Figure 2).

### 3.3. Docking Studies

The forty most structurally similar compounds were subjected to a molecular docking study in the hope of getting an insight into the way they interact with their biomolecular target. The papain-like protease (PLpro) crystal structure PDB ID: 3E9S in complex with the co-crystallized ligand, **TTT,** was adopted for the present study. A docking study was performed using MOE 14.0 software, Montreal, Canada. The calculated ΔG of the tested compounds is cited in Table 3.

**Figure 3:**
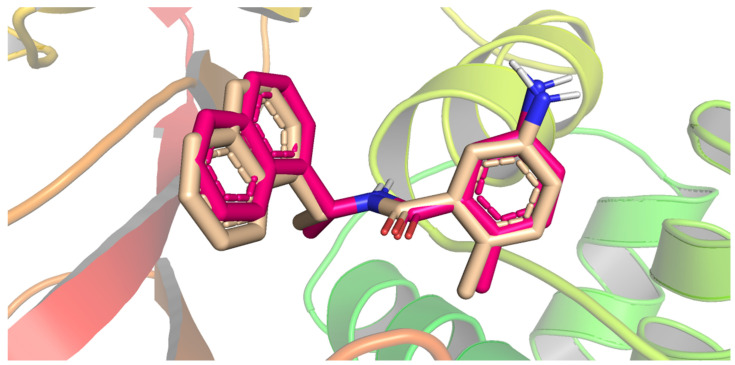
Superimposition of the co-crystallized ligand pose (pink) and the docking pose (wheat).

The docking protocol was first validated via the redocking of the co-crystallized ligand (**TTT**) against the active pocket of SARS papain-like protease (PLpro) active pocket. However, the validation step proved the suitability of the performed protocol for the intended docking study, as demonstrated by the small RMSD (0.51 Å) between the docked pose and the co-crystallized ligand (Figure 3).

**Figure 4:**
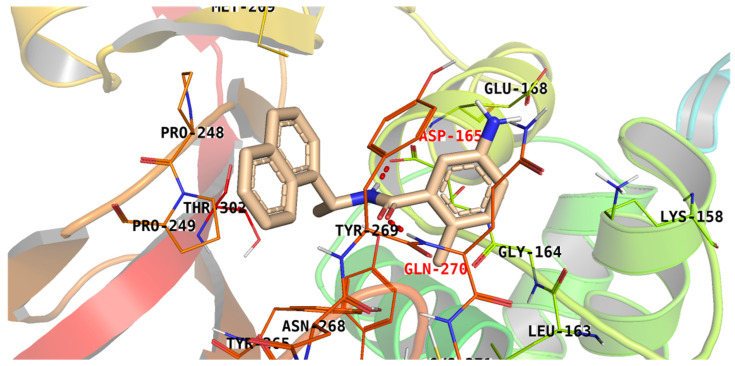
The proposed binding pattern of **TTT** against the PLpro active site.

**TTT**, the well-known PLpro inhibitor, was used as a reference in the current study. The binding affinity value of **TTT** was −9.30 kcal/mol. **TTT** interacted with the active pocket through the formation of two H-bonds. The amidic NH group of **TTT** formed an H-bond with the carboxylic acid side chain of Asp165, while the amidic carbonyl bound to the nitrogen backbone of Gln270. Additionally, the naphthyl moiety was involved in a hydrophobic interaction with the Pro249 side chain (Figure 4).

Results of the docking study showed that most of the tested compounds have a similar position and orientation inside the SARS PLpro active site. Among them, members **2195**, **1952**, **2982**, and **1330** revealed the greatest binding free energies of docking, which were almost close to the redocked ligand.

**Figure 5:**
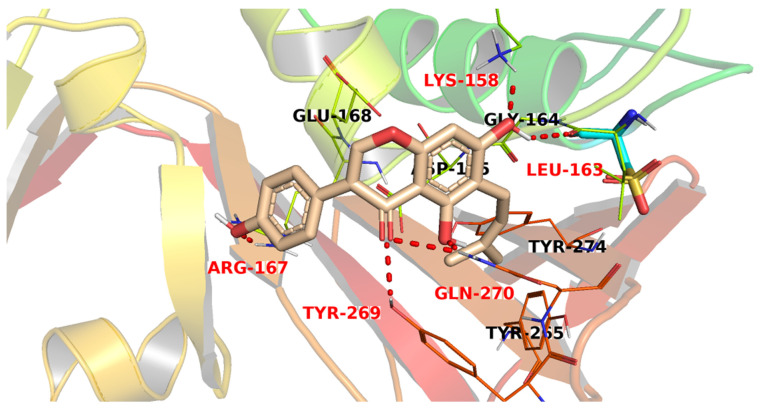
The proposed binding pattern of compound **2195**.

The docking simulation of compound **2195** revealed that it has the highest fitting into the enzyme active site with a docking score of −24.88 kcal/mol. It was stabilized in the active site through the formation of six H-bond interactions and many hydrophobic interactions. The chromenone moiety, via its carbonyl and hydroxyl groups, formed five H-bonds with Tyr269, Gln270, Leu263, and Lys253. On the other side, the p-hydroxyphenyl moiety was involved in an H-bond with Arg167 Figure 5. The chromenone moiety and the phenyl ring also formed hydrophobic interactions with Tyr269 and Lys158, respectively.

**Figure 6:**
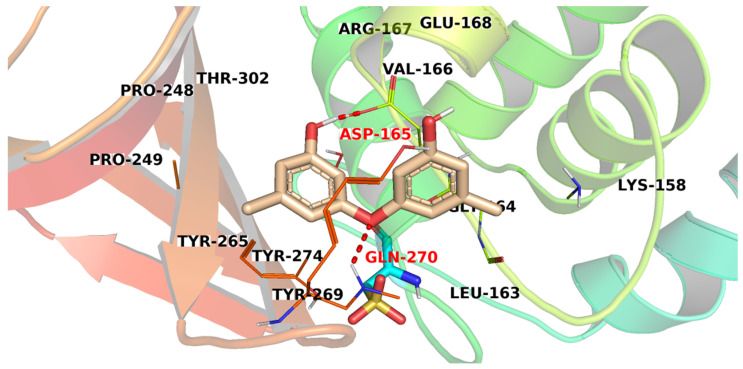
The proposed binding pattern of compound **1952**.

Compound **1952** exhibited a binding mode similar to that of the co-crystallized ligand with the formation of two H-bonds. One H-bond was formed between a hydroxyl group side chain with Asp165. The other H-bond was formed between the oxygen bridge and Gln270 (Figure 6). Additionally, a hydrophobic interaction was formed between one phenyl moiety and Tyr265 of the active site.

**Figure 7:**
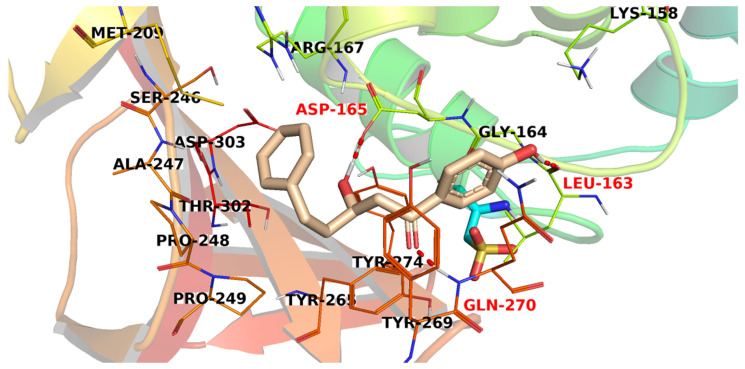
The proposed binding pattern of compound **2982**.

A study of the top docking poses of member **2982** (Figure 7) showed that it interacted with the PLpro active site through the formation of three H-bond interactions. The hydroxyphenyl moiety was involved in an H-bonding with Leu163, while the aliphatic hydroxyl group formed another H-bond with Asp165. In addition, the carbonyl group formed an H-bond with Gln270.

**Figure 8:**
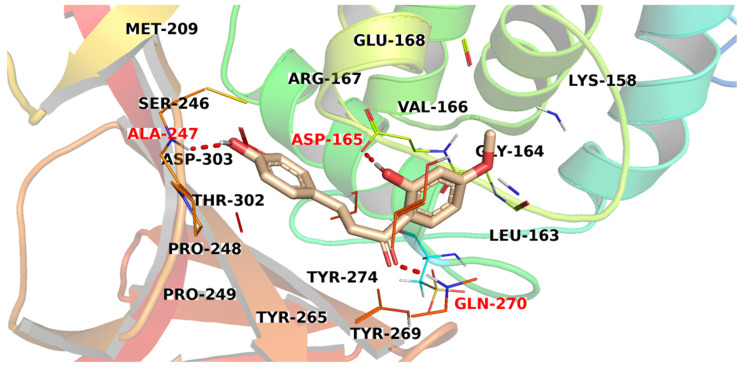
The proposed binding pattern of compound **1330**.

Figure 8 illustrates the proposed binding mode of compound **1330**. The two phenolic hydroxyl groups interacted with the active site by two H-bonds with Ala247 and Gln270. Furthermore, the carbonyl group formed an H-bond with Asp165.

### 3.4. ADMET Studies

The ability of a molecule to be a drug is decided not only by activity but also by acceptable pharmacokinetic properties. ADMET profile describes absorption, distribution, metabolism, excretion, and toxicity. Although the determination of the ADMET profile is available via several medium- and high-throughput in vitro methods, the ability to predict it depending on in silico is available with the advantage of saving time, money, effort, and animal lives [60]. ADMET prediction is an essential step in drug discovery [61].

**Figure 9:**
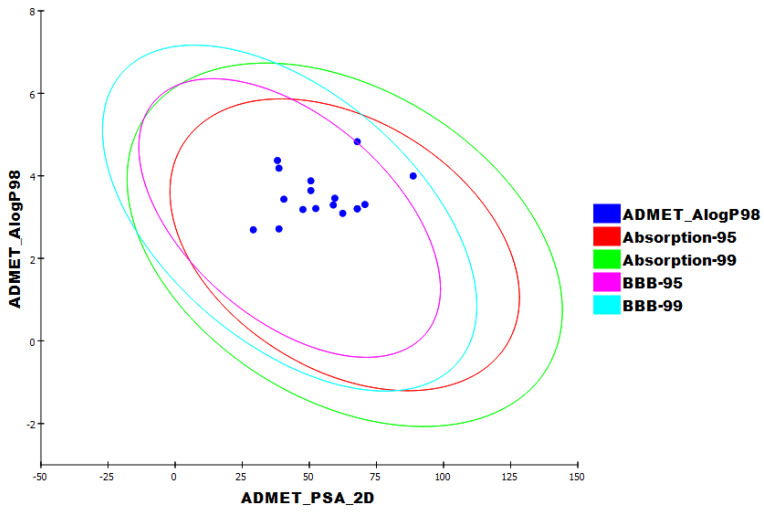
ADMET profile of the African metabolites and the reference.

The computed ADMET descriptors for 17 compounds that displayed correct binding mode and energy, as well as remdesivir as a reference drug, are listed in (Table 4 and Figure 9). Compounds **181**, **182**, **204**, **212**, **213**, **215**, **1952**, **2981**, **3040**, and **3396** were expected to have a high ability to pass BBB and, accordingly, were eliminated. Fortunately, the absorption levels of all compounds were computed as good. Similarly, all of them showed low to good aqueous solubility levels. All compounds were expected to bind to plasma protein with a ratio of more than 90%. Finally, according to these results, compounds Hippacine (**164**), Naamine D (**2197**), (±)-Enterofuran (**3412**), Daphnelone (**2982**), 4,2′-dihydroxy-4′-methoxychalcone (**1330**), 2′,5′-dihydroxy-4-methoxychalcone (**1331**), and wighteone (**2195**) were favored and subjected to the next toxicity examination.

### 3.5. Toxicity Studies

The prediction of toxicity of a molecule depending on computer software (in silico) has been employed effectively to select drug leads in the field of drug design, as in vitro and in vivo methods are usually limited by lack of time, budget, and ethical restrictions [62,63].

The toxicity of 7 compounds that displayed good ADMET profiles was predicted in silico using Discovery Studio software concerning 7 different models. The employed models are FDA rat carcinogenicity [64,65], carcinogenic potency median toxic dose, (TD_50_) [66], rat maximum tolerated dose (MTD) [67,68], rat oral LD_50_ [69], rat chronic lowest-observed-adverse-effect level (LOAEL) [70,71], and ocular and skin irritancy [72]. As shown in Table 5, compounds **2982** and **3412** were proposed as carcinogenic. In consequence, both were refused. Also, all compounds, excluding **2197**, are expected to have TD_50_ and TD_50_ values more than the reference. Thus, **2197** was excluded too. All compounds were computed to have LOAEL values more than the reference and to be non-irritant in the skin model. On the other hand, all compounds except **1330** showed different degrees of ocular irritancy.

The acquired results privilege compounds Hippacine (**164**), 4,2′-dihydroxy-4′-methoxychalcone (**1330**), 2′,5′-dihydroxy-4-methoxychalcone (**1331**), and wighteone (**2195**) as the most convenient inhibitors against the target enzyme. Among the selected compounds, wighteone displayed the most favorable docking score and energy. Wighteone is an isoflavonoid that has been isolated from the bark of a South African *Erythrina* species showing promising antibacterial effects [73]. It was also isolated from several *Maclura* species before [74,75]. The antiviral activity of wighteone against HIV has been reported in vitro [76], and its in silico potentiality against HIV-1 protease enzyme with a binding affinity of −8.7 Kcal/mol [77].

### 3.6. Molecular Dynamics (MD) Simulations

Although molecular docking can predict the correct binding poses of a molecule inside the active site of a certain protein, it has a major drawback in that it considers the proteins rigid, and thus doesn’t allow the protein to adjust its conformation during the docking process [78]. On the other hand, MD simulations can efficiently predict how every single atom in a specific protein will move over a specific time, depending on a physical model of the interatomic interactions [79]. Correspondingly, MD simulations have been successfully utilized to examine the conformation changes in protein-ligand interactions and protein dynamics and folding [80]. MD simulation is an effective and accurate in silico technique that can describe the binding mode, stability, and flexibility of a certain receptor and a specific ligand for a determined time [81].

Molecular dynamics (MD) simulations were carried out to mimic the dynamic nature of PLpro-wighteone interaction under physiological conditions and to investigate the stability of binding complex simulation for 100 ns.

#### RMSD and RMSF Analysis

**Figure 10:**
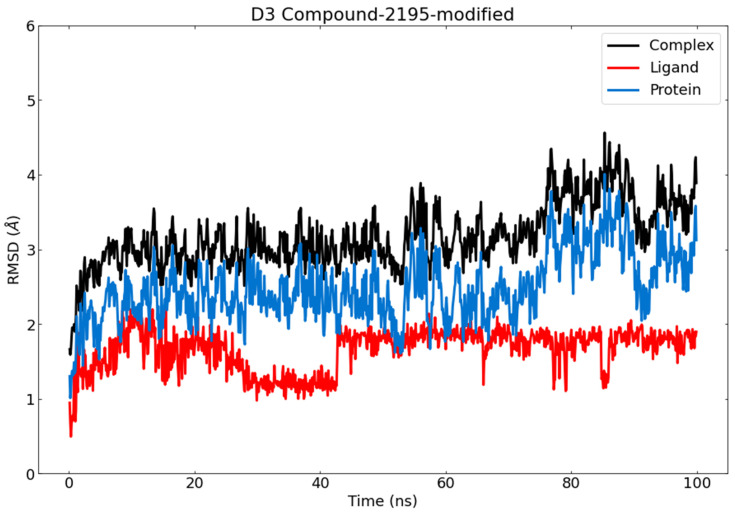
RMSD value during MD runs. (Red: (wighteone), blue: (PLpro), black: (PLpro- wighteone complex).

The binding of a certain ligand in a specific protein causes notable changes in the structure [82]. Consequently, the root mean square deviation (RMSD) parameter was investigated to explore whether the structure of the PLpro-wighteone complex is stable and near the experimental structure. Figure 10 shows that the PLpro-wighteone complex exhibited a good RMSD value along with 100 ns MD; the PLpro showed an RMSD value of 2.5 Å too, while the complex exhibited an average RMSD value of 3.5 Å, below the acceptable range of 4 Å. After 60 ns, no dramatic increment in the RMSD values was noticed and the complex system reached equilibrium.

**Figure 11:**
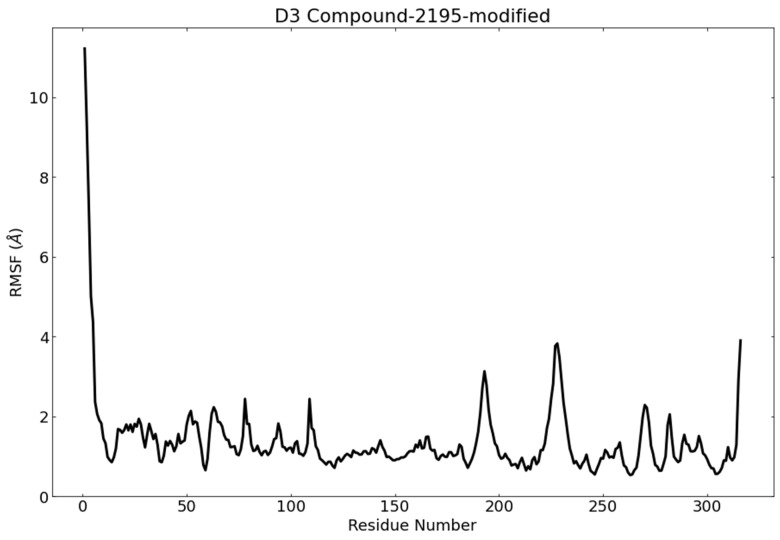
RMSF of PLpro in the MD run.

Root mean square fluctuation (RMSF) was utilized to describe the flexibility differences among wighteone, PLpro, and their complex during the MD simulation for 100 ns. Increasing RMSF value denotes a higher degree of flexibility, while the low value is related to limited movement during the MD simulation. To investigate the average fluctuation of PLpro during the MD study, the RMSF of PLpro upon the binding of wighteone was plotted as a function of residue number (Figure 11). RMSF plot indicated that the residual fluctuation of PLpro was minimized upon binding of wighteone. This result indicates that PLpro residues were more rigid in the presence of wighteone because of binding to wighteone.

**Figure 12:**
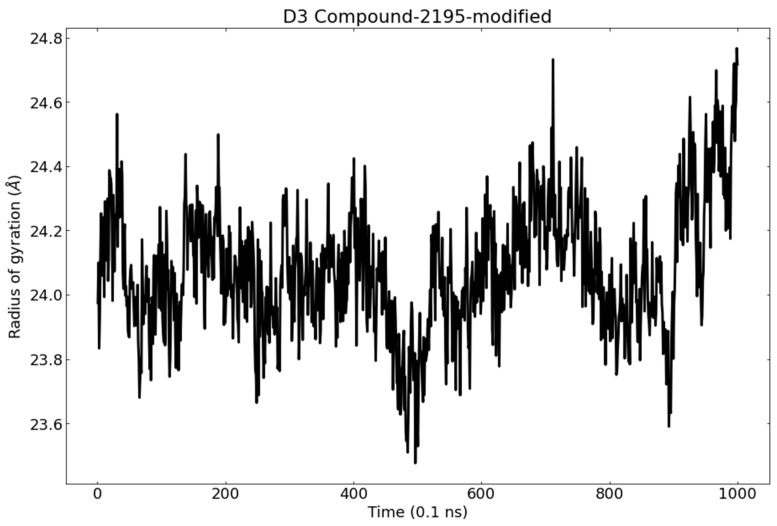
The radius of gyration of PLpro in the MD run.

The radius of gyration (R_g_) is an essential parameter that gives a clear insight into the protein stability in terms of volume change. R_g_ is defined as the RMSD of the mass-weighted of a group of atoms from their common mass center [83,84]. Accordingly, the analysis of R_g_ of PLpro during the MD simulation will describe its overall dimensions. The average R_g_ values were found to suggest the tight packing of PLpro in its native state and when bound to wighteone. PLpro-wighteone complex reached a stable conformation with the radius of gyration fluctuating around 24.4 Å (Figure 12).

**Figure 13:**
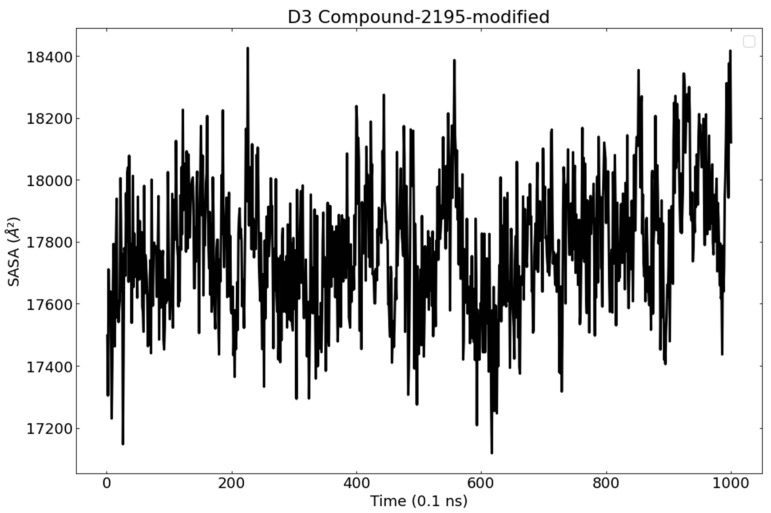
SASA of PLpro in the MD run.

The solvent-accessible surface area (SASA) is the surface area of the protein which can be accessible to a solvent [85]. The evaluation of SASA provides information about the conformational changes that happen in a protein because of ligand binding. The average SASA values for PLpro were monitored during 100 ns MD simulations. As shown in Figure 13, there were no major changes in the values of SASA of PLpro due to wighteone binding.

## 4. Discussion

The recent advancement in software enabled computational chemistry to perfectly describe the physical and chemical properties of a compound in addition to its potential to interact with a particular protein.

Accordingly, several researchers utilized computational chemistry to identify potential inhibitors against SARS-CoV-2 using different approaches. Exploring the potentialities of FDA-approved antivirus drugs against SARS-CoV-2 was one of the first computational approaches. For instance, the computational potentialities of remdesivir, the FDA-approved anti-ebola, and respiratory syncytial viruses against SARS-CoV-2 main protease were investigated [86]. The same approach was applied to lopinavir/ritonavir [87] and ribavirin [88] targeting SARS-CoV-2 3-chymotrypsin-like protease. One of the employed approaches was the computational-based drug repurposing of non-antiviral FDA-approved drugs such as lurasidone (anti-schizophrenia) against SARS-CoV-2 3CL hydrolase and protease enzymes [89], aclarubicin [90], and selinexor [91], FDA-approved anti-cancers that exhibited computational activities against SARS-CoV-2 main protease.

Our team employed computational chemistry to develop a multiphase in silico technique to discover the most appropriate natural inhibitor via large sets of molecules against a specific enzyme of COVID-19. Within 310 natural antiviral metabolites, the most effective inhibitor against SARS-CoV-2 nsp10 [92], main protease [93,94], and papain-like protease [95] were predicted. Also, within 3009 FDA approved drugs, the most potent inhibitors against SARS-CoV-2 nsp16-nsp10 2′-*o*-Methyltransferase Complex [96] and SARS-CoV-2 RNA-Dependent RNA Polymerase [97] were anticipated. The SARS-CoV-2 Helicase potential natural inhibitors were expected among 5956 compounds of traditional Chinese medicine also [98]. Further, the most active semisynthetic COVID-19 papain-like protease inhibitor was discovered amidst 69 molecules [99].

Unfortunately, at the current time, we don’t have access to investigate the experimental inhibitory effects of the pointed 4 metabolites (Hippacine, 4,2′-dihydroxy-4′-methoxychalcone, 2′,5′-dihydroxy-4-methoxychalcone, and wighteone) among 4924 African natural metabolites against SARS-CoV-2. However, we presented those 4 metabolites for all scientists worldwide to conduct further in vitro and in vivo studies. The binding potentialities of those metabolites against the SARS-CoV-2 papain-like protease were confirmed through 4 stages of in silico experiments:

Stage I: Selection of the most similar metabolites to the co-crystallized ligand (**TTT**) of SARS-CoV-2 papain-like protease (PDB ID: 3E9S) (fingerprints and molecular similarity studies). This stage selected the most similar 40 metabolites to the co-crystallized ligand;

Stage II: Evaluation and filtration according to the binding against papain-like protease by molecular docking to select 17 metabolites that showed correct binding;

Stage III: Evaluation and the filtration according to drug-likeness by ADMET and toxicity studies to point out the safest and most drug-like 4 metabolites;

Stage IV: Confirmation of the binding against papain-like protease by MD simulations to confirm the binding, conformational and energetic changes that combine the binding process. SARS-CoV-2 papain-like protease (PLpro, PDB ID: 3E9S). A multi-phased in silico approach was employed to select the most similar metabolites to the co-crystallized ligand (**TTT**).

## 5. Conclusions

Four metabolites, Hippacine (**164**), 4,2′-dihydroxy-4′-methoxychalcone (**1330**), 2′,5′-dihydroxy-4-methoxychalcone (**1331**), and Wighteone (**2195**), were selected through 4924 African natural products as the most potent inhibitor against Sars-Cov-2 papain-like protease. The selection is based on multiphase (six experiments) in silico studies. The structural fingerprint study against the co-crystallized ligand (**TTT**) of SARS-CoV-2 papain-like protease (PDB ID: 3E9S), chemical structural similarity study, molecular docking studies against SARS-CoV-2 papain-like protease (PDB ID: 3E9S), ADMET, and toxicity profiles. Wighteone (**2195**), the metabolite with the best docking score, was subjected to the molecular dynamics simulation (MD) at 100 ns confirming the binding of wighteone against the target enzyme. We present these interesting results for all scientists worldwide to conduct further in vitro and in vivo studies concerning these promising natural metabolites.

## Figures and Tables

**Table 1:** Fingerprint similarity between the tested African metabolites and **TTT**.

Compound	Similarity	SA	SB	SC	Compound	Similarity	SA	SB	SC
**TTT**	1.000	454	0	0	**3448**	0.632653	279	−13	175
**2538**	0.758	322	−29	132	**3647**	0.632479	296	14	158
**3518**	0.747	324	−20	130	**292**	0.632249	447	253	7
**3323**	0.743	324	−18	130	**1795**	0.632054	280	−11	174
**2982**	0.743	329	−11	125	**3414**	0.631699	554	423	−100
**2981**	0.738	327	−11	127	**2259**	0.631188	255	−50	199
**182**	0.732	314	−25	140	**3040**	0.63035	324	60	130
**2677**	0.720	317	−14	137	**1157**	0.630081	310	38	144
**2558**	0.712	442	167	12	**1141**	0.629797	279	−11	175
**2554**	0.710	316	−9	138	**2180**	0.62963	255	−49	199
**1875**	0.710	320	−3	134	**203**	0.628889	283	−4	171
**197**	0.708	334	18	120	**3413**	0.628831	554	427	−100
**1168**	0.703	298	−30	156	**2108**	0.628062	282	−5	172
**2556**	0.703	298	−30	156	**1332**	0.628009	287	3	167
**1001**	0.702	297	−31	157	**3039**	0.627907	324	62	130
**165**	0.701	303	−22	151	**3420**	0.627273	276	−14	178
**2221**	0.701	328	14	126	**3085**	0.626506	260	−39	194
**2579**	0.700	301	−24	153	**3115**	0.626223	320	57	134
**4579**	0.699	588	387	−134	**1154**	0.625541	289	8	165
**1195**	0.698	296	−30	158	**1153**	0.625541	289	8	165
**900**	0.698	296	−30	158	**1169**	0.625282	277	−11	177
**2197**	0.697	355	55	99	**161**	0.625282	277	−11	177
**212**	0.697	306	−15	148	**1140**	0.625282	277	−11	177
**211**	0.697	306	−15	148	**2588**	0.625282	277	−11	177
**2578**	0.694	300	−22	154	**4598**	0.62256	287	7	167
**205**	0.691	318	6	136	**848**	0.622517	282	−1	172
**2555**	0.688	296	−24	158	**1155**	0.62203	288	9	166
**3079**	0.687	398	125	56	**3412**	0.620843	280	−3	174
**4573**	0.687	417	153	37	**2137**	0.620536	278	−6	176
**2557**	0.686	308	−5	146	**2407**	0.620451	358	123	96
**4572**	0.686	410	144	44	**1147**	0.62	279	−4	175
**3421**	1	311	0	143	**637**	0.619048	325	71	129
**2202**	1	282	−42	172	**2201**	0.618893	380	160	74
**213**	1	311	1	143	**2199**	0.618487	368	141	86
**2067**	1	449	206	5	**189**	0.617849	270	−17	184
**126**	1	294	−21	160	**1156**	0.61753	310	48	144
**2070**	1	470	239	−16	**2203**	0.617021	261	−31	193
**1330**	1	293	−21	161	**2685**	0.616725	354	120	100
**168**	1	301	−9	153	**1992**	0.616279	265	−24	189
**4575**	1	417	163	37	**3469**	0.615	246	−54	208
**1132**	1	387	119	67	**4879**	0.614232	328	80	126
**3419**	0.673289	305	−1	149	**2**	0.614191	277	−3	177
**1133**	0.673043	387	121	67	**3924**	0.613333	276	−4	178
**190**	0.671772	307	3	147	**713**	0.612691	280	3	174
**1331**	0.670507	291	−20	163	**2206**	0.610132	277	0	177
**181**	0.67033	305	1	149	**1952**	0.609977	269	−13	185
**1000**	0.670306	307	4	147	**990**	0.609865	272	−8	182
**163**	0.668161	298	−8	156	**3392**	0.609865	272	−8	182
**2958**	0.667814	388	127	66	**166**	0.609589	267	−16	187
**3923**	0.667431	291	−18	163	**3411**	0.607692	553	456	−99
**926**	1	304	3	150	**2189**	0.606762	341	108	113
**3473**	1	304	3	150	**3445**	0.605905	472	325	−18
**2198**	1	375	110	79	**4580**	0.60414	467	319	−13
**2065**	1	455	231	−1	**1861**	0.60414	467	319	−13
**204**	1	290	−17	164	**1859**	0.60414	467	319	−13
**3395**	1	315	21	139	**3410**	0.603712	553	462	−99
**215**	1	291	−13	163	**4577**	0.603359	467	320	−13
**4571**	1	405	160	49	**1642**	0.602794	302	47	152
**169**	0.658314	289	−15	165	**1643**	0.602794	302	47	152
**3114**	0.657505	311	19	143	**1644**	0.602794	302	47	152
**3394**	0.655602	316	28	138	**2107**	0.602687	314	67	140
**198**	0.655012	281	−25	173	**671**	0.602637	320	77	134
**1212**	0.655012	281	−25	173	**4578**	0.602581	467	321	−13
**3124**	0.653277	309	19	145	**4581**	0.602581	467	321	−13
**3098**	0.653277	309	19	145	**1977**	0.602076	348	124	106
**2227**	0.652268	302	9	152	**3442**	0.60177	272	−2	182
**2471**	0.65	299	6	155	**2194**	0.601643	293	33	161
**3444**	0.649886	284	−17	170	**2467**	0.600887	542	448	−88
**3629**	0.649874	258	−57	196	**2636**	0.600751	480	345	−26
**199**	0.648402	284	−16	170	**2635**	0.600751	480	345	−26
**1137**	1	419	193	35	**4750**	0.600742	486	355	−32
**1136**	1	419	193	35	**2496**	0.600671	537	440	−83
**1138**	1	419	193	35	**4870**	0.600372	323	84	131
**1139**	1	419	193	35	**692**	0.599589	292	33	162
**1464**	0.647208	255	−60	199	**2195**	0.599589	292	33	162
**4574**	0.646965	405	172	49	**4549**	0.599567	554	470	−100
**1768**	1	357	98	97	**4551**	0.599341	546	457	−92
**3754**	1	486	298	−32	**1143**	0.597802	272	1	182
**2222**	1	285	−13	169	**4128**	0.597802	272	1	182
**3396**	1	313	31	141	**1352**	0.597802	272	1	182
**155**	0.645161	300	11	154	**4601**	0.597802	272	1	182
**167**	0.644295	288	−7	166	**2118**	0.597802	272	1	182
**2888**	1	284	−13	170	**2667**	0.596899	308	62	146
**4266**	0.643991	284	−13	170	**4486**	0.596491	272	2	182
**4759**	0.643392	258	−53	196	**3118**	0.596429	334	106	120
**1118**	0.642132	253	−60	201	**3073**	0.595133	269	−2	185
**3113**	0.642127	314	35	140	**227**	0.594771	273	5	181
**3925**	0.641553	281	−16	173	**228**	0.594771	273	5	181
**2220**	0.640244	315	38	139	**3071**	0.594714	270	0	184
**4899**	1	282	−13	172	**4570**	0.594714	270	0	184
**279**	1	282	−13	172	**4897**	0.594714	270	0	184
**1447**	1	282	−13	172	**2684**	0.594454	343	123	111
**1559**	1	282	−13	172	**4485**	0.594421	277	12	177
**281**	0.639456	282	−13	172	**278**	0.594298	542	458	−88
**164**	0.639269	280	−16	174	**4896**	0.593407	270	1	184
**4468**	0.639098	340	78	114	**1117**	0.593407	270	1	184
**1134**	0.638066	409	187	45	**1152**	0.593254	299	50	155
**1135**	0.638066	409	187	45	**1448**	0.593148	277	13	177
**2200**	1	376	138	78	**4129**	0.593148	277	13	177
**1949**	0.634033	272	−25	182	**1329**	0.593148	277	13	177
**4592**	0.633047	295	12	159					

**SA**: The number of bits in both **TTT** and the target. **SB**: The number of bits in the target but not **TTT**. **SC**: The number of bits in **TTT** but not the target.

**Table 2:** Molecular structural properties of the investigated compounds and **TTT**.

Compound	ALog p	M. Wt	HBA	HBD	Rotatable Bonds	Rings	Aromatic Rings	MFPSA	Minimum Distance
**126**	2.73	272.30	4	2	2	3	2	0.22	0.731
**165**	2.84	270.28	4	1	2	3	2	0.211	0.731
**2579**	2.88	268.26	4	1	2	3	2	0.215	0.730
**182**	2.71	256.30	3	1	2	3	2	0.151	0.718
**189**	2.41	267.28	3	1	1	4	3	0.216	0.696
**2203**	3.24	252.27	2	2	0	4	3	0.244	0.661
**204**	4.18	270.32	3	1	2	3	2	0.139	0.660
**164**	3.46	272.30	4	2	2	3	2	0.22	0.659
**3395**	3.55	298.33	4	2	4	3	2	0.229	0.656
**181**	3.18	300.35	4	1	3	3	2	0.153	0.652
**2202**	3.48	236.27	1	1	0	4	3	0.167	0.649
**2137**	4.14	266.33	2	2	2	3	2	0.145	0.619
**3396**	3.64	284.35	3	2	4	3	2	0.173	0.587
**212**	3.21	265.26	3	1	1	4	3	0.207	0.556
**211**	3.21	265.26	3	1	1	4	3	0.207	0.556
**213**	3.43	279.29	3	0	2	4	3	0.148	0.545
**2197**	3.31	323.39	4	2	6	3	3	0.21	0.545
**1875**	4.69	264.32	2	2	1	3	3	0.147	0.502
**3421**	3.01	281.31	3	1	2	4	3	0.157	0.493
**3412**	3.09	228.24	3	3	2	2	2	0.264	0.843
**2982**	3.29	270.32	3	2	6	2	2	0.204	0.822
**166**	2.63	242.23	4	1	2	3	2	0.245	0.816
**3323**	3.14	222.24	2	0	1	3	2	0.123	0.815
**197**	3.29	262.31	1	0	2	4	4	0.116	0.811
**215**	2.69	223.23	3	0	0	4	3	0.154	0.809
**2981**	4.37	252.31	2	1	5	2	2	0.139	0.807
**198**	2.32	257.28	4	2	3	3	2	0.196	0.788
**3469**	2.46	182.22	1	1	0	3	3	0.154	0.785
**3444**	3.04	268.26	4	1	1	3	2	0.241	0.781
**2578**	3.20	270.28	4	2	4	2	2	0.239	0.774
**1330**	3.20	270.28	4	2	4	2	2	0.239	0.774
**1331**	3.20	270.28	4	2	4	2	2	0.239	0.774
**2195**	4.00	338.35	5	3	3	3	2	0.257	0.772
**3040**	4.83	350.41	4	2	4	3	2	0.185	0.768
**4598**	2.86	298.29	5	1	3	3	2	0.22	0.761
**3518**	2.99	226.27	2	1	1	3	2	0.133	0.757
**168**	2.98	251.24	3	2	0	4	3	0.278	0.754
**2677**	3.30	265.31	3	0	2	3	2	0.107	0.743
**199**	2.73	271.31	4	1	4	3	2	0.14	0.741
**1952**	3.88	230.26	3	2	2	2	2	0.205	0.739
**TTT**	3.65	304.39	2	2	3	3	3	0.171	

**Table 3:** The calculated ΔG values of the African metabolites and **TTT**.

Compound	ΔG	Compound	ΔG
**126**	−10.70	**2982**	−11.85
**165**	−9.81	**166**	−7.96
**2579**	−9.17	**3323**	−10.11
**182**	−12.74	**197**	−9.72
**189**	−8.65	**215**	−13.18
**2203**	−8.14	**2981**	−13.72
**204**	−12.21	**198**	−8.01
**164**	−13.22	**3469**	−5.40
**3395**	−7.62	**3444**	−8.88
**181**	−11.55	**2578**	−9.51
**2202**	−9.22	**1330**	−12.20
**2137**	−9.98	**1331**	−14.09
**3396**	−14.14	**2195**	−16.52
**212**	−12.39	**3040**	−14.25
**211**	−10.35	**4598**	−10.13
**213**	−12.63	**3518**	−7.54
**2197**	−13.10	**168**	−16.41
**1875**	−8.65	**2677**	−9.54
**3421**	−7.76	**199**	−10.79
**3412**	−14.00	**1952**	−12.93
**TTT**	−9.30		

**Table 4:** Predicted ADMET descriptors for the African metabolites and the reference.

Compound	BBB Level ^a^	HIA ^b^	Aq ^c^	CYP2D6 ^d^	PPB ^e^
**164**	2	0	3	f	t
**181**	1	0	2	t	t
**182**	1	0	3	t	t
**204**	1	0	2	t	t
**212**	1	0	2	f	t
**213**	1	0	2	f	t
**215**	1	0	2	t	t
**1330**	2	0	3	f	t
**1331**	2	0	3	f	t
**1952**	1	0	3	f	t
**2195**	2	0	2	t	t
**2197**	2	0	2	t	t
**2981**	1	0	2	f	t
**2982**	2	0	3	t	t
**3040**	1	0	2	f	t
**3396**	1	0	3	f	t
**3412**	2	0	3	f	t
Remdesivir	4	3	3	f	f

^a^ BBB, Ability to pass the blood-brain barrier, 1 is high, 2 is medium, 3 is low, 4 is very low. ^b^ HIA, human intestinal absorption level, 0 is good, 1 is moderate, 2 is poor, and 3 is very poor. ^c^ Aq, Aqueous solubility level, 0 is extremely low, 1 is very low, 2 is low, 3 is good, and 4 is optimal. ^d^ CYP2D6, inhibition of CYP2D6 enzyme, t is an inhibitor, f is a non-inhibitor. ^e^ PPB, f means less than 90%, t means more than 90%.

**Table 5:** Toxicity properties of filtered African metabolites and the reference.

Compound	FDA Rat Carcinogenicity	TD_50_ (Rat) ^a^	MTD ^b^	Rat Oral LD_50_^b^	LOAEL ^b^	Ocular Irritancy	Skin Irritancy
Hippacine (**164**)	Not carcinogenic	63.019	0.285	0.441	0.052	Mild	None
Naamine D (**2197**)	Not carcinogenic	4.022	0.086	2.440	0.015	Moderate	None
(±)-Enterofuran (**3412**)	Carcinogenic	87.484	0.690	2.483	0.089	Severe	None
Daphnelone (**2982**)	Carcinogenic	184.723	0.829	0.646	0.173	Severe	None
4,2′-dihydroxy-4′-methoxychalcone (**1330**)	Not carcinogenic	259.532	0.320	1.010	0.060	None	None
2′,5′-dihydroxy-4-methoxychalcone (**1331**)	Not carcinogenic	259.532	0.320	1.010	0.060	Mild	None
Wighteone (**2195**)	Not carcinogenic	42.573	0.525	0.962	0.053	Severe	None
Remdesivir	Not carcinogenic	9.246	0.235	0.309	0.004	Mild	Mild

^a^ Unit: mg kg^−1^ day^−1^. ^b^ Unit: g. kg^−1^.

## Data Availability

The data presented in this study are available in the main article andthe supplementary materials.

## Supplementary Materials

The detailed methodology of this manuscript can be downloaded at: https://www.mdpi.com/article/10.3390/metabo12111122/s1, (Similarity, fingerprints, docking, and MD simulations).
