# Supplementary material for: The Discovery of Potential SARS-CoV-2 Natural Inhibitors among 4924 African Metabolites Targeting the Papain-like Protease: A Multi-Phase In Silico Approach
Source: Metabolites. 2022 Nov 16;12(11):1122. doi: 10.3390/metabo12111122 (PMC9693093; doi:10.3390/metabo12111122)
Supplement: Supplementary file 1 [file metabolites-12-01122-s001.zip › metabolites-2043551-supplementary.pdf]

Supporting Data

The Discovery of Potential SARS-CoV-2 Natural Inhibitors Among 4924 African Metabolites Targeting The Papain-Like Protease: A Multi-phase *in silico* Approach

Eslam B. Elkaeed[a]*, Mohamed M. Khalifa[b], Bshra A. Alsfouk[c], Aisha A. Alsfouk[c], Abdul-Aziz M.M. El-Attar[d], Ibrahim H. Eissa[b]* and Ahmed M. Metwaly[e,f]*

[a] Department of Pharmaceutical Sciences, College of Pharmacy, AlMaarefa University, Riyadh 13713, Saudi Arabia

[b] Pharmaceutical Medicinal Chemistry & Drug Design Department, Faculty of Pharmacy (Boys), Al-Azhar University, Cairo11884, Egypt

[c] Department of Pharmaceutical Sciences, College of Pharmacy, Princess Nourah bint Abdulrahman University, P.O. Box 84428, Riyadh 11671, Saudi Arabia

[d] Pharmaceutical Analytical Chemistry Department, Faculty of Pharmacy, Al-Azhar University, 11884, Nasr City, Cairo, Egypt

[e] Pharmacognosy and Medicinal Plants Department, Faculty of Pharmacy (Boys), Al-Azhar University, Cairo 11884, Egypt

[f] Biopharmaceutical Products Research Department, Genetic Engineering and Biotechnology Research Institute, City of Scientific Research and Technological Applications (SRTA-City), Alexandria 21934, Egypt

* Correspondence: **:** ametwaly@azhar.edu.eg, ikaeed@mcst.edu.sa

Content

Method	Fingerprint studies	
	Molecular Similarity	
	Docking studies	
	ADMET studies	
	Toxicity studies	
	Molecular dynamics simulations	
Toxicity report		

Method

Preparation of the tested compounds for Molecular Similarity, fingerprints, ADMET, and toxicity studies:

In this protocol, the general-purpose panel was utilized with the activation of the Prepare ligand option. The change ionization was switched on the true option using the Rule based as an ionization method. In Rule based task, we used the carboxylate as an acid ionization. Additionally, the primary, secondary, and tertiary amines were selected as Base ionization. The ionization enumeration option was switched on the one protomer. Under the filter smart option, we selected all options. The false option was selected for tasks Generate tautomers, generate isomers, Fix bad valencies, and parallel processing. The generate coordinates task was switched on the 3D option. Finally, the duplicate structure task was activated on the remove option.

- **Molecular Similarity**

The molecular Similarity of the tested compounds were checked using Discovery studio 4.0. **TTT** was used as a reference molecule. The study calculates the following parameters in a quantitative method

1. Number of rotatable bonds,
2. Number of rings,
3. Number of aromatic rings,
4. Number of hydrogen bond donors,
5. Number of hydrogen bond acceptors,
6. Octanol-water partition coefficient (ALog p),
7. Molecular weight, and
8. Molecular fractional polar surface area (MFPSA).

Running of Molecular Similarity protocol

In this protocol, after compounds preparation, the small molecules panel was utilized with the activation of the library analysis option. Then, the option of find similar molecules by numeric properties was activated. Furthermore, we selected the prepared compounds as the input ligands and GRL0617 as a reference ligand. In addition, what to find task was switched on as most similar molecules (number of similar molecules = 30). The Euclidean distance was selected. Then, the output of the running protocol was visualized to give the output chart of structural similarity.

- **Fingerprints check**

The Fingerprints check of the tested compounds were checked using Discovery studio 4.0. **TTT** was used as a reference molecule. The used fingerprints were based on the presence or absence of some parameters related to type of atoms, in detail,

1. Number of charges,
2. Hybridization,
3. Number of hydrogen bond acceptors,
4. Number of hydrogen bond donors,
5. Number of positive ionizable atoms,
6. Number of negative ionizable atoms,
7. Number of halogen atoms,
8. Number of aromatic rings
9. ALogP (octanol-water partition coefficient)

Running of Fingerprints check protocol

In this protocol, after compounds preparation, the small molecules panel was utilized with the activation of the library analysis option. Then, the option of find similar molecules by Fingerprints was activated. Furthermore, we selected the prepared compounds as the input ligands and GRL0617 as a reference ligand. In addition, what to find task was switched on as most similar molecules (number of similar molecules = 15). The Tanimoto coefficient was selected. Then, the output of the running protocol was visualized to give the output results.

- **Molecular docking**

Protein Preparation:

The crystal structure of SARS- papain-like protease (PDB ID: 3E9S) was obtained from Protein Data Bank (https://www.rcsb.org). At first, the crystal structure of the PLpro complexed with the TTT as a co-crystallized ligand was prepared by removing crystallographic water molecules. Only one chain was retained besides the co-crystallized ligand (TTT). The selected protein chain was protonated using the following setting. The used electrostatic functional form was

GB/VI with a distance cut-off of 15 Å. The used value of the dielectric constant was 2 with an 80 dielectric constant of the used solvent. The used Van der Waals functional form was 800R3 with a distance cut-off of 10 Å. Then, the energy of the protein chain was minimized using Hamiltonian AM1 implanted in Molecular Operating Environment (MOE 2019 and MMFF94x (Merck molecular force field) for structural optimization. Next, the active site of the target protein was defined for ligand docking and redocking (in case of validation of docking protocol). The active site of the protein was identified as the residues that fall within the 5 Å distance from the perimeter of the co-crystallized ligand.

Ligand Preparation: 2D structures of the synthesized compounds and the standard compound, sorafenib were drawn using ChemBioDraw Ultra 14.0 and saved in MDL-SD file format. The 3D structures of the ligands were protonated, and the structures were optimized by energy minimization using MM2 force-field and 10000 iteration steps of 2 fs. The conformationally optimized ligands were used for docking studies.

Docking Setup and Validation of Docking Protocol: The protein-ligand docking studies were carried out using MOE version 2019. Validation of the docking protocol was carried out by redocking the co-crystallized reference ligand (TTT) against the isolated pocket of PLpro. The docking protocol was validated by comparing the heavy atoms RMSD value of the re-docked ligand pose with the corresponding co-crystallized reference ligand structure.

The docking setup for the tested compounds was established according to the protocol followed in the validation step. For each docking run, 30 docked solutions were generated using ASE for scoring function and rigid receptor for refinement. The pose with ideal binding mode was selected for further investigations. The docking results were visualized using Discovery Studio (DS) 4.0. Analysis of the docking results was carried out by comparing the interactions and docking score obtained for the docked ligands with that of the re-docked reference molecule (TTT).

- **ADMET studies**

ADMET descriptors (absorption, distribution, metabolism, excretion and toxicity) of the compounds were determined using Discovery studio 4.0. **Remdesivir** was used as a reference molecule. At first, the CHARMM force field was applied then the tested compounds were prepared and minimized according to the preparation of small molecule protocol. The ADMET descriptors that applied including models for

1. Human intestinal absorption,
2. Aqueous solubility,
3. Blood brain barrier penetration,
4. Plasma protein binding,
5. Cytochrome P450 2D6 inhibition, and
6. Hepatotoxicity.

The examined molecules filtered to select those molecules that meet the rules specified by the set of selected SMARTS® rules.

.

Running of ADMET protocol

In this protocol, after compounds preparation, the small molecules panel was utilized with the activation of the ADMET descriptors option. Then, we selected the prepared compounds as the input ligands. Further, all the ADMET parameters (aqueous solubility, Blood brain barrier, intestinal absorption, CYP2D6, and plasma protein binding) were selected. Then, the output of the running protocol was visualized to give the ADMET chart.

- **Toxicity studies**

The toxicity parameters of the synthesized compounds were calculated using Discovery studio 4.0. Remdesivir was used as a reference molecule. Then different parameters were calculated from the toxicity prediction (extensible) protocol (TOPKAT) that evaluated the examined compounds' performance in experimental assays and animal models. TOPKAT computed and validated assessments of the toxic and environmental effects of the examined chemicals solely from their molecular structure. TOPKAT employs robust and cross-validated Quantitative Structure Toxicity Relationship (QSTR) models for assessing various measures of toxicity and utilizing the patented Optimal Predictive Space validation method to assist in interpreting the results.

The predicted models are

1. FDA rat carcinogenicity test,
2. Carcinogenic potentiality TD_{50} (the median toxic dose of a substance in which toxicity occurs in 50% of a species),
3. Maximum tolerated dose (MTD) in rats,

4. Oral LD_{50} in rats (the amount that kills 50% of test animals),
5. Chronic LOAEL (Lowest-observed-adverse-effect level) in rats,
6. Ocular irritancy and
7. Skin irritancy

Running of Toxicity protocol

In this protocol, after compounds preparation, the small molecules panel was utilized with the activation of the toxicity prediction (extensible) option. Then, we selected the prepared compounds as the input ligands. Further, the different toxicity models were selected from the model panel. The similarity search task was activated to be true. The detailed report task was switched on as a PDF file. Then, the output of the running protocol was visualized to give the toxicity PDF report.

- **Molecular Dynamics (MD) Simulations**

The system was prepared using the web-based CHARMM-GUI interface with the CHARMM36 force field. All the simulations were done using the NAMD 2.13 package. The TIP3P explicit solvation model was used, and the periodic boundary conditions were set with a dimension of the dimensions in x, y, and z, respectively. The parameters for the top docking results were generated using the CHARMM general force field Afterward, the system was neutralized using (Cl^-/Na^+) ions. The MD protocols involved minimization, equilibration, and production. a 2 *fs* time step of integration was chosen for all MD simulations, the equilibration was carried in the canonical (*NVT*) ensemble, while the isothermal–isobaric (*NPT*) ensemble was for the production. Through the 100 *ns* of MD production, the pressure was set at 1 atm using the Nose´–Hoover Langevin piston barostat with a Langevin piston decay of 0.05 *ps* and a period of 0.1 *ps*. The temperature was set at 298.15 K using the Langevin thermostat. A distance cutoff of 12.0 Å was applied to short-range nonbonded interactions with a pair list distance of 16 Å, and Lennard Jones interactions were smoothly truncated at 8.0 Å. Long-range electrostatic interactions were treated using the particle-mesh Ewald (PME) method, where a grid spacing of 1.0 Å was used for all simulation cells. All covalent bonds involving hydrogen atoms were constrained using the SHAKE algorithm[13]. For consistency, we have applied the same protocol for all MD simulations.

Binding Energy Calculations

The one-average molecular mechanics generalized Born surface area (MM/GBSA) approach implemented in the MOLAICAL code was used for the relative binding energy calculations, in which the ligand (L) binds to the protein receptor (R) to form the complex (RL),

$$\Delta G_{bind} = \Delta G_{RL} - \Delta G_R - \Delta G_L$$

which can be represented by contributions of different interactions,

$$\Delta G_{bind} = \Delta H - T\Delta S = \Delta E_{MM} + \Delta G_{Sol} - T\Delta S$$

where the changes in the gas phase molecular mechanics (ΔE_{MM}), solvation Gibbs energy (ΔG_{Sol}), and conformational entropy $(-T\Delta S)$ are determined as follows: ΔE_{MM} is the sum of the changes in the electrostatic energies ΔE_{ele}, the van der Waals energies ΔE_{vdW}, and the internal energies ΔE_{int} (bonded interactions); ΔG_{Sol} is the total of both the polar solvation (calculated using the generalized Born model) and the nonpolar solvation (the solvent-accessible surface area) and $-T\Delta S$ is calculated by the normal mode analysis. The solvent dielectric constant of 78.5 and the surface tension constant of 0.03012 kJ mol^{-1} Å^{2} were used for MM/GBSA calculations.

Toxicity Report

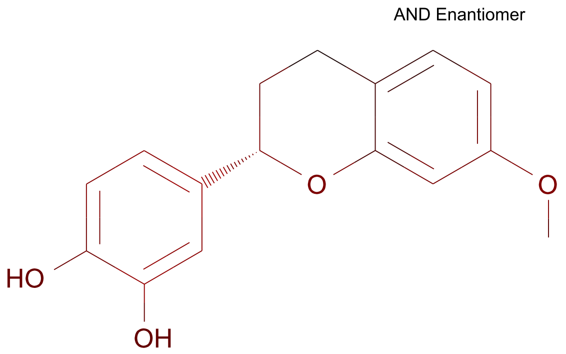

$C_{16}H_{16}O_4$
Molecular Weight: 272.296
ALogP: 3.456
Rotatable Bonds: 2
Acceptors: 4
Donors: 2

Model Prediction

Prediction: Toxic
Probability: 0.774
Enrichment: 1.47
Bayesian Score: 5.93
Mahalanobis Distance: 9.17
Mahalanobis Distance p-value: 0.13

Prediction: Positive if the Bayesian score is above the estimated best cutoff value from minimizing the false positive and false negative rate.
Probability: The esimated probability that the sample is in the positive category. This assumes that the Bayesian score follows a normal distribution and is different from the prediction using a cutoff.
Enrichment: An estimate of enrichment, that is, the increased likelihood (versus random) of this sample being in the category.
Bayesian Score: The standard Laplacian-modified Bayesian score.
Mahalanobis Distance: The Mahalanobis distance (MD) is the distance to the center of the training data. The larger the MD, the less trustworthy the prediction.
Mahalanobis Distance p-value: The p-value gives the fraction of training data with an MD greater than or equal to the one for the given sample, assuming normally distributed data. The smaller the p-value, the less trustworthy the prediciton. For highly non-normal X properties (e.g., fingerprints), the MD p-value is wildly inaccurate.

Structural Similar Compounds

Name	Diflunisal	Bisphenol a	D&C Yellow 8
Structure	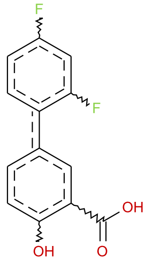	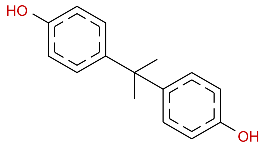	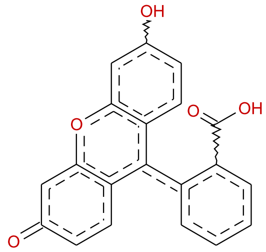
Actual Endpoint	Toxic	Non-Toxic	Non-Toxic
Predicted Endpoint	Toxic	Non-Toxic	Non-Toxic
Distance	0.543	0.579	0.593
Reference	Oyo Yakuri 17(4):551-557; 1979	Fundam Appl Toxicol 8:571-582; 1987	Food Chem Toxicol 24:819-823; 1986

Model Applicability

Unknown features are fingerprint features in the query molecule, but not found or appearing too infrequently in the training set.

1. All properties and OPS components are within expected ranges.

Feature Contribution

Top features for positive contribution				
**Fingerprint**	**Bit/Smiles**	**Feature Structure**	**Score**	**Toxic in training set**
SCFP_6	1702724181	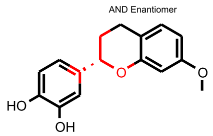 [*]C[C@H](O[*])[c](:[*]):[*]	0.558	9 out of 9

SCFP_6	-561151481	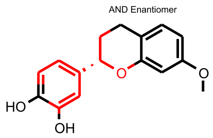<br>[*]C[C@H](O[*])[c]1:[cH]:[cH]:[*]:[c]([*]):[cH]:1	0.523	6 out of 6
SCFP_6	2116304939	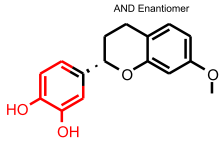<br>O[c]1:[cH]:[*]:[cH]:[cH]:[c]:1O	0.504	5 out of 5
**Top Features for negative contribution**				
**Fingerprint**	**Bit/Smiles**	**Feature Structure**	**Score**	**Toxic in training set**
SCFP_6	-1849867720	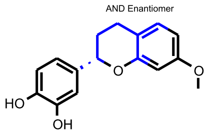<br>[*][C@H]1[*][c](:[*]):[c](CC1):[cH]:[*]	-0.189	2 out of 5
SCFP_6	-1379148975	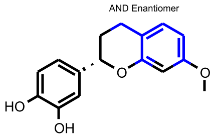<br>[*]C[c]1:[cH]:[cH]:[c]([*]):[*]:[c]:1[*]	0	29 out of 58
SCFP_6	2109165795	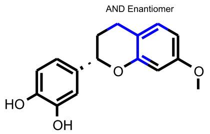<br>[*]C[c](:[cH]:[*]):[c]([*]):[*]	0	40 out of 77

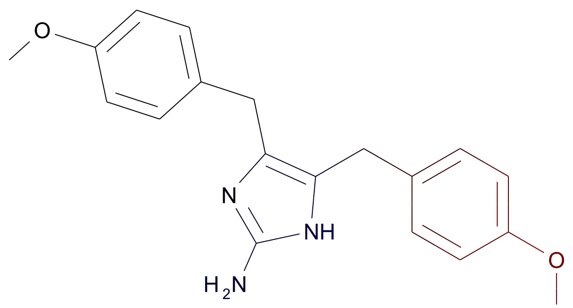

$C_{19}H_{21}N_3O_2$
Molecular Weight: 323.389
ALogP: 3.305
Rotatable Bonds: 6
Acceptors: 4
Donors: 2

Model Prediction

Prediction: Non-Toxic
Probability: 0.534
Enrichment: 1.02
Bayesian Score: -0.364
Mahalanobis Distance: 8.85
Mahalanobis Distance p-value: 0.224

Prediction: Positive if the Bayesian score is above the estimated best cutoff value from minimizing the false positive and false negative rate.
Probability: The esimated probability that the sample is in the positive category. This assumes that the Bayesian score follows a normal distribution and is different from the prediction using a cutoff.
Enrichment: An estimate of enrichment, that is, the increased likelihood (versus random) of this sample being in the category.
Bayesian Score: The standard Laplacian-modified Bayesian score.
Mahalanobis Distance: The Mahalanobis distance (MD) is the distance to the center of the training data. The larger the MD, the less trustworthy the prediction.
Mahalanobis Distance p-value: The p-value gives the fraction of training data with an MD greater than or equal to the one for the given sample, assuming normally distributed data. The smaller the p-value, the less trustworthy the prediciton. For highly non-normal X properties (e.g., fingerprints), the MD p-value is wildly inaccurate.

Structural Similar Compounds

Name	Clebopride Malate	Amsacrine	Benomyl
Structure	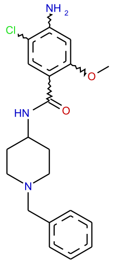	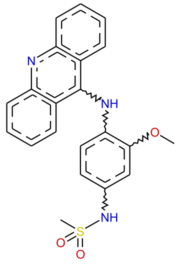	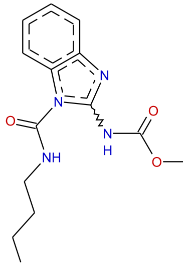
Actual Endpoint	Non-Toxic	Toxic	Toxic
Predicted Endpoint	Non-Toxic	Toxic	Toxic
Distance	0.542	0.566	0.570
Reference	Kiso to Rinsho 16:5649-5660; 1982	Fundam Appl Toxicol 7(2):214-20; 1986	J Toxicol Environ Health 17:405-417; 1986

Model Applicability

Unknown features are fingerprint features in the query molecule, but not found or appearing too infrequently in the training set.

1. All properties and OPS components are within expected ranges.

Feature Contribution

Top features for positive contribution

Fingerprint	Bit/Smiles	Feature Structure	Score	Toxic in training set
SCFP_6	-1849095515	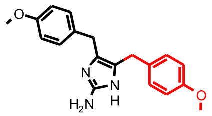 [*]C[c]1:[cH]:[cH]:[c](OC):[cH]:[c]:1[*]	0.478	4 out of 4

SCFP_6	1237755852	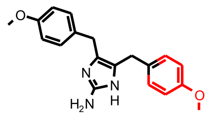<br>[*][c]1:[*]:[cH]:[cH]<br>:[c](OC):[cH]:1	0.453	8 out of 9
SCFP_6	591469355	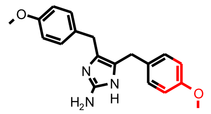<br>[*]:[cH]:[c](OC):[cH]<br>:[*]	0.411	10 out of 12
**Top Features for negative contribution**				
**Fingerprint**	**Bit/Smiles**	**Feature Structure**	**Score**	**Toxic in training set**
SCFP_6	2109374332	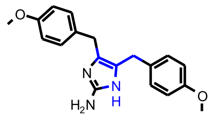<br>[*]C[c]1:[nH]:[*]:[*]<br>:[c]:1[*]	-0.446	3 out of 10
SCFP_6	-502559736	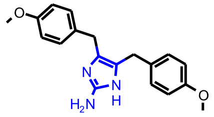<br>[*][c]1:[nH]:[c](N):n<br>:[c]:1[*]	-0.422	0 out of 1
SCFP_6	384920865	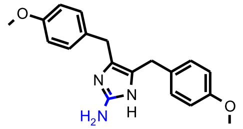<br>[*]:[c](:[*])N	-0.33	5 out of 14

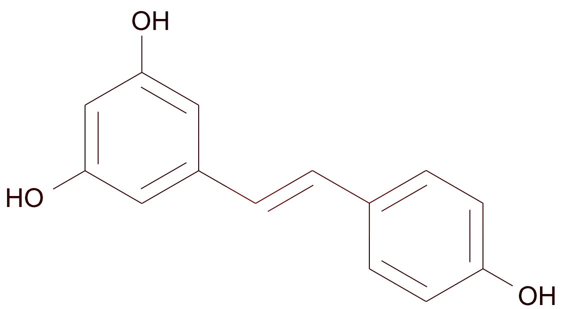

$C_{14}H_{12}O_3$
Molecular Weight: 228.243
ALogP: 3.09
Rotatable Bonds: 2
Acceptors: 3
Donors: 3

Model Prediction

Prediction: Toxic
Probability: 0.655
Enrichment: 1.25
Bayesian Score: 2.76
Mahalanobis Distance: 7.72
Mahalanobis Distance p-value: 0.734

Prediction: Positive if the Bayesian score is above the estimated best cutoff value from minimizing the false positive and false negative rate.
Probability: The esimated probability that the sample is in the positive category. This assumes that the Bayesian score follows a normal distribution and is different from the prediction using a cutoff.
Enrichment: An estimate of enrichment, that is, the increased likelihood (versus random) of this sample being in the category.
Bayesian Score: The standard Laplacian-modified Bayesian score.
Mahalanobis Distance: The Mahalanobis distance (MD) is the distance to the center of the training data. The larger the MD, the less trustworthy the prediction.
Mahalanobis Distance p-value: The p-value gives the fraction of training data with an MD greater than or equal to the one for the given sample, assuming normally distributed data. The smaller the p-value, the less trustworthy the prediciton. For highly non-normal X properties (e.g., fingerprints), the MD p-value is wildly inaccurate.

Structural Similar Compounds

Name	Diflunisal	Bisphenol a	Guanabenz
Structure	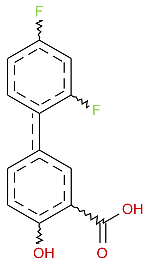	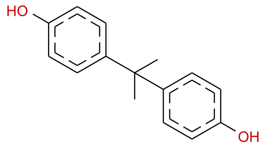	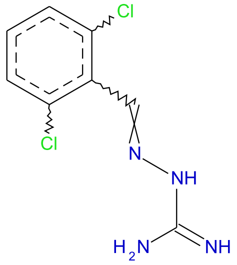
Actual Endpoint	Toxic	Non-Toxic	Toxic
Predicted Endpoint	Toxic	Non-Toxic	Toxic
Distance	0.560	0.595	0.611
Reference	Oyo Yakuri 17(4):551-557; 1979	Fundam Appl Toxicol 8:571-582; 1987	Journal of Toxic Sciences 11:107-119; 1982

Model Applicability

Unknown features are fingerprint features in the query molecule, but not found or appearing too infrequently in the training set.

1. All properties and OPS components are within expected ranges.

Feature Contribution

Top features for positive contribution				
**Fingerprint**	**Bit/Smiles**	**Feature Structure**	**Score**	**Toxic in training set**
SCFP_6	-538866216	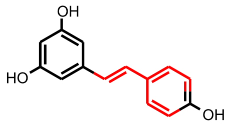 [*]\C=C\[c]1:[cH]:[cH]:[*]:[cH]:[cH]:1	0.478	4 out of 4

SCFP_6	2033992841	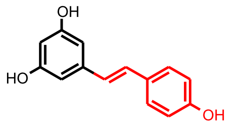<br>[*]\C=C\[c]1:[cH]:[cH<br>]:[c](O):[cH]:[cH]:1	0.441	3 out of 3
SCFP_6	-1971137145	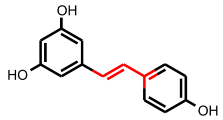<br>[*]\C=C\[c](:[*]):[*]	0.431	7 out of 8
**Top Features for negative contribution**				
**Fingerprint**	**Bit/Smiles**	**Feature Structure**	**Score**	**Toxic in training set**
SCFP_6	1112262477	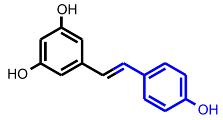<br>[*]=C[c]1:[cH]:[cH]:[<br>c](O):[cH]:[cH]:1	-0.422	0 out of 1
SCFP_6	-496409612	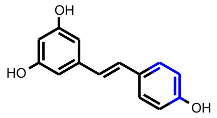<br>[*][c](:[*]):[cH]:[cH<br>]:[*]	0	82 out of 163
SCFP_6	611156666	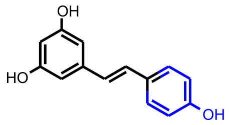<br>O[c]1:[cH]:[cH]:[*]:[<br>cH]:[cH]:1	0	6 out of 11

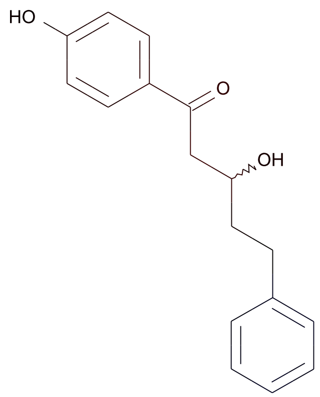

$C_{17}H_{18}O_3$
Molecular Weight: 270.323
ALogP: 3.293
Rotatable Bonds: 6
Acceptors: 3
Donors: 2

Model Prediction

Prediction: Toxic
Probability: 0.565
Enrichment: 1.07
Bayesian Score: 0.453
Mahalanobis Distance: 10.6
Mahalanobis Distance p-value: 0.00337

Prediction: Positive if the Bayesian score is above the estimated best cutoff value from minimizing the false positive and false negative rate.
Probability: The esimated probability that the sample is in the positive category. This assumes that the Bayesian score follows a normal distribution and is different from the prediction using a cutoff.
Enrichment: An estimate of enrichment, that is, the increased likelihood (versus random) of this sample being in the category.
Bayesian Score: The standard Laplacian-modified Bayesian score.
Mahalanobis Distance: The Mahalanobis distance (MD) is the distance to the center of the training data. The larger the MD, the less trustworthy the prediction.
Mahalanobis Distance p-value: The p-value gives the fraction of training data with an MD greater than or equal to the one for the given sample, assuming normally distributed data. The smaller the p-value, the less trustworthy the prediciton. For highly non-normal X properties (e.g., fingerprints), the MD p-value is wildly inaccurate.

Structural Similar Compounds

Name	Parbendazole	Bufexamac	Flufenamic Acid
Structure	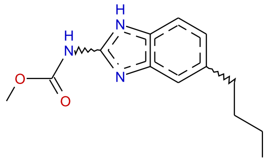	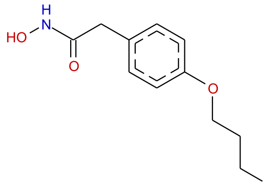	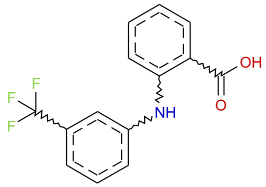
Actual Endpoint	Toxic	Toxic	Non-Toxic
Predicted Endpoint	Toxic	Toxic	Non-Toxic
Distance	0.502	0.539	0.550
Reference	Cornell Vet 64:Suppl 4:97-103; 1974	Arzneimittelforschung 20(4):565-9; 1970	Kiso to Rinsho 13:3302-3313; 1979

Model Applicability

Unknown features are fingerprint features in the query molecule, but not found or appearing too infrequently in the training set.

1. All properties and OPS components are within expected ranges.

Feature Contribution

Top features for positive contribution

Fingerprint	Bit/Smiles	Feature Structure	Score	Toxic in training set
SCFP_6	-424515134	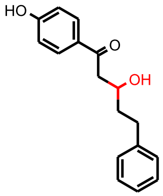 [*]C([*])O	0.275	39 out of 56

SCFP_6	-1462335598	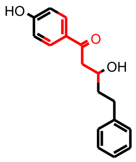 [*]C([*])CC(=O)[c](:[cH]:[*]):[cH]:[*]	0.271	1 out of 1
SCFP_6	-542118530	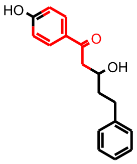 [*]CC(=O)[c]1:[cH]:[cH]:[*]:[cH]:[cH]:1	0.271	1 out of 1

Top Features for negative contribution

Fingerprint	Bit/Smiles	Feature Structure	Score	Toxic in training set
SCFP_6	1112262477	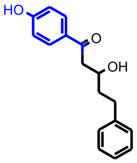 [*]=C[c]1:[cH]:[cH]:[c](O):[cH]:[cH]:1	-0.422	0 out of 1
SCFP_6	-1379591900	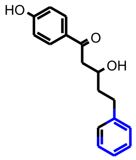 [*][c]1:[*]:[cH]:[cH]:[cH]:[cH]:1	-0.282	33 out of 84
SCFP_6	-1849867720	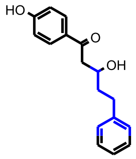 [*][C@H]1[*][c](:[*]):[c](CC1):[cH]:[*]	-0.189	2 out of 5

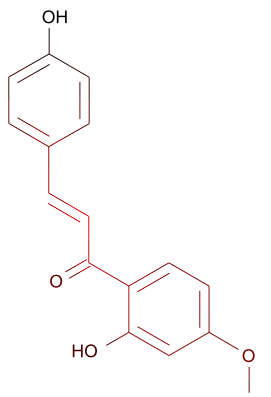

$C_{16}H_{14}O_4$
Molecular Weight: 270.28
ALogP: 3.201
Rotatable Bonds: 4
Acceptors: 4
Donors: 2

Model Prediction

Prediction: Toxic
Probability: 0.789
Enrichment: 1.5
Bayesian Score: 6.38
Mahalanobis Distance: 9.63
Mahalanobis Distance p-value: 0.0515

Prediction: Positive if the Bayesian score is above the estimated best cutoff value from minimizing the false positive and false negative rate.
Probability: The esimated probability that the sample is in the positive category. This assumes that the Bayesian score follows a normal distribution and is different from the prediction using a cutoff.
Enrichment: An estimate of enrichment, that is, the increased likelihood (versus random) of this sample being in the category.
Bayesian Score: The standard Laplacian-modified Bayesian score.
Mahalanobis Distance: The Mahalanobis distance (MD) is the distance to the center of the training data. The larger the MD, the less trustworthy the prediction.
Mahalanobis Distance p-value: The p-value gives the fraction of training data with an MD greater than or equal to the one for the given sample, assuming normally distributed data. The smaller the p-value, the less trustworthy the prediciton. For highly non-normal X properties (e.g., fingerprints), the MD p-value is wildly inaccurate.

Structural Similar Compounds

Name	Benomyl	Parbendazole	Diflunisal
Structure	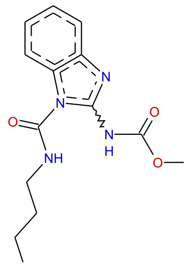	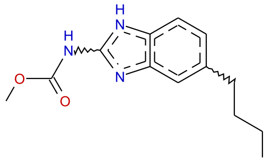	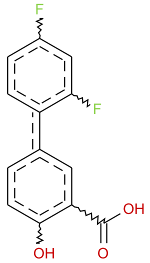
Actual Endpoint	Toxic	Toxic	Toxic
Predicted Endpoint	Toxic	Toxic	Toxic
Distance	0.533	0.545	0.553
Reference	J Toxicol Environ Health 17:405-417; 1986	Cornell Vet 64:Suppl 4:97-103; 1974	Oyo Yakuri 17(4):551-557; 1979

Model Applicability

Unknown features are fingerprint features in the query molecule, but not found or appearing too infrequently in the training set.

1. All properties and OPS components are within expected ranges.

Feature Contribution

Top features for positive contribution

Fingerprint	Bit/Smiles	Feature Structure	Score	Toxic in training set
SCFP_6	-1977229858	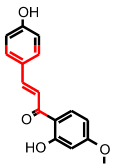 [*]C(=[*])\C=C\[c](:[cH]:[*]):[cH]:[*]	0.478	4 out of 4

SCFP_6	-538866216	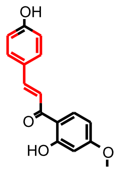<br>[*]\C=C\[c]1:[cH]:[cH]:[*]:[cH]:[cH]:1	0.478	4 out of 4
SCFP_6	1237755852	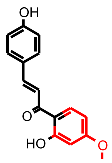<br>[*][c]1:[*]:[cH]:[cH]:[c](OC):[cH]:1	0.453	8 out of 9

Top Features for negative contribution

**Fingerprint**	**Bit/Smiles**	**Feature Structure**	**Score**	**Toxic in training set**
SCFP_6	1112262477	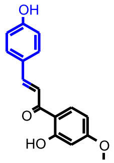<br>[*]=C[c]1:[cH]:[cH]:[c](O):[cH]:[cH]:1	-0.422	0 out of 1
SCFP_6	387787917	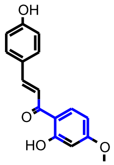<br>[*][c]1:[*]:[cH]:[c](C=[*]):[cH]:[cH]:1	0	22 out of 41
SCFP_6	1311071855	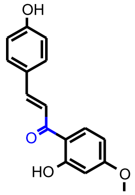<br>[*]C(=O)[*]	0	72 out of 141

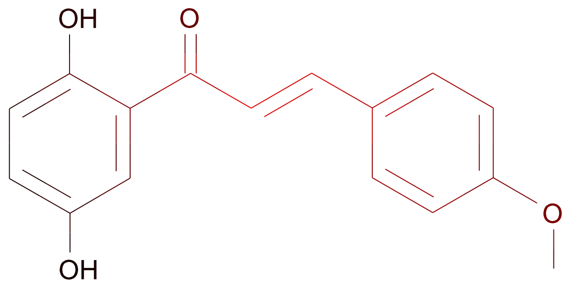

$C_{16}H_{14}O_4$
Molecular Weight: 270.28
ALogP: 3.201
Rotatable Bonds: 4
Acceptors: 4
Donors: 2

Model Prediction

Prediction: Toxic
Probability: 0.797
Enrichment: 1.52
Bayesian Score: 6.61
Mahalanobis Distance: 9.63
Mahalanobis Distance p-value: 0.0515

Prediction: Positive if the Bayesian score is above the estimated best cutoff value from minimizing the false positive and false negative rate.
Probability: The esimated probability that the sample is in the positive category. This assumes that the Bayesian score follows a normal distribution and is different from the prediction using a cutoff.
Enrichment: An estimate of enrichment, that is, the increased likelihood (versus random) of this sample being in the category.
Bayesian Score: The standard Laplacian-modified Bayesian score.
Mahalanobis Distance: The Mahalanobis distance (MD) is the distance to the center of the training data. The larger the MD, the less trustworthy the prediction.
Mahalanobis Distance p-value: The p-value gives the fraction of training data with an MD greater than or equal to the one for the given sample, assuming normally distributed data. The smaller the p-value, the less trustworthy the prediciton. For highly non-normal X properties (e.g., fingerprints), the MD p-value is wildly inaccurate.

Structural Similar Compounds

Name	Benomyl	Parbendazole	Diflunisal
Structure	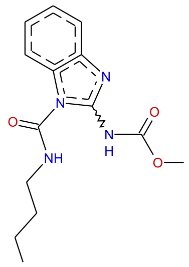	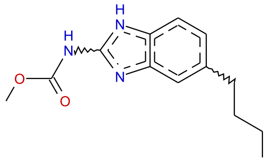	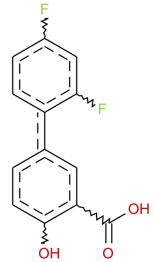
Actual Endpoint	Toxic	Toxic	Toxic
Predicted Endpoint	Toxic	Toxic	Toxic
Distance	0.534	0.546	0.555
Reference	J Toxicol Environ Health 17:405-417; 1986	Cornell Vet 64:Suppl 4:97-103; 1974	Oyo Yakuri 17(4):551-557; 1979

Model Applicability

Unknown features are fingerprint features in the query molecule, but not found or appearing too infrequently in the training set.

1. All properties and OPS components are within expected ranges.

Feature Contribution

Top features for positive contribution				
Fingerprint	Bit/Smiles	Feature Structure	Score	Toxic in training set
SCFP_6	-1977229858	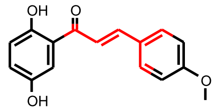 [*]C(=[*])\C=C\[c](:[cH]:[*]):[cH]:[*]	0.478	4 out of 4

SCFP_6	-538866216	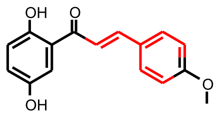 [*]\C=C\[c]1:[cH]:[cH]:[*]:[cH]:[cH]:1	0.478	4 out of 4
SCFP_6	1237755852	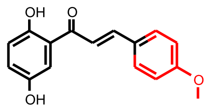 [*][c]1:[*]:[cH]:[cH]:[c](OC):[cH]:1	0.453	8 out of 9
**Top Features for negative contribution**				
**Fingerprint**	**Bit/Smiles**	**Feature Structure**	**Score**	**Toxic in training set**
SCFP_6	611156666	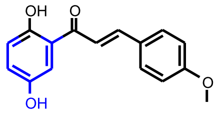 O[c]1:[cH]:[cH]:[*]:[cH]:[cH]:1	0	6 out of 11
SCFP_6	1	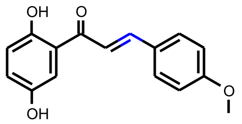 [*]C=[*]	0	90 out of 173
SCFP_6	3	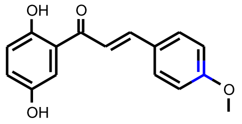 [*][c](:[*]):[*]	0	92 out of 181

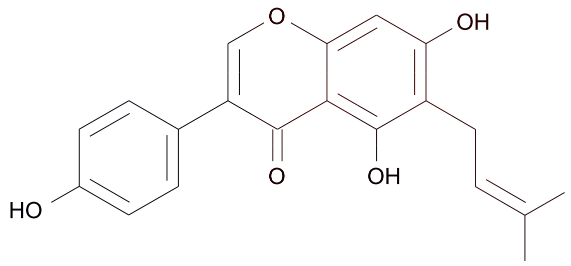

$C_{20}H_{18}O_5$
Molecular Weight: 338.354
ALogP: 3.997
Rotatable Bonds: 3
Acceptors: 5
Donors: 3

Model Prediction

Prediction: Toxic
Probability: 0.624
Enrichment: 1.19
Bayesian Score: 1.98
Mahalanobis Distance: 10.3
Mahalanobis Distance p-value: 0.00863

Prediction: Positive if the Bayesian score is above the estimated best cutoff value from minimizing the false positive and false negative rate.
Probability: The esimated probability that the sample is in the positive category. This assumes that the Bayesian score follows a normal distribution and is different from the prediction using a cutoff.
Enrichment: An estimate of enrichment, that is, the increased likelihood (versus random) of this sample being in the category.
Bayesian Score: The standard Laplacian-modified Bayesian score.
Mahalanobis Distance: The Mahalanobis distance (MD) is the distance to the center of the training data. The larger the MD, the less trustworthy the prediction.
Mahalanobis Distance p-value: The p-value gives the fraction of training data with an MD greater than or equal to the one for the given sample, assuming normally distributed data. The smaller the p-value, the less trustworthy the prediciton. For highly non-normal X properties (e.g., fingerprints), the MD p-value is wildly inaccurate.

Structural Similar Compounds

Name	D&C Yellow 8	Chenodiol	Ochratoxin a
Structure	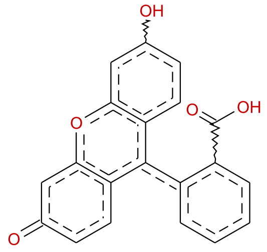	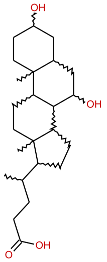	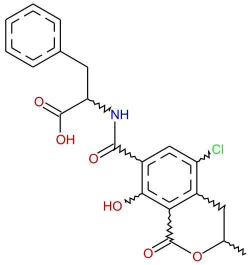
Actual Endpoint	Non-Toxic	Toxic	Toxic
Predicted Endpoint	Non-Toxic	Toxic	Toxic
Distance	0.579	0.619	0.626
Reference	Food Chem Toxicol 24:819-823; 1986	Arch Int Pharm 246:149-158; 1980	Toxicol Appl Pharmacol 37(2):331-8; 1976

Model Applicability

Unknown features are fingerprint features in the query molecule, but not found or appearing too infrequently in the training set.

1. All properties and OPS components are within expected ranges.

Feature Contribution

Top features for positive contribution

Fingerprint	Bit/Smiles	Feature Structure	Score	Toxic in training set
SCFP_6	279586273	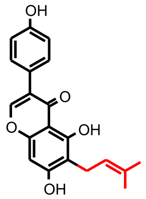 [*]CC=C(C)C	0.381	2 out of 2

SCFP_6	130348166	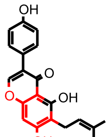 [*]O[c]1:[cH]:[*]:[c]([*]):[c](O[*]):[cH]:1	0.369	5 out of 6
SCFP_6	470101049	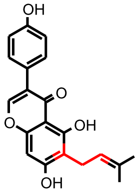 [*]=CC[c](:[*]):[*]	0.322	4 out of 5

Top Features for negative contribution

Fingerprint	Bit/Smiles	Feature Structure	Score	Toxic in training set
SCFP_6	1112262477	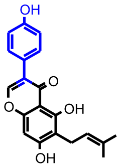 [*]=C[c]1:[cH]:[cH]:[c](O):[cH]:[cH]:1	-0.422	0 out of 1
SCFP_6	136627117	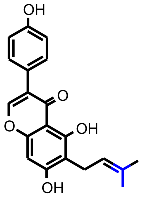 [*]C(=[*])C	-0.0885	10 out of 21
SCFP_6	611156666	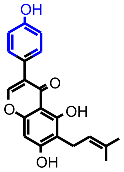 O[c]1:[cH]:[cH]:[*]:[cH]:[cH]:1	0	6 out of 11

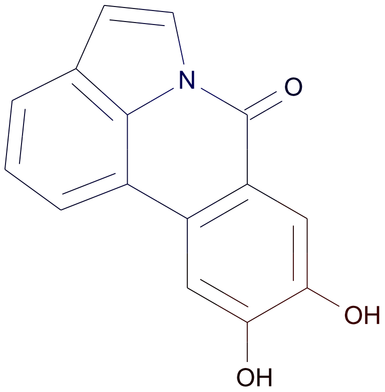

$C_{15}H_9NO_3$
Molecular Weight: 251.237
ALogP: 2.982
Rotatable Bonds: 0
Acceptors: 3
Donors: 2

Model Prediction

Prediction: Non-Toxic
Probability: 0.482
Enrichment: 0.916
Bayesian Score: -1.82
Mahalanobis Distance: 8.02
Mahalanobis Distance p-value: 0.596

Prediction: Positive if the Bayesian score is above the estimated best cutoff value from minimizing the false positive and false negative rate.
Probability: The esimated probability that the sample is in the positive category. This assumes that the Bayesian score follows a normal distribution and is different from the prediction using a cutoff.
Enrichment: An estimate of enrichment, that is, the increased likelihood (versus random) of this sample being in the category.
Bayesian Score: The standard Laplacian-modified Bayesian score.
Mahalanobis Distance: The Mahalanobis distance (MD) is the distance to the center of the training data. The larger the MD, the less trustworthy the prediction.
Mahalanobis Distance p-value: The p-value gives the fraction of training data with an MD greater than or equal to the one for the given sample, assuming normally distributed data. The smaller the p-value, the less trustworthy the prediciton. For highly non-normal X properties (e.g., fingerprints), the MD p-value is wildly inaccurate.

Structural Similar Compounds

Name	Diflunisal	3;6-Dichloropicolinic Acid	Cyclopiazonic Acid
Structure	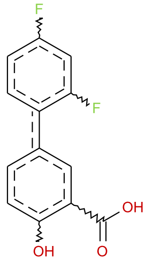	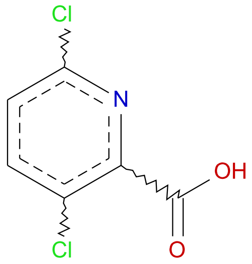	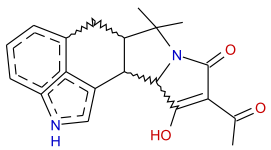
Actual Endpoint	Toxic	Non-Toxic	Non-Toxic
Predicted Endpoint	Toxic	Non-Toxic	Non-Toxic
Distance	0.551	0.623	0.625
Reference	Oyo Yakuri 17(4):551-557; 1979	Fundam Appl Toxicol 4:91-97; 1984	J Toxicol Environ Health 14:585-594; 1984

Model Applicability

Unknown features are fingerprint features in the query molecule, but not found or appearing too infrequently in the training set.

1. All properties and OPS components are within expected ranges.

Feature Contribution

Top features for positive contribution

Fingerprint	Bit/Smiles	Feature Structure	Score	Toxic in training set
SCFP_6	2116304939	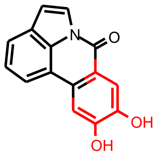 O[c]1:[cH]:[*]:[cH]:[cH]:[c]:1O	0.504	5 out of 5

SCFP_6	392579710	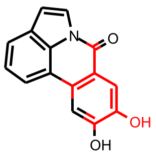 [*]=C[c]1:[cH]:[*]:[cH]:[c](O):[cH]:1	0.25	5 out of 7
SCFP_6	-424425761	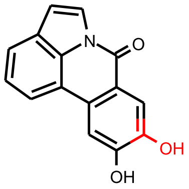 [*]:[c](:[*])O	0.196	20 out of 31

Top Features for negative contribution

Fingerprint	Bit/Smiles	Feature Structure	Score	Toxic in training set
SCFP_6	-485725958	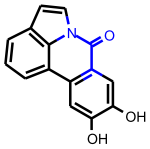 [*]:n(:[*])C(=O)[c](:[*]):[*]	-0.422	0 out of 1
SCFP_6	-1003223627	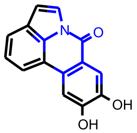 [*]:[cH]:[c]1C(=O)n2:[cH]:[*]:[*]:[c]:2:[*][c]:1:[*]	-0.422	0 out of 1
SCFP_6	-1798553344	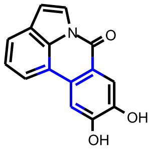 [*][c](:[*]):[c](:[cH]:[*])[c](:[*]):[*]	-0.358	3 out of 9

remdesivir

TOPKAT_Developmental_Toxicity_Potential

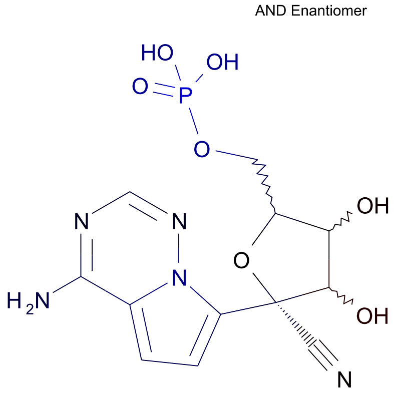

$C_{12}H_{14}N_5O_7P$
Molecular Weight: 371.24262
ALogP: -1.502
Rotatable Bonds: 4
Acceptors: 11
Donors: 5

Model Prediction

Prediction: Non-Toxic
Probability: 0.373
Enrichment: 0.709
Bayesian Score: -5.42
Mahalanobis Distance: 9.05
Mahalanobis Distance p-value: 0.163

Prediction: Positive if the Bayesian score is above the estimated best cutoff value from minimizing the false positive and false negative rate.
Probability: The esimated probability that the sample is in the positive category. This assumes that the Bayesian score follows a normal distribution and is different from the prediction using a cutoff.
Enrichment: An estimate of enrichment, that is, the increased likelihood (versus random) of this sample being in the category.
Bayesian Score: The standard Laplacian-modified Bayesian score.
Mahalanobis Distance: The Mahalanobis distance (MD) is the distance to the center of the training data. The larger the MD, the less trustworthy the prediction.
Mahalanobis Distance p-value: The p-value gives the fraction of training data with an MD greater than or equal to the one for the given sample, assuming normally distributed data. The smaller the p-value, the less trustworthy the prediciton. For highly non-normal X properties (e.g., fingerprints), the MD p-value is wildly inaccurate.

Structural Similar Compounds

Name	Sinigrin (Free Acid Form)	Azthreonam	Vidarabine
Structure	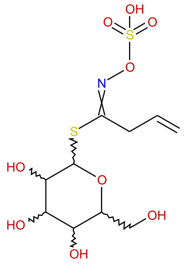	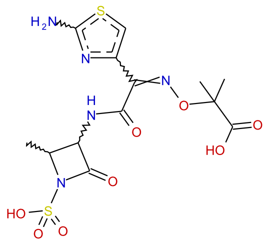	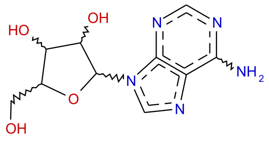
Actual Endpoint	Toxic	Non-Toxic	Non-Toxic
Predicted Endpoint	Toxic	Non-Toxic	Non-Toxic
Distance	0.632	0.707	0.714
Reference	Food Cosmet Toxicol 18(2):159-72; 1980	Chemotherapy 33:203-218; 1985	Teratology 15(3):231-41; 1977

Model Applicability

Unknown features are fingerprint features in the query molecule, but not found or appearing too infrequently in the training set.

1. All properties and OPS components are within expected ranges.

Feature Contribution

Top features for positive contribution

Fingerprint	Bit/Smiles	Feature Structure	Score	Toxic in training set
SCFP_6	-1486266146	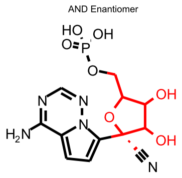 [*]CC1OC([*])([*])C(O)C1O	0.431	7 out of 8

SCFP_6	-1715619483	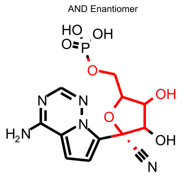 [*]OCC1OC([*])([*])C([*])C1O	0.298	6 out of 8
SCFP_6	-1181430618	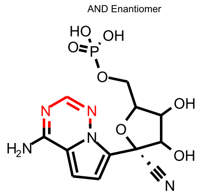 [*]:n:[cH]:n:[*]	0.298	6 out of 8

Top Features for negative contribution

Fingerprint	Bit/Smiles	Feature Structure	Score	Toxic in training set
SCFP_6	2108966103	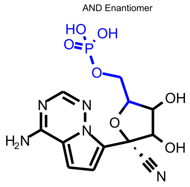 [*]C([*])COP(=O)(O)O	-0.945	0 out of 3
SCFP_6	-1375522316	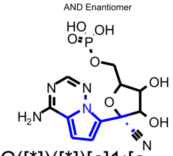 [*]C([*])([*])[c]1:[cH]:[cH]:[c](:[*]):n:1:[*]	-0.945	0 out of 3
SCFP_6	269938867	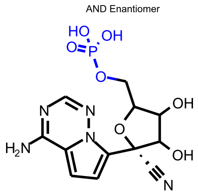 [*]OP(=O)(O)O	-0.729	1 out of 6

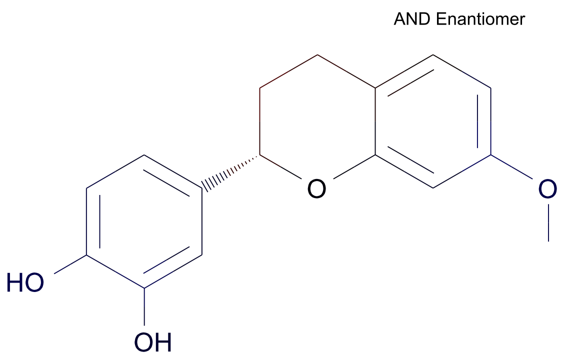

$C_{16}H_{16}O_4$
Molecular Weight: 272.296
ALogP: 3.456
Rotatable Bonds: 2
Acceptors: 4
Donors: 2

Model Prediction

Prediction: Non-Carcinogen
Probability: 0.223
Enrichment: 0.697
Bayesian Score: -2.63
Mahalanobis Distance: 10.9
Mahalanobis Distance p-value: 0.129

Prediction: Positive if the Bayesian score is above the estimated best cutoff value from minimizing the false positive and false negative rate.
Probability: The esimated probability that the sample is in the positive category. This assumes that the Bayesian score follows a normal distribution and is different from the prediction using a cutoff.
Enrichment: An estimate of enrichment, that is, the increased likelihood (versus random) of this sample being in the category.
Bayesian Score: The standard Laplacian-modified Bayesian score.
Mahalanobis Distance: The Mahalanobis distance (MD) is the distance to the center of the training data. The larger the MD, the less trustworthy the prediction.
Mahalanobis Distance p-value: The p-value gives the fraction of training data with an MD greater than or equal to the one for the given sample, assuming normally distributed data. The smaller the p-value, the less trustworthy the prediciton. For highly non-normal X properties (e.g., fingerprints), the MD p-value is wildly inaccurate.

Structural Similar Compounds

Name	Diflunisal	Phenolphthalein	Oxazepam
Structure	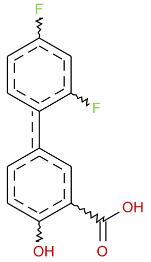	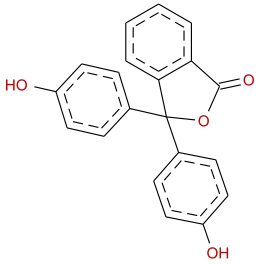	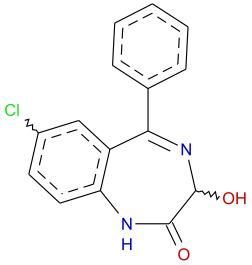
Actual Endpoint	Non-Carcinogen	Carcinogen	Carcinogen
Predicted Endpoint	Non-Carcinogen	Carcinogen	Carcinogen
Distance	0.556	0.561	0.592
Reference	US FDA (Centre for Drug Eval.& Res./Off. Testing & Res.) Sept. 1997	US FDA (Centre for Drug Eval.& Res./Off. Testing & Res.) Sept. 1997	US FDA (Centre for Drug Eval.& Res./Off. Testing & Res.) Sept. 1997

Model Applicability

Unknown features are fingerprint features in the query molecule, but not found or appearing too infrequently in the training set.

1. All properties and OPS components are within expected ranges.
2. Unknown ECFP_2 feature: -856154029: [*]C[C@H](O[*])[c](:[*]):[*]

Feature Contribution

Top features for positive contribution

Fingerprint	Bit/Smiles	Feature Structure	Score	Carcinogen in training set
ECFP_6	-1095664289	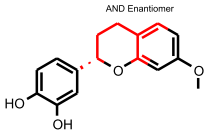 [*][C@H]1[*][c](:[*]):[c](CC1):[cH]:[*]	0.675	4 out of 5

ECFP_6	51876938	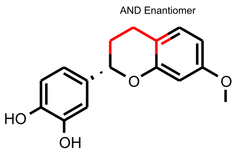 [*]CC[c](:[*]):[*]	0.473	16 out of 31
ECFP_6	1588719643	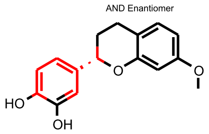 [*]C([*])[c]1:[cH]:[*]:[c]([*]):[cH]:[cH]:1	0.293	2 out of 4

Top Features for negative contribution

Fingerprint	Bit/Smiles	Feature Structure	Score	Carcinogen in training set
ECFP_6	2081544509	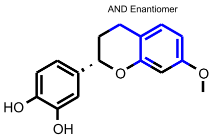 [*]C[c]1:[cH]:[cH]:[c]([*]):[*]:[c]:1[*]	-0.657	0 out of 3
ECFP_6	1307307440	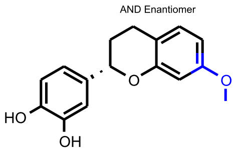 [*]:[c](:[*])OC	-0.558	4 out of 25
ECFP_6	1334400011	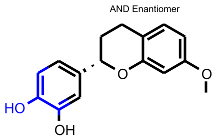 [*][c](:[*]):[c](O):[cH]:[*]	-0.496	3 out of 18

TOPKAT_Mouse_Female_FDA_None_vs_Carcinogen

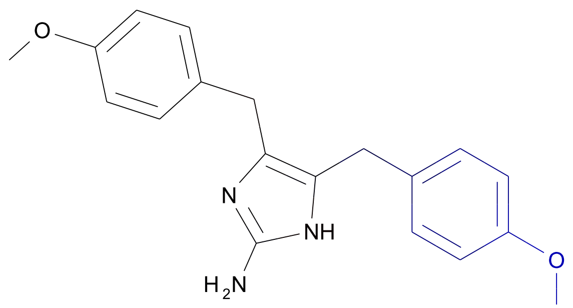

$C_{19}H_{21}N_3O_2$
Molecular Weight: 323.389
ALogP: 3.305
Rotatable Bonds: 6
Acceptors: 4
Donors: 2

Model Prediction

Prediction: Non-Carcinogen
Probability: 0.208
Enrichment: 0.648
Bayesian Score: -4.8
Mahalanobis Distance: 10.6
Mahalanobis Distance p-value: 0.214

Prediction: Positive if the Bayesian score is above the estimated best cutoff value from minimizing the false positive and false negative rate.
Probability: The esimated probability that the sample is in the positive category. This assumes that the Bayesian score follows a normal distribution and is different from the prediction using a cutoff.
Enrichment: An estimate of enrichment, that is, the increased likelihood (versus random) of this sample being in the category.
Bayesian Score: The standard Laplacian-modified Bayesian score.
Mahalanobis Distance: The Mahalanobis distance (MD) is the distance to the center of the training data. The larger the MD, the less trustworthy the prediction.
Mahalanobis Distance p-value: The p-value gives the fraction of training data with an MD greater than or equal to the one for the given sample, assuming normally distributed data. The smaller the p-value, the less trustworthy the prediciton. For highly non-normal X properties (e.g., fingerprints), the MD p-value is wildly inaccurate.

Structural Similar Compounds

Name	Bunolol	Metoclopramide	Omeprazole
Structure	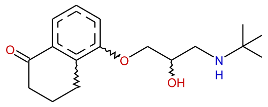	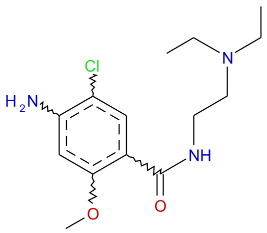	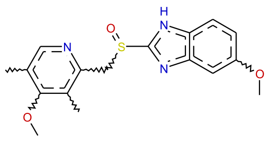
Actual Endpoint	Carcinogen	Non-Carcinogen	Non-Carcinogen
Predicted Endpoint	Carcinogen	Non-Carcinogen	Non-Carcinogen
Distance	0.581	0.608	0.609
Reference	US FDA (Centre for Drug Eval.& Res./Off. Testing & Res.) Sept. 1997	US FDA (Centre for Drug Eval.& Res./Off. Testing & Res.) Sept. 1997	US FDA (Centre for Drug Eval.& Res./Off. Testing & Res.) Sept. 1997

Model Applicability

Unknown features are fingerprint features in the query molecule, but not found or appearing too infrequently in the training set.

1. All properties and OPS components are within expected ranges.
2. Unknown ECFP_2 feature: -746759483: [*]C[c]1:[nH]:[*]:[*]:[c]:1[*]
3. Unknown ECFP_2 feature: -2046255371: N[c]1:[nH]:[*]:[*]:n:1

Feature Contribution

Top features for positive contribution

Fingerprint	Bit/Smiles	Feature Structure	Score	Carcinogen in training set
ECFP_6	-152683720	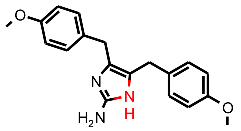 [*]:[nH]:[*]	0.245	7 out of 17

ECFP_6	-435942924	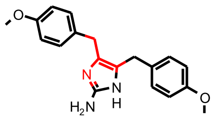 [*]C[c]1:n:[*]:[*]:[c]:1[*]	0.164	2 out of 5
ECFP_6	-938530932	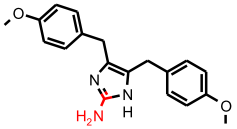 [*]:[c](:[*])N	0.0661	8 out of 24

Top Features for negative contribution

Fingerprint	Bit/Smiles	Feature Structure	Score	Carcinogen in training set
ECFP_6	-1271104377	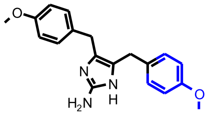 CO[c]1:[cH]:[cH]:[*]: [cH]:[cH]:1	-0.805	0 out of 4
ECFP_6	693720869	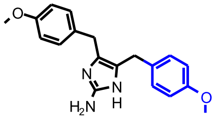 [*][c]1:[cH]:[cH]:[c] (OC):[cH]:[cH]:1	-0.805	0 out of 4
ECFP_6	1307307440	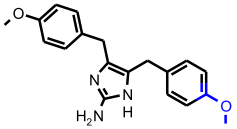 [*]:[c](:[*])OC	-0.558	4 out of 25

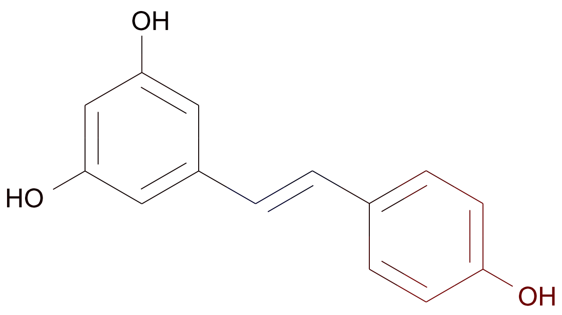

$C_{14}H_{12}O_3$
Molecular Weight: 228.243
ALogP: 3.09
Rotatable Bonds: 2
Acceptors: 3
Donors: 3

Model Prediction

Prediction: Carcinogen
Probability: 0.31
Enrichment: 0.967
Bayesian Score: 1.93
Mahalanobis Distance: 8.44
Mahalanobis Distance p-value: 0.963

Prediction: Positive if the Bayesian score is above the estimated best cutoff value from minimizing the false positive and false negative rate.
Probability: The esimated probability that the sample is in the positive category. This assumes that the Bayesian score follows a normal distribution and is different from the prediction using a cutoff.
Enrichment: An estimate of enrichment, that is, the increased likelihood (versus random) of this sample being in the category.
Bayesian Score: The standard Laplacian-modified Bayesian score.
Mahalanobis Distance: The Mahalanobis distance (MD) is the distance to the center of the training data. The larger the MD, the less trustworthy the prediction.
Mahalanobis Distance p-value: The p-value gives the fraction of training data with an MD greater than or equal to the one for the given sample, assuming normally distributed data. The smaller the p-value, the less trustworthy the prediciton. For highly non-normal X properties (e.g., fingerprints), the MD p-value is wildly inaccurate.

Structural Similar Compounds

Name	Diflunisal	Guanabenz	Guanfacine
Structure	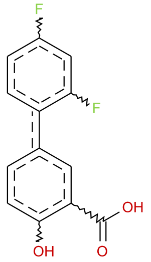	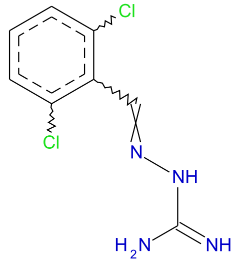	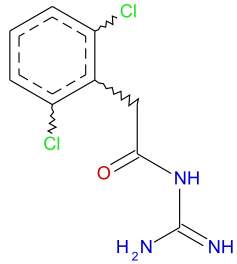
Actual Endpoint	Non-Carcinogen	Carcinogen	Non-Carcinogen
Predicted Endpoint	Non-Carcinogen	Carcinogen	Non-Carcinogen
Distance	0.602	0.625	0.645
Reference	US FDA (Centre for Drug Eval.& Res./Off. Testing & Res.) Sept. 1997	US FDA (Centre for Drug Eval.& Res./Off. Testing & Res.) Sept. 1997	US FDA (Centre for Drug Eval.& Res./Off. Testing & Res.) Sept. 1997

Model Applicability

Unknown features are fingerprint features in the query molecule, but not found or appearing too infrequently in the training set.

1. All properties and OPS components are within expected ranges.
2. Unknown ECFP_2 feature: -176483725: [*]=C[c](:c:[*]):c:[*]

Feature Contribution

Top features for positive contribution

Fingerprint	Bit/Smiles	Feature Structure	Score	Carcinogen in training set
ECFP_6	1419645508	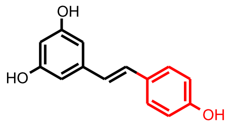 [*][c]1:[cH]:[cH]:[c](O):[cH]:[cH]:1	0.675	4 out of 5

ECFP_6	-790637051	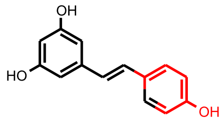 [*][c]1:[*]:[cH]:[c](O):[cH]:[cH]:1	0.615	6 out of 9
ECFP_6	1740779540	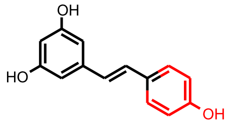 O[c]1:[cH]:[cH]:[*]:[cH]:[cH]:1	0.56	4 out of 6
**Top Features for negative contribution**				
**Fingerprint**	**Bit/Smiles**	**Feature Structure**	**Score**	**Carcinogen in training set**
ECFP_6	-1831055759	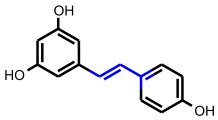 [*]\C=C\[c](:[*]):[*]	-0.805	0 out of 4
ECFP_6	-786013480	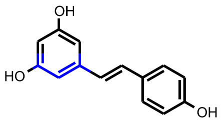 [*][c](:[*]):[cH]:[c] ([*]):[*]	-0.162	23 out of 88
ECFP_6	1996767644	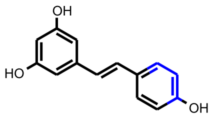 [*][c](:[*]):[cH]:[cH]:[*]	-0.109	59 out of 213

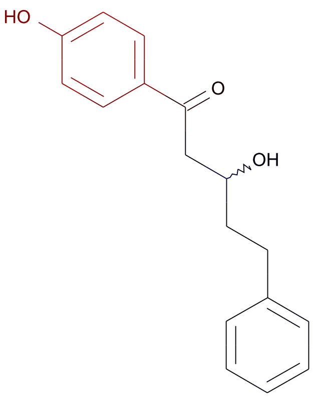

$C_{17}H_{18}O_3$
Molecular Weight: 270.323
ALogP: 3.293
Rotatable Bonds: 6
Acceptors: 3
Donors: 2

Structural Similar Compounds

Name	Propranolol	Penbutalol	Bunolol
Structure	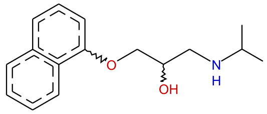	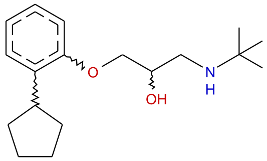	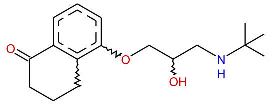
Actual Endpoint	Non-Carcinogen	Non-Carcinogen	Carcinogen
Predicted Endpoint	Non-Carcinogen	Non-Carcinogen	Carcinogen
Distance	0.546	0.574	0.577
Reference	US FDA (Centre for Drug Eval.& Res./Off. Testing & Res.) Sept. 1997	US FDA (Centre for Drug Eval.& Res./Off. Testing & Res.) Sept. 1997	US FDA (Centre for Drug Eval.& Res./Off. Testing & Res.) Sept. 1997

Model Prediction

Prediction: Carcinogen
Probability: 0.293
Enrichment: 0.915
Bayesian Score: 1.32
Mahalanobis Distance: 14.2
Mahalanobis Distance p-value: 5.88e-006

Prediction: Positive if the Bayesian score is above the estimated best cutoff value from minimizing the false positive and false negative rate.
Probability: The esimated probability that the sample is in the positive category. This assumes that the Bayesian score follows a normal distribution and is different from the prediction using a cutoff.
Enrichment: An estimate of enrichment, that is, the increased likelihood (versus random) of this sample being in the category.
Bayesian Score: The standard Laplacian-modified Bayesian score.
Mahalanobis Distance: The Mahalanobis distance (MD) is the distance to the center of the training data. The larger the MD, the less trustworthy the prediction.
Mahalanobis Distance p-value: The p-value gives the fraction of training data with an MD greater than or equal to the one for the given sample, assuming normally distributed data. The smaller the p-value, the less trustworthy the prediciton. For highly non-normal X properties (e.g., fingerprints), the MD p-value is wildly inaccurate.

Model Applicability

Unknown features are fingerprint features in the query molecule, but not found or appearing too infrequently in the training set.

1. All properties and OPS components are within expected ranges.

Feature Contribution

Top features for positive contribution				
**Fingerprint**	**Bit/Smiles**	**Feature Structure**	**Score**	**Carcinogen in training set**
ECFP_6	1419645508	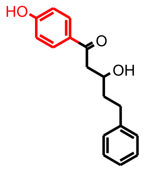 [*][c]1:[cH]:[cH]:[c](O):[cH]:[cH]:1	0.675	4 out of 5

ECFP_6	-790637051	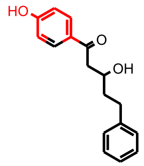 [*][c]1:[*]:[cH]:[c](O):[cH]:[cH]:1	0.615	6 out of 9
ECFP_6	1740779540	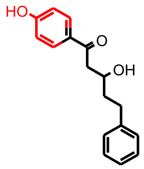 O[c]1:[cH]:[cH]:[*]:[cH]:[cH]:1	0.56	4 out of 6

Top Features for negative contribution

Fingerprint	Bit/Smiles	Feature Structure	Score	Carcinogen in training set
ECFP_6	-1310859884	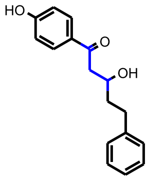 [*]C([*])CC(=[*])[*]	-0.482	0 out of 2
ECFP_6	-1910270391	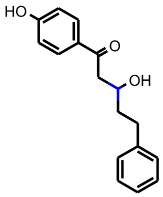 [*]C([*])[*]	-0.307	20 out of 89
ECFP_6	196083830	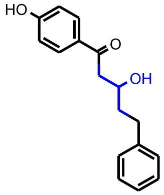 [*]CC(O)C[*]	-0.174	4 out of 16

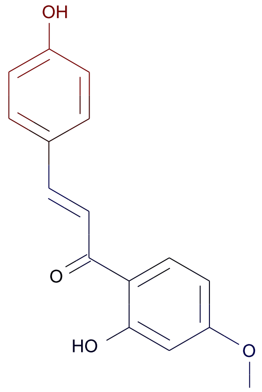

$C_{16}H_{14}O_4$
Molecular Weight: 270.28
ALogP: 3.201
Rotatable Bonds: 4
Acceptors: 4
Donors: 2

Model Prediction

Prediction: Non-Carcinogen
Probability: 0.235
Enrichment: 0.733
Bayesian Score: -1.67
Mahalanobis Distance: 10.4
Mahalanobis Distance p-value: 0.273

Prediction: Positive if the Bayesian score is above the estimated best cutoff value from minimizing the false positive and false negative rate.
Probability: The esimated probability that the sample is in the positive category. This assumes that the Bayesian score follows a normal distribution and is different from the prediction using a cutoff.
Enrichment: An estimate of enrichment, that is, the increased likelihood (versus random) of this sample being in the category.
Bayesian Score: The standard Laplacian-modified Bayesian score.
Mahalanobis Distance: The Mahalanobis distance (MD) is the distance to the center of the training data. The larger the MD, the less trustworthy the prediction.
Mahalanobis Distance p-value: The p-value gives the fraction of training data with an MD greater than or equal to the one for the given sample, assuming normally distributed data. The smaller the p-value, the less trustworthy the prediciton. For highly non-normal X properties (e.g., fingerprints), the MD p-value is wildly inaccurate.

Structural Similar Compounds

Name	Mebendazole	Cytembena	Etodolac
Structure	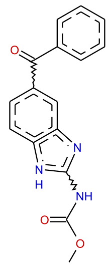	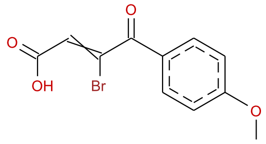	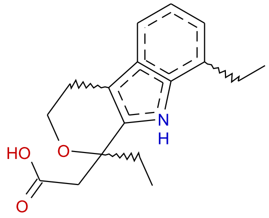
Actual Endpoint	Non-Carcinogen	Non-Carcinogen	Non-Carcinogen
Predicted Endpoint	Non-Carcinogen	Non-Carcinogen	Non-Carcinogen
Distance	0.523	0.562	0.570
Reference	US FDA (Centre for Drug Eval.& Res./Off. Testing & Res.) Sept. 1997	US FDA (Centre for Drug Eval.& Res./Off. Testing & Res.) Sept. 1997	US FDA (Centre for Drug Eval.& Res./Off. Testing & Res.) Sept. 1997

Model Applicability

Unknown features are fingerprint features in the query molecule, but not found or appearing too infrequently in the training set.

1. All properties and OPS components are within expected ranges.
2. Unknown ECFP_2 feature: 1430764055: [*]=CC(=O)[c]([*]):[*]
3. Unknown ECFP_2 feature: -176483725: [*]=C[c](:c:[*]):c:[*]

Feature Contribution

Top features for positive contribution

Fingerprint	Bit/Smiles	Feature Structure	Score	Carcinogen in training set
ECFP_6	1419645508	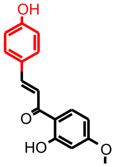 [*][c]1:[cH]:[cH]:[c](O):[cH]:[cH]:1	0.675	4 out of 5

ECFP_6	-790637051	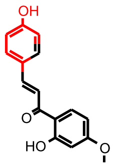 [*][c]1:[*]:[cH]:[c](O):[cH]:[cH]:1	0.615	6 out of 9
ECFP_6	1740779540	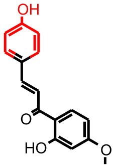 O[c]1:[cH]:[cH]:[*]:[cH]:[cH]:1	0.56	4 out of 6
**Top Features for negative contribution**				
**Fingerprint**	**Bit/Smiles**	**Feature Structure**	**Score**	**Carcinogen in training set**
ECFP_6	-1831055759	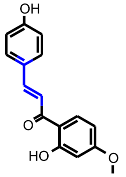 [*]\C=C\[c](:[*]):[*]	-0.805	0 out of 4
ECFP_6	-470416293	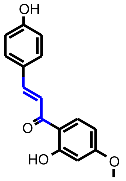 [*]\C=C\C(=[*])[*]	-0.657	0 out of 3
ECFP_6	1307307440	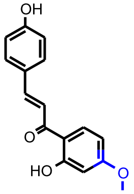 [*]:[c](:[*])OC	-0.558	4 out of 25

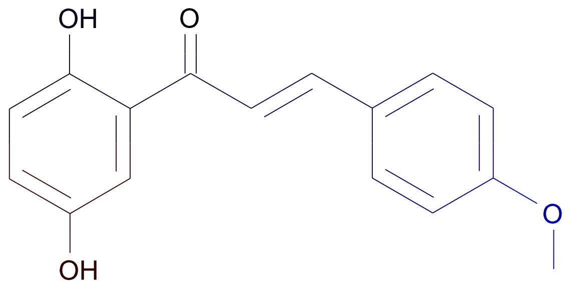

$C_{16}H_{14}O_4$
Molecular Weight: 270.28
ALogP: 3.201
Rotatable Bonds: 4
Acceptors: 4
Donors: 2

Model Prediction

Prediction: Non-Carcinogen
Probability: 0.209
Enrichment: 0.652
Bayesian Score: -4.52
Mahalanobis Distance: 10.4
Mahalanobis Distance p-value: 0.273

Prediction: Positive if the Bayesian score is above the estimated best cutoff value from minimizing the false positive and false negative rate.
Probability: The esimated probability that the sample is in the positive category. This assumes that the Bayesian score follows a normal distribution and is different from the prediction using a cutoff.
Enrichment: An estimate of enrichment, that is, the increased likelihood (versus random) of this sample being in the category.
Bayesian Score: The standard Laplacian-modified Bayesian score.
Mahalanobis Distance: The Mahalanobis distance (MD) is the distance to the center of the training data. The larger the MD, the less trustworthy the prediction.
Mahalanobis Distance p-value: The p-value gives the fraction of training data with an MD greater than or equal to the one for the given sample, assuming normally distributed data. The smaller the p-value, the less trustworthy the prediciton. For highly non-normal X properties (e.g., fingerprints), the MD p-value is wildly inaccurate.

Structural Similar Compounds

Name	Mebendazole	Cytembena	Diflunisal
Structure	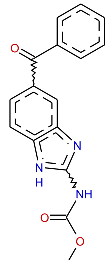	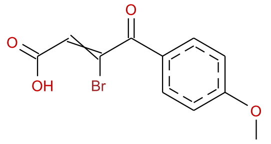	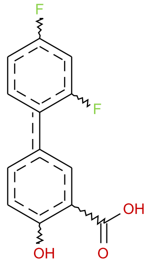
Actual Endpoint	Non-Carcinogen	Non-Carcinogen	Non-Carcinogen
Predicted Endpoint	Non-Carcinogen	Non-Carcinogen	Non-Carcinogen
Distance	0.523	0.546	0.570
Reference	US FDA (Centre for Drug Eval.& Res./Off. Testing & Res.) Sept. 1997	US FDA (Centre for Drug Eval.& Res./Off. Testing & Res.) Sept. 1997	US FDA (Centre for Drug Eval.& Res./Off. Testing & Res.) Sept. 1997

Model Applicability

Unknown features are fingerprint features in the query molecule, but not found or appearing too infrequently in the training set.

1. All properties and OPS components are within expected ranges.
2. Unknown ECFP_2 feature: -176483725: [*]=C[c](:c:[*]):c:[*]
3. Unknown ECFP_2 feature: 1430764055: [*]=CC(=O)[c](:[*]):[*]

Feature Contribution

Top features for positive contribution

Fingerprint	Bit/Smiles	Feature Structure	Score	Carcinogen in training set
ECFP_6	-790637051	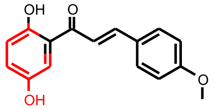 [*][c]1:[*]:[cH]:[c](O):[cH]:[cH]:1	0.615	6 out of 9

ECFP_6	-177786161	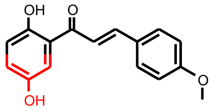 [*]:[cH]:[c](O):[cH]:[*]	0.406	7 out of 14
ECFP_6	-1925046727	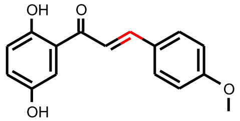 [*]C=[*]	0.391	11 out of 23

Top Features for negative contribution

Fingerprint	Bit/Smiles	Feature Structure	Score	Carcinogen in training set
ECFP_6	693720869	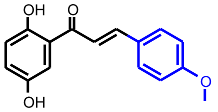 [*][c]1:[cH]:[cH]:[c](OC):[cH]:[cH]:1	-0.805	0 out of 4
ECFP_6	-1271104377	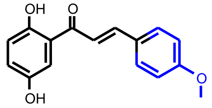 CO[c]1:[cH]:[cH]:[*]:[cH]:[cH]:1	-0.805	0 out of 4
ECFP_6	-1831055759	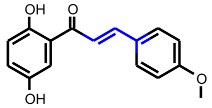 [*]\C=C\[c](:[*]):[*]	-0.805	0 out of 4

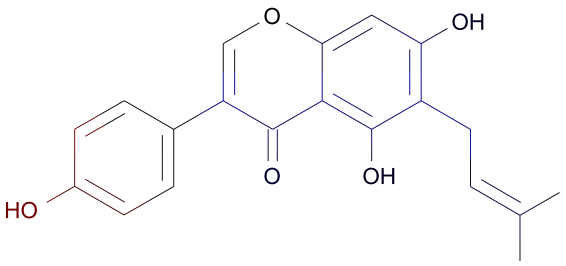

$C_{20}H_{18}O_5$
Molecular Weight: 338.354
ALogP: 3.997
Rotatable Bonds: 3
Acceptors: 5
Donors: 3

Model Prediction

Prediction: Non-Carcinogen
Probability: 0.207
Enrichment: 0.646
Bayesian Score: -5.01
Mahalanobis Distance: 13.6
Mahalanobis Distance p-value: 5.97e-005

Prediction: Positive if the Bayesian score is above the estimated best cutoff value from minimizing the false positive and false negative rate.
Probability: The esimated probability that the sample is in the positive category. This assumes that the Bayesian score follows a normal distribution and is different from the prediction using a cutoff.
Enrichment: An estimate of enrichment, that is, the increased likelihood (versus random) of this sample being in the category.
Bayesian Score: The standard Laplacian-modified Bayesian score.
Mahalanobis Distance: The Mahalanobis distance (MD) is the distance to the center of the training data. The larger the MD, the less trustworthy the prediction.
Mahalanobis Distance p-value: The p-value gives the fraction of training data with an MD greater than or equal to the one for the given sample, assuming normally distributed data. The smaller the p-value, the less trustworthy the prediciton. For highly non-normal X properties (e.g., fingerprints), the MD p-value is wildly inaccurate.

Structural Similar Compounds

Name	Phenolphthalein	Torsemide	Niclosamide
Structure	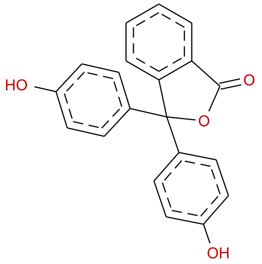	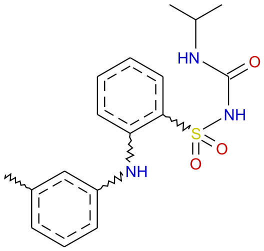	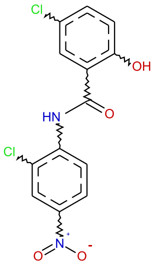
Actual Endpoint	Carcinogen	Non-Carcinogen	Non-Carcinogen
Predicted Endpoint	Carcinogen	Non-Carcinogen	Non-Carcinogen
Distance	0.616	0.632	0.645
Reference	US FDA (Centre for Drug Eval.& Res./Off. Testing & Res.) Sept. 1997	US FDA (Centre for Drug Eval.& Res./Off. Testing & Res.) Sept. 1997	US FDA (Centre for Drug Eval.& Res./Off. Testing & Res.) Sept. 1997

Model Applicability

Unknown features are fingerprint features in the query molecule, but not found or appearing too infrequently in the training set.

1. All properties and OPS components are within expected ranges.
2. Unknown ECFP_2 feature: -1774681326: [*]C=C(C)C
3. Unknown ECFP_2 feature: 1717082529: [*]\C=C(/C(=[*])[*])\[c](:[*]):[*]
4. Unknown ECFP_2 feature: 471124258: [*]OC=C([*])[*]
5. Unknown ECFP_2 feature: -554736825: [*]=CO[c](:[*]):[*]

Feature Contribution

Top features for positive contribution

Fingerprint	Bit/Smiles	Feature Structure	Score	Carcinogen in training set
ECFP_6	1419645508	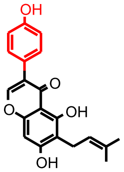 [*][c]1:[cH]:[cH]:[c](O):[cH]:[cH]:1	0.675	4 out of 5

ECFP_6	-790637051	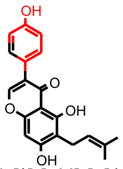 [*][c]1:[*]:[cH]:[c](O):[cH]:[cH]:1	0.615	6 out of 9
ECFP_6	1740779540	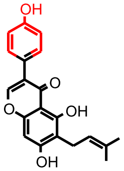 O[c]1:[cH]:[cH]:[*]:[cH]:[cH]:1	0.56	4 out of 6
**Top Features for negative contribution**				
**Fingerprint**	**Bit/Smiles**	**Feature Structure**	**Score**	**Carcinogen in training set**
ECFP_6	1717462980	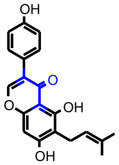 [*]C(=[*])C(=O)[c](:[*]):[*]	-1.25	0 out of 8
ECFP_6	-512323383	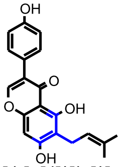 [*]C[c](:[c]([*]):[*]):[c]([*]):[*]	-0.935	0 out of 5
ECFP_6	-219423964	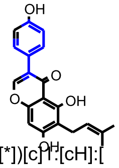 [*]C(=[*])[c]1:[cH]:[*]:[c]([*]):[cH]:[cH]:1	-0.935	0 out of 5

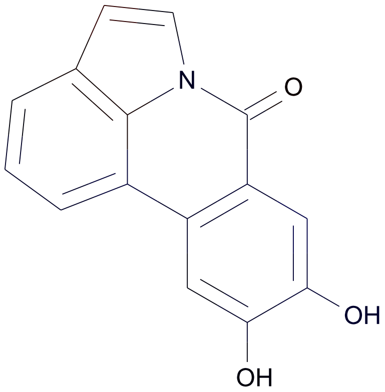

$C_{15}H_9NO_3$
Molecular Weight: 251.237
ALogP: 2.982
Rotatable Bonds: 0
Acceptors: 3
Donors: 2

Structural Similar Compounds

Name	Oxazepam	Diflunisal	Proflavine
Structure	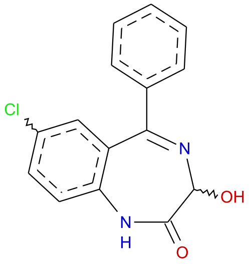	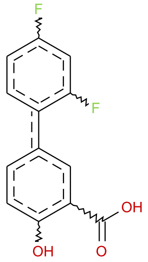	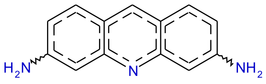
Actual Endpoint	Carcinogen	Non-Carcinogen	Carcinogen
Predicted Endpoint	Carcinogen	Non-Carcinogen	Carcinogen
Distance	0.555	0.574	0.595
Reference	US FDA (Centre for Drug Eval.& Res./Off. Testing & Res.) Sept. 1997	US FDA (Centre for Drug Eval.& Res./Off. Testing & Res.) Sept. 1997	US FDA (Centre for Drug Eval.& Res./Off. Testing & Res.) Sept. 1997

Model Prediction

Prediction: Non-Carcinogen
Probability: 0.23
Enrichment: 0.717
Bayesian Score: -2.06
Mahalanobis Distance: 10.7
Mahalanobis Distance p-value: 0.165

Prediction: Positive if the Bayesian score is above the estimated best cutoff value from minimizing the false positive and false negative rate.
Probability: The esimated probability that the sample is in the positive category. This assumes that the Bayesian score follows a normal distribution and is different from the prediction using a cutoff.
Enrichment: An estimate of enrichment, that is, the increased likelihood (versus random) of this sample being in the category.
Bayesian Score: The standard Laplacian-modified Bayesian score.
Mahalanobis Distance: The Mahalanobis distance (MD) is the distance to the center of the training data. The larger the MD, the less trustworthy the prediction.
Mahalanobis Distance p-value: The p-value gives the fraction of training data with an MD greater than or equal to the one for the given sample, assuming normally distributed data. The smaller the p-value, the less trustworthy the prediciton. For highly non-normal X properties (e.g., fingerprints), the MD p-value is wildly inaccurate.

Model Applicability

Unknown features are fingerprint features in the query molecule, but not found or appearing too infrequently in the training set.

1. All properties and OPS components are within expected ranges.
2. Unknown ECFP_2 feature: -1660898726: [*]n1:[*]:[*]:[c](:[*]):[c]:1:[c]([*]):[*]
3. Unknown ECFP_2 feature: 1444648700: [*]:n(:[*])C(=O)[c](:[*]):[*]

Feature Contribution

Top features for positive contribution

Fingerprint	Bit/Smiles	Feature Structure	Score	Carcinogen in training set
ECFP_6	-178525456	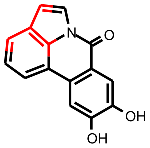 [*]:[cH]:[c]1:[cH]:[*]:[*]:[c]:1:[*]	0.457	4 out of 7

ECFP_6	717474525	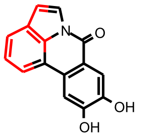 [*]1:[*]:[c]2:[*]:[cH]:[cH]:[cH]:[c]:2:[cH]:1	0.451	3 out of 5
ECFP_6	2106656448	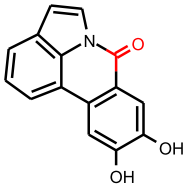 [*]C(=O)[*]	0.254	31 out of 77

Top Features for negative contribution

Fingerprint	Bit/Smiles	Feature Structure	Score	Carcinogen in training set
ECFP_6	2007300961	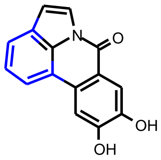 [*][c]1:[*]:[c](:[*]):[cH]:[cH]:[cH]:1	-0.652	5 out of 34
ECFP_6	1334400011	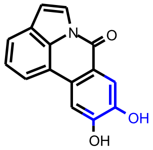 [*][c](:[*]):[c](O):[cH]:[*]	-0.496	3 out of 18
ECFP_6	-427397688	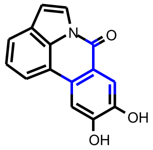 [*]C(=[*])[c](:[cH]:[*]):[c]([*]):[*]	-0.476	5 out of 28

remdesivir

TOPKAT_Mouse_Female_FDA_None_vs_Carcinogen

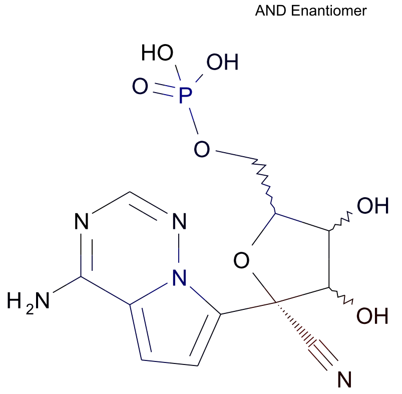

$C_{12}H_{14}N_5O_7P$
Molecular Weight: 371.24262
ALogP: -1.502
Rotatable Bonds: 4
Acceptors: 11
Donors: 5

Structural Similar Compounds

Name	Famotidine	Tetracycline	Oxytetracycline
Structure	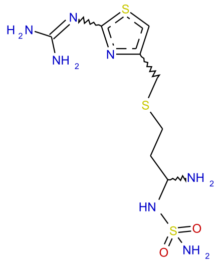	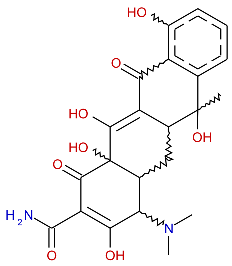	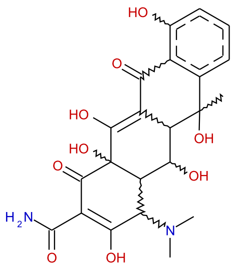
Actual Endpoint	Non-Carcinogen	Non-Carcinogen	Non-Carcinogen
Predicted Endpoint	Non-Carcinogen	Non-Carcinogen	Non-Carcinogen
Distance	0.846	0.848	0.870
Reference	US FDA (Centre for Drug Eval.& Res./Off. Testing & Res.) Sept. 1997	US FDA (Centre for Drug Eval.& Res./Off. Testing & Res.) Sept. 1997	US FDA (Centre for Drug Eval.& Res./Off. Testing & Res.) Sept. 1997

Model Prediction

Prediction: Non-Carcinogen
Probability: 0.206
Enrichment: 0.642
Bayesian Score: -7.17
Mahalanobis Distance: 12.9
Mahalanobis Distance p-value: 0.00074

Prediction: Positive if the Bayesian score is above the estimated best cutoff value from minimizing the false positive and false negative rate.
Probability: The esimated probability that the sample is in the positive category. This assumes that the Bayesian score follows a normal distribution and is different from the prediction using a cutoff.
Enrichment: An estimate of enrichment, that is, the increased likelihood (versus random) of this sample being in the category.
Bayesian Score: The standard Laplacian-modified Bayesian score.
Mahalanobis Distance: The Mahalanobis distance (MD) is the distance to the center of the training data. The larger the MD, the less trustworthy the prediction.
Mahalanobis Distance p-value: The p-value gives the fraction of training data with an MD greater than or equal to the one for the given sample, assuming normally distributed data. The smaller the p-value, the less trustworthy the prediciton. For highly non-normal X properties (e.g., fingerprints), the MD p-value is wildly inaccurate.

Model Applicability

Unknown features are fingerprint features in the query molecule, but not found or appearing too infrequently in the training set.

1. All properties and OPS components are within expected ranges.
2. Unknown ECFP_2 feature: 1126642748: [*]OP(=O)(O)O
3. Unknown ECFP_2 feature: -1250439909: [*]COP(=[*])([*])[*]
4. Unknown ECFP_2 feature: 1258791451: [*]C1[*][*]O[C@]1(C#[*])[c](:[*]):[*]
5. Unknown ECFP_2 feature: -1507082173: [*][c]1:[*]:[*]:[c](:[*]):n:1:n:[*]
6. Unknown ECFP_2 feature: -66263742: [*]C([*])([*])[c]1:[cH]:[*]:[*]:n:1:[*]

Feature Contribution

Top features for positive contribution

Fingerprint	Bit/Smiles	Feature Structure	Score	Carcinogen in training set

ECFP_6	-1114776580	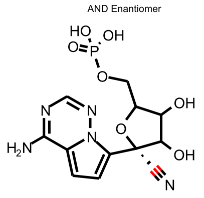 [*]C#[*]	0.755	11 out of 15
ECFP_6	-521596699	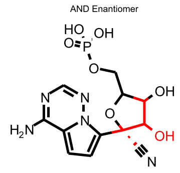 [*]C1[*][*]C([*])([*])C1O	0.451	3 out of 5
ECFP_6	-264833661	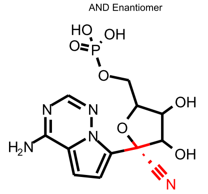 [*]C([*])([*])C#N	0.424	1 out of 1
**Top Features for negative contribution**				
**Fingerprint**	**Bit/Smiles**	**Feature Structure**	**Score**	**Carcinogen in training set**
ECFP_6	-826638028	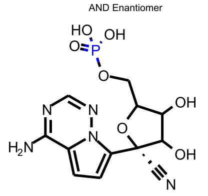 [*]P(=[*])([*])[*]	-0.935	0 out of 5
ECFP_6	2100964382	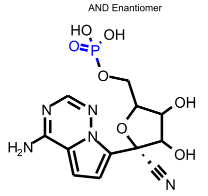 [*]P(=O)([*])[*]	-0.935	0 out of 5

ECFP_6	1334415134	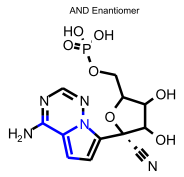 [*][c](:[*]):[c]1:[cH]:[*]:[*]:n:1:[*]	-0.935	0 out of 5

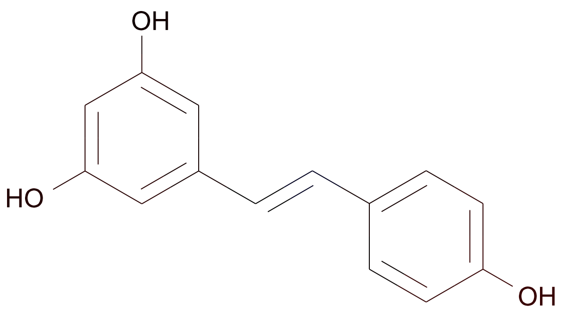

$C_{14}H_{12}O_3$
Molecular Weight: 228.243
ALogP: 3.09
Rotatable Bonds: 2
Acceptors: 3
Donors: 3

Structural Similar Compounds

Name	Guanabenz	Hydroquinone	Oxazepam
Structure	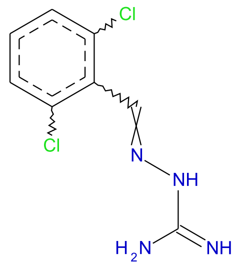	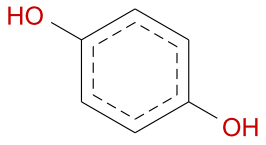	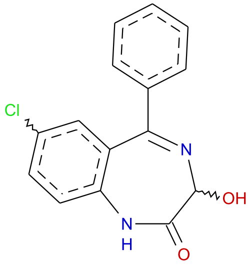
Actual Endpoint	Single-Carcinogen	Single-Carcinogen	Multiple-Carcinogen
Predicted Endpoint	Single-Carcinogen	Multiple-Carcinogen	Multiple-Carcinogen
Distance	0.618	0.651	0.667
Reference	US FDA (Centre for Drug Eval.& Res./Off. Testing & Res.) Sept. 1997	US FDA (Centre for Drug Eval.& Res./Off. Testing & Res.) Sept. 1997	US FDA (Centre for Drug Eval.& Res./Off. Testing & Res.) Sept. 1997

Model Prediction

Prediction: Multiple-Carcinogen
Probability: 0.447
Enrichment: 1.09
Bayesian Score: 1.07
Mahalanobis Distance: 8.87
Mahalanobis Distance p-value: 0.0952

Prediction: Positive if the Bayesian score is above the estimated best cutoff value from minimizing the false positive and false negative rate.
Probability: The esimated probability that the sample is in the positive category. This assumes that the Bayesian score follows a normal distribution and is different from the prediction using a cutoff.
Enrichment: An estimate of enrichment, that is, the increased likelihood (versus random) of this sample being in the category.
Bayesian Score: The standard Laplacian-modified Bayesian score.
Mahalanobis Distance: The Mahalanobis distance (MD) is the distance to the center of the training data. The larger the MD, the less trustworthy the prediction.
Mahalanobis Distance p-value: The p-value gives the fraction of training data with an MD greater than or equal to the one for the given sample, assuming normally distributed data. The smaller the p-value, the less trustworthy the prediciton. For highly non-normal X properties (e.g., fingerprints), the MD p-value is wildly inaccurate.

Model Applicability

Unknown features are fingerprint features in the query molecule, but not found or appearing too infrequently in the training set.

1. All properties and OPS components are within expected ranges.
2. Unknown ECFP_2 feature: -176483725: [*]=C[c](:c:[*]):c:[*]
3. Unknown ECFP_2 feature: -1831055759: [*]\C=C\[c](:[*]):[*]

Feature Contribution

Top features for positive contribution

Fingerprint	Bit/Smiles	Feature Structure	Score	Multiple-Carcinogen in training set
ECFP_4	-177786161	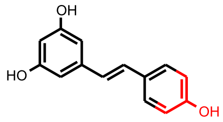 [*]:[cH]:[c](O):[cH]:[*]	0.441	5 out of 7

ECFP_4	-790637051	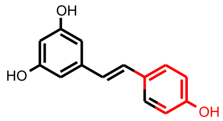 [*][c]1:[*]:[cH]:[c](O):[cH]:[cH]:1	0.371	4 out of 6
ECFP_4	1643326879	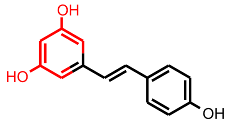 O[c]1:[cH]:[*]:[cH]:[c](O):[cH]:1	0.351	1 out of 1
**Top Features for negative contribution**				
**Fingerprint**	**Bit/Smiles**	**Feature Structure**	**Score**	**Multiple-Carcinogen in training set**
ECFP_4	-1925046727	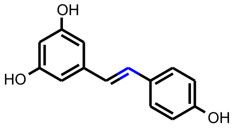 [*]C=[*]	-0.605	2 out of 11
ECFP_4	-182236392	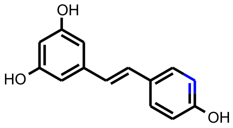 [*]:[cH]:[*]	-0.0651	34 out of 89
ECFP_4	1996767644	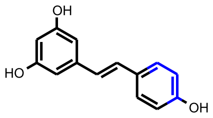 [*][c](:[*]):[cH]:[cH]:[*]	0	25 out of 59

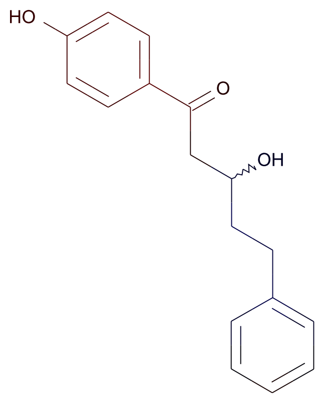

$C_{17}H_{18}O_3$
Molecular Weight: 270.323
ALogP: 3.293
Rotatable Bonds: 6
Acceptors: 3
Donors: 2

Model Prediction

Prediction: Multiple-Carcinogen
Probability: 0.486
Enrichment: 1.18
Bayesian Score: 1.89
Mahalanobis Distance: 9.72
Mahalanobis Distance p-value: 0.0263

Prediction: Positive if the Bayesian score is above the estimated best cutoff value from minimizing the false positive and false negative rate.
Probability: The esimated probability that the sample is in the positive category. This assumes that the Bayesian score follows a normal distribution and is different from the prediction using a cutoff.
Enrichment: An estimate of enrichment, that is, the increased likelihood (versus random) of this sample being in the category.
Bayesian Score: The standard Laplacian-modified Bayesian score.
Mahalanobis Distance: The Mahalanobis distance (MD) is the distance to the center of the training data. The larger the MD, the less trustworthy the prediction.
Mahalanobis Distance p-value: The p-value gives the fraction of training data with an MD greater than or equal to the one for the given sample, assuming normally distributed data. The smaller the p-value, the less trustworthy the prediciton. For highly non-normal X properties (e.g., fingerprints), the MD p-value is wildly inaccurate.

Structural Similar Compounds

Name	Bunolol	Prilocaine	Diethylstilbesterol
Structure	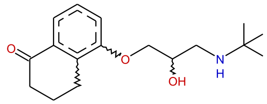	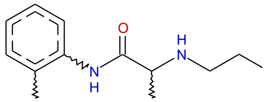	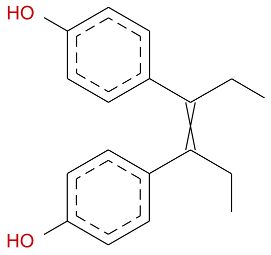
Actual Endpoint	Single-Carcinogen	Multiple-Carcinogen	Multiple-Carcinogen
Predicted Endpoint	Single-Carcinogen	Multiple-Carcinogen	Multiple-Carcinogen
Distance	0.561	0.589	0.592
Reference	US FDA (Centre for Drug Eval.& Res./Off. Testing & Res.) Sept. 1997	US FDA (Centre for Drug Eval.& Res./Off. Testing & Res.) Sept. 1997	US FDA (Centre for Drug Eval.& Res./Off. Testing & Res.) Sept. 1997

Model Applicability

Unknown features are fingerprint features in the query molecule, but not found or appearing too infrequently in the training set.

1. All properties and OPS components are within expected ranges.
2. Unknown ECFP_2 feature: -1310859884: [*]C([*])CC(=[*])[*]

Feature Contribution

Top features for positive contribution

Fingerprint	Bit/Smiles	Feature Structure	Score	Multiple-Carcinogen in training set
ECFP_4	-177786161	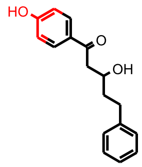 [*]:[cH]:[c](O):[cH]:[*]	0.441	5 out of 7

ECFP_4	-175146122	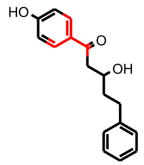 [*]C(=[*])[c](:[cH]:[*]):[cH]:[*]	0.403	6 out of 9
ECFP_4	-790637051	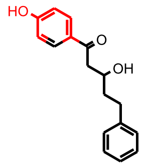 [*][c]1:[*]:[cH]:[c](O):[cH]:[cH]:1	0.371	4 out of 6
**Top Features for negative contribution**				
**Fingerprint**	**Bit/Smiles**	**Feature Structure**	**Score**	**Multiple-Carcinogen in training set**
ECFP_4	1878498340	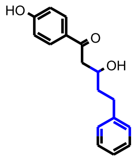 [*]C([*])CC[c](:[cH]: [*]):[cH]:[*]	-0.597	0 out of 2
ECFP_4	1205550831	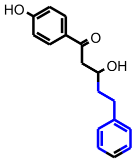 [*]CC[c]1:[cH]:[cH]:[*]:[cH]:[cH]:1	-0.545	1 out of 6
ECFP_4	2023785560	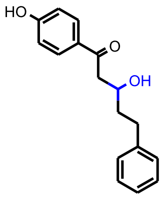 [*]C([*])O	-0.444	2 out of 9

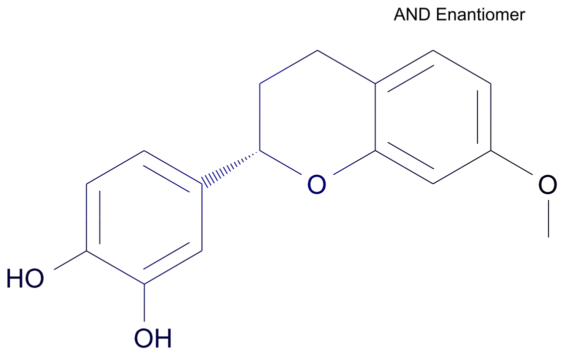

$C_{16}H_{16}O_4$
Molecular Weight: 272.296
ALogP: 3.456
Rotatable Bonds: 2
Acceptors: 4
Donors: 2

Model Prediction

Prediction: Non-Carcinogen
Probability: 0.197
Enrichment: 0.669
Bayesian Score: -5.19
Mahalanobis Distance: 13
Mahalanobis Distance p-value: 0.000245

Prediction: Positive if the Bayesian score is above the estimated best cutoff value from minimizing the false positive and false negative rate.
Probability: The esimated probability that the sample is in the positive category. This assumes that the Bayesian score follows a normal distribution and is different from the prediction using a cutoff.
Enrichment: An estimate of enrichment, that is, the increased likelihood (versus random) of this sample being in the category.
Bayesian Score: The standard Laplacian-modified Bayesian score.
Mahalanobis Distance: The Mahalanobis distance (MD) is the distance to the center of the training data. The larger the MD, the less trustworthy the prediction.
Mahalanobis Distance p-value: The p-value gives the fraction of training data with an MD greater than or equal to the one for the given sample, assuming normally distributed data. The smaller the p-value, the less trustworthy the prediciton. For highly non-normal X properties (e.g., fingerprints), the MD p-value is wildly inaccurate.

Structural Similar Compounds

Name	Phenolphthalein	Diflunisal	Oxazepam
Structure	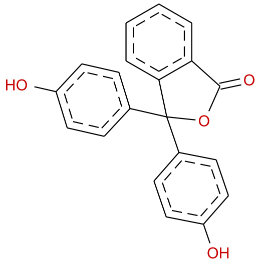	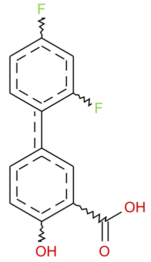	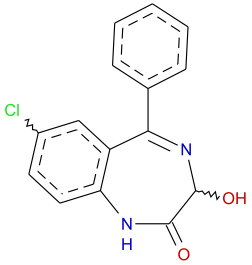
Actual Endpoint	Carcinogen	Non-Carcinogen	Carcinogen
Predicted Endpoint	Carcinogen	Non-Carcinogen	Carcinogen
Distance	0.521	0.540	0.586
Reference	US FDA (Centre for Drug Eval.& Res./Off. Testing & Res.) Sept. 1997	US FDA (Centre for Drug Eval.& Res./Off. Testing & Res.) Sept. 1997	US FDA (Centre for Drug Eval.& Res./Off. Testing & Res.) Sept. 1997

Model Applicability

Unknown features are fingerprint features in the query molecule, but not found or appearing too infrequently in the training set.

1. All properties and OPS components are within expected ranges.

Feature Contribution

Top features for positive contribution

Fingerprint	Bit/Smiles	Feature Structure	Score	Carcinogen in training set
FCFP_6	1679744180	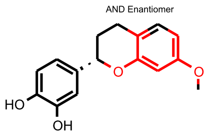 [*]O[c]1:[cH]:[*]:[c]([*]):[c](O[*]):[cH]:1	0.271	1 out of 2

FCFP_6	0	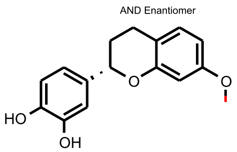<br>[*]C	0.114	90 out of 305
FCFP_6	346218766	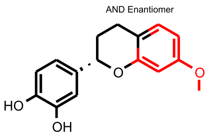<br>[*][c]1:[*]:[cH]:[cH]:[c](OC):[cH]:1	0.105	8 out of 27

Top Features for negative contribution

Fingerprint	Bit/Smiles	Feature Structure	Score	Carcinogen in training set
FCFP_6	1186333723	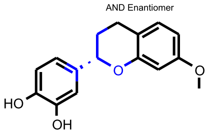<br>[*]C[C@H](O[*])[c](:[*]):[*]	-0.719	0 out of 4
FCFP_6	1916525245	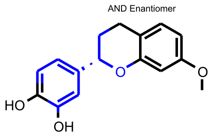<br>[*]C[C@H](O[*])[c]1:[cH]:[cH]:[*]:[c]([*]):[cH]:1	-0.582	0 out of 3
FCFP_6	184987616	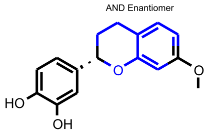<br>[*]1CC[c]2:[cH]:[cH]:[*]:[cH]:[c]:2O1	-0.582	0 out of 3

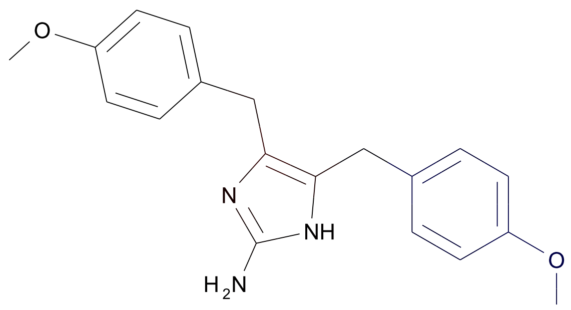

$C_{19}H_{21}N_3O_2$
Molecular Weight: 323.389
ALogP: 3.305
Rotatable Bonds: 6
Acceptors: 4
Donors: 2

Model Prediction

Prediction: Non-Carcinogen
Probability: 0.231
Enrichment: 0.786
Bayesian Score: -3.2
Mahalanobis Distance: 11.6
Mahalanobis Distance p-value: 0.0191

Prediction: Positive if the Bayesian score is above the estimated best cutoff value from minimizing the false positive and false negative rate.
Probability: The esimated probability that the sample is in the positive category. This assumes that the Bayesian score follows a normal distribution and is different from the prediction using a cutoff.
Enrichment: An estimate of enrichment, that is, the increased likelihood (versus random) of this sample being in the category.
Bayesian Score: The standard Laplacian-modified Bayesian score.
Mahalanobis Distance: The Mahalanobis distance (MD) is the distance to the center of the training data. The larger the MD, the less trustworthy the prediction.
Mahalanobis Distance p-value: The p-value gives the fraction of training data with an MD greater than or equal to the one for the given sample, assuming normally distributed data. The smaller the p-value, the less trustworthy the prediciton. For highly non-normal X properties (e.g., fingerprints), the MD p-value is wildly inaccurate.

Structural Similar Compounds

Name	Bunolol	Mebendazole	Metoclopramide
Structure	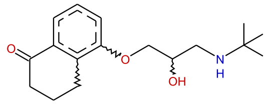	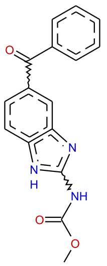	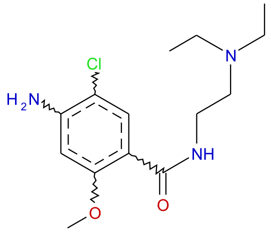
Actual Endpoint	Non-Carcinogen	Non-Carcinogen	Non-Carcinogen
Predicted Endpoint	Non-Carcinogen	Non-Carcinogen	Non-Carcinogen
Distance	0.551	0.582	0.589
Reference	US FDA (Centre for Drug Eval.& Res./Off. Testing & Res.) Sept. 1997	US FDA (Centre for Drug Eval.& Res./Off. Testing & Res.) Sept. 1997	US FDA (Centre for Drug Eval.& Res./Off. Testing & Res.) Sept. 1997

Model Applicability

Unknown features are fingerprint features in the query molecule, but not found or appearing too infrequently in the training set.

1. All properties and OPS components are within expected ranges.

Feature Contribution

Top features for positive contribution

Fingerprint	Bit/Smiles	Feature Structure	Score	Carcinogen in training set
FCFP_6	203707511	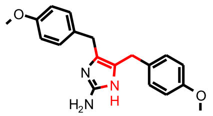 [*]C[c]1:[nH]:[*]:[*]:[c]:1[*]	0.38	2 out of 4

FCFP_6	-1539132615	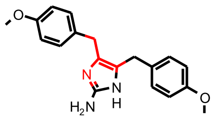 [*]C[c]1:n:[*]:[*]:[c]:1[*]	0.328	19 out of 51
FCFP_6	1747237384	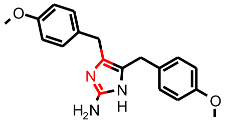 [*][c]1:[*]:[*]:[c]([*]):n:1	0.201	24 out of 74
**Top Features for negative contribution**				
**Fingerprint**	**Bit/Smiles**	**Feature Structure**	**Score**	**Carcinogen in training set**
FCFP_6	-9847677	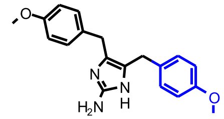 [*][c]1:[cH]:[cH]:[c] (OC):[cH]:[cH]:1	-0.719	0 out of 4
FCFP_6	136627117	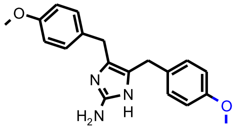 [*]OC	-0.252	10 out of 50
FCFP_6	906530397	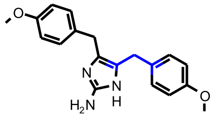 [*]:[c](:[*])C[c](:[*]):[*]	-0.233	0 out of 1

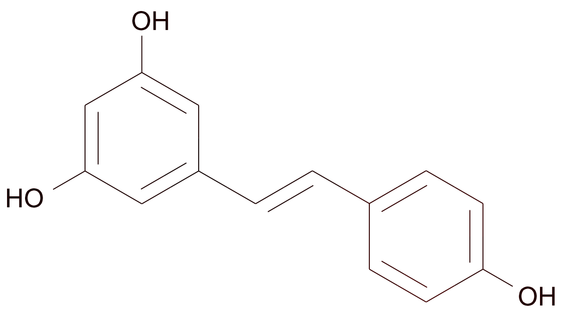

$C_{14}H_{12}O_3$
Molecular Weight: 228.243
ALogP: 3.09
Rotatable Bonds: 2
Acceptors: 3
Donors: 3

Model Prediction

Prediction: Carcinogen
Probability: 0.332
Enrichment: 1.13
Bayesian Score: 0.914
Mahalanobis Distance: 6.73
Mahalanobis Distance p-value: 1

Prediction: Positive if the Bayesian score is above the estimated best cutoff value from minimizing the false positive and false negative rate.
Probability: The esimated probability that the sample is in the positive category. This assumes that the Bayesian score follows a normal distribution and is different from the prediction using a cutoff.
Enrichment: An estimate of enrichment, that is, the increased likelihood (versus random) of this sample being in the category.
Bayesian Score: The standard Laplacian-modified Bayesian score.
Mahalanobis Distance: The Mahalanobis distance (MD) is the distance to the center of the training data. The larger the MD, the less trustworthy the prediction.
Mahalanobis Distance p-value: The p-value gives the fraction of training data with an MD greater than or equal to the one for the given sample, assuming normally distributed data. The smaller the p-value, the less trustworthy the prediciton. For highly non-normal X properties (e.g., fingerprints), the MD p-value is wildly inaccurate.

Structural Similar Compounds

Name	Diflunisal	Phenolphthalein	Terbutaline
Structure	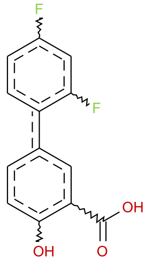	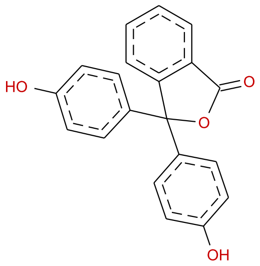	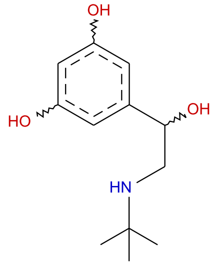
Actual Endpoint	Non-Carcinogen	Carcinogen	Non-Carcinogen
Predicted Endpoint	Non-Carcinogen	Carcinogen	Non-Carcinogen
Distance	0.574	0.615	0.617
Reference	US FDA (Centre for Drug Eval.& Res./Off. Testing & Res.) Sept. 1997	US FDA (Centre for Drug Eval.& Res./Off. Testing & Res.) Sept. 1997	US FDA (Centre for Drug Eval.& Res./Off. Testing & Res.) Sept. 1997

Model Applicability

Unknown features are fingerprint features in the query molecule, but not found or appearing too infrequently in the training set.

1. All properties and OPS components are within expected ranges.

Feature Contribution

Top features for positive contribution				
**Fingerprint**	**Bit/Smiles**	**Feature Structure**	**Score**	**Carcinogen in training set**
FCFP_6	-1066794953	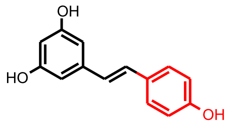 [*][c]1:[cH]:[cH]:[c](O):[cH]:[cH]:1	0.668	3 out of 4

FCFP_6	-1847351220	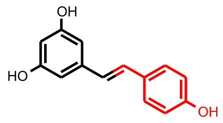 [*]=C[c]1:[cH]:[cH]:[c](O):[cH]:[cH]:1	0.547	3 out of 5
FCFP_6	451371068	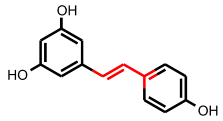 [*]\C=C\[c](:[*]):[*]	0.439	3 out of 6
**Top Features for negative contribution**				
**Fingerprint**	**Bit/Smiles**	**Feature Structure**	**Score**	**Carcinogen in training set**
FCFP_6	7	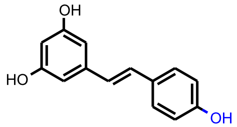 [*]O	-0.308	15 out of 79
FCFP_6	-549108873	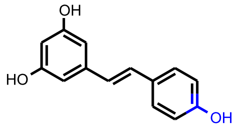 [*]:[c](:[*])O	-0.243	7 out of 35
FCFP_6	74595001	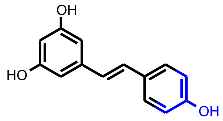 [*][c](:[*]):[c](O):[cH]:[*]	-0.243	7 out of 35

2982

TOPKAT_Mouse_Male_FDA_None_vs_Carcinogen

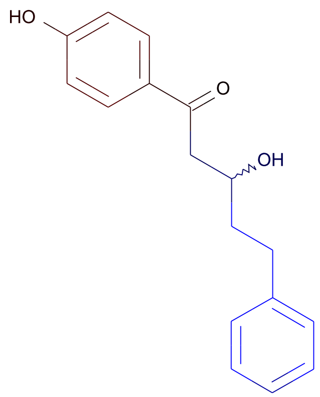

$C_{17}H_{18}O_3$
Molecular Weight: 270.323
ALogP: 3.293
Rotatable Bonds: 6
Acceptors: 3
Donors: 2

Model Prediction

Prediction: Non-Carcinogen
Probability: 0.191
Enrichment: 0.647
Bayesian Score: -5.6
Mahalanobis Distance: 10.4
Mahalanobis Distance p-value: 0.234

Prediction: Positive if the Bayesian score is above the estimated best cutoff value from minimizing the false positive and false negative rate.
Probability: The esimated probability that the sample is in the positive category. This assumes that the Bayesian score follows a normal distribution and is different from the prediction using a cutoff.
Enrichment: An estimate of enrichment, that is, the increased likelihood (versus random) of this sample being in the category.
Bayesian Score: The standard Laplacian-modified Bayesian score.
Mahalanobis Distance: The Mahalanobis distance (MD) is the distance to the center of the training data. The larger the MD, the less trustworthy the prediction.
Mahalanobis Distance p-value: The p-value gives the fraction of training data with an MD greater than or equal to the one for the given sample, assuming normally distributed data. The smaller the p-value, the less trustworthy the prediciton. For highly non-normal X properties (e.g., fingerprints), the MD p-value is wildly inaccurate.

Structural Similar Compounds

Name	Bunolol	Penbutalol	Propranolol
Structure	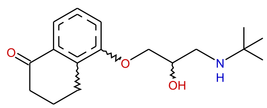	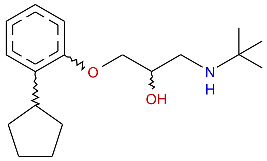	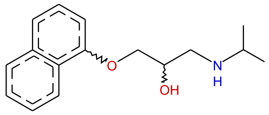
Actual Endpoint	Non-Carcinogen	Non-Carcinogen	Non-Carcinogen
Predicted Endpoint	Non-Carcinogen	Non-Carcinogen	Non-Carcinogen
Distance	0.521	0.546	0.547
Reference	US FDA (Centre for Drug Eval.& Res./Off. Testing & Res.) Sept. 1997	US FDA (Centre for Drug Eval.& Res./Off. Testing & Res.) Sept. 1997	US FDA (Centre for Drug Eval.& Res./Off. Testing & Res.) Sept. 1997

Model Applicability

Unknown features are fingerprint features in the query molecule, but not found or appearing too infrequently in the training set.

1. All properties and OPS components are within expected ranges.

Feature Contribution

Top features for positive contribution

Fingerprint	Bit/Smiles	Feature Structure	Score	Carcinogen in training set
FCFP_6	-1066794953	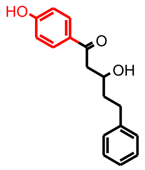 [*][c]1:[cH]:[cH]:[c](O):[cH]:[cH]:1	0.668	3 out of 4

FCFP_6	-1847351220	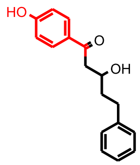<br>[*]=C[c]1:[cH]:[cH]:[c](O):[cH]:[cH]:1	0.547	3 out of 5
FCFP_6	-463243689	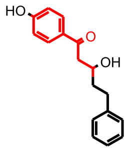<br>[*]C([*])CC(=O)[c]1:[cH]:[cH]:[c]([*]):[c H]:[cH]:1	0.46	1 out of 1

Top Features for negative contribution

Fingerprint	Bit/Smiles	Feature Structure	Score	Carcinogen in training set
FCFP_6	1981711554	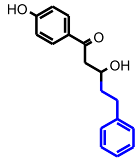<br>[*]CC[c]1:[cH]:[cH]:[cH]:[cH]:[cH]:1	-1.42	0 out of 12
FCFP_6	1388176727	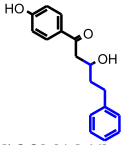<br>[*]C([*])CC[c]1:[cH]: [cH]:[cH]:[cH]:[cH]: 1	-1.21	0 out of 9
FCFP_6	-497728148	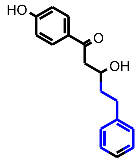<br>[*]CC[c]1:[cH]:[cH]:[*]:[cH]:[cH]:1	-0.96	2 out of 26

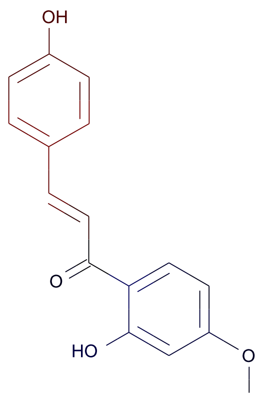

$C_{16}H_{14}O_4$
Molecular Weight: 270.28
ALogP: 3.201
Rotatable Bonds: 4
Acceptors: 4
Donors: 2

Model Prediction

Prediction: Carcinogen
Probability: 0.281
Enrichment: 0.953
Bayesian Score: -0.969
Mahalanobis Distance: 10.6
Mahalanobis Distance p-value: 0.149

Prediction: Positive if the Bayesian score is above the estimated best cutoff value from minimizing the false positive and false negative rate.
Probability: The esimated probability that the sample is in the positive category. This assumes that the Bayesian score follows a normal distribution and is different from the prediction using a cutoff.
Enrichment: An estimate of enrichment, that is, the increased likelihood (versus random) of this sample being in the category.
Bayesian Score: The standard Laplacian-modified Bayesian score.
Mahalanobis Distance: The Mahalanobis distance (MD) is the distance to the center of the training data. The larger the MD, the less trustworthy the prediction.
Mahalanobis Distance p-value: The p-value gives the fraction of training data with an MD greater than or equal to the one for the given sample, assuming normally distributed data. The smaller the p-value, the less trustworthy the prediciton. For highly non-normal X properties (e.g., fingerprints), the MD p-value is wildly inaccurate.

Structural Similar Compounds

Name	Cytembena	Mebendazole	Phenolphthalein
Structure	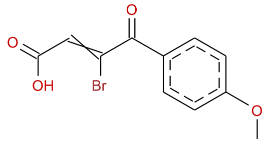	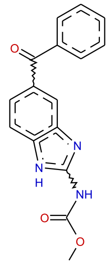	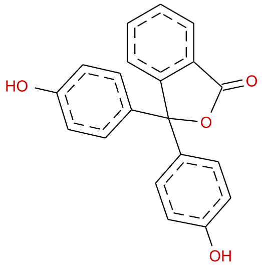
Actual Endpoint	Non-Carcinogen	Non-Carcinogen	Carcinogen
Predicted Endpoint	Non-Carcinogen	Non-Carcinogen	Carcinogen
Distance	0.519	0.526	0.549
Reference	US FDA (Centre for Drug Eval.& Res./Off. Testing & Res.) Sept. 1997	US FDA (Centre for Drug Eval.& Res./Off. Testing & Res.) Sept. 1997	US FDA (Centre for Drug Eval.& Res./Off. Testing & Res.) Sept. 1997

Model Applicability

Unknown features are fingerprint features in the query molecule, but not found or appearing too infrequently in the training set.

1. All properties and OPS components are within expected ranges.

Feature Contribution

Top features for positive contribution

Fingerprint	Bit/Smiles	Feature Structure	Score	Carcinogen in training set
FCFP_6	-146015125	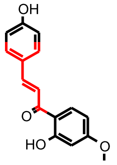 [*]C(=[*])\C=C\[c](:[cH]:[*]):[cH]:[*]	0.676	2 out of 2

FCFP_6	-1066794953	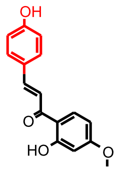 [*][c]1:[cH]:[cH]:[c](O):[cH]:[cH]:1	0.668	3 out of 4
FCFP_6	-1847351220	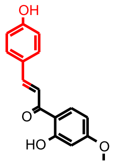 [*]=C[c]1:[cH]:[cH]:[c](O):[cH]:[cH]:1	0.547	3 out of 5

Top Features for negative contribution

Fingerprint	Bit/Smiles	Feature Structure	Score	Carcinogen in training set
FCFP_6	-1549192822	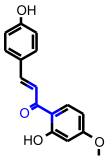 [*]CC(=O)[c](:[*]):[*]	-0.489	3 out of 21
FCFP_6	-1604301295	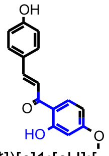 [*]C(=[*])[c]1:[cH]:[*]:[c]([*]):[cH]:[c]:1O	-0.445	2 out of 14
FCFP_6	7	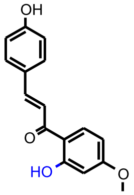 [*]O	-0.308	15 out of 79

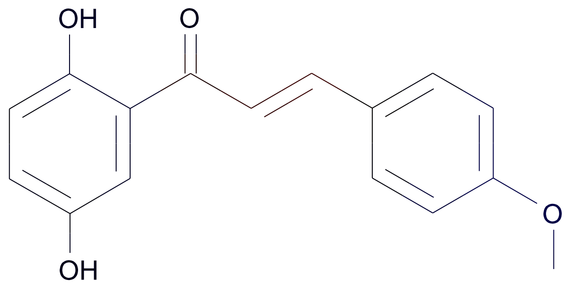

$C_{16}H_{14}O_4$
Molecular Weight: 270.28
ALogP: 3.201
Rotatable Bonds: 4
Acceptors: 4
Donors: 2

Model Prediction

Prediction: Non-Carcinogen
Probability: 0.238
Enrichment: 0.809
Bayesian Score: -2.87
Mahalanobis Distance: 10.6
Mahalanobis Distance p-value: 0.149

Prediction: Positive if the Bayesian score is above the estimated best cutoff value from minimizing the false positive and false negative rate.
Probability: The esimated probability that the sample is in the positive category. This assumes that the Bayesian score follows a normal distribution and is different from the prediction using a cutoff.
Enrichment: An estimate of enrichment, that is, the increased likelihood (versus random) of this sample being in the category.
Bayesian Score: The standard Laplacian-modified Bayesian score.
Mahalanobis Distance: The Mahalanobis distance (MD) is the distance to the center of the training data. The larger the MD, the less trustworthy the prediction.
Mahalanobis Distance p-value: The p-value gives the fraction of training data with an MD greater than or equal to the one for the given sample, assuming normally distributed data. The smaller the p-value, the less trustworthy the prediciton. For highly non-normal X properties (e.g., fingerprints), the MD p-value is wildly inaccurate.

Structural Similar Compounds

Name	Cytembena	Mebendazole	Etodolac
Structure	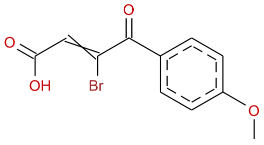	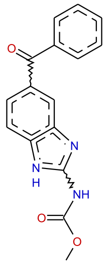	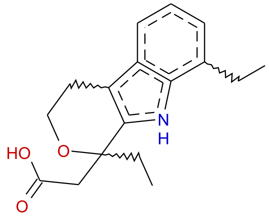
Actual Endpoint	Non-Carcinogen	Non-Carcinogen	Non-Carcinogen
Predicted Endpoint	Non-Carcinogen	Non-Carcinogen	Non-Carcinogen
Distance	0.510	0.526	0.562
Reference	US FDA (Centre for Drug Eval.& Res./Off. Testing & Res.) Sept. 1997	US FDA (Centre for Drug Eval.& Res./Off. Testing & Res.) Sept. 1997	US FDA (Centre for Drug Eval.& Res./Off. Testing & Res.) Sept. 1997

Model Applicability

Unknown features are fingerprint features in the query molecule, but not found or appearing too infrequently in the training set.

1. All properties and OPS components are within expected ranges.

Feature Contribution

Top features for positive contribution

Fingerprint	Bit/Smiles	Feature Structure	Score	Carcinogen in training set
FCFP_6	-146015125	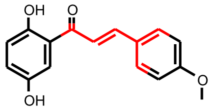 [*]C(=[*])\C=C\[c](:[cH]:[*]):[cH]:[*]	0.676	2 out of 2

FCFP_6	451847724	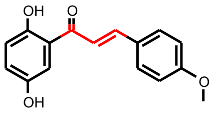 [*]\C=C\C(=[*])[*]	0.479	21 out of 48
FCFP_6	451371068	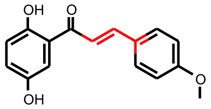 [*]\C=C\[c](:[*]):[*]	0.439	3 out of 6
**Top Features for negative contribution**				
**Fingerprint**	**Bit/Smiles**	**Feature Structure**	**Score**	**Carcinogen in training set**
FCFP_6	-9847677	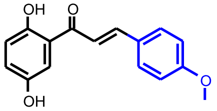 [*][c]1:[cH]:[cH]:[c](OC):[cH]:[cH]:1	-0.719	0 out of 4
FCFP_6	-1549192822	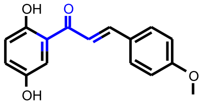 [*]CC(=O)[c](:[*]):[*]	-0.489	3 out of 21
FCFP_6	-1604301295	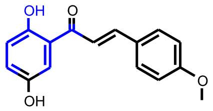 [*]C(=[*])[c]1:[cH]:[*]:[c]([*]):[cH]:[c]:1O	-0.445	2 out of 14

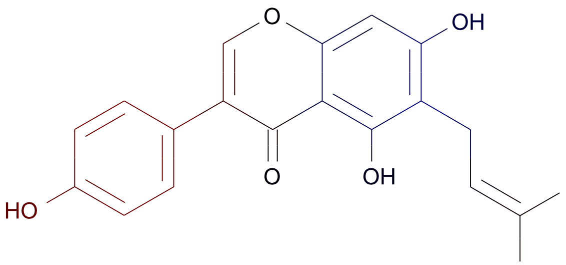

$C_{20}H_{18}O_5$
Molecular Weight: 338.354
ALogP: 3.997
Rotatable Bonds: 3
Acceptors: 5
Donors: 3

Model Prediction

Prediction: Non-Carcinogen
Probability: 0.242
Enrichment: 0.822
Bayesian Score: -2.67
Mahalanobis Distance: 13.1
Mahalanobis Distance p-value: 0.000221

Prediction: Positive if the Bayesian score is above the estimated best cutoff value from minimizing the false positive and false negative rate.
Probability: The esimated probability that the sample is in the positive category. This assumes that the Bayesian score follows a normal distribution and is different from the prediction using a cutoff.
Enrichment: An estimate of enrichment, that is, the increased likelihood (versus random) of this sample being in the category.
Bayesian Score: The standard Laplacian-modified Bayesian score.
Mahalanobis Distance: The Mahalanobis distance (MD) is the distance to the center of the training data. The larger the MD, the less trustworthy the prediction.
Mahalanobis Distance p-value: The p-value gives the fraction of training data with an MD greater than or equal to the one for the given sample, assuming normally distributed data. The smaller the p-value, the less trustworthy the prediciton. For highly non-normal X properties (e.g., fingerprints), the MD p-value is wildly inaccurate.

Structural Similar Compounds

Name	Phenolphthalein	Niclosamide	Torsemide
Structure	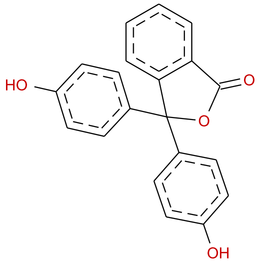	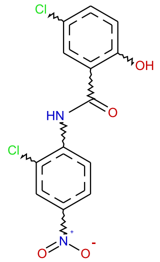	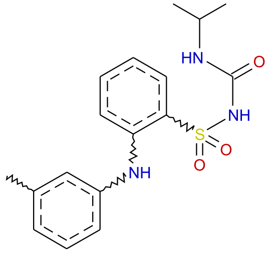
Actual Endpoint	Carcinogen	Non-Carcinogen	Non-Carcinogen
Predicted Endpoint	Carcinogen	Non-Carcinogen	Non-Carcinogen
Distance	0.584	0.617	0.619
Reference	US FDA (Centre for Drug Eval.& Res./Off. Testing & Res.) Sept. 1997	US FDA (Centre for Drug Eval.& Res./Off. Testing & Res.) Sept. 1997	US FDA (Centre for Drug Eval.& Res./Off. Testing & Res.) Sept. 1997

Model Applicability

Unknown features are fingerprint features in the query molecule, but not found or appearing too infrequently in the training set.

1. All properties and OPS components are within expected ranges.

Feature Contribution

Top features for positive contribution

Fingerprint	Bit/Smiles	Feature Structure	Score	Carcinogen in training set
FCFP_6	-1066794953	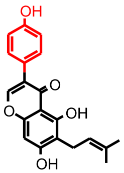 [*][c]1:[cH]:[cH]:[c](O):[cH]:[cH]:1	0.668	3 out of 4

FCFP_6	-1847351220	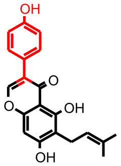 [*]=C[c]1:[cH]:[cH]:[c](O):[cH]:[cH]:1	0.547	3 out of 5
FCFP_6	451847724	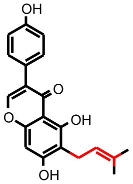 [*]\C=C\C(=[*])[*]	0.479	21 out of 48

Top Features for negative contribution

Fingerprint	Bit/Smiles	Feature Structure	Score	Carcinogen in training set
FCFP_6	-1601875224	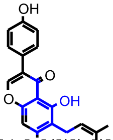 [*]C[c]1:[c]([*]):[*]:[c]([*]):[c](C(=[*])[*]):[c]:1O	-0.582	0 out of 3
FCFP_6	-1549192822	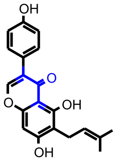 [*]CC(=O)[c](:[*]):[*]	-0.489	3 out of 21
FCFP_6	-1604301295	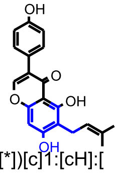 [*]C(=[*])[c]1:[cH]:[*]:[c]([*]):[cH]:[c]:1O	-0.445	2 out of 14

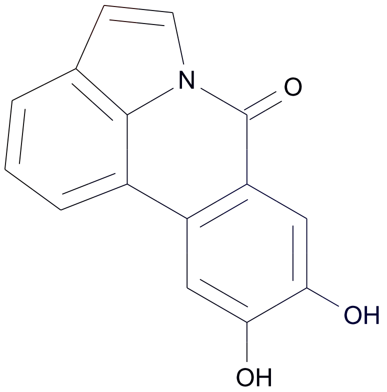

$C_{15}H_9NO_3$
Molecular Weight: 251.237
ALogP: 2.982
Rotatable Bonds: 0
Acceptors: 3
Donors: 2

Model Prediction

Prediction: Carcinogen
Probability: 0.276
Enrichment: 0.938
Bayesian Score: -1.16
Mahalanobis Distance: 14.6
Mahalanobis Distance p-value: 6.24e-007

Prediction: Positive if the Bayesian score is above the estimated best cutoff value from minimizing the false positive and false negative rate.
Probability: The esimated probability that the sample is in the positive category. This assumes that the Bayesian score follows a normal distribution and is different from the prediction using a cutoff.
Enrichment: An estimate of enrichment, that is, the increased likelihood (versus random) of this sample being in the category.
Bayesian Score: The standard Laplacian-modified Bayesian score.
Mahalanobis Distance: The Mahalanobis distance (MD) is the distance to the center of the training data. The larger the MD, the less trustworthy the prediction.
Mahalanobis Distance p-value: The p-value gives the fraction of training data with an MD greater than or equal to the one for the given sample, assuming normally distributed data. The smaller the p-value, the less trustworthy the prediciton. For highly non-normal X properties (e.g., fingerprints), the MD p-value is wildly inaccurate.

Structural Similar Compounds

Name	Danthron	Diflunisal	Oxazepam
Structure	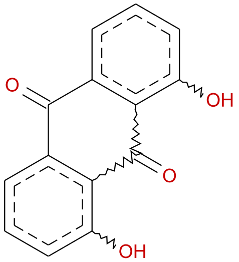	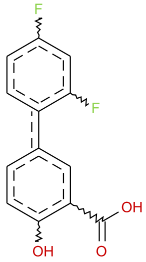	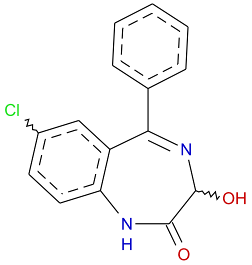
Actual Endpoint	Carcinogen	Non-Carcinogen	Carcinogen
Predicted Endpoint	Carcinogen	Non-Carcinogen	Carcinogen
Distance	0.553	0.556	0.565
Reference	US FDA (Centre for Drug Eval.& Res./Off. Testing & Res.) Sept. 1997	US FDA (Centre for Drug Eval.& Res./Off. Testing & Res.) Sept. 1997	US FDA (Centre for Drug Eval.& Res./Off. Testing & Res.) Sept. 1997

Model Applicability

Unknown features are fingerprint features in the query molecule, but not found or appearing too infrequently in the training set.

1. All properties and OPS components are within expected ranges.

Feature Contribution

Top features for positive contribution

Fingerprint	Bit/Smiles	Feature Structure	Score	Carcinogen in training set
FCFP_6	-1320007763	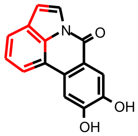 [*]1:[*]:[c]2:[*]:[cH]:[cH]:[cH]:[c]:2:[cH]:1	0.348	6 out of 15

FCFP_6	1804743636	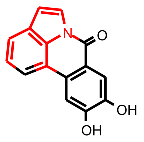<br>[*]n1:[cH]:[cH]:[c]2:[cH]:[cH]:[*]:[c]([*]):[c]:1:2	0.271	1 out of 2
FCFP_6	-124655670	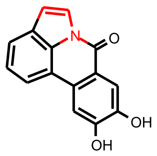<br>[*]n1:[*]:[*]:[cH]:[cH]:1	0.264	14 out of 40
**Top Features for negative contribution**				
**Fingerprint**	**Bit/Smiles**	**Feature Structure**	**Score**	**Carcinogen in training set**
FCFP_6	7	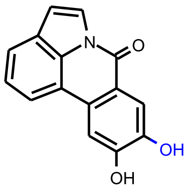<br>[*]O	-0.308	15 out of 79
FCFP_6	-306856457	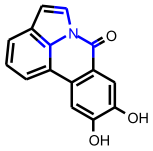<br>[*]C(=[*])n1:[cH]:[*]:[*]:[c]:1:[*]	-0.264	3 out of 16
FCFP_6	-549108873	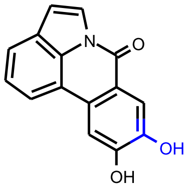<br>[*]:[c](:[*])O	-0.243	7 out of 35

remdesivir

TOPKAT_Mouse_Male_FDA_None_vs_Carcinogen

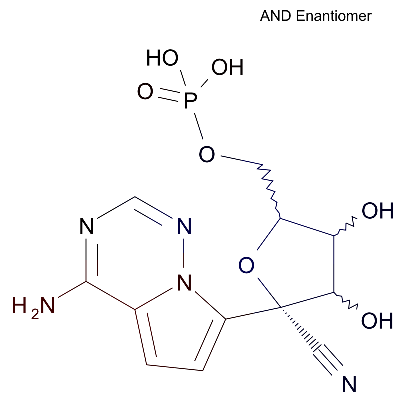

$C_{12}H_{14}N_5O_7P$

Molecular Weight: 371.24262

ALogP: -1.502

Rotatable Bonds: 4

Acceptors: 11

Donors: 5

Model Prediction

Prediction: Non-Carcinogen

Probability: 0.239

Enrichment: 0.812

Bayesian Score: -2.82

Mahalanobis Distance: 19.2

Mahalanobis Distance p-value: 7.81e-017

Prediction: Positive if the Bayesian score is above the estimated best cutoff value from minimizing the false positive and false negative rate.
Probability: The esimated probability that the sample is in the positive category. This assumes that the Bayesian score follows a normal distribution and is different from the prediction using a cutoff.
Enrichment: An estimate of enrichment, that is, the increased likelihood (versus random) of this sample being in the category.
Bayesian Score: The standard Laplacian-modified Bayesian score.
Mahalanobis Distance: The Mahalanobis distance (MD) is the distance to the center of the training data. The larger the MD, the less trustworthy the prediction.
Mahalanobis Distance p-value: The p-value gives the fraction of training data with an MD greater than or equal to the one for the given sample, assuming normally distributed data. The smaller the p-value, the less trustworthy the prediciton. For highly non-normal X properties (e.g., fingerprints), the MD p-value is wildly inaccurate.

Structural Similar Compounds

Name	Famotidine	Tetracycline	Ribavirin
Structure	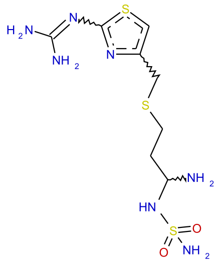	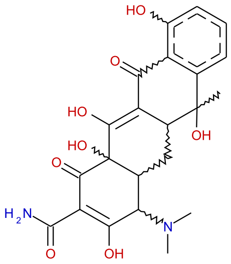	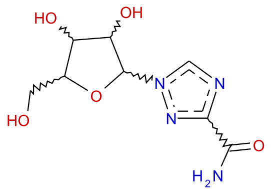
Actual Endpoint	Non-Carcinogen	Non-Carcinogen	Non-Carcinogen
Predicted Endpoint	Non-Carcinogen	Non-Carcinogen	Non-Carcinogen
Distance	0.813	0.843	0.860
Reference	US FDA (Centre for Drug Eval.& Res./Off. Testing & Res.) Sept. 1997	US FDA (Centre for Drug Eval.& Res./Off. Testing & Res.) Sept. 1997	US FDA (Centre for Drug Eval.& Res./Off. Testing & Res.) Sept. 1997

Model Applicability

Unknown features are fingerprint features in the query molecule, but not found or appearing too infrequently in the training set.

1. All properties and OPS components are within expected ranges.
2. Unknown FCFP_2 feature: 472180098: [*]OP(=O)(O)O
3. Unknown FCFP_2 feature: -332197802: [*][c]1:[*]:[*]:[c](:[*]):n:1:n:[*]

Feature Contribution

Top features for positive contribution

Fingerprint	Bit/Smiles	Feature Structure	Score	Carcinogen in training set
FCFP_6	-450797925	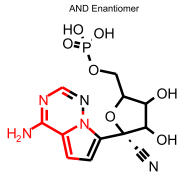 N[c]1:n:[cH]:[*]:n2:[*]:[*]:[cH]:[c]:1:2	0.676	2 out of 2

FCFP_6	-1151884458	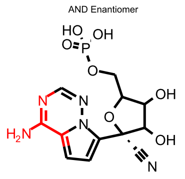 [*]:n:[c](N):[c](:[*]):[*]	0.348	6 out of 15
FCFP_6	-1280036918	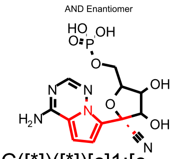 [*]C([*])([*])[c]1:[cH]:[cH]:[c](:[*]):n:1:[*]	0.333	7 out of 18
**Top Features for negative contribution**				
**Fingerprint**	**Bit/Smiles**	**Feature Structure**	**Score**	**Carcinogen in training set**
FCFP_6	-124685461	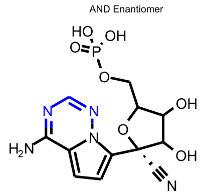 [*]:n:[cH]:n:[*]	-0.731	1 out of 12
FCFP_6	422052003	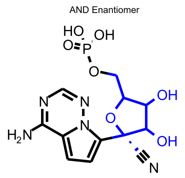 [*]CC1OC([*])([*])C(O)C1O	-0.582	0 out of 3
FCFP_6	-1277879912	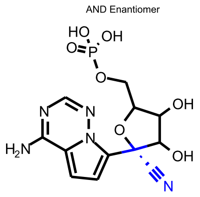 [*]C([*])([*])C#N	-0.582	0 out of 3

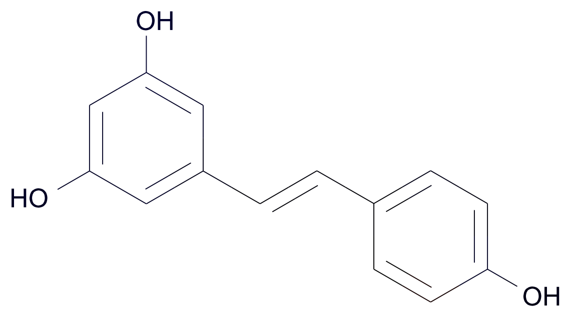

$C_{14}H_{12}O_3$
Molecular Weight: 228.243
ALogP: 3.09
Rotatable Bonds: 2
Acceptors: 3
Donors: 3

Model Prediction

Prediction: Multiple-Carcinogen
Probability: 0.174
Enrichment: 0.579
Bayesian Score: -4.3
Mahalanobis Distance: 7.02
Mahalanobis Distance p-value: 0.602

Prediction: Positive if the Bayesian score is above the estimated best cutoff value from minimizing the false positive and false negative rate.
Probability: The esimated probability that the sample is in the positive category. This assumes that the Bayesian score follows a normal distribution and is different from the prediction using a cutoff.
Enrichment: An estimate of enrichment, that is, the increased likelihood (versus random) of this sample being in the category.
Bayesian Score: The standard Laplacian-modified Bayesian score.
Mahalanobis Distance: The Mahalanobis distance (MD) is the distance to the center of the training data. The larger the MD, the less trustworthy the prediction.
Mahalanobis Distance p-value: The p-value gives the fraction of training data with an MD greater than or equal to the one for the given sample, assuming normally distributed data. The smaller the p-value, the less trustworthy the prediciton. For highly non-normal X properties (e.g., fingerprints), the MD p-value is wildly inaccurate.

Structural Similar Compounds

Name	Phenolphthalein	Danthron	Metaproterenol
Structure	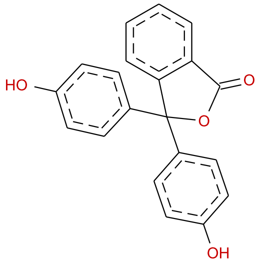	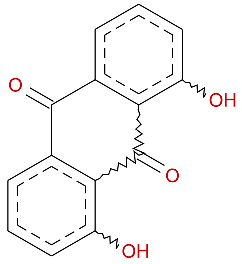	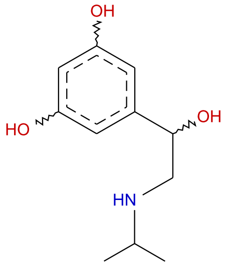
Actual Endpoint	Multiple-Carcinogen	Single-Carcinogen	Single-Carcinogen
Predicted Endpoint	Multiple-Carcinogen	Single-Carcinogen	Single-Carcinogen
Distance	0.685	0.692	0.697
Reference	US FDA (Centre for Drug Eval.& Res./Off. Testing & Res.) Sept. 1997	US FDA (Centre for Drug Eval.& Res./Off. Testing & Res.) Sept. 1997	US FDA (Centre for Drug Eval.& Res./Off. Testing & Res.) Sept. 1997

Model Applicability

Unknown features are fingerprint features in the query molecule, but not found or appearing too infrequently in the training set.

1. All properties and OPS components are within expected ranges.

Feature Contribution

Top features for positive contribution

Fingerprint	Bit/Smiles	Feature Structure	Score	Multiple-Carcinogen in training set
FCFP_12	-1847351220	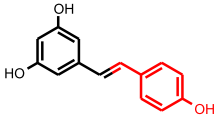 [*]=C[c]1:[cH]:[cH]:[c](O):[cH]:[cH]:1	0.395	2 out of 3

FCFP_12	949015626	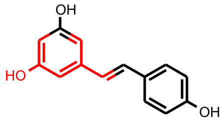 [*]=C[c]1:[cH]:[*]:[cH]:[c](O):[cH]:1	0.174	1 out of 2
FCFP_12	-158888774	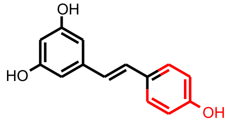 O[c]1:[cH]:[cH]:[*]:[cH]:[cH]:1	0.105	2 out of 5

Top Features for negative contribution

Fingerprint	Bit/Smiles	Feature Structure	Score	Multiple-Carcinogen in training set
FCFP_12	7	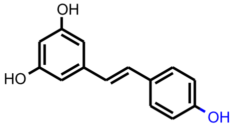 [*]O	-0.71	2 out of 15
FCFP_12	1618154665	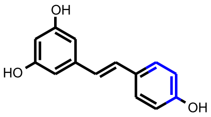 [*][c](:[*]):[cH]:[cH]:[*]	-0.409	13 out of 59
FCFP_12	16	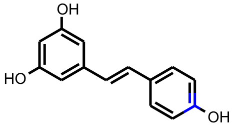 [*][c](:[*]):[*]	-0.308	16 out of 65

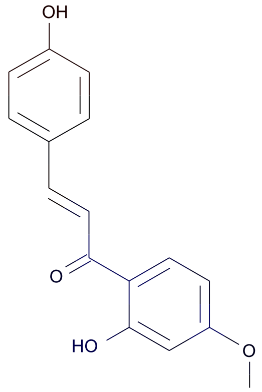

$C_{16}H_{14}O_4$
Molecular Weight: 270.28
ALogP: 3.201
Rotatable Bonds: 4
Acceptors: 4
Donors: 2

Model Prediction

Prediction: Single-Carcinogen
Probability: 0.158
Enrichment: 0.524
Bayesian Score: -6.02
Mahalanobis Distance: 11.9
Mahalanobis Distance p-value: 0.000679

Prediction: Positive if the Bayesian score is above the estimated best cutoff value from minimizing the false positive and false negative rate.
Probability: The esimated probability that the sample is in the positive category. This assumes that the Bayesian score follows a normal distribution and is different from the prediction using a cutoff.
Enrichment: An estimate of enrichment, that is, the increased likelihood (versus random) of this sample being in the category.
Bayesian Score: The standard Laplacian-modified Bayesian score.
Mahalanobis Distance: The Mahalanobis distance (MD) is the distance to the center of the training data. The larger the MD, the less trustworthy the prediction.
Mahalanobis Distance p-value: The p-value gives the fraction of training data with an MD greater than or equal to the one for the given sample, assuming normally distributed data. The smaller the p-value, the less trustworthy the prediciton. For highly non-normal X properties (e.g., fingerprints), the MD p-value is wildly inaccurate.

Structural Similar Compounds

Name	Phenolphthalein	Suprofen	Oxazepam
Structure	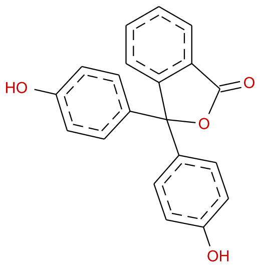	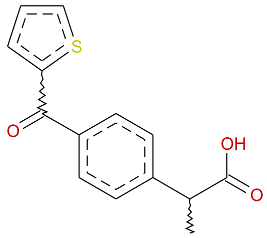	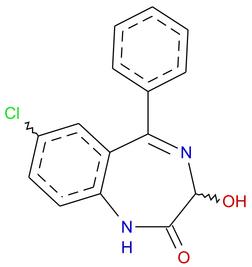
Actual Endpoint	Multiple-Carcinogen	Single-Carcinogen	Single-Carcinogen
Predicted Endpoint	Multiple-Carcinogen	Single-Carcinogen	Single-Carcinogen
Distance	0.590	0.693	0.695
Reference	US FDA (Centre for Drug Eval.& Res./Off. Testing & Res.) Sept. 1997	US FDA (Centre for Drug Eval.& Res./Off. Testing & Res.) Sept. 1997	US FDA (Centre for Drug Eval.& Res./Off. Testing & Res.) Sept. 1997

Model Applicability

Unknown features are fingerprint features in the query molecule, but not found or appearing too infrequently in the training set.

1. All properties and OPS components are within expected ranges.

Feature Contribution

Top features for positive contribution

Fingerprint	Bit/Smiles	Feature Structure	Score	Multiple-Carcinogen in training set
FCFP_12	-1847351220	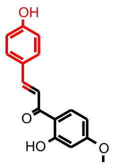 [*]=C[c]1:[cH]:[cH]:[c](O):[cH]:[cH]:1	0.395	2 out of 3

FCFP_12	451847724	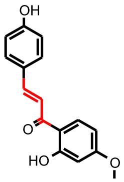 [*]\C=C\C(=[*])[*]	0.3	10 out of 21
FCFP_12	-1034142694	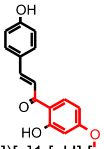 [*]C(=[*])[c]1:[cH]:[cH]:[c](OC):[cH]:[c]:1[*]	0.239	2 out of 4
**Top Features for negative contribution**				
**Fingerprint**	**Bit/Smiles**	**Feature Structure**	**Score**	**Multiple-Carcinogen in training set**
FCFP_12	7	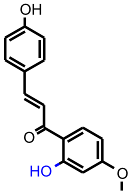 [*]O	-0.71	2 out of 15
FCFP_12	-1549192822	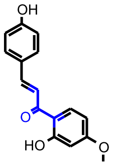 [*]=CC(=O)[c](:[*]):[*]	-0.704	0 out of 3
FCFP_12	-146015125	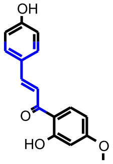 [*]C(=[*])\C=C\[c](:[cH]:[*]):[cH]:[*]	-0.519	0 out of 2

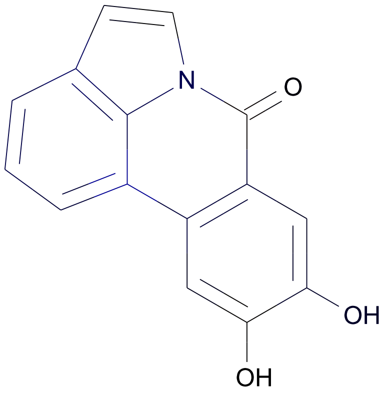

$C_{15}H_9NO_3$
Molecular Weight: 251.237
ALogP: 2.982
Rotatable Bonds: 0
Acceptors: 3
Donors: 2

Model Prediction

Prediction: Single-Carcinogen
Probability: 0.149
Enrichment: 0.496
Bayesian Score: -7.56
Mahalanobis Distance: 12.6
Mahalanobis Distance p-value: 0.000199

Prediction: Positive if the Bayesian score is above the estimated best cutoff value from minimizing the false positive and false negative rate.
Probability: The esimated probability that the sample is in the positive category. This assumes that the Bayesian score follows a normal distribution and is different from the prediction using a cutoff.
Enrichment: An estimate of enrichment, that is, the increased likelihood (versus random) of this sample being in the category.
Bayesian Score: The standard Laplacian-modified Bayesian score.
Mahalanobis Distance: The Mahalanobis distance (MD) is the distance to the center of the training data. The larger the MD, the less trustworthy the prediction.
Mahalanobis Distance p-value: The p-value gives the fraction of training data with an MD greater than or equal to the one for the given sample, assuming normally distributed data. The smaller the p-value, the less trustworthy the prediciton. For highly non-normal X properties (e.g., fingerprints), the MD p-value is wildly inaccurate.

Structural Similar Compounds

Name	Oxazepam	Danthron	Proflavine
Structure	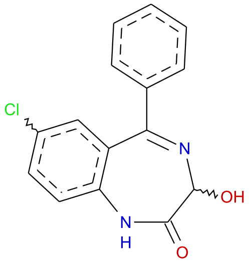	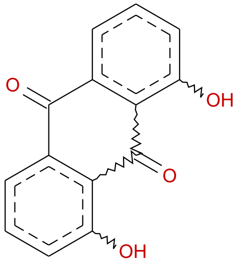	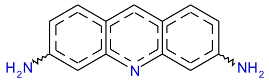
Actual Endpoint	Single-Carcinogen	Single-Carcinogen	Single-Carcinogen
Predicted Endpoint	Single-Carcinogen	Single-Carcinogen	Single-Carcinogen
Distance	0.577	0.580	0.586
Reference	US FDA (Centre for Drug Eval.& Res./Off. Testing & Res.) Sept. 1997	US FDA (Centre for Drug Eval.& Res./Off. Testing & Res.) Sept. 1997	US FDA (Centre for Drug Eval.& Res./Off. Testing & Res.) Sept. 1997

Model Applicability

Unknown features are fingerprint features in the query molecule, but not found or appearing too infrequently in the training set.

1. All properties and OPS components are within expected ranges.
2. Unknown FCFP_2 feature: -1549639687: [*]:n(:[*])C(=O)[c](:[*]):[*]

Feature Contribution

Top features for positive contribution

Fingerprint	Bit/Smiles	Feature Structure	Score	Multiple-Carcinogen in training set
FCFP_12	-306856457	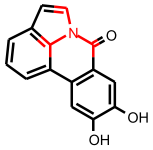 [*]C(=[*])n1:[cH]:[*]:[*]:[c]:1:[*]	0.395	2 out of 3

FCFP_12	949015626	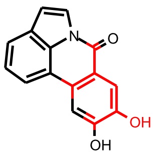 [*]=C[c]1:[cH]:[*]:[cH]:[c](O):[cH]:1	0.174	1 out of 2
**Top Features for negative contribution**				
**Fingerprint**	**Bit/Smiles**	**Feature Structure**	**Score**	**Multiple-Carcinogen in training set**
FCFP_12	178336375	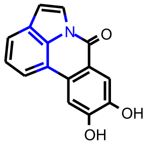 [*]n1:[*]:[*]:[c](:[*]):[c]:1:[c]([*]):[*]	-0.994	0 out of 5
FCFP_12	7	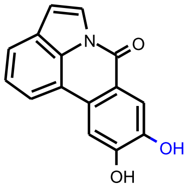 [*]O	-0.71	2 out of 15
FCFP_12	991735244	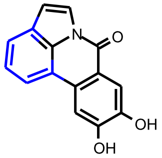 [*][c]1:[*]:[c](:[*]):[cH]:[cH]:[cH]:1	-0.562	5 out of 28

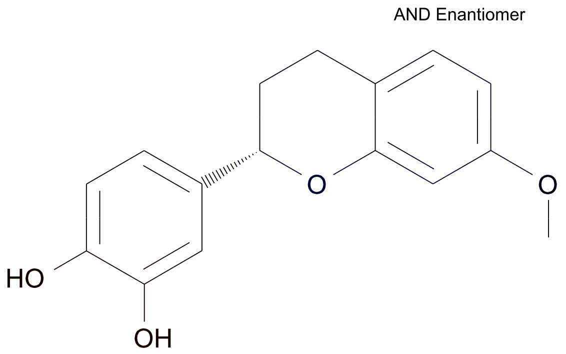

$C_{16}H_{16}O_4$
Molecular Weight: 272.296
ALogP: 3.456
Rotatable Bonds: 2
Acceptors: 4
Donors: 2

Structural Similar Compounds

Name	BENZOIN; OXIME	2;4-Dihydroxybenzophenone	PHENOL; 4;4'-THIODI-
Structure	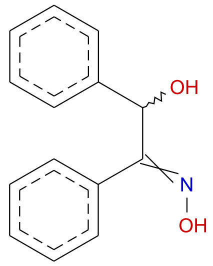	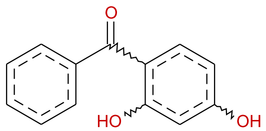	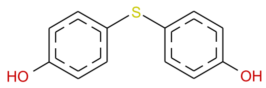
Actual Endpoint	Mild	Mild	Moderate_Severe
Predicted Endpoint	Mild	Mild	Moderate_Severe
Distance	0.606	0.609	0.619
Reference	28ZPAK-;111;72	J. Am. Coll. Toxicol. 2(5):35;1983	BIOFX* A408;71

Model Prediction

Prediction: Mild
Probability: 0.803
Enrichment: 1.16
Bayesian Score: -0.904
Mahalanobis Distance: 7.44
Mahalanobis Distance p-value: 0.988

Prediction: Positive if the Bayesian score is above the estimated best cutoff value from minimizing the false positive and false negative rate.
Probability: The esimated probability that the sample is in the positive category. This assumes that the Bayesian score follows a normal distribution and is different from the prediction using a cutoff.
Enrichment: An estimate of enrichment, that is, the increased likelihood (versus random) of this sample being in the category.
Bayesian Score: The standard Laplacian-modified Bayesian score.
Mahalanobis Distance: The Mahalanobis distance (MD) is the distance to the center of the training data. The larger the MD, the less trustworthy the prediction.
Mahalanobis Distance p-value: The p-value gives the fraction of training data with an MD greater than or equal to the one for the given sample, assuming normally distributed data. The smaller the p-value, the less trustworthy the prediciton. For highly non-normal X properties (e.g., fingerprints), the MD p-value is wildly inaccurate.

Model Applicability

Unknown features are fingerprint features in the query molecule, but not found or appearing too infrequently in the training set.

1. All properties and OPS components are within expected ranges.

Feature Contribution

Top features for positive contribution

Fingerprint	Bit/Smiles	Feature Structure	Score	Moderate_Severe in training set
FCFP_10	-1034142694	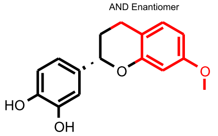 [*]C[c]1:[cH]:[cH]:[c](OC):[cH]:[c]:1[*]	0.338	18 out of 19

FCFP_10	1679744180	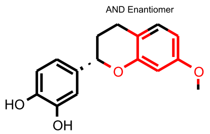<br>[*]O[c]1:[cH]:[*]:[c]([*]):[c](O[*]):[cH]:1	0.256	2 out of 2
FCFP_10	7	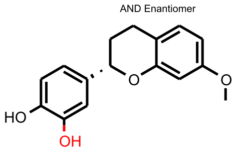<br>[*]O	0.219	117 out of 142
**Top Features for negative contribution**				
**Fingerprint**	**Bit/Smiles**	**Feature Structure**	**Score**	**Moderate_Severe in training set**
FCFP_10	-1977641857	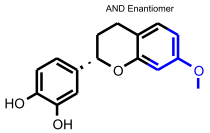<br>[*]:[cH]:[c](OC):[cH]:[*]	-0.78	4 out of 15
FCFP_10	184987616	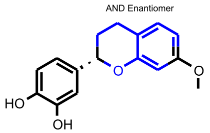<br>[*]1CC[c]2:[cH]:[cH]:[*]:[cH]:[c]:2O1	-0.507	0 out of 1
FCFP_10	-1099193755	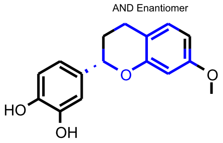<br>[*][C@@H]1[*]C[c]2:[cH]:[*]:[c]([*]):[cH]:[c]:2O1	-0.361	2 out of 5

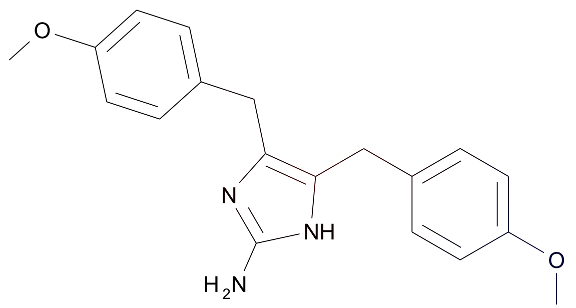

$C_{19}H_{21}N_3O_2$
Molecular Weight: 323.389
ALogP: 3.305
Rotatable Bonds: 6
Acceptors: 4
Donors: 2

Model Prediction

Prediction: Moderate_Severe
Probability: 0.814
Enrichment: 1.18
Bayesian Score: -0.392
Mahalanobis Distance: 6.65
Mahalanobis Distance p-value: 1

Prediction: Positive if the Bayesian score is above the estimated best cutoff value from minimizing the false positive and false negative rate.
Probability: The esimated probability that the sample is in the positive category. This assumes that the Bayesian score follows a normal distribution and is different from the prediction using a cutoff.
Enrichment: An estimate of enrichment, that is, the increased likelihood (versus random) of this sample being in the category.
Bayesian Score: The standard Laplacian-modified Bayesian score.
Mahalanobis Distance: The Mahalanobis distance (MD) is the distance to the center of the training data. The larger the MD, the less trustworthy the prediction.
Mahalanobis Distance p-value: The p-value gives the fraction of training data with an MD greater than or equal to the one for the given sample, assuming normally distributed data. The smaller the p-value, the less trustworthy the prediciton. For highly non-normal X properties (e.g., fingerprints), the MD p-value is wildly inaccurate.

Structural Similar Compounds

Name	1-AMINO-4-BENZOYLAMINO-ANTHRAQUINONE	ANTHRAQUINONE; 1-AMINO-4-HYDROXY-2-PHENOXY-	s-TRIAZINE; 2-CHLORO-4;6-BIS(ISOPROPYLAMINO)-
Structure	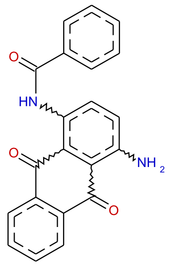	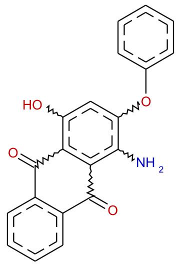	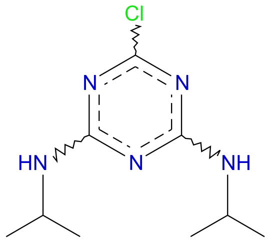
Actual Endpoint	Mild	Mild	Mild
Predicted Endpoint	Mild	Mild	Mild
Distance	0.620	0.642	0.674
Reference	28ZPAK-;124;72	28ZPAK 239;72	CIGET 77

Model Applicability

Unknown features are fingerprint features in the query molecule, but not found or appearing too infrequently in the training set.

1. All properties and OPS components are within expected ranges.
2. Unknown FCFP_2 feature: 203707511: [*]C[c]1:[nH]:[*]:[*]:[c]:1[*]
3. Unknown FCFP_2 feature: -1151854667: N[c]1:[nH]:[*]:[*]:n:1

Feature Contribution

Top features for positive contribution

Fingerprint	Bit/Smiles	Feature Structure	Score	Moderate_Severe in training set
FCFP_10	-1034142694	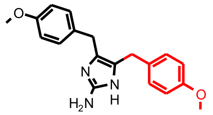 [*]C[c]1:[cH]:[cH]:[c](OC):[cH]:[c]:1[*]	0.338	18 out of 19

FCFP_10	906530397	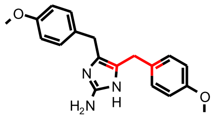<br>[*]:[c](:[*])C[c](:[*<br>]):[*]	0.332	5 out of 5
FCFP_10	1758843539	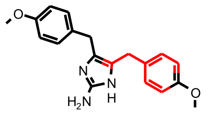<br>[*]:[c](:[*])C[c]1:[c<br>H]:[cH]:[*]:[cH]:[cH<br>]:1	0.294	3 out of 3
**Top Features for negative contribution**				
**Fingerprint**	**Bit/Smiles**	**Feature Structure**	**Score**	**Moderate_Severe in training set**
FCFP_10	-1977641857	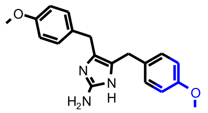<br>[*]:[cH]:[c](OC):[cH]<br>:[*]	-0.78	4 out of 15
FCFP_10	-9847677	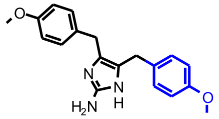<br>[*][c]1:[cH]:[cH]:[c]<br>(OC):[cH]:[cH]:1	-0.4	1 out of 3
FCFP_10	136627117	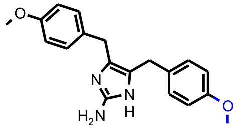<br>[*]OC	-0.316	46 out of 96

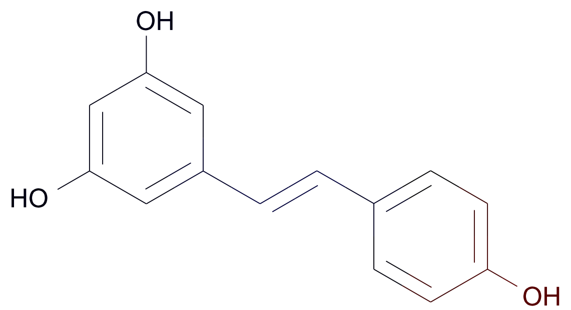

$C_{14}H_{12}O_3$
Molecular Weight: 228.243
ALogP: 3.09
Rotatable Bonds: 2
Acceptors: 3
Donors: 3

Model Prediction

Prediction: Moderate_Severe
Probability: 0.814
Enrichment: 1.18
Bayesian Score: -0.415
Mahalanobis Distance: 5.92
Mahalanobis Distance p-value: 1

Prediction: Positive if the Bayesian score is above the estimated best cutoff value from minimizing the false positive and false negative rate.
Probability: The esimated probability that the sample is in the positive category. This assumes that the Bayesian score follows a normal distribution and is different from the prediction using a cutoff.
Enrichment: An estimate of enrichment, that is, the increased likelihood (versus random) of this sample being in the category.
Bayesian Score: The standard Laplacian-modified Bayesian score.
Mahalanobis Distance: The Mahalanobis distance (MD) is the distance to the center of the training data. The larger the MD, the less trustworthy the prediction.
Mahalanobis Distance p-value: The p-value gives the fraction of training data with an MD greater than or equal to the one for the given sample, assuming normally distributed data. The smaller the p-value, the less trustworthy the prediciton. For highly non-normal X properties (e.g., fingerprints), the MD p-value is wildly inaccurate.

Structural Similar Compounds

Name	METHANE;TRIS(4-AMINOPHENYL)-	2;4-Dihydroxybenzophenone	4;4'METHYLENE DIPHENOL
Structure	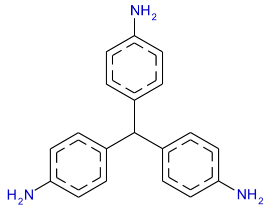	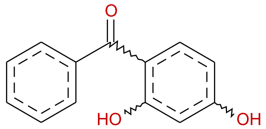	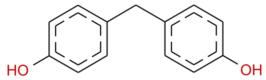
Actual Endpoint	Moderate_Severe	Mild	Moderate_Severe
Predicted Endpoint	Moderate_Severe	Mild	Moderate_Severe
Distance	0.564	0.623	0.633
Reference	28ZPAK-;73;72	J. Am. Coll. Toxicol. 2(5):35;1983	AIHAAP 23;95;62

Model Applicability

Unknown features are fingerprint features in the query molecule, but not found or appearing too infrequently in the training set.

1. All properties and OPS components are within expected ranges.

Feature Contribution

Top features for positive contribution

Fingerprint	Bit/Smiles	Feature Structure	Score	Moderate_Severe in training set
FCFP_10	-1066794953	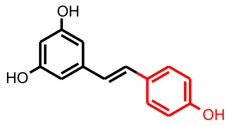 [*][c]1:[cH]:[cH]:[c](O):[cH]:[cH]:1	0.378	13 out of 13

FCFP_10	-158888774	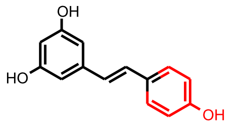 O[c]1:[cH]:[cH]:[*]:[cH]:[cH]:1	0.356	24 out of 25
FCFP_10	-1847351220	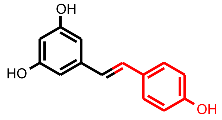 [*]=C[c]1:[cH]:[cH]:[c](O):[cH]:[cH]:1	0.273	9 out of 10
**Top Features for negative contribution**				
**Fingerprint**	**Bit/Smiles**	**Feature Structure**	**Score**	**Moderate_Severe in training set**
FCFP_10	-1748394506	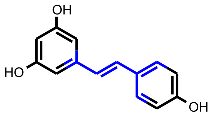 [*]:[cH]:[c](\C=C\[c](:[*]):[*]):[cH]:[*]	-0.842	0 out of 2
FCFP_10	946068634	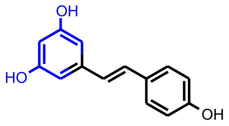 O[c]1:[cH]:[*]:[cH]:[c](O):[cH]:1	-0.4	1 out of 3
FCFP_10	451371068	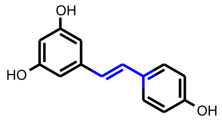 [*]\C=C\[c](:[*]):[*]	-0.216	3 out of 6

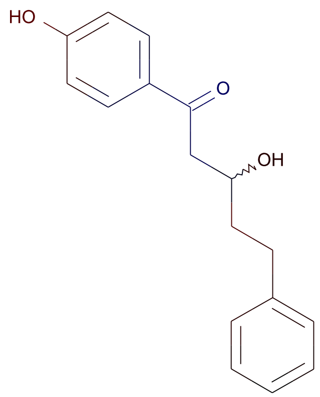

$C_{17}H_{18}O_3$
Molecular Weight: 270.323
ALogP: 3.293
Rotatable Bonds: 6
Acceptors: 3
Donors: 2

Model Prediction

Prediction: Moderate_Severe
Probability: 0.833
Enrichment: 1.21
Bayesian Score: 0.779
Mahalanobis Distance: 8.4
Mahalanobis Distance p-value: 0.807

Prediction: Positive if the Bayesian score is above the estimated best cutoff value from minimizing the false positive and false negative rate.
Probability: The esimated probability that the sample is in the positive category. This assumes that the Bayesian score follows a normal distribution and is different from the prediction using a cutoff.
Enrichment: An estimate of enrichment, that is, the increased likelihood (versus random) of this sample being in the category.
Bayesian Score: The standard Laplacian-modified Bayesian score.
Mahalanobis Distance: The Mahalanobis distance (MD) is the distance to the center of the training data. The larger the MD, the less trustworthy the prediction.
Mahalanobis Distance p-value: The p-value gives the fraction of training data with an MD greater than or equal to the one for the given sample, assuming normally distributed data. The smaller the p-value, the less trustworthy the prediciton. For highly non-normal X properties (e.g., fingerprints), the MD p-value is wildly inaccurate.

Structural Similar Compounds

Name	BENZOIN; OXIME	FLUORENE-9;9-(BIS)PROPYLAMINE	2;4-Dihydroxybenzophenone
Structure	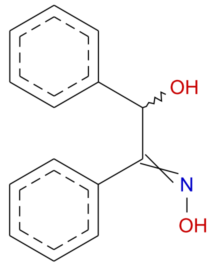	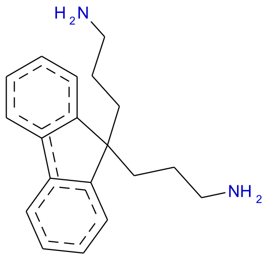	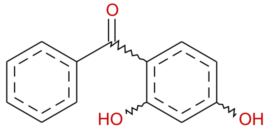
Actual Endpoint	Mild	Moderate_Severe	Mild
Predicted Endpoint	Mild	Moderate_Severe	Mild
Distance	0.535	0.554	0.572
Reference	28ZPAK-;111;72	IHFCAY 6;1;67	J. Am. Coll. Toxicol. 2(5):35;1983

Model Applicability

Unknown features are fingerprint features in the query molecule, but not found or appearing too infrequently in the training set.

1. All properties and OPS components are within expected ranges.

Feature Contribution

Top features for positive contribution

Fingerprint	Bit/Smiles	Feature Structure	Score	Moderate_Severe in training set
FCFP_10	1388176727	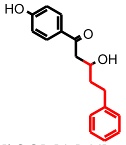 [*]C([*])CC[c]1:[cH]:[cH]:[cH]:[cH]:[cH]:1	0.389	19 out of 19

FCFP_10	-1066794953	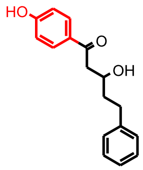 [*][c]1:[cH]:[cH]:[c](O):[cH]:[cH]:1	0.378	13 out of 13
FCFP_10	-497728148	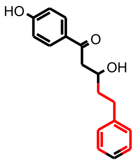 [*]CC[c]1:[cH]:[cH]:[*]:[cH]:[cH]:1	0.356	24 out of 25

Top Features for negative contribution

Fingerprint	Bit/Smiles	Feature Structure	Score	Moderate_Severe in training set
FCFP_10	-1426444049	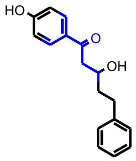 [*]C([*])CC(=O)[c](:[cH]:[*]):[cH]:[*]	-1.09	0 out of 3
FCFP_10	-463243689	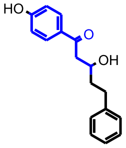 [*]C([*])CC(=O)[c]1:[cH]:[cH]:[c]([*]):[cH]:[cH]:1	-0.507	0 out of 1
FCFP_10	-1698724694	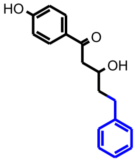 [*]C[c]1:[cH]:[cH]:[cH]:[cH]:[cH]:1	-0.284	53 out of 107

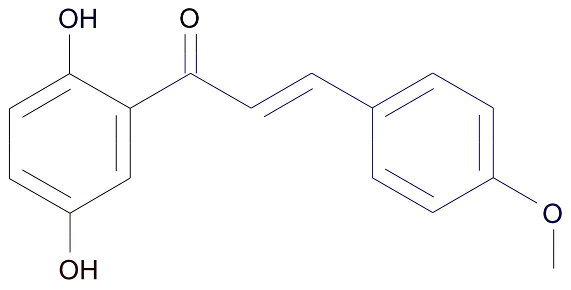

$C_{16}H_{14}O_4$
Molecular Weight: 270.28
ALogP: 3.201
Rotatable Bonds: 4
Acceptors: 4
Donors: 2

Model Prediction

Prediction: Mild
Probability: 0.722
Enrichment: 1.05
Bayesian Score: -3.1
Mahalanobis Distance: 7.59
Mahalanobis Distance p-value: 0.979

Prediction: Positive if the Bayesian score is above the estimated best cutoff value from minimizing the false positive and false negative rate.
Probability: The esimated probability that the sample is in the positive category. This assumes that the Bayesian score follows a normal distribution and is different from the prediction using a cutoff.
Enrichment: An estimate of enrichment, that is, the increased likelihood (versus random) of this sample being in the category.
Bayesian Score: The standard Laplacian-modified Bayesian score.
Mahalanobis Distance: The Mahalanobis distance (MD) is the distance to the center of the training data. The larger the MD, the less trustworthy the prediction.
Mahalanobis Distance p-value: The p-value gives the fraction of training data with an MD greater than or equal to the one for the given sample, assuming normally distributed data. The smaller the p-value, the less trustworthy the prediciton. For highly non-normal X properties (e.g., fingerprints), the MD p-value is wildly inaccurate.

Structural Similar Compounds

Name	2;4-Dihydroxybenzophenone	BENZOIN; OXIME	2-BIPHENYLCARBOXYLIC ACID; 2'-HYDROXYMETHYL-
Structure	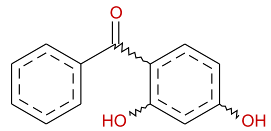	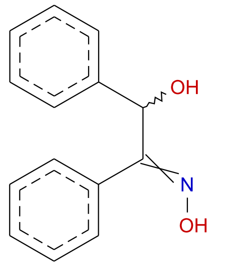	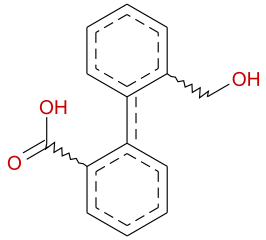
Actual Endpoint	Mild	Mild	Moderate_Severe
Predicted Endpoint	Mild	Mild	Mild
Distance	0.571	0.582	0.596
Reference	J. Am. Coll. Toxicol. 2(5):35;1983	28ZPAK-;111;72	IHFCAY 6;1;67

Model Applicability

Unknown features are fingerprint features in the query molecule, but not found or appearing too infrequently in the training set.

1. All properties and OPS components are within expected ranges.

Feature Contribution

Top features for positive contribution

Fingerprint	Bit/Smiles	Feature Structure	Score	Moderate_Severe in training set
FCFP_10	-158888774	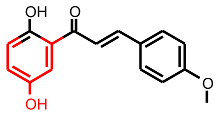 O[c]1:[cH]:[cH]:[*]:[cH]:[cH]:1	0.356	24 out of 25

FCFP_10	-1034142694	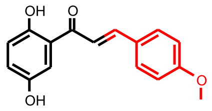 [*]C[c]1:[cH]:[cH]:[c](OC):[cH]:[c]:1[*]	0.338	18 out of 19
FCFP_10	7	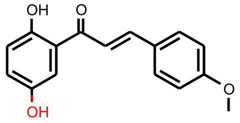 [*]O	0.219	117 out of 142

Top Features for negative contribution

Fingerprint	Bit/Smiles	Feature Structure	Score	Moderate_Severe in training set
FCFP_10	-305225196	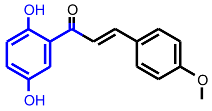 [*]C(=[*])[c]1:[cH]:[c](O):[cH]:[cH]:[c]: 1O	-0.842	0 out of 2
FCFP_10	-1977641857	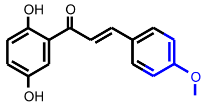 [*]:[cH]:[c](OC):[cH] :[*]	-0.78	4 out of 15
FCFP_10	2011169140	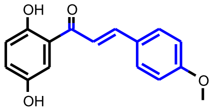 [*]C(=[*])\C=C\[c]1:[cH]:[cH]:[c]([*]):[c H]:[cH]:1	-0.507	0 out of 1

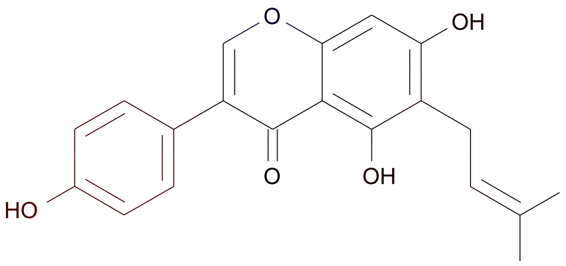

$C_{20}H_{18}O_5$
Molecular Weight: 338.354
ALogP: 3.997
Rotatable Bonds: 3
Acceptors: 5
Donors: 3

Model Prediction

Prediction: Moderate_Severe
Probability: 0.835
Enrichment: 1.21
Bayesian Score: 0.926
Mahalanobis Distance: 10.3
Mahalanobis Distance p-value: 0.0495

Prediction: Positive if the Bayesian score is above the estimated best cutoff value from minimizing the false positive and false negative rate.
Probability: The esimated probability that the sample is in the positive category. This assumes that the Bayesian score follows a normal distribution and is different from the prediction using a cutoff.
Enrichment: An estimate of enrichment, that is, the increased likelihood (versus random) of this sample being in the category.
Bayesian Score: The standard Laplacian-modified Bayesian score.
Mahalanobis Distance: The Mahalanobis distance (MD) is the distance to the center of the training data. The larger the MD, the less trustworthy the prediction.
Mahalanobis Distance p-value: The p-value gives the fraction of training data with an MD greater than or equal to the one for the given sample, assuming normally distributed data. The smaller the p-value, the less trustworthy the prediciton. For highly non-normal X properties (e.g., fingerprints), the MD p-value is wildly inaccurate.

Structural Similar Compounds

Name	ANTHRAQUINONE; 1-AMINO-4-HYDROXY-2-PHENOXY-	ANTHRAQUINONE; 1-((2-HYDROXYETHYL)AMINO)-4-(METHYLAMINO)-	METHANE;TRIS(4-AMINOPHENYL)-
Structure	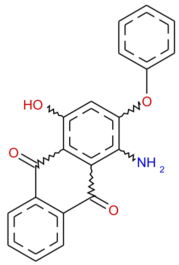	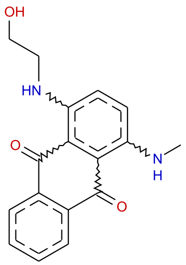	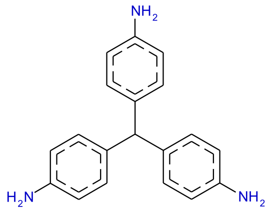
Actual Endpoint	Mild	Mild	Moderate_Severe
Predicted Endpoint	Mild	Mild	Moderate_Severe
Distance	0.691	0.699	0.708
Reference	28ZPAK 239;72	28ZPAK 245;72	28ZPAK-;73;72

Model Applicability

Unknown features are fingerprint features in the query molecule, but not found or appearing too infrequently in the training set.

1. All properties and OPS components are within expected ranges.

Feature Contribution

Top features for positive contribution				
**Fingerprint**	**Bit/Smiles**	**Feature Structure**	**Score**	**Moderate_Severe in training set**
FCFP_10	-1066794953	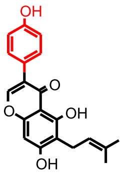 [*][c]1:[cH]:[cH]:[c](O):[cH]:[cH]:1	0.378	13 out of 13

FCFP_10	-158888774	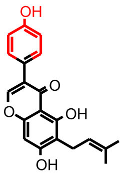<br>O[c]1:[cH]:[cH]:[*]:[cH]:[cH]:1	0.356	24 out of 25
FCFP_10	-1601875224	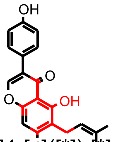<br>[*]C[c]1:[c]([*]):[*]:[c]([*]):[c](C(=[*])[*]):[c]:1O	0.352	7 out of 7
**Top Features for negative contribution**				
**Fingerprint**	**Bit/Smiles**	**Feature Structure**	**Score**	**Moderate_Severe in training set**
FCFP_10	-1099193755	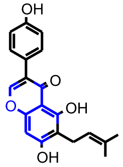<br>[*][C@@H]1[*]C[c]2:[cH]:[*]:[c]([*]):[cH]:[c]:2O1	-0.361	2 out of 5
FCFP_10	451877515	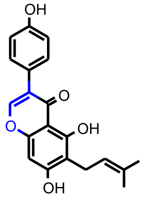<br>[*]OC=C([*])[*]	-0.35	16 out of 35
FCFP_10	-1604301295	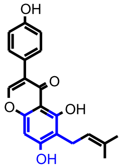<br>[*]C(=[*])[c]1:[cH]:[*]:[cH]:[cH]:[c]:1O	-0.164	15 out of 27

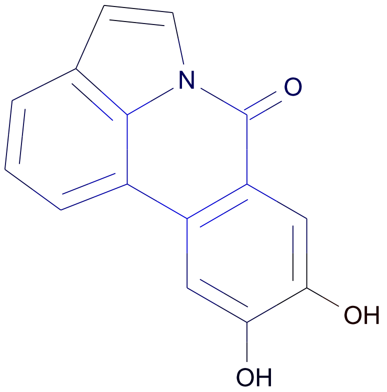

$C_{15}H_9NO_3$
Molecular Weight: 251.237
ALogP: 2.982
Rotatable Bonds: 0
Acceptors: 3
Donors: 2

Structural Similar Compounds

Name	2;4-Dihydroxybenzophenone	2-BIPHENYLCARBOXYLIC ACID; 2'-HYDROXYMETHYL-	PHENOL; 4;4'-THIODI-
Structure	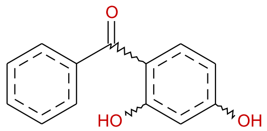	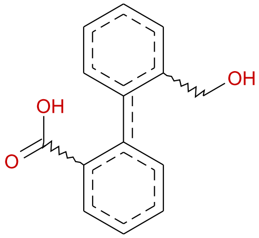	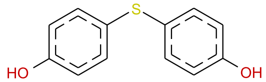
Actual Endpoint	Mild	Moderate_Severe	Moderate_Severe
Predicted Endpoint	Mild	Mild	Moderate_Severe
Distance	0.532	0.554	0.566
Reference	J. Am. Coll. Toxicol. 2(5):35;1983	IHFCAY 6;1;67	BIOFX* A408;71

Model Prediction

Prediction: Mild
Probability: 0.541
Enrichment: 0.785
Bayesian Score: -5.71
Mahalanobis Distance: 5.04
Mahalanobis Distance p-value: 1

Prediction: Positive if the Bayesian score is above the estimated best cutoff value from minimizing the false positive and false negative rate.
Probability: The esimated probability that the sample is in the positive category. This assumes that the Bayesian score follows a normal distribution and is different from the prediction using a cutoff.
Enrichment: An estimate of enrichment, that is, the increased likelihood (versus random) of this sample being in the category.
Bayesian Score: The standard Laplacian-modified Bayesian score.
Mahalanobis Distance: The Mahalanobis distance (MD) is the distance to the center of the training data. The larger the MD, the less trustworthy the prediction.
Mahalanobis Distance p-value: The p-value gives the fraction of training data with an MD greater than or equal to the one for the given sample, assuming normally distributed data. The smaller the p-value, the less trustworthy the prediciton. For highly non-normal X properties (e.g., fingerprints), the MD p-value is wildly inaccurate.

Model Applicability

Unknown features are fingerprint features in the query molecule, but not found or appearing too infrequently in the training set.

1. All properties and OPS components are within expected ranges.

Feature Contribution

Top features for positive contribution

Fingerprint	Bit/Smiles	Feature Structure	Score	Moderate_Severe in training set
FCFP_10	-124655670	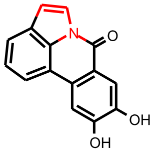 [*]n1:[*]:[*]:[cH]:[cH]:1	0.259	14 out of 16

FCFP_10	1804743636	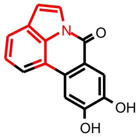 [*]n1:[cH]:[cH]:[c]2:[cH]:[cH]:[*]:[c]([*]):[c]:1:2	0.256	2 out of 2
FCFP_10	546102568	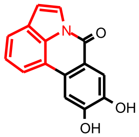 [*]n1:[cH]:[cH]:[c]2:[cH]:[cH]:[cH]:[c]([*]):[c]:1:2	0.256	2 out of 2

Top Features for negative contribution

Fingerprint	Bit/Smiles	Feature Structure	Score	Moderate_Severe in training set
FCFP_10	900733322	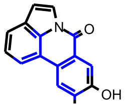 [*][c]1:[*]:[cH]:[c]2C(=[*])[*]:[c](:[*]):[c](:[cH]:[*])[c]:2:[cH]:1	-0.874	3 out of 13
FCFP_10	-306856457	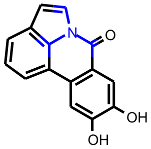 [*]C(=[*])n1:[cH]:[*]:[*]:[c]:1:[*]	-0.842	0 out of 2
FCFP_10	1900256554	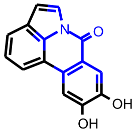 [*]:[cH]:[c]1C(=O)n2:[cH]:[*]:[*]:[c]:2:[*][c]:1:[*]	-0.842	0 out of 2

remdesivir

TOPKAT_Ocular_Irritancy_Mild_vs_Moderate_Severe

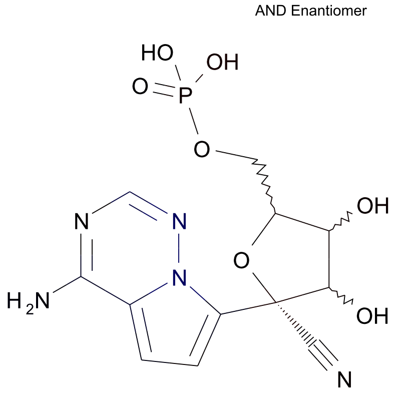

$C_{12}H_{14}N_5O_7P$

Molecular Weight: 371.24262

ALogP: -1.502

Rotatable Bonds: 4

Acceptors: 11

Donors: 5

Structural Similar Compounds

Name	1;3;6-NAPHTHALENE TRISULFONIC ACID;7-AMINO-	Methanol; (s-triazine-2;4;6-triyltrinitrilo)hexa-	2;2'-Biphenyldisulfonic acid; 4;4'-diamino-
Structure	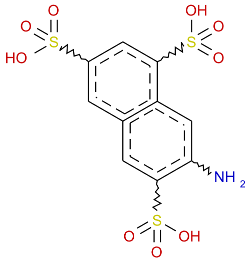	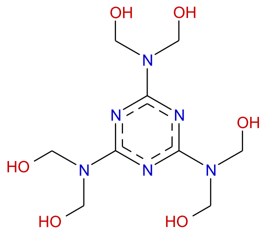	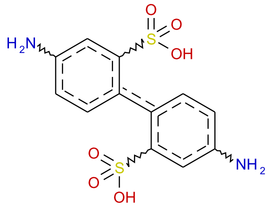
Actual Endpoint	Mild	Moderate_Severe	Mild
Predicted Endpoint	Mild	Moderate_Severe	Mild
Distance	0.776	0.802	0.878
Reference	28ZPAK-;190;72	Prehled Prumyslove Toxikologie; Organicke Latky; Marhold; J. -;876;86	Prehled Prumyslove Toxikologie; Organicke Latky; Marhold; J. pp 1061;86

Model Prediction

Prediction: Mild

Probability: 0.789

Enrichment: 1.15

Bayesian Score: -1.39

Mahalanobis Distance: 13.8

Mahalanobis Distance p-value: 1.42e-009

Prediction: Positive if the Bayesian score is above the estimated best cutoff value from minimizing the false positive and false negative rate.
Probability: The esimated probability that the sample is in the positive category. This assumes that the Bayesian score follows a normal distribution and is different from the prediction using a cutoff.
Enrichment: An estimate of enrichment, that is, the increased likelihood (versus random) of this sample being in the category.
Bayesian Score: The standard Laplacian-modified Bayesian score.
Mahalanobis Distance: The Mahalanobis distance (MD) is the distance to the center of the training data. The larger the MD, the less trustworthy the prediction.
Mahalanobis Distance p-value: The p-value gives the fraction of training data with an MD greater than or equal to the one for the given sample, assuming normally distributed data. The smaller the p-value, the less trustworthy the prediciton. For highly non-normal X properties (e.g., fingerprints), the MD p-value is wildly inaccurate.

Model Applicability

Unknown features are fingerprint features in the query molecule, but not found or appearing too infrequently in the training set.

1. OPS PC17 out of range. Value: 4.6782. Training min, max, SD, explained variance: -4.348, 3.9505, 1.094, 0.0146.
2. Unknown FCFP_2 feature: 472180098: [*]OP(=O)(O)O
3. Unknown FCFP_2 feature: -836603894: [*]C1[*][*]O[C@]1(C#[*])[c](:[*]):[*]
4. Unknown FCFP_2 feature: -124685461: [*]:n:[cH]:n:[*]
5. Unknown FCFP_2 feature: -1151884458: [*]:n:[c](N):[c](:[*]):[*]

Feature Contribution

Top features for positive contribution

Fingerprint	Bit/Smiles	Feature Structure	Score	Moderate_Severe in training set

FCFP_10	1070061035	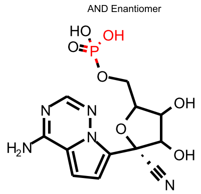 [*]C([*])O	0.239	284 out of 338
FCFP_10	-1539132615	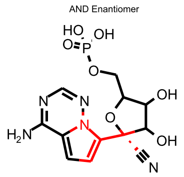 [*]C[c]1:n:[*]:[*]:[c]:1[*]	0.224	11 out of 13
FCFP_10	-1043250487	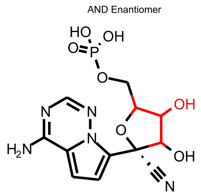 [*]CC(O)C[*]	0.22	62 out of 75

Top Features for negative contribution

Fingerprint	Bit/Smiles	Feature Structure	Score	Moderate_Severe in training set
FCFP_10	4427049	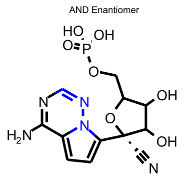 [*]:[cH]:n:n(:[*]):[*]	-1.29	0 out of 4
FCFP_10	-332197802	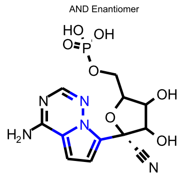 [*][c]1:[*]:[*]:[c](: [*]):n:1:n:[*]	-0.507	0 out of 1

FCFP_10	713358128	AND Enantiomer 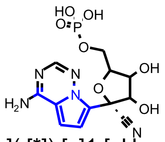 [*][c](:[*]):[c]1:[cH]:[cH]:[c]([*]):n:1:[*]	-0.307	8 out of 17

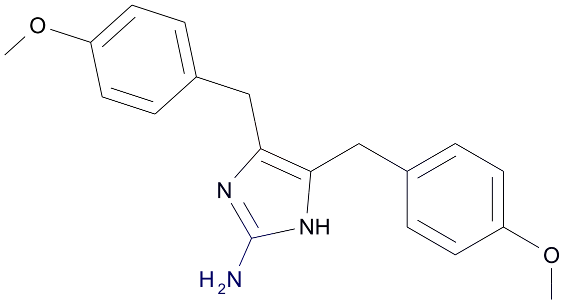

$C_{19}H_{21}N_3O_2$
Molecular Weight: 323.389
ALogP: 3.305
Rotatable Bonds: 6
Acceptors: 4
Donors: 2

Structural Similar Compounds

Name	5-NORBORNENE-2;3-DICARBOXYLIC ACID; 1;4;5;6;7;7-HEXACHLORO-	FLUORENE-9;9-(BIS)PROPYLAMINE	1;2-ETHANEDIOL;2;4-DICHLOROPHENOXY-
Structure	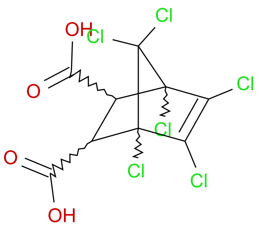	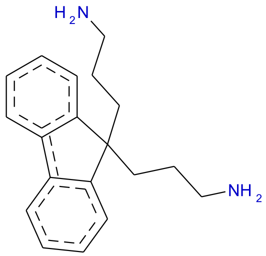	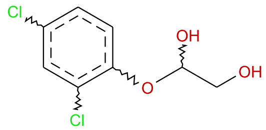
Actual Endpoint	Severe	Severe	Severe
Predicted Endpoint	Severe	Severe	Severe
Distance	0.674	0.680	0.693
Reference	28ZPAK-;92;72	IHFCAY 6;1;67	AMIHBC 4;119;51

Model Prediction

Prediction: Moderate
Probability: 0.585
Enrichment: 0.943
Bayesian Score: -2.57
Mahalanobis Distance: 7.1
Mahalanobis Distance p-value: 0.987

Prediction: Positive if the Bayesian score is above the estimated best cutoff value from minimizing the false positive and false negative rate.
Probability: The esimated probability that the sample is in the positive category. This assumes that the Bayesian score follows a normal distribution and is different from the prediction using a cutoff.
Enrichment: An estimate of enrichment, that is, the increased likelihood (versus random) of this sample being in the category.
Bayesian Score: The standard Laplacian-modified Bayesian score.
Mahalanobis Distance: The Mahalanobis distance (MD) is the distance to the center of the training data. The larger the MD, the less trustworthy the prediction.
Mahalanobis Distance p-value: The p-value gives the fraction of training data with an MD greater than or equal to the one for the given sample, assuming normally distributed data. The smaller the p-value, the less trustworthy the prediciton. For highly non-normal X properties (e.g., fingerprints), the MD p-value is wildly inaccurate.

Model Applicability

Unknown features are fingerprint features in the query molecule, but not found or appearing too infrequently in the training set.

1. All properties and OPS components are within expected ranges.

Feature Contribution

Top features for positive contribution

Fingerprint	Bit/Smiles	Feature Structure	Score	Severe in training set
SCFP_12	2109374332	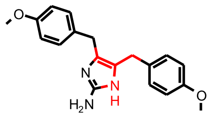 [*]C[c]1:[nH]:[*]:[*]:[c]:1[*]	0.311	7 out of 8

SCFP_12	772279865	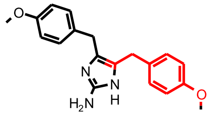 [*]O[c]1:[cH]:[cH]:[c](C[c](:[*]):[*]):[cH]:[cH]:1	0.218	1 out of 1
SCFP_12	1237755852	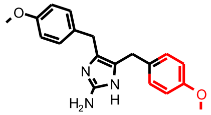 CO[c]1:[cH]:[cH]:[*]:[cH]:[cH]:1	0.126	16 out of 23
**Top Features for negative contribution**				
**Fingerprint**	**Bit/Smiles**	**Feature Structure**	**Score**	**Severe in training set**
SCFP_12	384920865	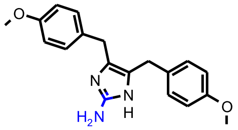 [*]:[c](:[*])N	-0.968	10 out of 46
SCFP_12	649648475	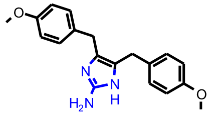 N[c]1:[nH]:[*]:[*]:n:1	-0.561	2 out of 7
SCFP_12	1287669168	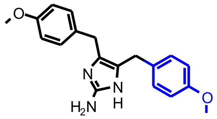 [*][c]1:[cH]:[cH]:[c](OC):[cH]:[cH]:1	-0.475	0 out of 1

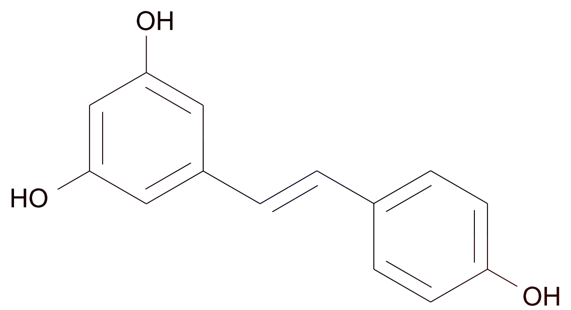

$C_{14}H_{12}O_3$
Molecular Weight: 228.243
ALogP: 3.09
Rotatable Bonds: 2
Acceptors: 3
Donors: 3

Structural Similar Compounds

Name	METHANE;TRIS(4-AMINOPHENYL)-	PHENOL; 4;4'-THIODI-	Benzimidazole; 6-amino-2-(4'-aminophenyl)-; dihydrochloride
Structure	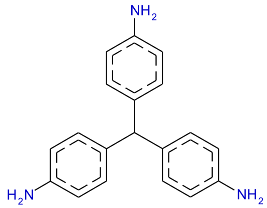	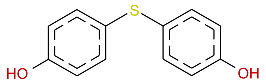	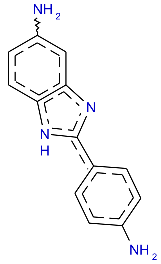
Actual Endpoint	Moderate	Severe	Moderate
Predicted Endpoint	Moderate	Severe	Moderate
Distance	0.593	0.665	0.678
Reference	28ZPAK-;73;72	BIOFX* A408;71	Prehled Prumyslove Toxikologie; Organicke Latky; Marhold; J. -;831;86

Model Prediction

Prediction: Severe
Probability: 0.684
Enrichment: 1.1
Bayesian Score: -0.204
Mahalanobis Distance: 7.22
Mahalanobis Distance p-value: 0.98

Prediction: Positive if the Bayesian score is above the estimated best cutoff value from minimizing the false positive and false negative rate.
Probability: The esimated probability that the sample is in the positive category. This assumes that the Bayesian score follows a normal distribution and is different from the prediction using a cutoff.
Enrichment: An estimate of enrichment, that is, the increased likelihood (versus random) of this sample being in the category.
Bayesian Score: The standard Laplacian-modified Bayesian score.
Mahalanobis Distance: The Mahalanobis distance (MD) is the distance to the center of the training data. The larger the MD, the less trustworthy the prediction.
Mahalanobis Distance p-value: The p-value gives the fraction of training data with an MD greater than or equal to the one for the given sample, assuming normally distributed data. The smaller the p-value, the less trustworthy the prediciton. For highly non-normal X properties (e.g., fingerprints), the MD p-value is wildly inaccurate.

Model Applicability

Unknown features are fingerprint features in the query molecule, but not found or appearing too infrequently in the training set.

1. All properties and OPS components are within expected ranges.

Feature Contribution

Top features for positive contribution

Fingerprint	Bit/Smiles	Feature Structure	Score	Severe in training set
SCFP_12	130348166	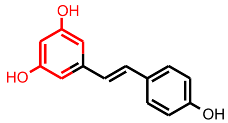 O[c]1:[cH]:[*]:[cH]:[c](O):[cH]:1	0.376	4 out of 4

SCFP_12	611156666	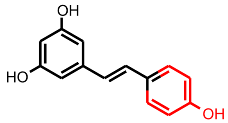<br>O[c]1:[cH]:[cH]:[*]:[cH]:[cH]:1	0.298	20 out of 24
SCFP_12	1112262477	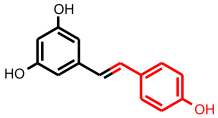<br>[*]=C[c]1:[cH]:[cH]:[c](O):[cH]:[cH]:1	0.218	1 out of 1

Top Features for negative contribution

Fingerprint	Bit/Smiles	Feature Structure	Score	Severe in training set
SCFP_12	-538866216	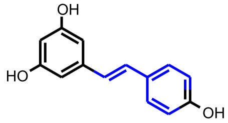<br>[*]\C=C\[c]1:[cH]:[cH]:[*]:[cH]:[cH]:1	-0.475	0 out of 1
SCFP_12	387787917	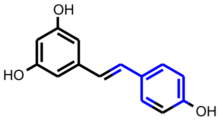<br>[*][c]1:[*]:[cH]:[c](C=[*]):[cH]:[cH]:1	-0.184	15 out of 30
SCFP_12	-2056718782	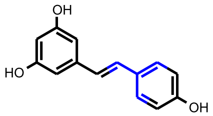<br>[*]=C[c](:[cH]:[*]):[cH]:[*]	-0.163	20 out of 39

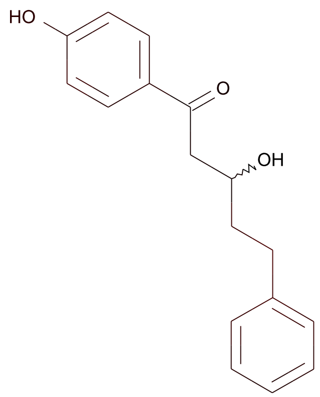

$C_{17}H_{18}O_3$
Molecular Weight: 270.323
ALogP: 3.293
Rotatable Bonds: 6
Acceptors: 3
Donors: 2

Structural Similar Compounds

Name	FLUORENE-9;9-(BIS)PROPYLAMINE	2-BIPHENYLCARBOXYLIC ACID; 2'-HYDROXYMETHYL-	PHENOL;4;4'-ISOPROPYLIDENEDI-
Structure	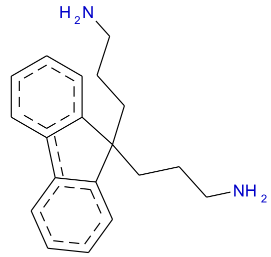	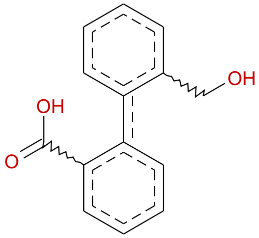	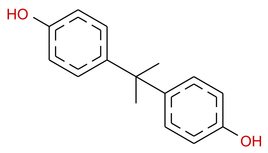
Actual Endpoint	Severe	Moderate	Moderate
Predicted Endpoint	Severe	Moderate	Moderate
Distance	0.573	0.573	0.601
Reference	IHFCAY 6;1;67	IHFCAY 6;1;67	28ZPAK-;58;72

Model Prediction

Prediction: Severe
Probability: 0.742
Enrichment: 1.2
Bayesian Score: 1.83
Mahalanobis Distance: 8.08
Mahalanobis Distance p-value: 0.797

Prediction: Positive if the Bayesian score is above the estimated best cutoff value from minimizing the false positive and false negative rate.
Probability: The esimated probability that the sample is in the positive category. This assumes that the Bayesian score follows a normal distribution and is different from the prediction using a cutoff.
Enrichment: An estimate of enrichment, that is, the increased likelihood (versus random) of this sample being in the category.
Bayesian Score: The standard Laplacian-modified Bayesian score.
Mahalanobis Distance: The Mahalanobis distance (MD) is the distance to the center of the training data. The larger the MD, the less trustworthy the prediction.
Mahalanobis Distance p-value: The p-value gives the fraction of training data with an MD greater than or equal to the one for the given sample, assuming normally distributed data. The smaller the p-value, the less trustworthy the prediciton. For highly non-normal X properties (e.g., fingerprints), the MD p-value is wildly inaccurate.

Model Applicability

Unknown features are fingerprint features in the query molecule, but not found or appearing too infrequently in the training set.

1. All properties and OPS components are within expected ranges.

Feature Contribution

Top features for positive contribution

Fingerprint	Bit/Smiles	Feature Structure	Score	Severe in training set
SCFP_12	-1640858361	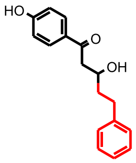 [*]CC[c]1:[cH]:[cH]:[cH]:[cH]:[cH]:1	0.376	4 out of 4

SCFP_12	-542118530	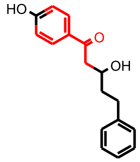 [*]CC(=O)[c]1:[cH]:[cH]:[*]:[cH]:[cH]:1	0.303	2 out of 2
SCFP_12	571854834	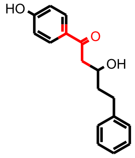 [*]CC(=O)[c](:[*]):[*]	0.303	2 out of 2

Top Features for negative contribution

Fingerprint	Bit/Smiles	Feature Structure	Score	Severe in training set
SCFP_12	387787917	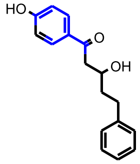 [*][c]1:[*]:[cH]:[c](C=[*]):[cH]:[cH]:1	-0.184	15 out of 30
SCFP_12	-2056718782	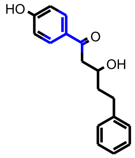 [*]=C[c](:[cH]:[*]):[cH]:[*]	-0.163	20 out of 39
SCFP_12	13	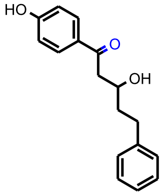 [*]=O	-0.105	185 out of 338

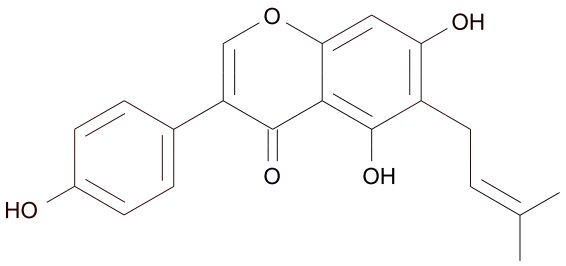

$C_{20}H_{18}O_5$

Molecular Weight: 338.354

ALogP: 3.997

Rotatable Bonds: 3

Acceptors: 5

Donors: 3

Model Prediction

Prediction: Severe

Probability: 0.707

Enrichment: 1.14

Bayesian Score: 0.481

Mahalanobis Distance: 8.71

Mahalanobis Distance p-value: 0.486

Prediction: Positive if the Bayesian score is above the estimated best cutoff value from minimizing the false positive and false negative rate.
Probability: The esimated probability that the sample is in the positive category. This assumes that the Bayesian score follows a normal distribution and is different from the prediction using a cutoff.
Enrichment: An estimate of enrichment, that is, the increased likelihood (versus random) of this sample being in the category.
Bayesian Score: The standard Laplacian-modified Bayesian score.
Mahalanobis Distance: The Mahalanobis distance (MD) is the distance to the center of the training data. The larger the MD, the less trustworthy the prediction.
Mahalanobis Distance p-value: The p-value gives the fraction of training data with an MD greater than or equal to the one for the given sample, assuming normally distributed data. The smaller the p-value, the less trustworthy the prediciton. For highly non-normal X properties (e.g., fingerprints), the MD p-value is wildly inaccurate.

Structural Similar Compounds

Name	METHANE;TRIS(4-AMINOPHENYL)-	5-NORBORNENE-2;3-DICARBOXYLIC ACID; 1;4;5;6;7;7-HEXACHLORO-	PHENOL;4-(3-CARBAZOLYLAMINO)-
Structure	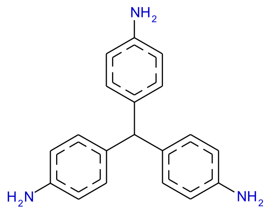	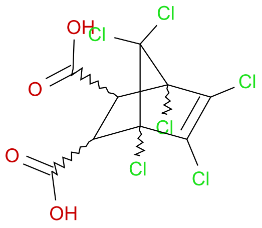	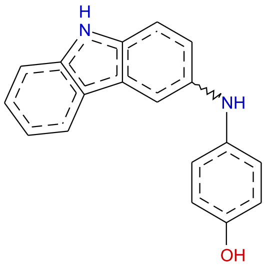
Actual Endpoint	Moderate	Severe	Moderate
Predicted Endpoint	Moderate	Severe	Moderate
Distance	0.715	0.788	0.791
Reference	28ZPAK-;73;72	28ZPAK-;92;72	28ZPAK-;143;72

Model Applicability

Unknown features are fingerprint features in the query molecule, but not found or appearing too infrequently in the training set.

1. All properties and OPS components are within expected ranges.

Feature Contribution

Top features for positive contribution				
Fingerprint	Bit/Smiles	Feature Structure	Score	Severe in training set
SCFP_12	130348166	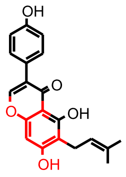 O[c]1:[cH]:[*]:[cH]:[c](O):[cH]:1	0.376	4 out of 4

SCFP_12	611156666	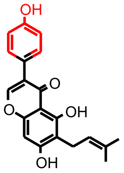 O[c]1:[cH]:[cH]:[*]:[cH]:[cH]:1	0.298	20 out of 24
SCFP_12	470101049	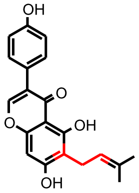 [*]=CC[c](:[*]):[*]	0.218	1 out of 1
**Top Features for negative contribution**				
**Fingerprint**	**Bit/Smiles**	**Feature Structure**	**Score**	**Severe in training set**
SCFP_12	-1980302127	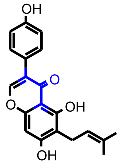 [*]C(=[*])C(=O)[c](:[*]):[*]	-0.345	1 out of 3
SCFP_12	387787917	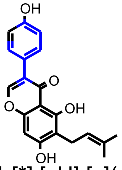 [*][c]1:[*]:[cH]:[c](C=[*]):[cH]:[cH]:1	-0.184	15 out of 30
SCFP_12	-2056718782	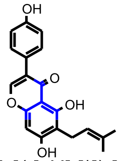 [*]=C[c](:[cH]:[*]):[cH]:[*]	-0.163	20 out of 39

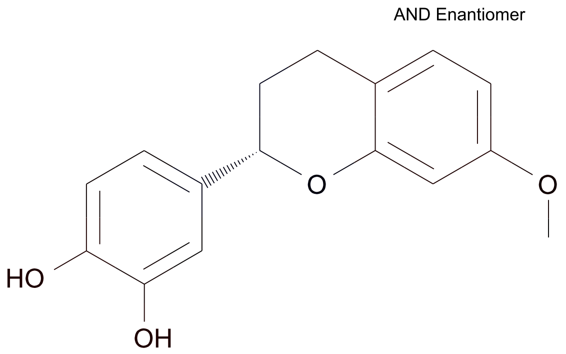

$C_{16}H_{16}O_4$
Molecular Weight: 272.296
ALogP: 3.456
Rotatable Bonds: 2
Acceptors: 4
Donors: 2

Model Prediction

Prediction: Irritant
Probability: 1
Enrichment: 1.18
Bayesian Score: 0.607
Mahalanobis Distance: 7.02
Mahalanobis Distance p-value: 0.998

Prediction: Positive if the Bayesian score is above the estimated best cutoff value from minimizing the false positive and false negative rate.
Probability: The esimated probability that the sample is in the positive category. This assumes that the Bayesian score follows a normal distribution and is different from the prediction using a cutoff.
Enrichment: An estimate of enrichment, that is, the increased likelihood (versus random) of this sample being in the category.
Bayesian Score: The standard Laplacian-modified Bayesian score.
Mahalanobis Distance: The Mahalanobis distance (MD) is the distance to the center of the training data. The larger the MD, the less trustworthy the prediction.
Mahalanobis Distance p-value: The p-value gives the fraction of training data with an MD greater than or equal to the one for the given sample, assuming normally distributed data. The smaller the p-value, the less trustworthy the prediciton. For highly non-normal X properties (e.g., fingerprints), the MD p-value is wildly inaccurate.

Structural Similar Compounds

Name	2;2'-Dihydroxy-4-methoxybenzophenone	2;2';-Dihydroxy-4;4'-dimethoxybenzophenone	BENZOIN; OXIME
Structure	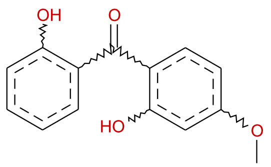	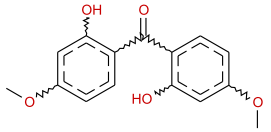	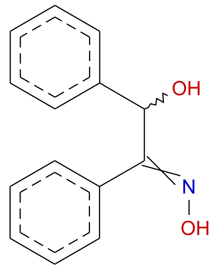
Actual Endpoint	Non-Irritant	Non-Irritant	Irritant
Predicted Endpoint	Non-Irritant	Non-Irritant	Irritant
Distance	0.506	0.540	0.596
Reference	J. Am. Coll. Toxicol. 2(5):35;1983	J. Am. Coll. Toxicol. 2(5):35;1983	28ZPAK-;111;72

Model Applicability

Unknown features are fingerprint features in the query molecule, but not found or appearing too infrequently in the training set.

1. All properties and OPS components are within expected ranges.

Feature Contribution

Top features for positive contribution				
**Fingerprint**	**Bit/Smiles**	**Feature Structure**	**Score**	**Irritant in training set**
FCFP_12	-204034463	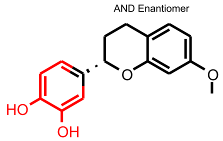 O[c]1:[cH]:[*]:[cH]:[cH]:[c]:1O	0.175	5 out of 5

FCFP_12	-1099193755	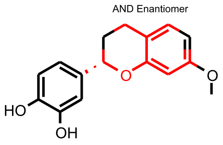<br>[*][C@@H]1[*]C[c]2:[c<br>H]:[*]:[c]([*]):[cH]<br>:[c]:2O1	0.175	5 out of 5
FCFP_12	666068305	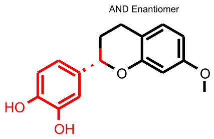<br>[*]C([*])[c]1:[cH]:[c<br>H]:[c](O):[c](O):[cH<br>]:1	0.137	2 out of 2

Top Features for negative contribution

**Fingerprint**	**Bit/Smiles**	**Feature Structure**	**Score**	**Irritant in training set**
FCFP_12	1186333723	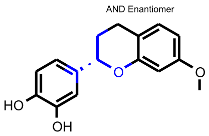<br>[*]C[C@H](O[*])[c](:[<br>*]):[*]	-0.344	2 out of 4
FCFP_12	1916525245	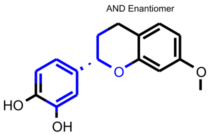<br>[*]C[C@H](O[*])[c]1:[<br>cH]:[cH]:[*]:[c]([*]<br>):[cH]:1	-0.344	2 out of 4
FCFP_12	-1034142694	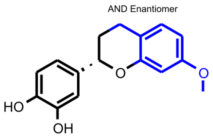<br>[*]C[c]1:[cH]:[cH]:[c<br>](OC):[cH]:[c]:1[*]	-0.0579	19 out of 25

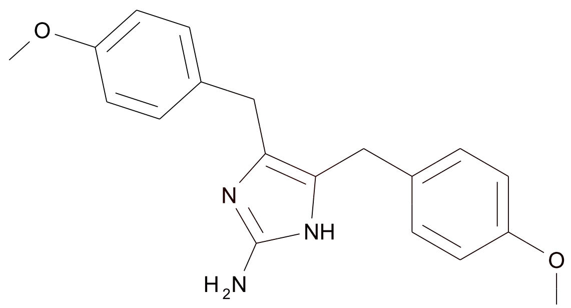

$C_{19}H_{21}N_3O_2$
Molecular Weight: 323.389
ALogP: 3.305
Rotatable Bonds: 6
Acceptors: 4
Donors: 2

Structural Similar Compounds

Name	2;2';-Dihydroxy-4;4'-dimethoxybenzophenone	2;2'-Dihydroxy-4-methoxybenzophenone	1-AMINO-4-BENZOYLAMINO-ANTHRAQUINONE
Structure	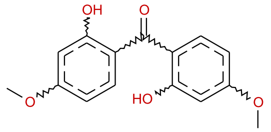	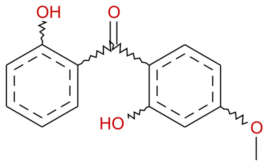	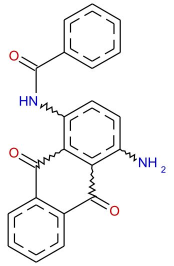
Actual Endpoint	Non-Irritant	Non-Irritant	Irritant
Predicted Endpoint	Non-Irritant	Non-Irritant	Irritant
Distance	0.591	0.612	0.614
Reference	J. Am. Coll. Toxicol. 2(5):35;1983	J. Am. Coll. Toxicol. 2(5):35;1983	28ZPAK-;124;72

Model Prediction

Prediction: Irritant
Probability: 1
Enrichment: 1.18
Bayesian Score: 1.21
Mahalanobis Distance: 6.33
Mahalanobis Distance p-value: 1

Prediction: Positive if the Bayesian score is above the estimated best cutoff value from minimizing the false positive and false negative rate.
Probability: The esimated probability that the sample is in the positive category. This assumes that the Bayesian score follows a normal distribution and is different from the prediction using a cutoff.
Enrichment: An estimate of enrichment, that is, the increased likelihood (versus random) of this sample being in the category.
Bayesian Score: The standard Laplacian-modified Bayesian score.
Mahalanobis Distance: The Mahalanobis distance (MD) is the distance to the center of the training data. The larger the MD, the less trustworthy the prediction.
Mahalanobis Distance p-value: The p-value gives the fraction of training data with an MD greater than or equal to the one for the given sample, assuming normally distributed data. The smaller the p-value, the less trustworthy the prediciton. For highly non-normal X properties (e.g., fingerprints), the MD p-value is wildly inaccurate.

Model Applicability

Unknown features are fingerprint features in the query molecule, but not found or appearing too infrequently in the training set.

1. All properties and OPS components are within expected ranges.
2. Unknown FCFP_2 feature: 203707511: [*]C[c]1:[nH]:[*]:[*]:[c]:1[*]
3. Unknown FCFP_2 feature: -1151854667: N[c]1:[nH]:[*]:[*]:n:1

Feature Contribution

Top features for positive contribution

Fingerprint	Bit/Smiles	Feature Structure	Score	Irritant in training set
FCFP_12	1747237384	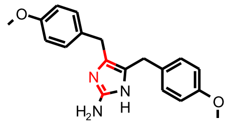 [*][c]1:[*]:[*]:[c]([*]):n:1	0.208	44 out of 44

FCFP_12	-1539132615	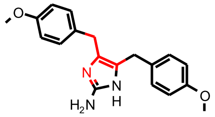<br>[*]C[c]1:n:[*]:[*]:[c<br>]:1[*]	0.197	13 out of 13
FCFP_12	17	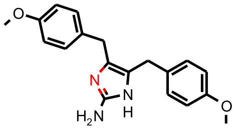<br>[*]:n:[*]	0.189	48 out of 49
**Top Features for negative contribution**				
**Fingerprint**	**Bit/Smiles**	**Feature Structure**	**Score**	**Irritant in training set**
FCFP_12	-1034142694	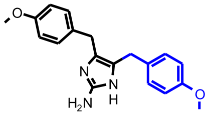<br>[*]C[c]1:[cH]:[cH]:[c<br>](OC):[cH]:[c]:1[*]	-0.0579	19 out of 25
FCFP_12	136627117	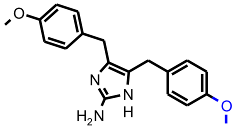<br>[*]OC	0	96 out of 113
FCFP_12	203677720	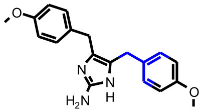<br>[*]C[c](:[cH]:[*]):[c<br>]([*]):[*]	0	319 out of 382

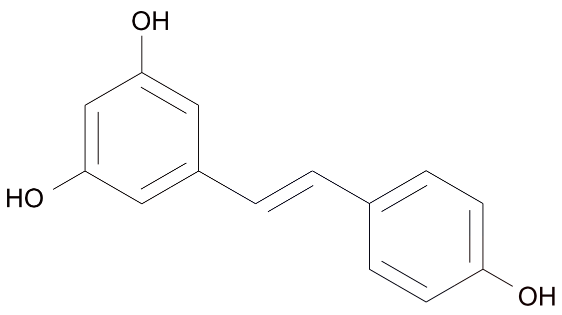

$C_{14}H_{12}O_3$
Molecular Weight: 228.243
ALogP: 3.09
Rotatable Bonds: 2
Acceptors: 3
Donors: 3

Model Prediction

Prediction: Irritant
Probability: 0.976
Enrichment: 1.15
Bayesian Score: 0.203
Mahalanobis Distance: 5.64
Mahalanobis Distance p-value: 1

Prediction: Positive if the Bayesian score is above the estimated best cutoff value from minimizing the false positive and false negative rate.
Probability: The esimated probability that the sample is in the positive category. This assumes that the Bayesian score follows a normal distribution and is different from the prediction using a cutoff.
Enrichment: An estimate of enrichment, that is, the increased likelihood (versus random) of this sample being in the category.
Bayesian Score: The standard Laplacian-modified Bayesian score.
Mahalanobis Distance: The Mahalanobis distance (MD) is the distance to the center of the training data. The larger the MD, the less trustworthy the prediction.
Mahalanobis Distance p-value: The p-value gives the fraction of training data with an MD greater than or equal to the one for the given sample, assuming normally distributed data. The smaller the p-value, the less trustworthy the prediciton. For highly non-normal X properties (e.g., fingerprints), the MD p-value is wildly inaccurate.

Structural Similar Compounds

Name	METHANE;TRIS(4-AMINOPHENYL)-	2;4-Dihydroxybenzophenone	4;4'METHYLENE DIPHENOL
Structure	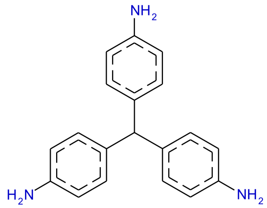	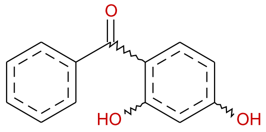	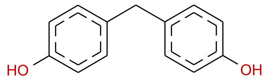
Actual Endpoint	Irritant	Irritant	Irritant
Predicted Endpoint	Irritant	Non-Irritant	Irritant
Distance	0.557	0.623	0.631
Reference	28ZPAK-;73;72	J. Am. Coll. Toxicol. 2(5):35;1983	AIHAAP 23;95;62

Model Applicability

Unknown features are fingerprint features in the query molecule, but not found or appearing too infrequently in the training set.

1. All properties and OPS components are within expected ranges.

Feature Contribution

Top features for positive contribution

Fingerprint	Bit/Smiles	Feature Structure	Score	Irritant in training set
FCFP_12	946068634	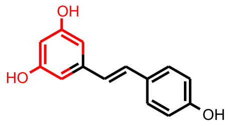 O[c]1:[cH]:[*]:[cH]:[c](O):[cH]:1	0.156	3 out of 3

FCFP_12	-1748394506	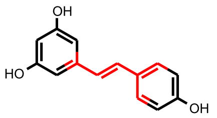<br>[*]:[cH]:[c](\C=C\[c]<br>(:[*]):[*]):[cH]:[*]	0.137	2 out of 2
FCFP_12	7	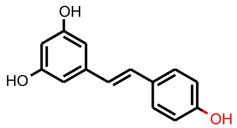<br>[*]O	0.119	142 out of 156

Top Features for negative contribution

Fingerprint	Bit/Smiles	Feature Structure	Score	Irritant in training set
FCFP_12	-1078052987	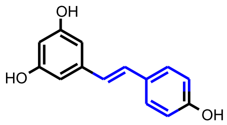<br>[*]\C=C\[c]1:[cH]:[cH<br>]:[*]:[cH]:[cH]:1	-0.344	2 out of 4
FCFP_12	451371068	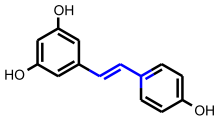<br>[*]\C=C\[c](:[*]):[*]	-0.167	6 out of 9
FCFP_12	-1066794953	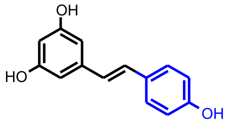<br>[*][c]1:[cH]:[cH]:[c]<br>(O):[cH]:[cH]:1	-0.0509	13 out of 17

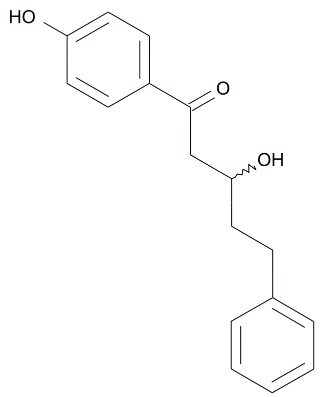

$C_{17}H_{18}O_3$
Molecular Weight: 270.323
ALogP: 3.293
Rotatable Bonds: 6
Acceptors: 3
Donors: 2

Model Prediction

Prediction: Irritant
Probability: 1
Enrichment: 1.18
Bayesian Score: 0.777
Mahalanobis Distance: 6.79
Mahalanobis Distance p-value: 0.999

Prediction: Positive if the Bayesian score is above the estimated best cutoff value from minimizing the false positive and false negative rate.
Probability: The esimated probability that the sample is in the positive category. This assumes that the Bayesian score follows a normal distribution and is different from the prediction using a cutoff.
Enrichment: An estimate of enrichment, that is, the increased likelihood (versus random) of this sample being in the category.
Bayesian Score: The standard Laplacian-modified Bayesian score.
Mahalanobis Distance: The Mahalanobis distance (MD) is the distance to the center of the training data. The larger the MD, the less trustworthy the prediction.
Mahalanobis Distance p-value: The p-value gives the fraction of training data with an MD greater than or equal to the one for the given sample, assuming normally distributed data. The smaller the p-value, the less trustworthy the prediciton. For highly non-normal X properties (e.g., fingerprints), the MD p-value is wildly inaccurate.

Structural Similar Compounds

Name	BENZOIN; OXIME	FLUORENE-9;9-(BIS)PROPYLAMINE	2-BIPHENYLCARBOXYLIC ACID; 2'-HYDROXYMETHYL-
Structure	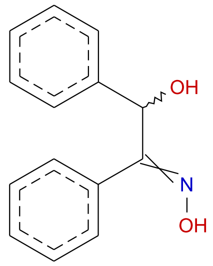	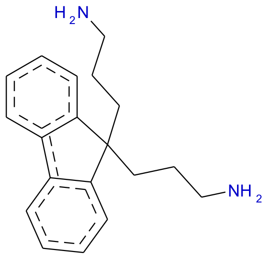	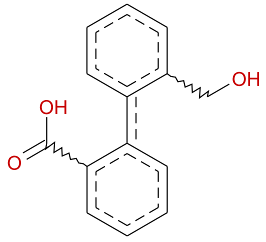
Actual Endpoint	Irritant	Irritant	Irritant
Predicted Endpoint	Irritant	Irritant	Irritant
Distance	0.529	0.552	0.567
Reference	28ZPAK-;111;72	IHFCAY 6;1;67	IHFCAY 6;1;67

Model Applicability

Unknown features are fingerprint features in the query molecule, but not found or appearing too infrequently in the training set.

1. All properties and OPS components are within expected ranges.

Feature Contribution

Top features for positive contribution				
Fingerprint	Bit/Smiles	Feature Structure	Score	Irritant in training set
FCFP_12	-1426444049	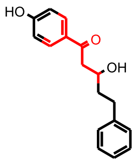 [*]C([*])CC(=O)[c](:[cH]:[*]):[cH]:[*]	0.156	3 out of 3

FCFP_12	-1442566880	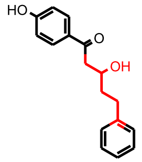 [*]CC(O)CC[c](:[*]):[*]	0.137	2 out of 2
FCFP_12	7	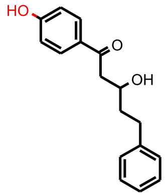 [*]O	0.119	142 out of 156
**Top Features for negative contribution**				
**Fingerprint**	**Bit/Smiles**	**Feature Structure**	**Score**	**Irritant in training set**
FCFP_12	1242035682	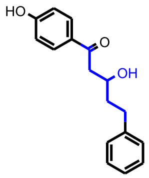 [*]CCC(O)CC(=[*])[*]	-0.212	7 out of 11
FCFP_12	1981711554	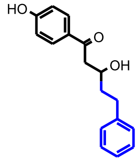 [*]CC[c]1:[cH]:[cH]:[cH]:[cH]:[cH]:1	-0.103	5 out of 7
FCFP_12	-1698724694	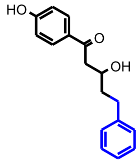 [*]C[c]1:[cH]:[cH]:[cH]:[cH]:[cH]:1	-0.0964	107 out of 146

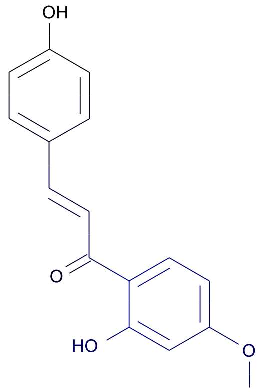

$C_{16}H_{14}O_4$
Molecular Weight: 270.28
ALogP: 3.201
Rotatable Bonds: 4
Acceptors: 4
Donors: 2

Model Prediction

Prediction: Non-Irritant
Probability: 0.932
Enrichment: 1.1
Bayesian Score: -3.15
Mahalanobis Distance: 6.75
Mahalanobis Distance p-value: 1

Prediction: Positive if the Bayesian score is above the estimated best cutoff value from minimizing the false positive and false negative rate.
Probability: The esimated probability that the sample is in the positive category. This assumes that the Bayesian score follows a normal distribution and is different from the prediction using a cutoff.
Enrichment: An estimate of enrichment, that is, the increased likelihood (versus random) of this sample being in the category.
Bayesian Score: The standard Laplacian-modified Bayesian score.
Mahalanobis Distance: The Mahalanobis distance (MD) is the distance to the center of the training data. The larger the MD, the less trustworthy the prediction.
Mahalanobis Distance p-value: The p-value gives the fraction of training data with an MD greater than or equal to the one for the given sample, assuming normally distributed data. The smaller the p-value, the less trustworthy the prediciton. For highly non-normal X properties (e.g., fingerprints), the MD p-value is wildly inaccurate.

Structural Similar Compounds

Name	2;2'-Dihydroxy-4-methoxybenzophenone	2;2';-Dihydroxy-4;4'-dimethoxybenzophenone	2;4-Dihydroxybenzophenone
Structure	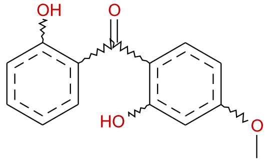	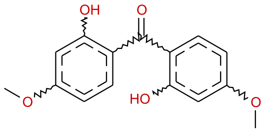	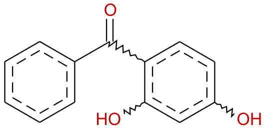
Actual Endpoint	Non-Irritant	Non-Irritant	Irritant
Predicted Endpoint	Non-Irritant	Non-Irritant	Non-Irritant
Distance	0.434	0.464	0.561
Reference	J. Am. Coll. Toxicol. 2(5):35;1983	J. Am. Coll. Toxicol. 2(5):35;1983	J. Am. Coll. Toxicol. 2(5):35;1983

Model Applicability

Unknown features are fingerprint features in the query molecule, but not found or appearing too infrequently in the training set.

1. All properties and OPS components are within expected ranges.

Feature Contribution

Top features for positive contribution				
**Fingerprint**	**Bit/Smiles**	**Feature Structure**	**Score**	**Irritant in training set**
FCFP_12	7	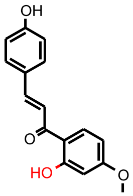 [*]O	0.119	142 out of 156

FCFP_12	2011169140	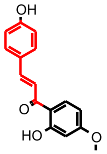 [*]C(=[*])\C=C\[c]1:[cH]:[cH]:[c]([*]):[cH]:[cH]:1	0.101	1 out of 1
FCFP_12	1036089772	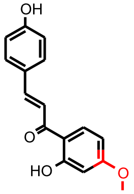 [*]:[c](:[*])OC	0.0988	75 out of 84

Top Features for negative contribution

Fingerprint	Bit/Smiles	Feature Structure	Score	Irritant in training set
FCFP_12	1800760657	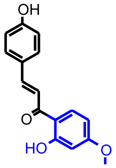 [*][c]1:[cH]:[cH]:[c](OC):[cH]:[c]:1O	-1.23	0 out of 3
FCFP_12	1863797348	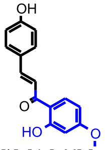 [*]C(=[*])[c]1:[cH]:[cH]:[c](OC):[cH]:[c]:1O	-0.749	1 out of 4
FCFP_12	1244036906	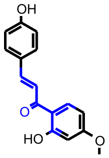 [*]\C=C\C(=O)[c](:[cH]:[*]):[c]([*]):[*]	-0.592	0 out of 1

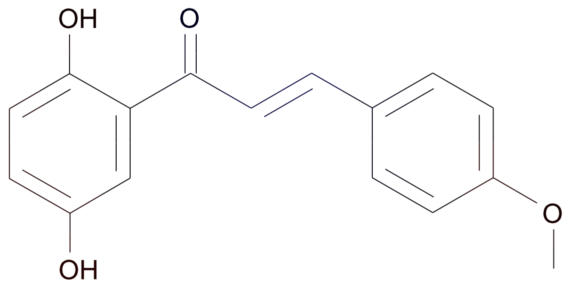

$C_{16}H_{14}O_4$
Molecular Weight: 270.28
ALogP: 3.201
Rotatable Bonds: 4
Acceptors: 4
Donors: 2

Model Prediction

Prediction: Irritant
Probability: 0.975
Enrichment: 1.15
Bayesian Score: -0.309
Mahalanobis Distance: 6.75
Mahalanobis Distance p-value: 1

Prediction: Positive if the Bayesian score is above the estimated best cutoff value from minimizing the false positive and false negative rate.
Probability: The esimated probability that the sample is in the positive category. This assumes that the Bayesian score follows a normal distribution and is different from the prediction using a cutoff.
Enrichment: An estimate of enrichment, that is, the increased likelihood (versus random) of this sample being in the category.
Bayesian Score: The standard Laplacian-modified Bayesian score.
Mahalanobis Distance: The Mahalanobis distance (MD) is the distance to the center of the training data. The larger the MD, the less trustworthy the prediction.
Mahalanobis Distance p-value: The p-value gives the fraction of training data with an MD greater than or equal to the one for the given sample, assuming normally distributed data. The smaller the p-value, the less trustworthy the prediciton. For highly non-normal X properties (e.g., fingerprints), the MD p-value is wildly inaccurate.

Structural Similar Compounds

Name	2;2'-Dihydroxy-4-methoxybenzophenone	2;2';-Dihydroxy-4;4'-dimethoxybenzophenone	2;4-Dihydroxybenzophenone
Structure	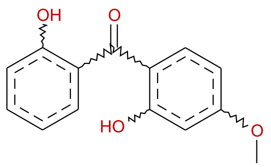	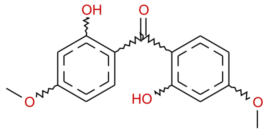	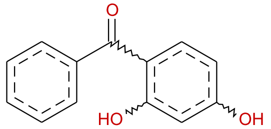
Actual Endpoint	Non-Irritant	Non-Irritant	Irritant
Predicted Endpoint	Non-Irritant	Non-Irritant	Non-Irritant
Distance	0.455	0.486	0.566
Reference	J. Am. Coll. Toxicol. 2(5):35;1983	J. Am. Coll. Toxicol. 2(5):35;1983	J. Am. Coll. Toxicol. 2(5):35;1983

Model Applicability

Unknown features are fingerprint features in the query molecule, but not found or appearing too infrequently in the training set.

1. All properties and OPS components are within expected ranges.

Feature Contribution

Top features for positive contribution				
**Fingerprint**	**Bit/Smiles**	**Feature Structure**	**Score**	**Irritant in training set**
FCFP_12	-9847677	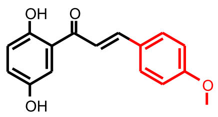 [*][c]1:[cH]:[cH]:[c](OC):[cH]:[cH]:1	0.156	3 out of 3

FCFP_12	-305225196	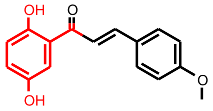 [*]C(=[*])[c]1:[cH]:[c](O):[cH]:[cH]:[c]: 1O	0.137	2 out of 2
FCFP_12	7	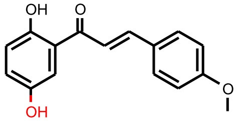 [*]O	0.119	142 out of 156

Top Features for negative contribution

Fingerprint	Bit/Smiles	Feature Structure	Score	Irritant in training set
FCFP_12	1244036906	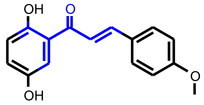 [*]\C=C\C(=O)[c](:[cH]:[*]):[c]([*]):[*]	-0.592	0 out of 1
FCFP_12	-1078052987	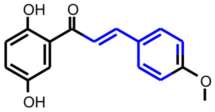 [*]\C=C\[c]1:[cH]:[cH]:[*]:[cH]:[cH]:1	-0.344	2 out of 4
FCFP_12	-146015125	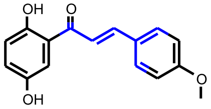 [*]C(=[*])\C=C\[c](:[cH]:[*]):[cH]:[*]	-0.268	1 out of 2

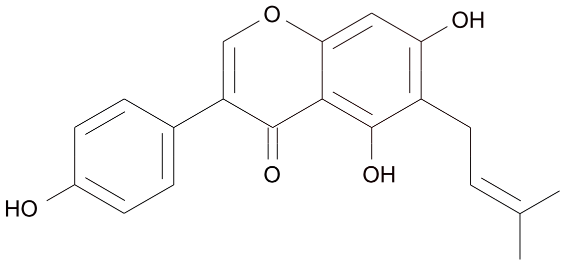

$C_{20}H_{18}O_5$
Molecular Weight: 338.354
ALogP: 3.997
Rotatable Bonds: 3
Acceptors: 5
Donors: 3

Structural Similar Compounds

Name	ANTHRAQUINONE; 1-((2-HYDROXYETHYL)AMINO)-4-(METHYLAMINO)-	Disperse Black 9	ANTHRAQUINONE; 1-AMINO-4-HYDROXY-2-PHENOXY-
Structure	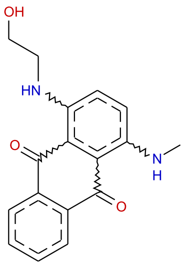	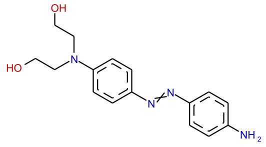	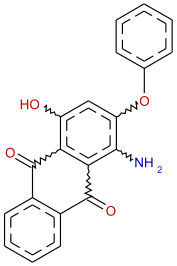
Actual Endpoint	Irritant	Non-Irritant	Irritant
Predicted Endpoint	Irritant	Non-Irritant	Irritant
Distance	0.673	0.684	0.692
Reference	28ZPAK 245;72	J. Am. Coll. Toxicol. 5(3):205;1986	28ZPAK 239;72

Model Prediction

Prediction: Irritant
Probability: 1
Enrichment: 1.18
Bayesian Score: 0.647
Mahalanobis Distance: 11.5
Mahalanobis Distance p-value: 0.000737

Prediction: Positive if the Bayesian score is above the estimated best cutoff value from minimizing the false positive and false negative rate.
Probability: The esimated probability that the sample is in the positive category. This assumes that the Bayesian score follows a normal distribution and is different from the prediction using a cutoff.
Enrichment: An estimate of enrichment, that is, the increased likelihood (versus random) of this sample being in the category.
Bayesian Score: The standard Laplacian-modified Bayesian score.
Mahalanobis Distance: The Mahalanobis distance (MD) is the distance to the center of the training data. The larger the MD, the less trustworthy the prediction.
Mahalanobis Distance p-value: The p-value gives the fraction of training data with an MD greater than or equal to the one for the given sample, assuming normally distributed data. The smaller the p-value, the less trustworthy the prediciton. For highly non-normal X properties (e.g., fingerprints), the MD p-value is wildly inaccurate.

Model Applicability

Unknown features are fingerprint features in the query molecule, but not found or appearing too infrequently in the training set.

1. All properties and OPS components are within expected ranges.

Feature Contribution

Top features for positive contribution

Fingerprint	Bit/Smiles	Feature Structure	Score	Irritant in training set
FCFP_12	-1601875224	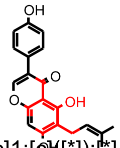 [*]C[c]1:[c]([*]):[*]:[c]([*]):[c](C(=[*])[*]):[c]:1O	0.184	7 out of 7

FCFP_12	-1099193755	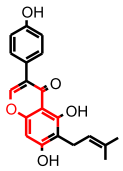<br>[*][C@@H]1[*]C[c]2:[cH]:[*]:[c]([*]):[cH]:[c]:2O1	0.175	5 out of 5
FCFP_12	7	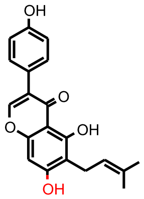<br>[*]O	0.119	142 out of 156
**Top Features for negative contribution**				
**Fingerprint**	**Bit/Smiles**	**Feature Structure**	**Score**	**Irritant in training set**
FCFP_12	1673930087	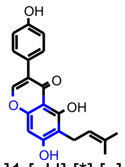<br>[*]O[c]1:[cH]:[*]:[c]([*]):[c](O):[cH]:1	-0.218	5 out of 8
FCFP_12	-628297815	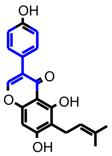<br>[*]\C=C(/C(=[*])[*])\[c]1:[cH]:[cH]:[*]:[cH]:[cH]:1	-0.132	2 out of 3
FCFP_12	-1582522951	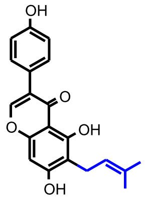<br>[*]CC=C(C)C	-0.0561	3 out of 4

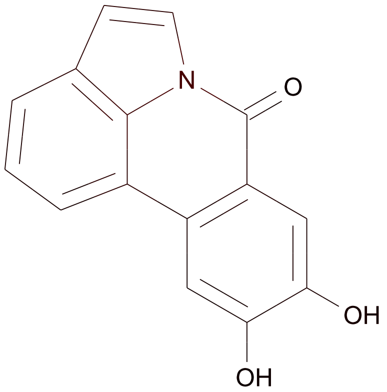

$C_{15}H_9NO_3$
Molecular Weight: 251.237
ALogP: 2.982
Rotatable Bonds: 0
Acceptors: 3
Donors: 2

Structural Similar Compounds

Name	2;4-Dihydroxybenzophenone	2-BIPHENYLCARBOXYLIC ACID; 2'-HYDROXYMETHYL-	PHENOL; 4;4'-THIODI-
Structure	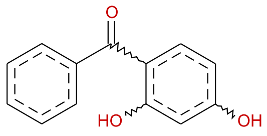	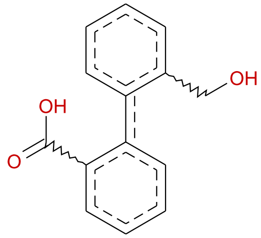	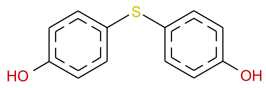
Actual Endpoint	Irritant	Irritant	Irritant
Predicted Endpoint	Non-Irritant	Irritant	Irritant
Distance	0.526	0.544	0.560
Reference	J. Am. Coll. Toxicol. 2(5):35;1983	IHFCAY 6;1;67	BIOFX* A408;71

Model Prediction

Prediction: Irritant
Probability: 1
Enrichment: 1.18
Bayesian Score: 2.34
Mahalanobis Distance: 3.92
Mahalanobis Distance p-value: 1

Prediction: Positive if the Bayesian score is above the estimated best cutoff value from minimizing the false positive and false negative rate.
Probability: The esimated probability that the sample is in the positive category. This assumes that the Bayesian score follows a normal distribution and is different from the prediction using a cutoff.
Enrichment: An estimate of enrichment, that is, the increased likelihood (versus random) of this sample being in the category.
Bayesian Score: The standard Laplacian-modified Bayesian score.
Mahalanobis Distance: The Mahalanobis distance (MD) is the distance to the center of the training data. The larger the MD, the less trustworthy the prediction.
Mahalanobis Distance p-value: The p-value gives the fraction of training data with an MD greater than or equal to the one for the given sample, assuming normally distributed data. The smaller the p-value, the less trustworthy the prediciton. For highly non-normal X properties (e.g., fingerprints), the MD p-value is wildly inaccurate.

Model Applicability

Unknown features are fingerprint features in the query molecule, but not found or appearing too infrequently in the training set.

1. All properties and OPS components are within expected ranges.

Feature Contribution

Top features for positive contribution

Fingerprint	Bit/Smiles	Feature Structure	Score	Irritant in training set
FCFP_12	178336375	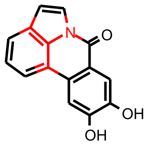 [*]n1:[*]:[*]:[c](:[*]):[c]:1:[c]([*]):[*]	0.202	19 out of 19

FCFP_12	-124655670	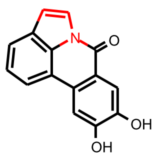 [*]n1:[*]:[*]:[cH]:[cH]:1	0.2	16 out of 16
FCFP_12	17	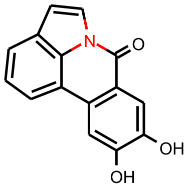 [*]:n:[*]	0.189	48 out of 49
**Top Features for negative contribution**				
**Fingerprint**	**Bit/Smiles**	**Feature Structure**	**Score**	**Irritant in training set**
FCFP_12	991735244	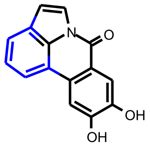 [*][c]1:[*]:[cH]:[cH]:[cH]:[cH]:1	0	237 out of 291
FCFP_12	-620155118	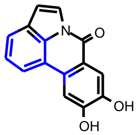 [*]:[c](:[*])[c]1:[cH]:[cH]:[cH]:[*]:[c]:1:[*]	0	26 out of 31
FCFP_12	949015626	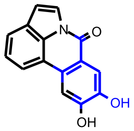 [*]C([*])[c]1:[cH]:[*]:[c]([*]):[c](O):[cH]:1	0	7 out of 9

remdesivir

TOPKAT_Ocular_Irritancy_None_vs_Irritant

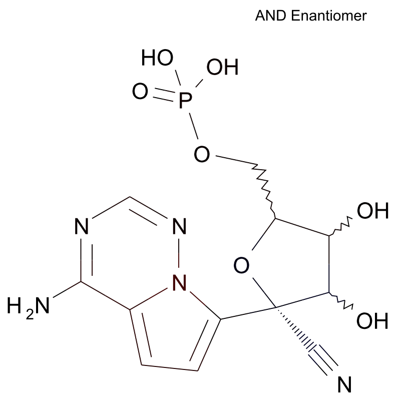

$C_{12}H_{14}N_5O_7P$
Molecular Weight: 371.24262
ALogP: -1.502
Rotatable Bonds: 4
Acceptors: 11
Donors: 5

Structural Similar Compounds

Name	1;3;6-NAPHTHALENE TRISULFONIC ACID;7-AMINO-	Methanol; (s-triazine-2;4;6-triyltrinitrilo)hexa-	2;2'-Biphenyldisulfonic acid; 4;4'-diamino-
Structure	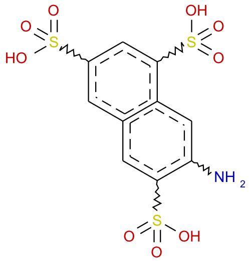	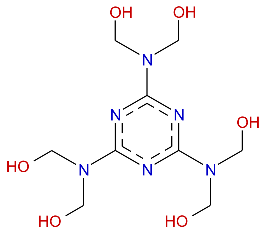	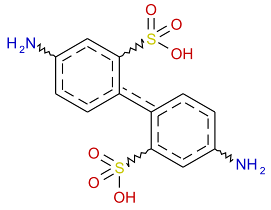
Actual Endpoint	Irritant	Irritant	Irritant
Predicted Endpoint	Irritant	Irritant	Irritant
Distance	0.766	0.795	0.859
Reference	28ZPAK-;190;72	Prehled Prumyslove Toxikologie; Organicke Latky; Marhold; J. -;876;86	Prehled Prumyslove Toxikologie; Organicke Latky; Marhold; J. pp 1061;86

Model Prediction

Prediction: Irritant
Probability: 1
Enrichment: 1.18
Bayesian Score: 1.33
Mahalanobis Distance: 10.7
Mahalanobis Distance p-value: 0.0147

Prediction: Positive if the Bayesian score is above the estimated best cutoff value from minimizing the false positive and false negative rate.
Probability: The esimated probability that the sample is in the positive category. This assumes that the Bayesian score follows a normal distribution and is different from the prediction using a cutoff.
Enrichment: An estimate of enrichment, that is, the increased likelihood (versus random) of this sample being in the category.
Bayesian Score: The standard Laplacian-modified Bayesian score.
Mahalanobis Distance: The Mahalanobis distance (MD) is the distance to the center of the training data. The larger the MD, the less trustworthy the prediction.
Mahalanobis Distance p-value: The p-value gives the fraction of training data with an MD greater than or equal to the one for the given sample, assuming normally distributed data. The smaller the p-value, the less trustworthy the prediciton. For highly non-normal X properties (e.g., fingerprints), the MD p-value is wildly inaccurate.

Model Applicability

Unknown features are fingerprint features in the query molecule, but not found or appearing too infrequently in the training set.

1. All properties and OPS components are within expected ranges.
2. Unknown FCFP_2 feature: 472180098: [*]OP(=O)(O)O
3. Unknown FCFP_2 feature: -124685461: [*]:n:[cH]:n:[*]
4. Unknown FCFP_2 feature: -1151884458: [*]:n:[c](N):[c](:[*]):[*]

Feature Contribution

Top features for positive contribution				
Fingerprint	Bit/Smiles	Feature Structure	Score	Irritant in training set

FCFP_12	1747237384	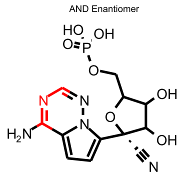 [*][c]1:[*]:[*]:[c]([*]):n:1	0.208	44 out of 44
FCFP_12	178336375	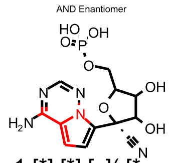 [*]n1:[*]:[*]:[c](:[*]):[c]:1:[c]([*]):[*]	0.202	19 out of 19
FCFP_12	713358128	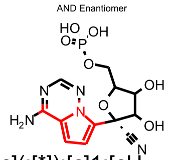 [*][c](:[*]):[c]1:[cH]:[cH]:[c]([*]):n:1:[*]	0.2	17 out of 17
**Top Features for negative contribution**				
**Fingerprint**	**Bit/Smiles**	**Feature Structure**	**Score**	**Irritant in training set**
FCFP_12	-836603894	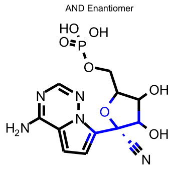 [*]C1[*][*]O[C@]1(C#[*])[c](:[*]):[*]	-0.592	0 out of 1
FCFP_12	-1277879912	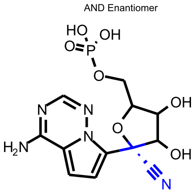 [*]C([*])([*])C#N	-0.0939	33 out of 45

FCFP_12	-1272768868	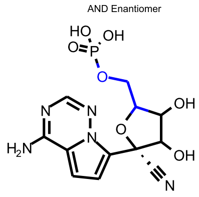<br>[*]OCC([*])[*]	0	396 out of 514

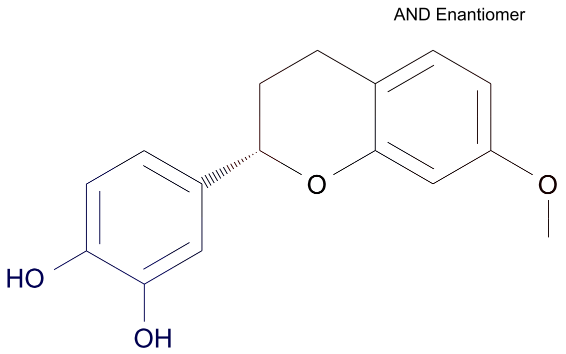

$C_{16}H_{16}O_4$
Molecular Weight: 272.296
ALogP: 3.456
Rotatable Bonds: 2
Acceptors: 4
Donors: 2

Model Prediction

Prediction: Carcinogen
Probability: 0.293
Enrichment: 0.909
Bayesian Score: -0.3
Mahalanobis Distance: 11.1
Mahalanobis Distance p-value: 0.0502

Prediction: Positive if the Bayesian score is above the estimated best cutoff value from minimizing the false positive and false negative rate.
Probability: The esimated probability that the sample is in the positive category. This assumes that the Bayesian score follows a normal distribution and is different from the prediction using a cutoff.
Enrichment: An estimate of enrichment, that is, the increased likelihood (versus random) of this sample being in the category.
Bayesian Score: The standard Laplacian-modified Bayesian score.
Mahalanobis Distance: The Mahalanobis distance (MD) is the distance to the center of the training data. The larger the MD, the less trustworthy the prediction.
Mahalanobis Distance p-value: The p-value gives the fraction of training data with an MD greater than or equal to the one for the given sample, assuming normally distributed data. The smaller the p-value, the less trustworthy the prediciton. For highly non-normal X properties (e.g., fingerprints), the MD p-value is wildly inaccurate.

Structural Similar Compounds

Name	Diflunisal	Phenolphthalein	Oxazepam
Structure	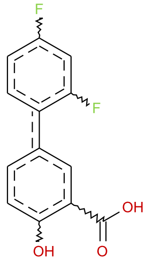	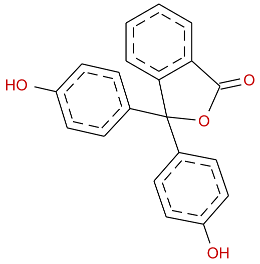	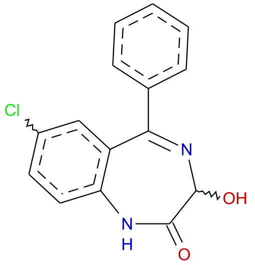
Actual Endpoint	Non-Carcinogen	Carcinogen	Non-Carcinogen
Predicted Endpoint	Non-Carcinogen	Carcinogen	Non-Carcinogen
Distance	0.570	0.570	0.602
Reference	US FDA (Centre for Drug Eval.& Res./Off. Testing & Res.) Sept. 1997	US FDA (Centre for Drug Eval.& Res./Off. Testing & Res.) Sept. 1997	US FDA (Centre for Drug Eval.& Res./Off. Testing & Res.) Sept. 1997

Model Applicability

Unknown features are fingerprint features in the query molecule, but not found or appearing too infrequently in the training set.

1. All properties and OPS components are within expected ranges.
2. Unknown ECFP_2 feature: -856154029: [*]C[C@H](O[*])[c](:[*]):[*]

Feature Contribution

Top features for positive contribution

Fingerprint	Bit/Smiles	Feature Structure	Score	Carcinogen in training set
ECFP_12	-1095664289	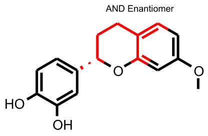 [*][C@H]1[*][c](:[*]) :[c](CC1):[cH]:[*]	0.33	3 out of 6

ECFP_12	683445015	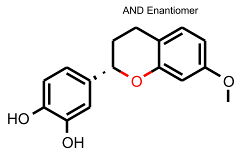<br>[*]O[*]	0.294	28 out of 66
ECFP_12	1588719643	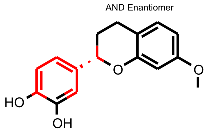<br>[*]C([*])[c]1:[cH]:[*]:[c]([*]):[cH]:[cH]:1	0.288	2 out of 4
**Top Features for negative contribution**				
**Fingerprint**	**Bit/Smiles**	**Feature Structure**	**Score**	**Carcinogen in training set**
ECFP_12	-101223435	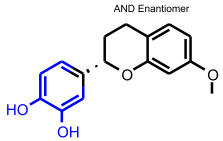<br>O[c]1:[cH]:[*]:[cH]:[cH]:[c]:1O	-0.485	0 out of 2
ECFP_12	-1364960748	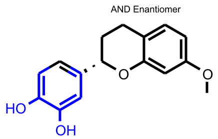<br>[*][c]1:[*]:[cH]:[c](O):[c](O):[cH]:1	-0.485	0 out of 2
ECFP_12	1310213750	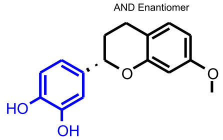<br>[*][c]1:[cH]:[cH]:[c](O):[c](O):[cH]:1	-0.485	0 out of 2

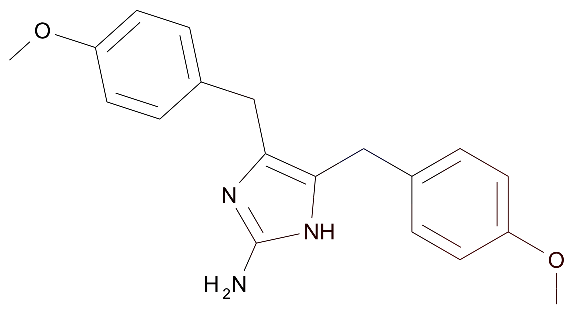

$C_{19}H_{21}N_3O_2$
Molecular Weight: 323.389
ALogP: 3.305
Rotatable Bonds: 6
Acceptors: 4
Donors: 2

Model Prediction

Prediction: Carcinogen
Probability: 0.303
Enrichment: 0.94
Bayesian Score: 0.197
Mahalanobis Distance: 9.73
Mahalanobis Distance p-value: 0.496

Prediction: Positive if the Bayesian score is above the estimated best cutoff value from minimizing the false positive and false negative rate.
Probability: The esimated probability that the sample is in the positive category. This assumes that the Bayesian score follows a normal distribution and is different from the prediction using a cutoff.
Enrichment: An estimate of enrichment, that is, the increased likelihood (versus random) of this sample being in the category.
Bayesian Score: The standard Laplacian-modified Bayesian score.
Mahalanobis Distance: The Mahalanobis distance (MD) is the distance to the center of the training data. The larger the MD, the less trustworthy the prediction.
Mahalanobis Distance p-value: The p-value gives the fraction of training data with an MD greater than or equal to the one for the given sample, assuming normally distributed data. The smaller the p-value, the less trustworthy the prediciton. For highly non-normal X properties (e.g., fingerprints), the MD p-value is wildly inaccurate.

Structural Similar Compounds

Name	Bunolol	Metoclopramide	Mebendazole
Structure	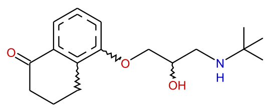	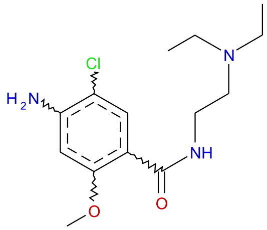	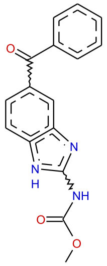
Actual Endpoint	Non-Carcinogen	Non-Carcinogen	Non-Carcinogen
Predicted Endpoint	Non-Carcinogen	Non-Carcinogen	Non-Carcinogen
Distance	0.592	0.627	0.630
Reference	US FDA (Centre for Drug Eval.& Res./Off. Testing & Res.) Sept. 1997	US FDA (Centre for Drug Eval.& Res./Off. Testing & Res.) Sept. 1997	US FDA (Centre for Drug Eval.& Res./Off. Testing & Res.) Sept. 1997

Model Applicability

Unknown features are fingerprint features in the query molecule, but not found or appearing too infrequently in the training set.

1. All properties and OPS components are within expected ranges.
2. Unknown ECFP_2 feature: -2046255371: N[c]1:[nH]:[*]:[*]:n:1

Feature Contribution

Top features for positive contribution

Fingerprint	Bit/Smiles	Feature Structure	Score	Carcinogen in training set
ECFP_12	558201926	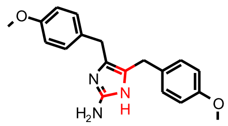 [*][c]1:[*]:[*]:[c]([*]):[nH]:1	0.539	5 out of 8

ECFP_12	-152683720	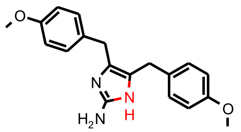 [*]:[nH]:[*]	0.412	9 out of 18
ECFP_12	-1271104377	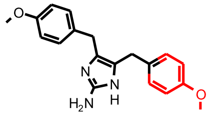 CO[c]1:[cH]:[cH]:[*]:[cH]:[cH]:1	0.33	3 out of 6

Top Features for negative contribution

Fingerprint	Bit/Smiles	Feature Structure	Score	Carcinogen in training set
ECFP_12	765434811	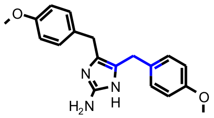 [*]:[c](:[*])C[c](:[*]):[*]	-0.272	0 out of 1
ECFP_12	-746759483	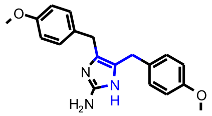 [*]C[c]1:[nH]:[*]:[*]:[c]:1[*]	-0.272	0 out of 1
ECFP_12	864909220	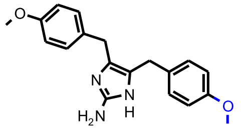 [*]OC	-0.147	12 out of 45

TOPKAT_Rat_Female_FDA_None_vs_Carcinogen

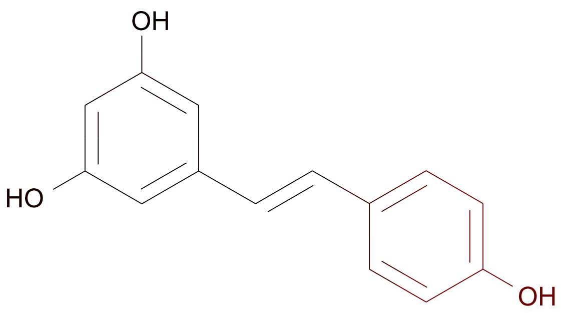

$C_{14}H_{12}O_3$
Molecular Weight: 228.243
ALogP: 3.09
Rotatable Bonds: 2
Acceptors: 3
Donors: 3

Model Prediction

Prediction: Carcinogen
Probability: 0.339
Enrichment: 1.05
Bayesian Score: 1.82
Mahalanobis Distance: 7.44
Mahalanobis Distance p-value: 0.999

Prediction: Positive if the Bayesian score is above the estimated best cutoff value from minimizing the false positive and false negative rate.
Probability: The esimated probability that the sample is in the positive category. This assumes that the Bayesian score follows a normal distribution and is different from the prediction using a cutoff.
Enrichment: An estimate of enrichment, that is, the increased likelihood (versus random) of this sample being in the category.
Bayesian Score: The standard Laplacian-modified Bayesian score.
Mahalanobis Distance: The Mahalanobis distance (MD) is the distance to the center of the training data. The larger the MD, the less trustworthy the prediction.
Mahalanobis Distance p-value: The p-value gives the fraction of training data with an MD greater than or equal to the one for the given sample, assuming normally distributed data. The smaller the p-value, the less trustworthy the prediciton. For highly non-normal X properties (e.g., fingerprints), the MD p-value is wildly inaccurate.

Structural Similar Compounds

Name	Diflunisal	Guanabenz	Guanfacine
Structure	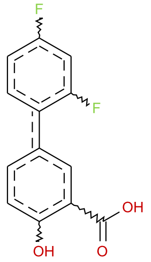	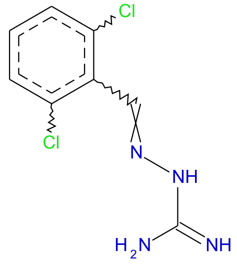	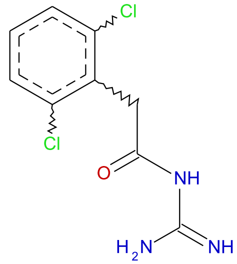
Actual Endpoint	Non-Carcinogen	Non-Carcinogen	Non-Carcinogen
Predicted Endpoint	Non-Carcinogen	Non-Carcinogen	Non-Carcinogen
Distance	0.612	0.635	0.653
Reference	US FDA (Centre for Drug Eval.& Res./Off. Testing & Res.) Sept. 1997	US FDA (Centre for Drug Eval.& Res./Off. Testing & Res.) Sept. 1997	US FDA (Centre for Drug Eval.& Res./Off. Testing & Res.) Sept. 1997

Model Applicability

Unknown features are fingerprint features in the query molecule, but not found or appearing too infrequently in the training set.

1. All properties and OPS components are within expected ranges.
2. Unknown ECFP_2 feature: -176483725: [*]=C[c](:c:[*]):c:[*]

Feature Contribution

Top features for positive contribution

Fingerprint	Bit/Smiles	Feature Structure	Score	Carcinogen in training set
ECFP_12	1419645508	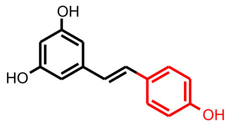 [*][c]1:[cH]:[cH]:[c](O):[cH]:[cH]:1	0.736	5 out of 6

ECFP_12	1740779540	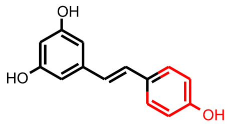 O[c]1:[cH]:[cH]:[*]:[cH]:[cH]:1	0.633	5 out of 7
ECFP_12	-790637051	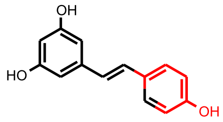 [*][c]1:[*]:[cH]:[c](O):[cH]:[cH]:1	0.529	6 out of 10
**Top Features for negative contribution**				
**Fingerprint**	**Bit/Smiles**	**Feature Structure**	**Score**	**Carcinogen in training set**
ECFP_12	-1884411803	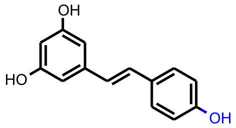 [*]O	-0.106	55 out of 196
ECFP_12	-786013480	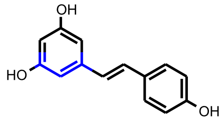 [*][c](:[*]):[cH]:[c]([*]):[*]	-0.0828	33 out of 115
ECFP_12	-1831055759	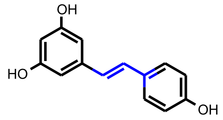 [*]\C=C\[c](:[*]):[*]	-0.0606	2 out of 7

TOPKAT_Rat_Female_FDA_None_vs_Carcinogen

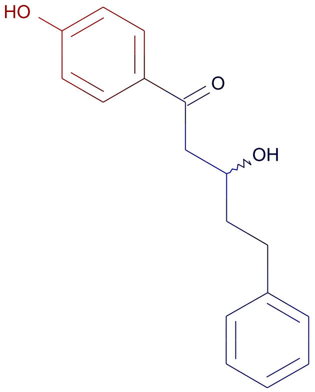

$C_{17}H_{18}O_3$
Molecular Weight: 270.323
ALogP: 3.293
Rotatable Bonds: 6
Acceptors: 3
Donors: 2

Structural Similar Compounds

Name	Propranolol	Penbutalol	Bunolol
Structure	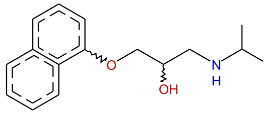	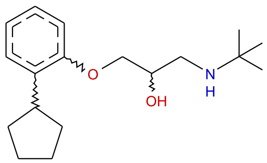	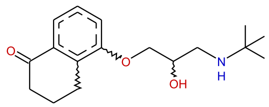
Actual Endpoint	Non-Carcinogen	Non-Carcinogen	Non-Carcinogen
Predicted Endpoint	Non-Carcinogen	Non-Carcinogen	Non-Carcinogen
Distance	0.562	0.589	0.595
Reference	US FDA (Centre for Drug Eval.& Res./Off. Testing & Res.) Sept. 1997	US FDA (Centre for Drug Eval.& Res./Off. Testing & Res.) Sept. 1997	US FDA (Centre for Drug Eval.& Res./Off. Testing & Res.) Sept. 1997

Model Prediction

Prediction: Non-Carcinogen
Probability: 0.247
Enrichment: 0.767
Bayesian Score: -2.98
Mahalanobis Distance: 14.2
Mahalanobis Distance p-value: 8.15e-007

Prediction: Positive if the Bayesian score is above the estimated best cutoff value from minimizing the false positive and false negative rate.
Probability: The esimated probability that the sample is in the positive category. This assumes that the Bayesian score follows a normal distribution and is different from the prediction using a cutoff.
Enrichment: An estimate of enrichment, that is, the increased likelihood (versus random) of this sample being in the category.
Bayesian Score: The standard Laplacian-modified Bayesian score.
Mahalanobis Distance: The Mahalanobis distance (MD) is the distance to the center of the training data. The larger the MD, the less trustworthy the prediction.
Mahalanobis Distance p-value: The p-value gives the fraction of training data with an MD greater than or equal to the one for the given sample, assuming normally distributed data. The smaller the p-value, the less trustworthy the prediciton. For highly non-normal X properties (e.g., fingerprints), the MD p-value is wildly inaccurate.

Model Applicability

Unknown features are fingerprint features in the query molecule, but not found or appearing too infrequently in the training set.

1. All properties and OPS components are within expected ranges.

Feature Contribution

Top features for positive contribution

Fingerprint	Bit/Smiles	Feature Structure	Score	Carcinogen in training set
ECFP_12	1419645508	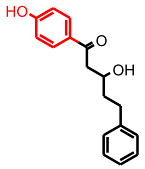 [*][c]1:[cH]:[cH]:[c](O):[cH]:[cH]:1	0.736	5 out of 6

ECFP_12	1740779540	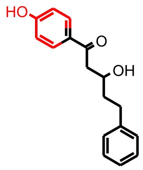<br>O[c]1:[cH]:[cH]:[*]:[<br>cH]:[cH]:1	0.633	5 out of 7
ECFP_12	1187082817	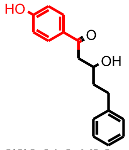<br>[*]C(=[*])[c]1:[cH]:[<br>cH]:[c](O):[cH]:[cH]<br>:1	0.613	2 out of 2
**Top Features for negative contribution**				
**Fingerprint**	**Bit/Smiles**	**Feature Structure**	**Score**	**Carcinogen in training set**
ECFP_12	-1310859884	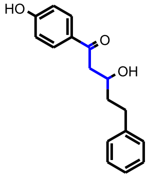<br>[*]C([*])CC(=[*])[*]	-0.661	0 out of 3
ECFP_12	196083830	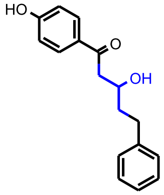<br>[*]CC(O)C[*]	-0.64	2 out of 15
ECFP_12	-281505363	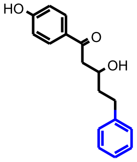<br>[*][c]1:[cH]:[cH]:[cH<br>]:[cH]:[cH]:1	-0.56	11 out of 64

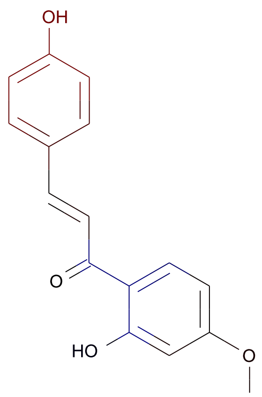

$C_{16}H_{14}O_4$
Molecular Weight: 270.28
ALogP: 3.201
Rotatable Bonds: 4
Acceptors: 4
Donors: 2

Model Prediction

Prediction: Carcinogen
Probability: 0.295
Enrichment: 0.918
Bayesian Score: -0.152
Mahalanobis Distance: 10.1
Mahalanobis Distance p-value: 0.341

Prediction: Positive if the Bayesian score is above the estimated best cutoff value from minimizing the false positive and false negative rate.
Probability: The esimated probability that the sample is in the positive category. This assumes that the Bayesian score follows a normal distribution and is different from the prediction using a cutoff.
Enrichment: An estimate of enrichment, that is, the increased likelihood (versus random) of this sample being in the category.
Bayesian Score: The standard Laplacian-modified Bayesian score.
Mahalanobis Distance: The Mahalanobis distance (MD) is the distance to the center of the training data. The larger the MD, the less trustworthy the prediction.
Mahalanobis Distance p-value: The p-value gives the fraction of training data with an MD greater than or equal to the one for the given sample, assuming normally distributed data. The smaller the p-value, the less trustworthy the prediciton. For highly non-normal X properties (e.g., fingerprints), the MD p-value is wildly inaccurate.

Structural Similar Compounds

Name	Mebendazole	Etodolac	Cytembena
Structure	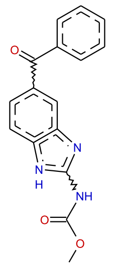	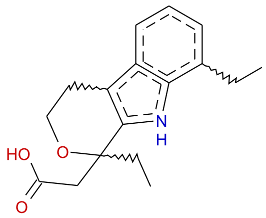	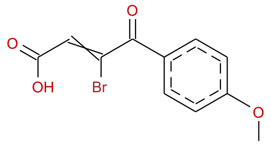
Actual Endpoint	Non-Carcinogen	Non-Carcinogen	Carcinogen
Predicted Endpoint	Non-Carcinogen	Non-Carcinogen	Carcinogen
Distance	0.540	0.583	0.594
Reference	US FDA (Centre for Drug Eval.& Res./Off. Testing & Res.) Sept. 1997	US FDA (Centre for Drug Eval.& Res./Off. Testing & Res.) Sept. 1997	US FDA (Centre for Drug Eval.& Res./Off. Testing & Res.) Sept. 1997

Model Applicability

Unknown features are fingerprint features in the query molecule, but not found or appearing too infrequently in the training set.

1. All properties and OPS components are within expected ranges.
2. Unknown ECFP_2 feature: 1430764055: [*]=CC(=O)[c](:[*]):[*]
3. Unknown ECFP_2 feature: -176483725: [*]=C[c](:c:[*]):c:[*]

Feature Contribution

Top features for positive contribution

Fingerprint	Bit/Smiles	Feature Structure	Score	Carcinogen in training set
ECFP_12	1419645508	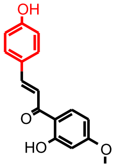 [*][c]1:[cH]:[cH]:[c](O):[cH]:[cH]:1	0.736	5 out of 6

ECFP_12	1740779540	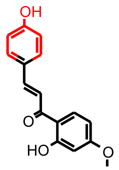 O[c]1:[cH]:[cH]:[*]:[cH]:[cH]:1	0.633	5 out of 7
ECFP_12	-790637051	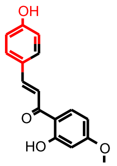 [*][c]1:[*]:[cH]:[c](O):[cH]:[cH]:1	0.529	6 out of 10
**Top Features for negative contribution**				
**Fingerprint**	**Bit/Smiles**	**Feature Structure**	**Score**	**Carcinogen in training set**
ECFP_12	1337040050	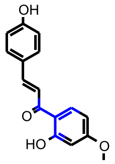 [*]C(=[*])[c](:[cH]:[*]):[c]([*]):[*]	-1.84	0 out of 17
ECFP_12	2078324322	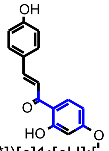 [*]C(=[*])[c]1:[cH]:[cH]:[c]([*]):[*]:[c] :1[*]	-0.485	0 out of 2
ECFP_12	1334400011	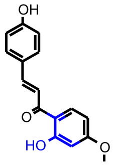 [*][c](:[*]):[c](O):[cH]:[*]	-0.189	5 out of 20

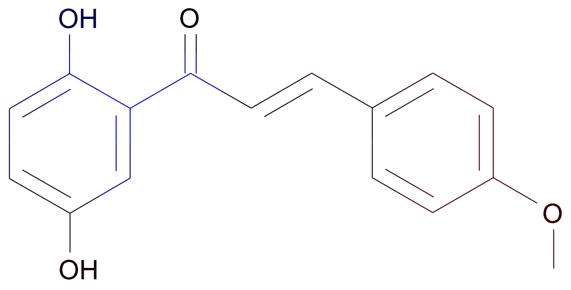

$C_{16}H_{14}O_4$
Molecular Weight: 270.28
ALogP: 3.201
Rotatable Bonds: 4
Acceptors: 4
Donors: 2

Model Prediction

Prediction: Non-Carcinogen
Probability: 0.263
Enrichment: 0.818
Bayesian Score: -1.93
Mahalanobis Distance: 10.1
Mahalanobis Distance p-value: 0.341

Prediction: Positive if the Bayesian score is above the estimated best cutoff value from minimizing the false positive and false negative rate.
Probability: The esimated probability that the sample is in the positive category. This assumes that the Bayesian score follows a normal distribution and is different from the prediction using a cutoff.
Enrichment: An estimate of enrichment, that is, the increased likelihood (versus random) of this sample being in the category.
Bayesian Score: The standard Laplacian-modified Bayesian score.
Mahalanobis Distance: The Mahalanobis distance (MD) is the distance to the center of the training data. The larger the MD, the less trustworthy the prediction.
Mahalanobis Distance p-value: The p-value gives the fraction of training data with an MD greater than or equal to the one for the given sample, assuming normally distributed data. The smaller the p-value, the less trustworthy the prediciton. For highly non-normal X properties (e.g., fingerprints), the MD p-value is wildly inaccurate.

Structural Similar Compounds

Name	Mebendazole	Cytembena	Etodolac
Structure	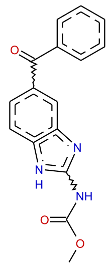	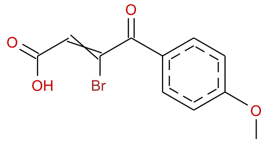	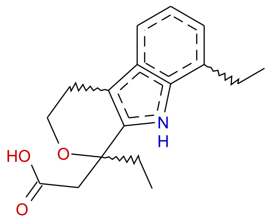
Actual Endpoint	Non-Carcinogen	Carcinogen	Non-Carcinogen
Predicted Endpoint	Non-Carcinogen	Carcinogen	Non-Carcinogen
Distance	0.539	0.579	0.583
Reference	US FDA (Centre for Drug Eval.& Res./Off. Testing & Res.) Sept. 1997	US FDA (Centre for Drug Eval.& Res./Off. Testing & Res.) Sept. 1997	US FDA (Centre for Drug Eval.& Res./Off. Testing & Res.) Sept. 1997

Model Applicability

Unknown features are fingerprint features in the query molecule, but not found or appearing too infrequently in the training set.

1. All properties and OPS components are within expected ranges.
2. Unknown ECFP_2 feature: -176483725: [*]=C[c](:c:[*]):c:[*]
3. Unknown ECFP_2 feature: 1430764055: [*]=CC(=O)[c](:[*]):[*]

Feature Contribution

Top features for positive contribution

Fingerprint	Bit/Smiles	Feature Structure	Score	Carcinogen in training set
ECFP_12	-790637051	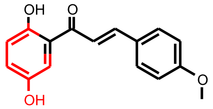 [*][c]1:[*]:[cH]:[c](O):[cH]:[cH]:1	0.529	6 out of 10

ECFP_12	-1925046727	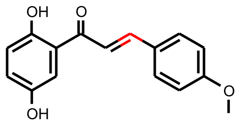<br>[*]C=[*]	0.407	16 out of 33
ECFP_12	-177786161	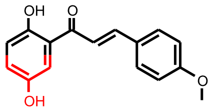<br>[*]:[cH]:[c](O):[cH]:<br>[*]	0.341	7 out of 15

Top Features for negative contribution

Fingerprint	Bit/Smiles	Feature Structure	Score	Carcinogen in training set
ECFP_12	1337040050	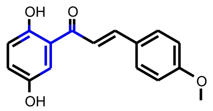<br>[*]C(=[*])[c](:[cH]:[<br>*]):[c]([*]):[*]	-1.84	0 out of 17
ECFP_12	-104590004	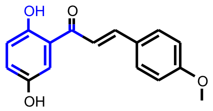<br>[*]C(=[*])[c]1:[cH]:[<br>*]:[cH]:[cH]:[c]:1O	-1.06	0 out of 6
ECFP_12	477042227	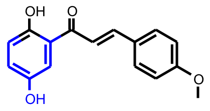<br>[*][c]1:[*]:[cH]:[cH]<br>:[c](O):[cH]:1	-0.363	1 out of 6

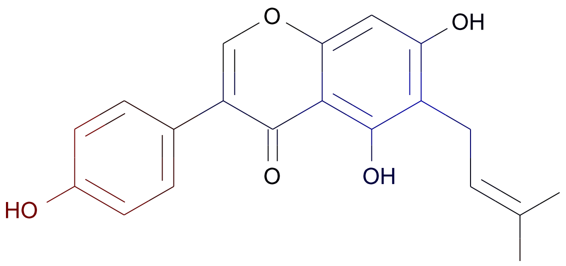

$C_{20}H_{18}O_5$
Molecular Weight: 338.354
ALogP: 3.997
Rotatable Bonds: 3
Acceptors: 5
Donors: 3

Model Prediction

Prediction: Non-Carcinogen
Probability: 0.248
Enrichment: 0.769
Bayesian Score: -2.95
Mahalanobis Distance: 11.5
Mahalanobis Distance p-value: 0.0184

Prediction: Positive if the Bayesian score is above the estimated best cutoff value from minimizing the false positive and false negative rate.
Probability: The esimated probability that the sample is in the positive category. This assumes that the Bayesian score follows a normal distribution and is different from the prediction using a cutoff.
Enrichment: An estimate of enrichment, that is, the increased likelihood (versus random) of this sample being in the category.
Bayesian Score: The standard Laplacian-modified Bayesian score.
Mahalanobis Distance: The Mahalanobis distance (MD) is the distance to the center of the training data. The larger the MD, the less trustworthy the prediction.
Mahalanobis Distance p-value: The p-value gives the fraction of training data with an MD greater than or equal to the one for the given sample, assuming normally distributed data. The smaller the p-value, the less trustworthy the prediciton. For highly non-normal X properties (e.g., fingerprints), the MD p-value is wildly inaccurate.

Structural Similar Compounds

Name	Phenolphthalein	Torsemide	Ursodiol
Structure	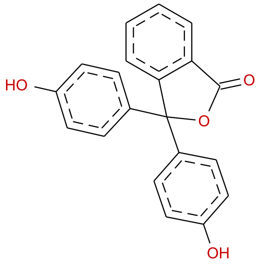	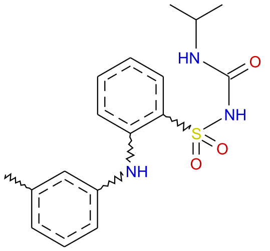	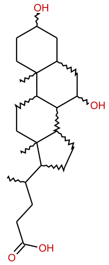
Actual Endpoint	Carcinogen	Carcinogen	Carcinogen
Predicted Endpoint	Carcinogen	Carcinogen	Carcinogen
Distance	0.637	0.644	0.655
Reference	US FDA (Centre for Drug Eval.& Res./Off. Testing & Res.) Sept. 1997	US FDA (Centre for Drug Eval.& Res./Off. Testing & Res.) Sept. 1997	US FDA (Centre for Drug Eval.& Res./Off. Testing & Res.) Sept. 1997

Model Applicability

Unknown features are fingerprint features in the query molecule, but not found or appearing too infrequently in the training set.

1. All properties and OPS components are within expected ranges.
2. Unknown ECFP_2 feature: -1774681326: [*]C=C(C)C
3. Unknown ECFP_2 feature: 1717082529: [*]\C=C(/C(=[*])[*])\[c](:[*]):[*]
4. Unknown ECFP_2 feature: 471124258: [*]OC=C([*])[*]
5. Unknown ECFP_2 feature: -554736825: [*]=CO[c](:[*]):[*]

Feature Contribution

Top features for positive contribution

Fingerprint	Bit/Smiles	Feature Structure	Score	Carcinogen in training set
ECFP_12	1419645508	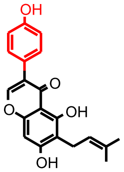 [*][c]1:[cH]:[cH]:[c](O):[cH]:[cH]:1	0.736	5 out of 6

ECFP_12	1740779540	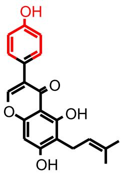<br>O[c]1:[cH]:[cH]:[*]:[cH]:[cH]:1	0.633	5 out of 7
ECFP_12	-790637051	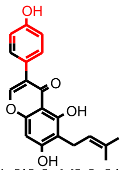<br>[*][c]1:[*]:[cH]:[c](O):[cH]:[cH]:1	0.529	6 out of 10
**Top Features for negative contribution**				
**Fingerprint**	**Bit/Smiles**	**Feature Structure**	**Score**	**Carcinogen in training set**
ECFP_12	-1660913849	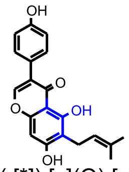<br>[*][c](:[*]):[c](O):[c]([*]):[*]	-0.941	0 out of 5
ECFP_12	-512323383	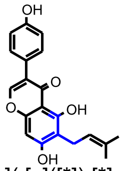<br>[*]C[c](:[c]([*]):[*]):[c]([*]):[*]	-0.941	0 out of 5
ECFP_12	-181568884	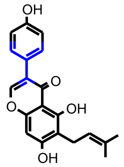<br>[*]C(=[*])[c](:[cH]:[*]):[cH]:[*]	-0.505	3 out of 18

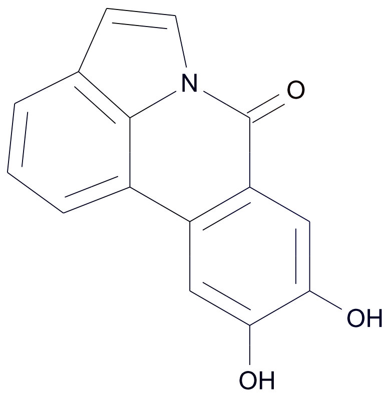

$C_{15}H_9NO_3$
Molecular Weight: 251.237
ALogP: 2.982
Rotatable Bonds: 0
Acceptors: 3
Donors: 2

Model Prediction

Prediction: Non-Carcinogen
Probability: 0.256
Enrichment: 0.794
Bayesian Score: -2.41
Mahalanobis Distance: 10.2
Mahalanobis Distance p-value: 0.27

Prediction: Positive if the Bayesian score is above the estimated best cutoff value from minimizing the false positive and false negative rate.
Probability: The esimated probability that the sample is in the positive category. This assumes that the Bayesian score follows a normal distribution and is different from the prediction using a cutoff.
Enrichment: An estimate of enrichment, that is, the increased likelihood (versus random) of this sample being in the category.
Bayesian Score: The standard Laplacian-modified Bayesian score.
Mahalanobis Distance: The Mahalanobis distance (MD) is the distance to the center of the training data. The larger the MD, the less trustworthy the prediction.
Mahalanobis Distance p-value: The p-value gives the fraction of training data with an MD greater than or equal to the one for the given sample, assuming normally distributed data. The smaller the p-value, the less trustworthy the prediciton. For highly non-normal X properties (e.g., fingerprints), the MD p-value is wildly inaccurate.

Structural Similar Compounds

Name	Oxazepam	Diflunisal	Proflavine
Structure	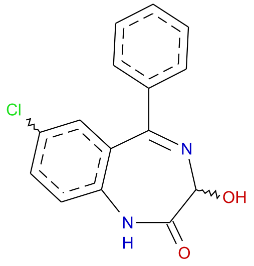	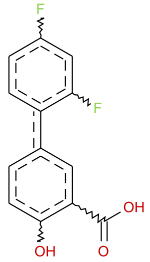	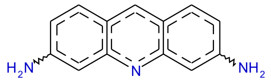
Actual Endpoint	Non-Carcinogen	Non-Carcinogen	Non-Carcinogen
Predicted Endpoint	Non-Carcinogen	Non-Carcinogen	Non-Carcinogen
Distance	0.567	0.586	0.607
Reference	US FDA (Centre for Drug Eval.& Res./Off. Testing & Res.) Sept. 1997	US FDA (Centre for Drug Eval.& Res./Off. Testing & Res.) Sept. 1997	US FDA (Centre for Drug Eval.& Res./Off. Testing & Res.) Sept. 1997

Model Applicability

Unknown features are fingerprint features in the query molecule, but not found or appearing too infrequently in the training set.

1. All properties and OPS components are within expected ranges.
2. Unknown ECFP_2 feature: -1660898726: [*]n1:[*]:[*]:[c](:[*]):[c]:1:[c]([*]):[*]
3. Unknown ECFP_2 feature: 1444648700: [*]:n(:[*])C(=O)[c](:[*]):[*]

Feature Contribution

Top features for positive contribution

Fingerprint	Bit/Smiles	Feature Structure	Score	Carcinogen in training set
ECFP_12	717474525	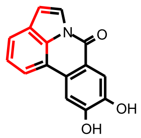 [*]1:[*]:[c]2:[*]:[cH]:[cH]:[cH]:[c]:2:[cH]:1	0.158	2 out of 5

ECFP_12	2106656448	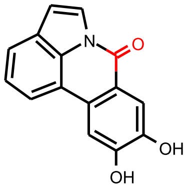 [*]C(=O)[*]	0.141	30 out of 83
ECFP_12	-1074141656	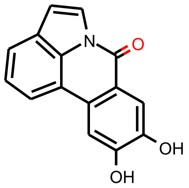 [*]=O	0.103	86 out of 248
**Top Features for negative contribution**				
**Fingerprint**	**Bit/Smiles**	**Feature Structure**	**Score**	**Carcinogen in training set**
ECFP_12	-1364960748	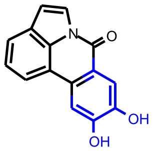 [*][c]1:[*]:[cH]:[c](O):[c](O):[cH]:1	-0.485	0 out of 2
ECFP_12	2007300961	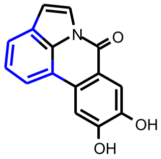 [*][c]1:[*]:[c](:[*]) :[cH]:[cH]:[cH]:1	-0.426	7 out of 36
ECFP_12	1997021792	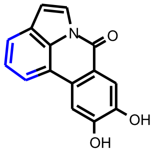 [*]:[cH]:[cH]:[cH]:[*]	-0.296	36 out of 156

remdesivir

TOPKAT_Rat_Female_FDA_None_vs_Carcinogen

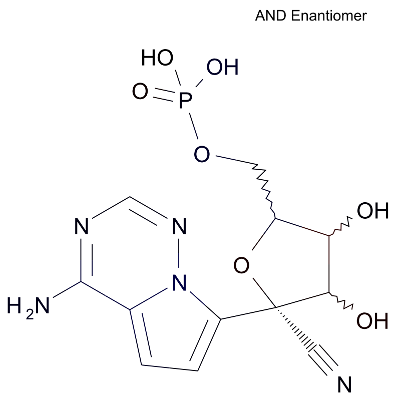

$C_{12}H_{14}N_5O_7P$
Molecular Weight: 371.24262
ALogP: -1.502
Rotatable Bonds: 4
Acceptors: 11
Donors: 5

Model Prediction

Prediction: Non-Carcinogen
Probability: 0.243
Enrichment: 0.756
Bayesian Score: -3.24
Mahalanobis Distance: 13.8
Mahalanobis Distance p-value: 5.04e-006

Prediction: Positive if the Bayesian score is above the estimated best cutoff value from minimizing the false positive and false negative rate.
Probability: The esimated probability that the sample is in the positive category. This assumes that the Bayesian score follows a normal distribution and is different from the prediction using a cutoff.
Enrichment: An estimate of enrichment, that is, the increased likelihood (versus random) of this sample being in the category.
Bayesian Score: The standard Laplacian-modified Bayesian score.
Mahalanobis Distance: The Mahalanobis distance (MD) is the distance to the center of the training data. The larger the MD, the less trustworthy the prediction.
Mahalanobis Distance p-value: The p-value gives the fraction of training data with an MD greater than or equal to the one for the given sample, assuming normally distributed data. The smaller the p-value, the less trustworthy the prediciton. For highly non-normal X properties (e.g., fingerprints), the MD p-value is wildly inaccurate.

Structural Similar Compounds

Name	Streptozocin	Tetracycline	Famotidine
Structure	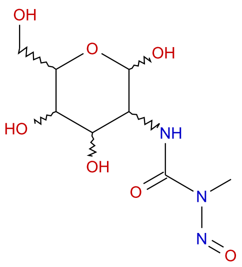	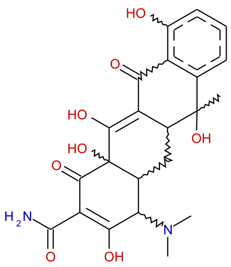	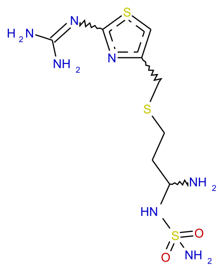
Actual Endpoint	Carcinogen	Non-Carcinogen	Non-Carcinogen
Predicted Endpoint	Carcinogen	Non-Carcinogen	Non-Carcinogen
Distance	0.810	0.858	0.861
Reference	US FDA (Centre for Drug Eval.& Res./Off. Testing & Res.) Sept. 1997	US FDA (Centre for Drug Eval.& Res./Off. Testing & Res.) Sept. 1997	US FDA (Centre for Drug Eval.& Res./Off. Testing & Res.) Sept. 1997

Model Applicability

Unknown features are fingerprint features in the query molecule, but not found or appearing too infrequently in the training set.

1. All properties and OPS components are within expected ranges.
2. Unknown ECFP_2 feature: 1126642748: [*]OP(=O)(O)O
3. Unknown ECFP_2 feature: -1250439909: [*]COP(=[*])([*])[*]
4. Unknown ECFP_2 feature: 1258791451: [*]C1[*][*]O[C@]1(C#[*])[c](:[*]):[*]
5. Unknown ECFP_2 feature: -1507082173: [*][c]1:[*]:[*]:[c](:[*]):n:1:n:[*]
6. Unknown ECFP_2 feature: -66263742: [*]C([*])([*])[c]1:[cH]:[*]:[*]:n:1:[*]

Feature Contribution

Top features for positive contribution				
**Fingerprint**	**Bit/Smiles**	**Feature Structure**	**Score**	**Carcinogen in training set**

ECFP_12	-553149446	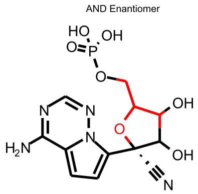 [*]CC1O[*][*]C1[*]	0.575	3 out of 4
ECFP_12	-1114776580	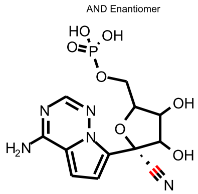 [*]C#[*]	0.461	10 out of 19
ECFP_12	-521596699	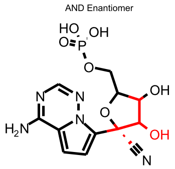 [*]C1[*][*]C([*])([*])C1O	0.445	3 out of 5
**Top Features for negative contribution**				
**Fingerprint**	**Bit/Smiles**	**Feature Structure**	**Score**	**Carcinogen in training set**
ECFP_12	2024329577	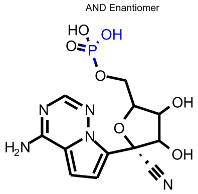 [*]P(=[*])([*])O	-0.661	0 out of 3
ECFP_12	-1687549011	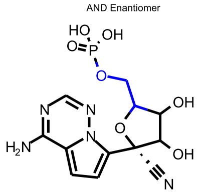 [*]OCC([*])[*]	-0.661	0 out of 3

ECFP_12	-1734834311	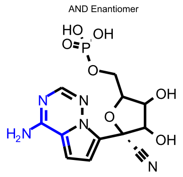 [*]:n:[c](N):[c](:[*]):[*]	-0.56	1 out of 8

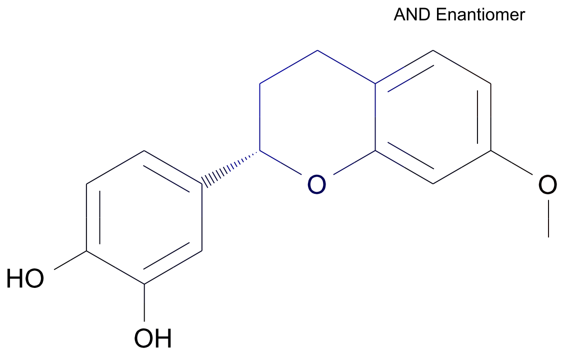

$C_{16}H_{16}O_4$
Molecular Weight: 272.296
ALogP: 3.456
Rotatable Bonds: 2
Acceptors: 4
Donors: 2

Model Prediction

Prediction: Single-Carcinogen
Probability: 0.431
Enrichment: 1.15
Bayesian Score: -2.46
Mahalanobis Distance: 9.97
Mahalanobis Distance p-value: 0.0636

Prediction: Positive if the Bayesian score is above the estimated best cutoff value from minimizing the false positive and false negative rate.
Probability: The esimated probability that the sample is in the positive category. This assumes that the Bayesian score follows a normal distribution and is different from the prediction using a cutoff.
Enrichment: An estimate of enrichment, that is, the increased likelihood (versus random) of this sample being in the category.
Bayesian Score: The standard Laplacian-modified Bayesian score.
Mahalanobis Distance: The Mahalanobis distance (MD) is the distance to the center of the training data. The larger the MD, the less trustworthy the prediction.
Mahalanobis Distance p-value: The p-value gives the fraction of training data with an MD greater than or equal to the one for the given sample, assuming normally distributed data. The smaller the p-value, the less trustworthy the prediciton. For highly non-normal X properties (e.g., fingerprints), the MD p-value is wildly inaccurate.

Structural Similar Compounds

Name	Phenolphthalein	Doxefazepam	Cytembena
Structure	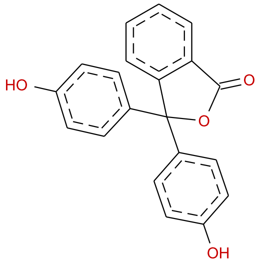	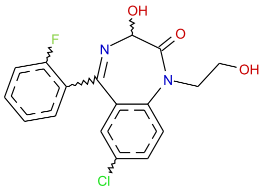	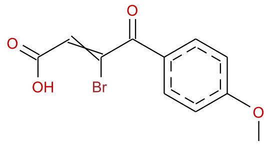
Actual Endpoint	Single-Carcinogen	Multiple-Carcinogen	Multiple-Carcinogen
Predicted Endpoint	Single-Carcinogen	Multiple-Carcinogen	Multiple-Carcinogen
Distance	0.487	0.628	0.631
Reference	US FDA (Centre for Drug Eval.& Res./Off. Testing & Res.) Sept. 1997	US FDA (Centre for Drug Eval.& Res./Off. Testing & Res.) Sept. 1997	US FDA (Centre for Drug Eval.& Res./Off. Testing & Res.) Sept. 1997

Model Applicability

Unknown features are fingerprint features in the query molecule, but not found or appearing too infrequently in the training set.

1. All properties and OPS components are within expected ranges.

Feature Contribution

Top features for positive contribution

Fingerprint	Bit/Smiles	Feature Structure	Score	Multiple-Carcinogen in training set
SCFP_4	1237755852	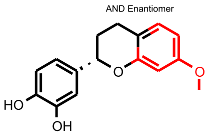 [*][c]1:[*]:[cH]:[cH]:[c](OC):[cH]:1	0.295	5 out of 11

SCFP_4	-1374800107	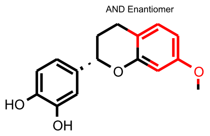 [*]O[c]1:[cH]:[*]:[c]([*]):[cH]:[cH]:1	0.288	10 out of 23
SCFP_4	-424425761	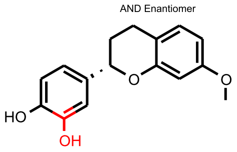 [*]:[c](:[*])O	0.201	6 out of 15

Top Features for negative contribution

**Fingerprint**	**Bit/Smiles**	**Feature Structure**	**Score**	**Multiple-Carcinogen in training set**
SCFP_4	-1272709286	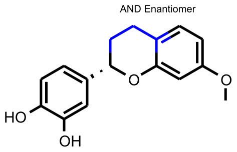 [*]CC[c](:[*]):[*]	-1.16	1 out of 17
SCFP_4	-1849867720	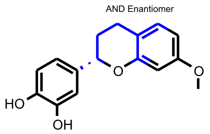 [*][C@H]1[*][c](:[*]):[c](CC1):[cH]:[*]	-0.73	1 out of 10
SCFP_4	1238198777	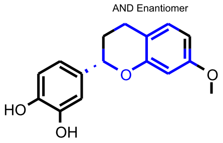 [*][C@@H]1[*]C[c]2:[cH]:[*]:[c]([*]):[cH]:[c]:2O1	-0.489	0 out of 2

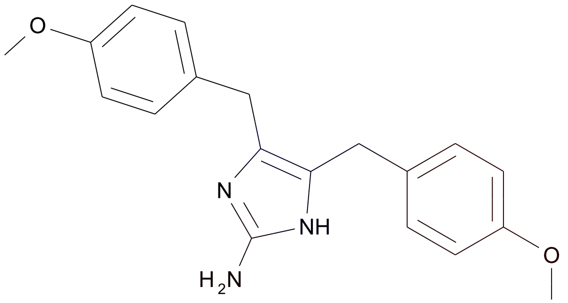

$C_{19}H_{21}N_3O_2$
Molecular Weight: 323.389
ALogP: 3.305
Rotatable Bonds: 6
Acceptors: 4
Donors: 2

Model Prediction

Prediction: Multiple-Carcinogen
Probability: 0.528
Enrichment: 1.41
Bayesian Score: 1.09
Mahalanobis Distance: 12.7
Mahalanobis Distance p-value: 0.000398

Prediction: Positive if the Bayesian score is above the estimated best cutoff value from minimizing the false positive and false negative rate.
Probability: The esimated probability that the sample is in the positive category. This assumes that the Bayesian score follows a normal distribution and is different from the prediction using a cutoff.
Enrichment: An estimate of enrichment, that is, the increased likelihood (versus random) of this sample being in the category.
Bayesian Score: The standard Laplacian-modified Bayesian score.
Mahalanobis Distance: The Mahalanobis distance (MD) is the distance to the center of the training data. The larger the MD, the less trustworthy the prediction.
Mahalanobis Distance p-value: The p-value gives the fraction of training data with an MD greater than or equal to the one for the given sample, assuming normally distributed data. The smaller the p-value, the less trustworthy the prediciton. For highly non-normal X properties (e.g., fingerprints), the MD p-value is wildly inaccurate.

Structural Similar Compounds

Name	Omeprazole	Lansoprazole	Cytembena
Structure	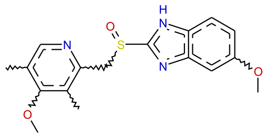	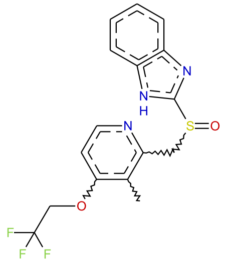	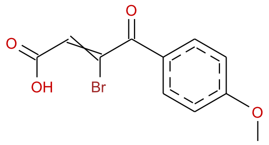
Actual Endpoint	Multiple-Carcinogen	Single-Carcinogen	Multiple-Carcinogen
Predicted Endpoint	Multiple-Carcinogen	Single-Carcinogen	Multiple-Carcinogen
Distance	0.577	0.607	0.630
Reference	US FDA (Centre for Drug Eval.& Res./Off. Testing & Res.) Sept. 1997	US FDA (Centre for Drug Eval.& Res./Off. Testing & Res.) Sept. 1997	US FDA (Centre for Drug Eval.& Res./Off. Testing & Res.) Sept. 1997

Model Applicability

Unknown features are fingerprint features in the query molecule, but not found or appearing too infrequently in the training set.

1. All properties and OPS components are within expected ranges.

Feature Contribution

Top features for positive contribution

Fingerprint	Bit/Smiles	Feature Structure	Score	Multiple-Carcinogen in training set
SCFP_4	1237755852	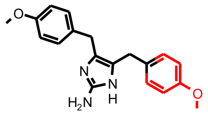 [*][c]1:[*]:[cH]:[cH]:[c](OC):[cH]:1	0.295	5 out of 11

SCFP_4	-1374800107	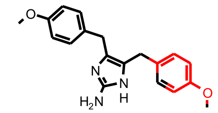	0.288	10 out of 23
SCFP_4	149003983	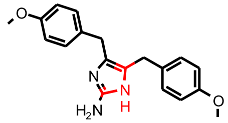	0.266	11 out of 26
**Top Features for negative contribution**				
**Fingerprint**	**Bit/Smiles**	**Feature Structure**	**Score**	**Multiple-Carcinogen in training set**
SCFP_4	2109374332	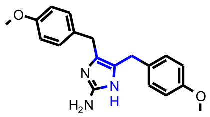	-0.651	1 out of 9
SCFP_4	-502559736	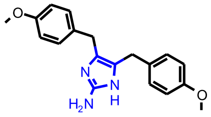	-0.274	0 out of 1
SCFP_4	276283342	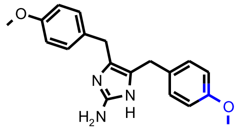	-0.106	5 out of 18

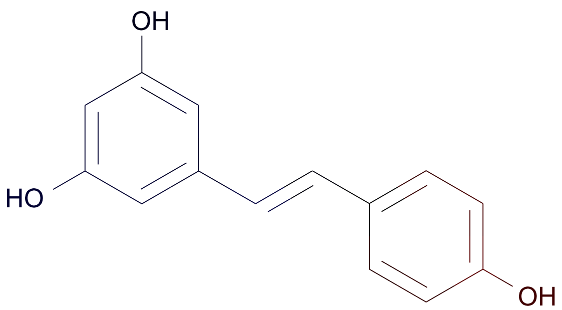

$C_{14}H_{12}O_3$
Molecular Weight: 228.243
ALogP: 3.09
Rotatable Bonds: 2
Acceptors: 3
Donors: 3

Model Prediction

Prediction: Single-Carcinogen
Probability: 0.491
Enrichment: 1.31
Bayesian Score: -0.729
Mahalanobis Distance: 8.67
Mahalanobis Distance p-value: 0.329

Prediction: Positive if the Bayesian score is above the estimated best cutoff value from minimizing the false positive and false negative rate.
Probability: The esimated probability that the sample is in the positive category. This assumes that the Bayesian score follows a normal distribution and is different from the prediction using a cutoff.
Enrichment: An estimate of enrichment, that is, the increased likelihood (versus random) of this sample being in the category.
Bayesian Score: The standard Laplacian-modified Bayesian score.
Mahalanobis Distance: The Mahalanobis distance (MD) is the distance to the center of the training data. The larger the MD, the less trustworthy the prediction.
Mahalanobis Distance p-value: The p-value gives the fraction of training data with an MD greater than or equal to the one for the given sample, assuming normally distributed data. The smaller the p-value, the less trustworthy the prediciton. For highly non-normal X properties (e.g., fingerprints), the MD p-value is wildly inaccurate.

Structural Similar Compounds

Name	Dienestrol	Phenolphthalein	Diethylstilbesterol
Structure	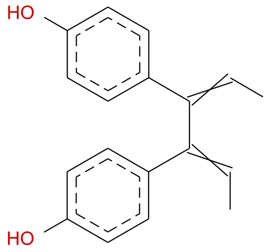	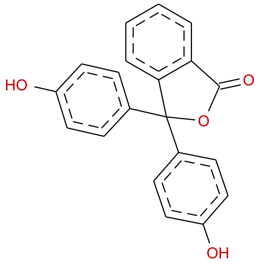	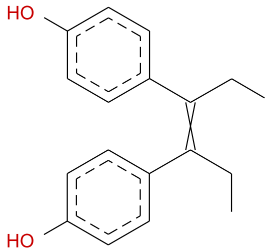
Actual Endpoint	Multiple-Carcinogen	Single-Carcinogen	Multiple-Carcinogen
Predicted Endpoint	Multiple-Carcinogen	Single-Carcinogen	Multiple-Carcinogen
Distance	0.579	0.607	0.618
Reference	US FDA (Centre for Drug Eval.& Res./Off. Testing & Res.) Sept. 1997	US FDA (Centre for Drug Eval.& Res./Off. Testing & Res.) Sept. 1997	US FDA (Centre for Drug Eval.& Res./Off. Testing & Res.) Sept. 1997

Model Applicability

Unknown features are fingerprint features in the query molecule, but not found or appearing too infrequently in the training set.

1. All properties and OPS components are within expected ranges.

Feature Contribution

Top features for positive contribution

Fingerprint	Bit/Smiles	Feature Structure	Score	Multiple-Carcinogen in training set
SCFP_4	611156666	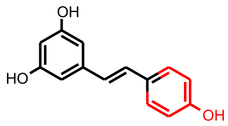 O[c]1:[cH]:[cH]:[*]:[cH]:[cH]:1	0.627	5 out of 7

SCFP_4	387787917	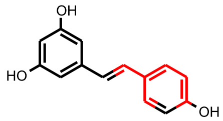 [*][c]1:[*]:[cH]:[c](C=[*]):[cH]:[cH]:1	0.449	6 out of 11
SCFP_4	-1374800107	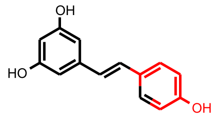 [*]O[c]1:[cH]:[*]:[c] ([*]):[cH]:[cH]:1	0.288	10 out of 23
**Top Features for negative contribution**				
**Fingerprint**	**Bit/Smiles**	**Feature Structure**	**Score**	**Multiple-Carcinogen in training set**
SCFP_4	-1971137145	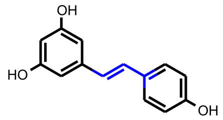 [*]\C=C\[c](:[*]):[*]	-0.489	0 out of 2
SCFP_4	130348166	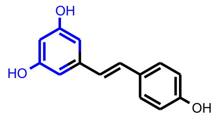 [*]O[c]1:[cH]:[*]:[c] ([*]):[c](O[*]):[cH] :1	-0.489	0 out of 2
SCFP_4	392579710	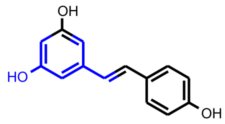 [*]=C[c]1:[cH]:[*]:[c H]:[c](O):[cH]:1	-0.489	0 out of 2

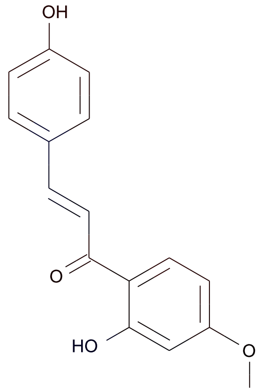

$C_{16}H_{14}O_4$
Molecular Weight: 270.28
ALogP: 3.201
Rotatable Bonds: 4
Acceptors: 4
Donors: 2

Structural Similar Compounds

Name	Cytembena	Phenolphthalein	Doxefazepam
Structure	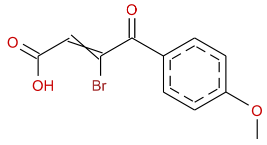	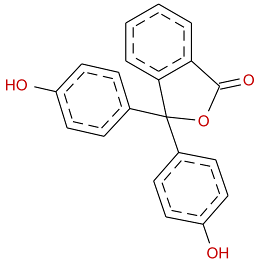	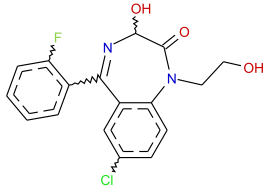
Actual Endpoint	Multiple-Carcinogen	Single-Carcinogen	Multiple-Carcinogen
Predicted Endpoint	Multiple-Carcinogen	Single-Carcinogen	Multiple-Carcinogen
Distance	0.473	0.496	0.582
Reference	US FDA (Centre for Drug Eval.& Res./Off. Testing & Res.) Sept. 1997	US FDA (Centre for Drug Eval.& Res./Off. Testing & Res.) Sept. 1997	US FDA (Centre for Drug Eval.& Res./Off. Testing & Res.) Sept. 1997

Model Prediction

Prediction: Multiple-Carcinogen
Probability: 0.535
Enrichment: 1.43
Bayesian Score: 1.8
Mahalanobis Distance: 10.4
Mahalanobis Distance p-value: 0.0317

Prediction: Positive if the Bayesian score is above the estimated best cutoff value from minimizing the false positive and false negative rate.
Probability: The esimated probability that the sample is in the positive category. This assumes that the Bayesian score follows a normal distribution and is different from the prediction using a cutoff.
Enrichment: An estimate of enrichment, that is, the increased likelihood (versus random) of this sample being in the category.
Bayesian Score: The standard Laplacian-modified Bayesian score.
Mahalanobis Distance: The Mahalanobis distance (MD) is the distance to the center of the training data. The larger the MD, the less trustworthy the prediction.
Mahalanobis Distance p-value: The p-value gives the fraction of training data with an MD greater than or equal to the one for the given sample, assuming normally distributed data. The smaller the p-value, the less trustworthy the prediciton. For highly non-normal X properties (e.g., fingerprints), the MD p-value is wildly inaccurate.

Model Applicability

Unknown features are fingerprint features in the query molecule, but not found or appearing too infrequently in the training set.

1. All properties and OPS components are within expected ranges.

Feature Contribution

Top features for positive contribution

Fingerprint	Bit/Smiles	Feature Structure	Score	Multiple-Carcinogen in training set
SCFP_4	611156666	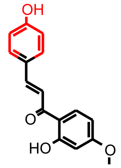 O[c]1:[cH]:[cH]:[*]:[cH]:[cH]:1	0.627	5 out of 7

SCFP_4	387787917	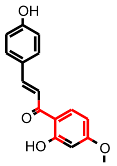 [*][c]1:[*]:[cH]:[c](C=[*]):[cH]:[cH]:1	0.449	6 out of 11
SCFP_4	1237755852	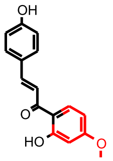 [*][c]1:[*]:[cH]:[cH] :[c](OC):[cH]:1	0.295	5 out of 11
**Top Features for negative contribution**				
**Fingerprint**	**Bit/Smiles**	**Feature Structure**	**Score**	**Multiple-Carcinogen in training set**
SCFP_4	130348166	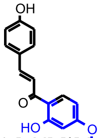 [*]O[c]1:[cH]:[*]:[c] ([*]):[c](O[*]):[cH] :1	-0.489	0 out of 2
SCFP_4	-1971137145	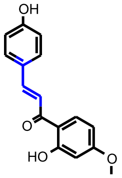 [*]\C=C\[c](:[*]):[*]	-0.489	0 out of 2
SCFP_4	-1977229858	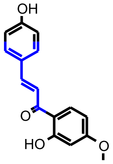 [*]C(=[*])\C=C\[c](:[cH]:[*]):[cH]:[*]	-0.274	0 out of 1

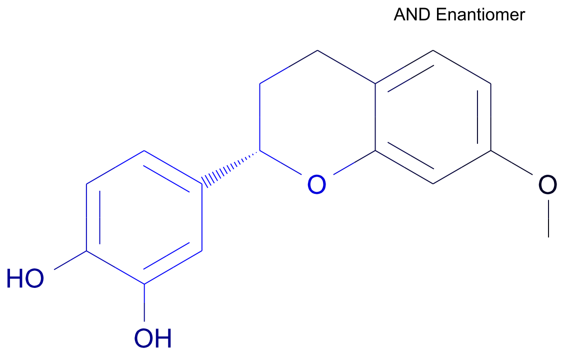

$C_{16}H_{16}O_4$
Molecular Weight: 272.296
ALogP: 3.456
Rotatable Bonds: 2
Acceptors: 4
Donors: 2

Model Prediction

Prediction: Non-Carcinogen
Probability: 0.184
Enrichment: 0.552
Bayesian Score: -8.46
Mahalanobis Distance: 12.7
Mahalanobis Distance p-value: 0.00411

Prediction: Positive if the Bayesian score is above the estimated best cutoff value from minimizing the false positive and false negative rate.
Probability: The esimated probability that the sample is in the positive category. This assumes that the Bayesian score follows a normal distribution and is different from the prediction using a cutoff.
Enrichment: An estimate of enrichment, that is, the increased likelihood (versus random) of this sample being in the category.
Bayesian Score: The standard Laplacian-modified Bayesian score.
Mahalanobis Distance: The Mahalanobis distance (MD) is the distance to the center of the training data. The larger the MD, the less trustworthy the prediction.
Mahalanobis Distance p-value: The p-value gives the fraction of training data with an MD greater than or equal to the one for the given sample, assuming normally distributed data. The smaller the p-value, the less trustworthy the prediciton. For highly non-normal X properties (e.g., fingerprints), the MD p-value is wildly inaccurate.

Structural Similar Compounds

Name	Phenolphthalein	Diflunisal	Oxazepam
Structure	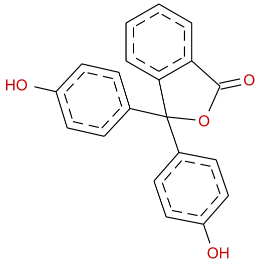	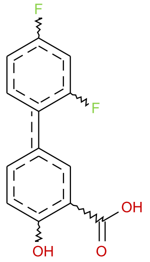	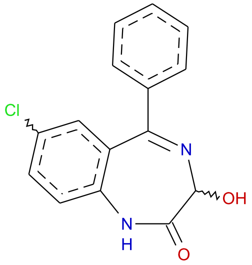
Actual Endpoint	Carcinogen	Non-Carcinogen	Carcinogen
Predicted Endpoint	Carcinogen	Non-Carcinogen	Non-Carcinogen
Distance	0.533	0.564	0.601
Reference	US FDA (Centre for Drug Eval.& Res./Off. Testing & Res.) Sept. 1997	US FDA (Centre for Drug Eval.& Res./Off. Testing & Res.) Sept. 1997	US FDA (Centre for Drug Eval.& Res./Off. Testing & Res.) Sept. 1997

Model Applicability

Unknown features are fingerprint features in the query molecule, but not found or appearing too infrequently in the training set.

1. All properties and OPS components are within expected ranges.

Feature Contribution

Top features for positive contribution

Fingerprint	Bit/Smiles	Feature Structure	Score	Carcinogen in training set
SCFP_6	1237755852	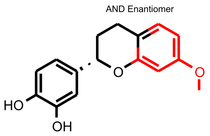 [*][c]1:[*]:[cH]:[cH]:[c](OC):[cH]:1	0.122	11 out of 30

SCFP_6	1238198777	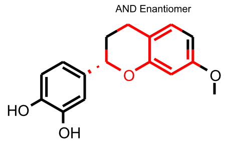<br>[*][C@@H]1[*]C[c]2:[cH]:[*]:[c]([*]):[cH]:[c]:2O1	0.115	3 out of 8
SCFP_6	-1849867720	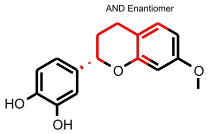<br>[*][C@H]1[*][c](:[*]):[c](CC1):[cH]:[*]	0.111	8 out of 22

Top Features for negative contribution

**Fingerprint**	**Bit/Smiles**	**Feature Structure**	**Score**	**Carcinogen in training set**
SCFP_6	1702724181	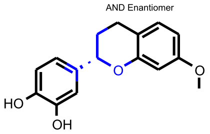<br>[*]C[C@H](O[*])[c](:[*]):[*]	-1.22	1 out of 18
SCFP_6	1578545183	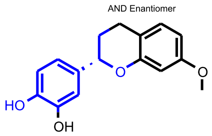<br>[*]C[C@H](O[*])[c]1:[cH]:[cH]:[c](O):[c]([*]):[cH]:1	-1.07	0 out of 6
SCFP_6	-561151481	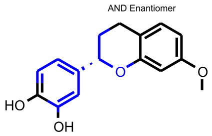<br>[*]C[C@H](O[*])[c]1:[cH]:[cH]:[*]:[c]([*]):[cH]:1	-1.07	1 out of 15

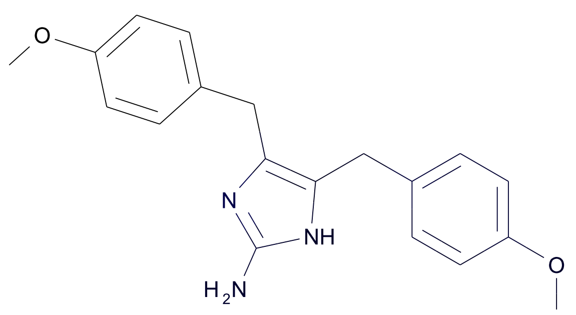

$C_{19}H_{21}N_3O_2$
Molecular Weight: 323.389
ALogP: 3.305
Rotatable Bonds: 6
Acceptors: 4
Donors: 2

Structural Similar Compounds

Name	Bunolol	Metoclopramide	Omeprazole
Structure	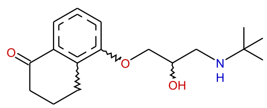	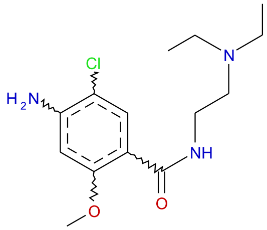	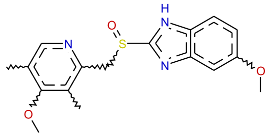
Actual Endpoint	Carcinogen	Non-Carcinogen	Carcinogen
Predicted Endpoint	Carcinogen	Non-Carcinogen	Carcinogen
Distance	0.562	0.591	0.596
Reference	US FDA (Centre for Drug Eval.& Res./Off. Testing & Res.) Sept. 1997	US FDA (Centre for Drug Eval.& Res./Off. Testing & Res.) Sept. 1997	US FDA (Centre for Drug Eval.& Res./Off. Testing & Res.) Sept. 1997

Model Prediction

Prediction: Non-Carcinogen
Probability: 0.304
Enrichment: 0.909
Bayesian Score: -1.97
Mahalanobis Distance: 10.6
Mahalanobis Distance p-value: 0.374

Prediction: Positive if the Bayesian score is above the estimated best cutoff value from minimizing the false positive and false negative rate.
Probability: The esimated probability that the sample is in the positive category. This assumes that the Bayesian score follows a normal distribution and is different from the prediction using a cutoff.
Enrichment: An estimate of enrichment, that is, the increased likelihood (versus random) of this sample being in the category.
Bayesian Score: The standard Laplacian-modified Bayesian score.
Mahalanobis Distance: The Mahalanobis distance (MD) is the distance to the center of the training data. The larger the MD, the less trustworthy the prediction.
Mahalanobis Distance p-value: The p-value gives the fraction of training data with an MD greater than or equal to the one for the given sample, assuming normally distributed data. The smaller the p-value, the less trustworthy the prediciton. For highly non-normal X properties (e.g., fingerprints), the MD p-value is wildly inaccurate.

Model Applicability

Unknown features are fingerprint features in the query molecule, but not found or appearing too infrequently in the training set.

1. All properties and OPS components are within expected ranges.

Feature Contribution

Top features for positive contribution				
**Fingerprint**	**Bit/Smiles**	**Feature Structure**	**Score**	**Carcinogen in training set**
SCFP_6	384920865	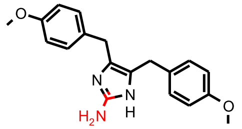 [*]:[c](:[*])N	0.322	11 out of 24

SCFP_6	8	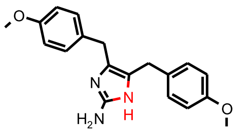 [*]:[nH]:[*]	0.24	39 out of 95
SCFP_6	2109374332	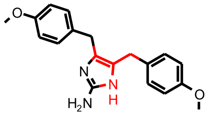 [*]C[c]1:[nH]:[*]:[*]:[c]:1[*]	0.217	11 out of 27

Top Features for negative contribution

Fingerprint	Bit/Smiles	Feature Structure	Score	Carcinogen in training set
SCFP_6	-502559736	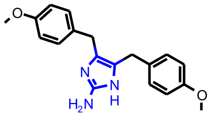 [*][c]1:[nH]:[c](N):n:[c]:1[*]	-0.674	0 out of 3
SCFP_6	649648475	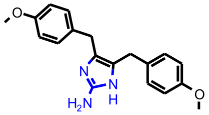 N[c]1:[nH]:[*]:[*]:n:1	-0.48	2 out of 12
SCFP_6	1287669168	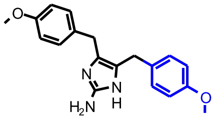 [*][c]1:[cH]:[cH]:[c](OC):[cH]:[cH]:1	-0.38	1 out of 6

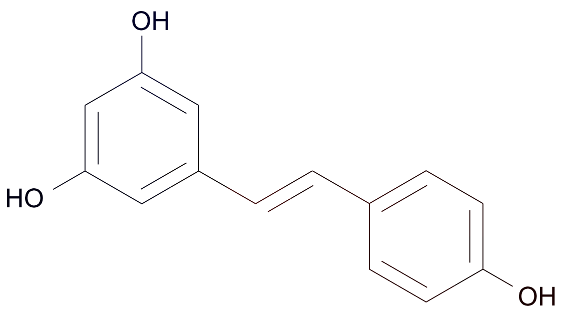

$C_{14}H_{12}O_3$
Molecular Weight: 228.243
ALogP: 3.09
Rotatable Bonds: 2
Acceptors: 3
Donors: 3

Model Prediction

Prediction: Carcinogen
Probability: 0.361
Enrichment: 1.08
Bayesian Score: 0.147
Mahalanobis Distance: 8.28
Mahalanobis Distance p-value: 0.996

Prediction: Positive if the Bayesian score is above the estimated best cutoff value from minimizing the false positive and false negative rate.
Probability: The esimated probability that the sample is in the positive category. This assumes that the Bayesian score follows a normal distribution and is different from the prediction using a cutoff.
Enrichment: An estimate of enrichment, that is, the increased likelihood (versus random) of this sample being in the category.
Bayesian Score: The standard Laplacian-modified Bayesian score.
Mahalanobis Distance: The Mahalanobis distance (MD) is the distance to the center of the training data. The larger the MD, the less trustworthy the prediction.
Mahalanobis Distance p-value: The p-value gives the fraction of training data with an MD greater than or equal to the one for the given sample, assuming normally distributed data. The smaller the p-value, the less trustworthy the prediciton. For highly non-normal X properties (e.g., fingerprints), the MD p-value is wildly inaccurate.

Structural Similar Compounds

Name	Diflunisal	Dienestrol	Guanabenz
Structure	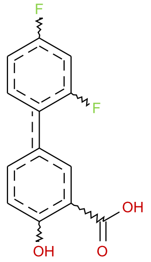	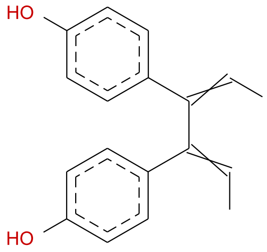	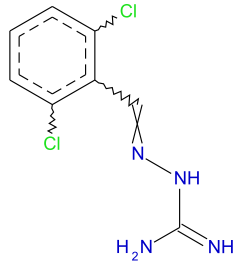
Actual Endpoint	Non-Carcinogen	Non-Carcinogen	Non-Carcinogen
Predicted Endpoint	Non-Carcinogen	Non-Carcinogen	Non-Carcinogen
Distance	0.573	0.612	0.621
Reference	US FDA (Centre for Drug Eval.& Res./Off. Testing & Res.) Sept. 1997	US FDA (Centre for Drug Eval.& Res./Off. Testing & Res.) Sept. 1997	US FDA (Centre for Drug Eval.& Res./Off. Testing & Res.) Sept. 1997

Model Applicability

Unknown features are fingerprint features in the query molecule, but not found or appearing too infrequently in the training set.

1. All properties and OPS components are within expected ranges.

Feature Contribution

Top features for positive contribution

Fingerprint	Bit/Smiles	Feature Structure	Score	Carcinogen in training set
SCFP_6	1958008606	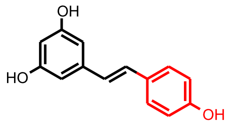 [*][c]1:[cH]:[cH]:[c](O):[cH]:[cH]:1	0.536	4 out of 6

SCFP_6	-1971137145	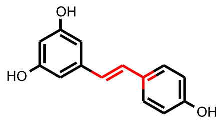 [*]\C=C\[c](:[*]):[*]	0.434	5 out of 9
SCFP_6	392579710	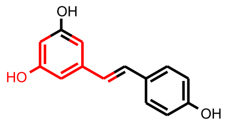 [*]=C[c]1:[cH]:[*]:[cH]:[c](O):[cH]:1	0.425	2 out of 3

Top Features for negative contribution

Fingerprint	Bit/Smiles	Feature Structure	Score	Carcinogen in training set
SCFP_6	-2010583826	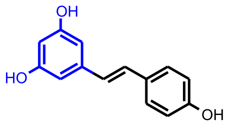 [*][c]1:[cH]:[c](O):[cH]:[c](O):[cH]:1	-0.496	0 out of 2
SCFP_6	130348166	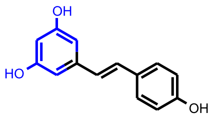 [*]O[c]1:[cH]:[*]:[c]([*]):[c](O[*]):[cH]:1	-0.264	1 out of 5
SCFP_6	-2056718782	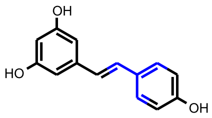 [*]=C[c](:[cH]:[*]):[cH]:[*]	-0.219	23 out of 90

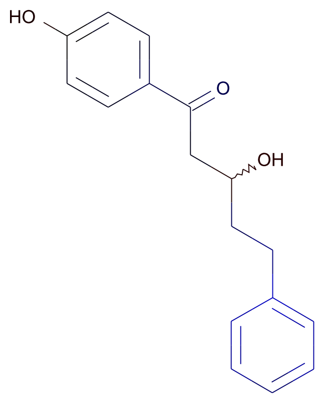

$C_{17}H_{18}O_3$
Molecular Weight: 270.323
ALogP: 3.293
Rotatable Bonds: 6
Acceptors: 3
Donors: 2

Structural Similar Compounds

Name	Bunolol	Penbutalol	Propranolol
Structure	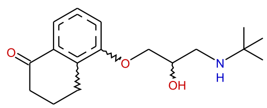	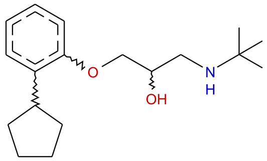	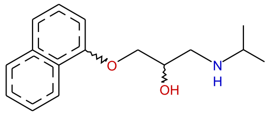
Actual Endpoint	Carcinogen	Non-Carcinogen	Non-Carcinogen
Predicted Endpoint	Carcinogen	Non-Carcinogen	Non-Carcinogen
Distance	0.493	0.541	0.543
Reference	US FDA (Centre for Drug Eval.& Res./Off. Testing & Res.) Sept. 1997	US FDA (Centre for Drug Eval.& Res./Off. Testing & Res.) Sept. 1997	US FDA (Centre for Drug Eval.& Res./Off. Testing & Res.) Sept. 1997

Model Prediction

Prediction: Non-Carcinogen
Probability: 0.256
Enrichment: 0.767
Bayesian Score: -4.09
Mahalanobis Distance: 11.4
Mahalanobis Distance p-value: 0.103

Prediction: Positive if the Bayesian score is above the estimated best cutoff value from minimizing the false positive and false negative rate.
Probability: The esimated probability that the sample is in the positive category. This assumes that the Bayesian score follows a normal distribution and is different from the prediction using a cutoff.
Enrichment: An estimate of enrichment, that is, the increased likelihood (versus random) of this sample being in the category.
Bayesian Score: The standard Laplacian-modified Bayesian score.
Mahalanobis Distance: The Mahalanobis distance (MD) is the distance to the center of the training data. The larger the MD, the less trustworthy the prediction.
Mahalanobis Distance p-value: The p-value gives the fraction of training data with an MD greater than or equal to the one for the given sample, assuming normally distributed data. The smaller the p-value, the less trustworthy the prediciton. For highly non-normal X properties (e.g., fingerprints), the MD p-value is wildly inaccurate.

Model Applicability

Unknown features are fingerprint features in the query molecule, but not found or appearing too infrequently in the training set.

1. All properties and OPS components are within expected ranges.

Feature Contribution

Top features for positive contribution

Fingerprint	Bit/Smiles	Feature Structure	Score	Carcinogen in training set
SCFP_6	-662060434	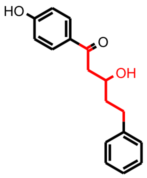 [*]CCC(O)CC(=[*])[*]	0.784	4 out of 4

SCFP_6	1958008606	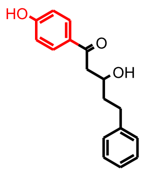 [*][c]1:[cH]:[cH]:[c](O):[cH]:[cH]:1	0.536	4 out of 6
SCFP_6	-1272768868	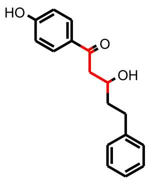 [*]C([*])CC(=[*])[*]	0.242	26 out of 63

Top Features for negative contribution

Fingerprint	Bit/Smiles	Feature Structure	Score	Carcinogen in training set
SCFP_6	-1211866396	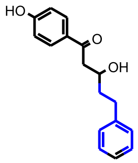 [*]CC[c]1:[cH]:[cH]:[*]:[cH]:[cH]:1	-1.1	2 out of 25
SCFP_6	-542118530	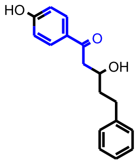 [*]CC(=O)[c]1:[cH]:[cH]:[*]:[cH]:[cH]:1	-0.825	0 out of 4
SCFP_6	-1640858361	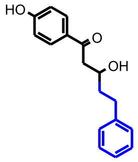 [*]CC[c]1:[cH]:[cH]:[cH]:[cH]:[cH]:1	-0.817	1 out of 11

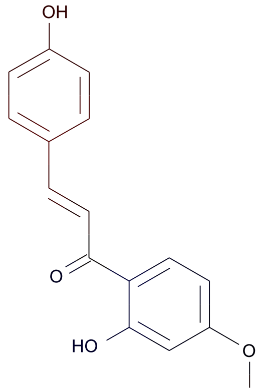

$C_{16}H_{14}O_4$
Molecular Weight: 270.28
ALogP: 3.201
Rotatable Bonds: 4
Acceptors: 4
Donors: 2

Model Prediction

Prediction: Carcinogen
Probability: 0.371
Enrichment: 1.11
Bayesian Score: 0.51
Mahalanobis Distance: 11.7
Mahalanobis Distance p-value: 0.0599

Prediction: Positive if the Bayesian score is above the estimated best cutoff value from minimizing the false positive and false negative rate.
Probability: The esimated probability that the sample is in the positive category. This assumes that the Bayesian score follows a normal distribution and is different from the prediction using a cutoff.
Enrichment: An estimate of enrichment, that is, the increased likelihood (versus random) of this sample being in the category.
Bayesian Score: The standard Laplacian-modified Bayesian score.
Mahalanobis Distance: The Mahalanobis distance (MD) is the distance to the center of the training data. The larger the MD, the less trustworthy the prediction.
Mahalanobis Distance p-value: The p-value gives the fraction of training data with an MD greater than or equal to the one for the given sample, assuming normally distributed data. The smaller the p-value, the less trustworthy the prediciton. For highly non-normal X properties (e.g., fingerprints), the MD p-value is wildly inaccurate.

Structural Similar Compounds

Name	Cytembena	Mebendazole	Phenolphthalein
Structure	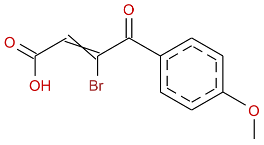	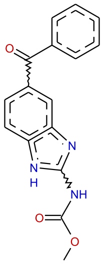	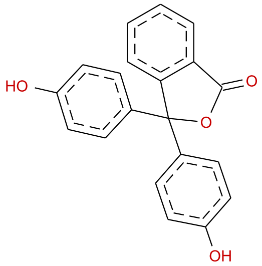
Actual Endpoint	Carcinogen	Non-Carcinogen	Carcinogen
Predicted Endpoint	Carcinogen	Non-Carcinogen	Carcinogen
Distance	0.506	0.508	0.556
Reference	US FDA (Centre for Drug Eval.& Res./Off. Testing & Res.) Sept. 1997	US FDA (Centre for Drug Eval.& Res./Off. Testing & Res.) Sept. 1997	US FDA (Centre for Drug Eval.& Res./Off. Testing & Res.) Sept. 1997

Model Applicability

Unknown features are fingerprint features in the query molecule, but not found or appearing too infrequently in the training set.

1. All properties and OPS components are within expected ranges.

Feature Contribution

Top features for positive contribution

Fingerprint	Bit/Smiles	Feature Structure	Score	Carcinogen in training set
SCFP_6	1958008606	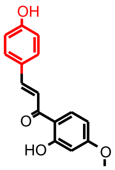 [*][c]1:[cH]:[cH]:[c](O):[cH]:[cH]:1	0.536	4 out of 6

SCFP_6	-1971137145	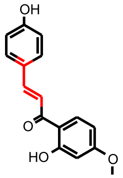<br>[*]\C=C\[c](:[*]):[*]	0.434	5 out of 9
SCFP_6	90200732	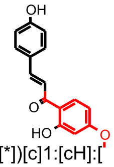<br>[*]C(=[*])[c]1:[cH]:[cH]:[c](OC):[cH]:[c]:1[*]	0.425	2 out of 3

Top Features for negative contribution

Fingerprint	Bit/Smiles	Feature Structure	Score	Carcinogen in training set
SCFP_6	964001357	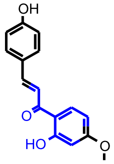<br>[*]=CC(=O)[c]1:[cH]:[cH]:[*]:[cH]:[c]:1O	-0.278	0 out of 1
SCFP_6	-617610981	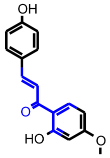<br>[*]\C=C\C(=O)[c](:[cH]:[*]):[c]([*]):[*]	-0.278	0 out of 1
SCFP_6	130348166	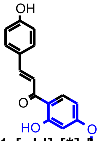<br>[*]O[c]1:[cH]:[*]:[c]([*]):[c](O[*]):[cH]:1	-0.264	1 out of 5

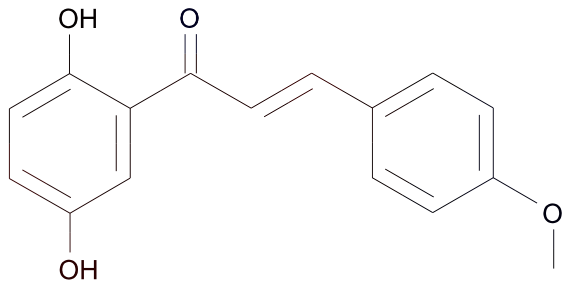

$C_{16}H_{14}O_4$
Molecular Weight: 270.28
ALogP: 3.201
Rotatable Bonds: 4
Acceptors: 4
Donors: 2

Model Prediction

Prediction: Carcinogen
Probability: 0.371
Enrichment: 1.11
Bayesian Score: 0.5
Mahalanobis Distance: 11.7
Mahalanobis Distance p-value: 0.0599

Prediction: Positive if the Bayesian score is above the estimated best cutoff value from minimizing the false positive and false negative rate.
Probability: The esimated probability that the sample is in the positive category. This assumes that the Bayesian score follows a normal distribution and is different from the prediction using a cutoff.
Enrichment: An estimate of enrichment, that is, the increased likelihood (versus random) of this sample being in the category.
Bayesian Score: The standard Laplacian-modified Bayesian score.
Mahalanobis Distance: The Mahalanobis distance (MD) is the distance to the center of the training data. The larger the MD, the less trustworthy the prediction.
Mahalanobis Distance p-value: The p-value gives the fraction of training data with an MD greater than or equal to the one for the given sample, assuming normally distributed data. The smaller the p-value, the less trustworthy the prediciton. For highly non-normal X properties (e.g., fingerprints), the MD p-value is wildly inaccurate.

Structural Similar Compounds

Name	Cytembena	Mebendazole	Phenolphthalein
Structure	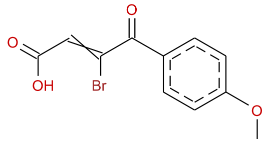	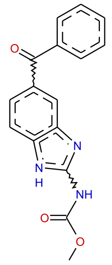	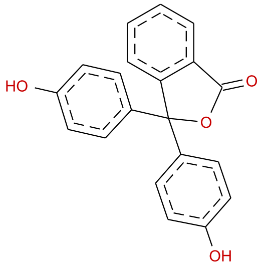
Actual Endpoint	Carcinogen	Non-Carcinogen	Carcinogen
Predicted Endpoint	Carcinogen	Non-Carcinogen	Carcinogen
Distance	0.497	0.509	0.567
Reference	US FDA (Centre for Drug Eval.& Res./Off. Testing & Res.) Sept. 1997	US FDA (Centre for Drug Eval.& Res./Off. Testing & Res.) Sept. 1997	US FDA (Centre for Drug Eval.& Res./Off. Testing & Res.) Sept. 1997

Model Applicability

Unknown features are fingerprint features in the query molecule, but not found or appearing too infrequently in the training set.

1. All properties and OPS components are within expected ranges.

Feature Contribution

Top features for positive contribution

Fingerprint	Bit/Smiles	Feature Structure	Score	Carcinogen in training set
SCFP_6	-1971137145	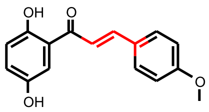 [*]\C=C\[c](:[*]):[*]	0.434	5 out of 9

SCFP_6	392579710	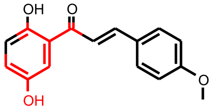 [*]=C[c]1:[cH]:[*]:[cH]:[c](O):[cH]:1	0.425	2 out of 3
SCFP_6	-1977229858	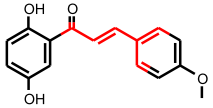 [*]C(=[*])\C=C\[c](:[cH]:[*]):[cH]:[*]	0.425	2 out of 3

Top Features for negative contribution

Fingerprint	Bit/Smiles	Feature Structure	Score	Carcinogen in training set
SCFP_6	1287669168	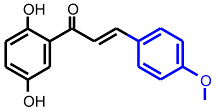 [*][c]1:[cH]:[cH]:[c](OC):[cH]:[cH]:1	-0.38	1 out of 6
SCFP_6	964001357	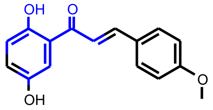 [*]=CC(=O)[c]1:[cH]:[cH]:[*]:[cH]:[c]:1O	-0.278	0 out of 1
SCFP_6	-617610981	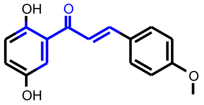 [*]\C=C\C(=O)[c](:[cH]:[*]):[c]([*]):[*]	-0.278	0 out of 1

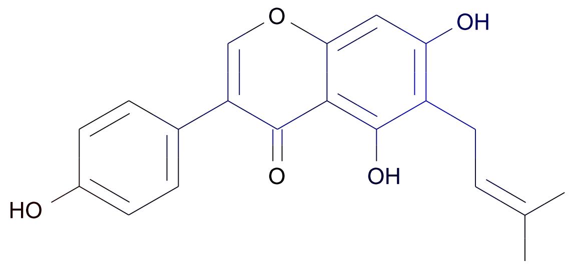

$C_{20}H_{18}O_5$
Molecular Weight: 338.354
ALogP: 3.997
Rotatable Bonds: 3
Acceptors: 5
Donors: 3

Model Prediction

Prediction: Non-Carcinogen
Probability: 0.23
Enrichment: 0.688
Bayesian Score: -5.48
Mahalanobis Distance: 12.8
Mahalanobis Distance p-value: 0.00302

Prediction: Positive if the Bayesian score is above the estimated best cutoff value from minimizing the false positive and false negative rate.
Probability: The esimated probability that the sample is in the positive category. This assumes that the Bayesian score follows a normal distribution and is different from the prediction using a cutoff.
Enrichment: An estimate of enrichment, that is, the increased likelihood (versus random) of this sample being in the category.
Bayesian Score: The standard Laplacian-modified Bayesian score.
Mahalanobis Distance: The Mahalanobis distance (MD) is the distance to the center of the training data. The larger the MD, the less trustworthy the prediction.
Mahalanobis Distance p-value: The p-value gives the fraction of training data with an MD greater than or equal to the one for the given sample, assuming normally distributed data. The smaller the p-value, the less trustworthy the prediciton. For highly non-normal X properties (e.g., fingerprints), the MD p-value is wildly inaccurate.

Structural Similar Compounds

Name	Phenolphthalein	Torsemide	Niclosamide
Structure	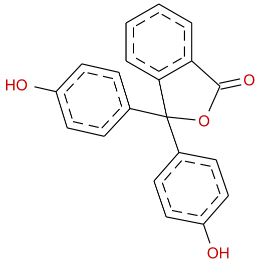	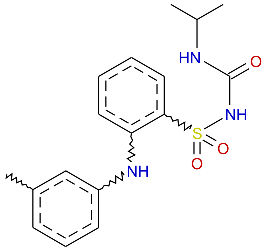	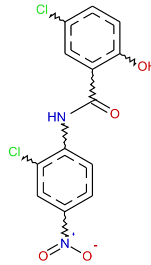
Actual Endpoint	Carcinogen	Carcinogen	Non-Carcinogen
Predicted Endpoint	Carcinogen	Carcinogen	Non-Carcinogen
Distance	0.583	0.630	0.634
Reference	US FDA (Centre for Drug Eval.& Res./Off. Testing & Res.) Sept. 1997	US FDA (Centre for Drug Eval.& Res./Off. Testing & Res.) Sept. 1997	US FDA (Centre for Drug Eval.& Res./Off. Testing & Res.) Sept. 1997

Model Applicability

Unknown features are fingerprint features in the query molecule, but not found or appearing too infrequently in the training set.

1. All properties and OPS components are within expected ranges.

Feature Contribution

Top features for positive contribution

Fingerprint	Bit/Smiles	Feature Structure	Score	Carcinogen in training set
SCFP_6	1958008606	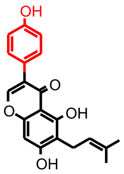 [*][c]1:[cH]:[cH]:[c](O):[cH]:[cH]:1	0.536	4 out of 6

SCFP_6	55434585	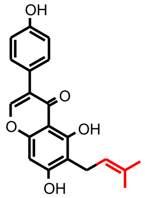 [*]C=C(C)C	0.331	12 out of 26
SCFP_6	1157879834	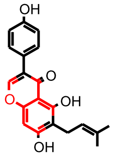 [*][c]1:[*]:[c]([*]):[c]2C(=[*])[*]=CO[c]:2:[cH]:1	0.198	1 out of 2

Top Features for negative contribution

Fingerprint	Bit/Smiles	Feature Structure	Score	Carcinogen in training set
SCFP_6	1798334293	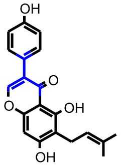 [*]\C=C(/C(=[*])[*])\[c](:[*]):[*]	-0.674	0 out of 3
SCFP_6	-1931277081	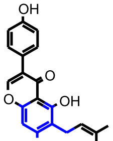 [*]C[c]1:[c]([*]):[*]:[c]([*]):[cH]:[c]:1O	-0.674	0 out of 3
SCFP_6	74606309	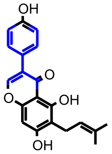 [*]\C=C(/C(=[*])[*])\[c]1:[cH]:[cH]:[*]:[cH]:[cH]:1	-0.674	0 out of 3

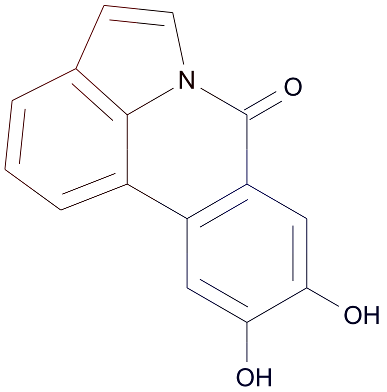

$C_{15}H_9NO_3$
Molecular Weight: 251.237
ALogP: 2.982
Rotatable Bonds: 0
Acceptors: 3
Donors: 2

Model Prediction

Prediction: Non-Carcinogen
Probability: 0.35
Enrichment: 1.05
Bayesian Score: -0.221
Mahalanobis Distance: 14.4
Mahalanobis Distance p-value: 1e-005

Prediction: Positive if the Bayesian score is above the estimated best cutoff value from minimizing the false positive and false negative rate.
Probability: The esimated probability that the sample is in the positive category. This assumes that the Bayesian score follows a normal distribution and is different from the prediction using a cutoff.
Enrichment: An estimate of enrichment, that is, the increased likelihood (versus random) of this sample being in the category.
Bayesian Score: The standard Laplacian-modified Bayesian score.
Mahalanobis Distance: The Mahalanobis distance (MD) is the distance to the center of the training data. The larger the MD, the less trustworthy the prediction.
Mahalanobis Distance p-value: The p-value gives the fraction of training data with an MD greater than or equal to the one for the given sample, assuming normally distributed data. The smaller the p-value, the less trustworthy the prediciton. For highly non-normal X properties (e.g., fingerprints), the MD p-value is wildly inaccurate.

Structural Similar Compounds

Name	Danthron	Diflunisal	Oxazepam
Structure	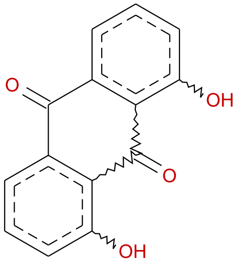	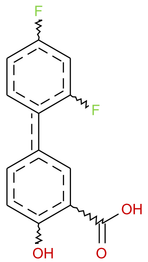	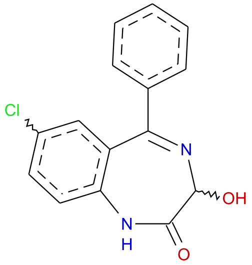
Actual Endpoint	Carcinogen	Non-Carcinogen	Carcinogen
Predicted Endpoint	Carcinogen	Non-Carcinogen	Non-Carcinogen
Distance	0.555	0.555	0.561
Reference	US FDA (Centre for Drug Eval.& Res./Off. Testing & Res.) Sept. 1997	US FDA (Centre for Drug Eval.& Res./Off. Testing & Res.) Sept. 1997	US FDA (Centre for Drug Eval.& Res./Off. Testing & Res.) Sept. 1997

Model Applicability

Unknown features are fingerprint features in the query molecule, but not found or appearing too infrequently in the training set.

1. All properties and OPS components are within expected ranges.

Feature Contribution

Top features for positive contribution

Fingerprint	Bit/Smiles	Feature Structure	Score	Carcinogen in training set
SCFP_6	1401386208	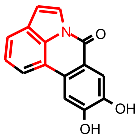 [*]n1:[cH]:[cH]:[c]2:[cH]:[cH]:[*]:[c]([*]):[c]:1:2	0.603	2 out of 2

SCFP_6	-1379673609	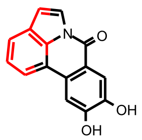 [*]1:[*]:[c]2:[*]:[cH]:[cH]:[cH]:[c]:2:[cH]:1	0.526	11 out of 19
SCFP_6	392579710	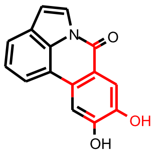 [*]=C[c]1:[cH]:[*]:[cH]:[c](O):[cH]:1	0.425	2 out of 3
**Top Features for negative contribution**				
**Fingerprint**	**Bit/Smiles**	**Feature Structure**	**Score**	**Carcinogen in training set**
SCFP_6	2116304939	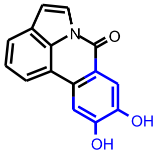 O[c]1:[cH]:[*]:[cH]:[cH]:[c]:1O	-0.825	0 out of 4
SCFP_6	-758932618	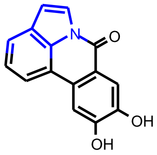 [*]n1:[cH]:[cH]:[c](:[cH]:[*]):[c]:1:[*]	-0.278	0 out of 1
SCFP_6	-314422371	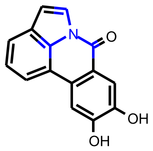 [*]C(=[*])n1:[cH]:[*]:[*]:[c]:1:[*]	-0.278	0 out of 1

remdesivir

TOPKAT_Rat_Male_FDA_None_vs_Carcinogen

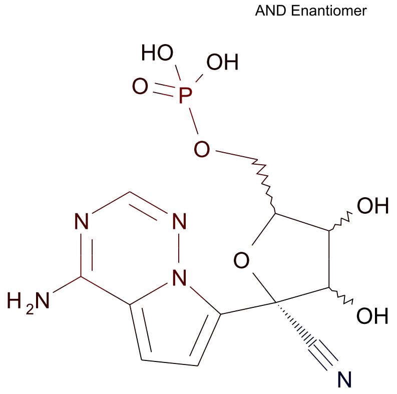

$C_{12}H_{14}N_5O_7P$

Molecular Weight: 371.24262

ALogP: -1.502

Rotatable Bonds: 4

Acceptors: 11

Donors: 5

Model Prediction

Prediction: Carcinogen

Probability: 0.481

Enrichment: 1.44

Bayesian Score: 3.82

Mahalanobis Distance: 14.1

Mahalanobis Distance p-value: 3.32e-005

Prediction: Positive if the Bayesian score is above the estimated best cutoff value from minimizing the false positive and false negative rate.
Probability: The esimated probability that the sample is in the positive category. This assumes that the Bayesian score follows a normal distribution and is different from the prediction using a cutoff.
Enrichment: An estimate of enrichment, that is, the increased likelihood (versus random) of this sample being in the category.
Bayesian Score: The standard Laplacian-modified Bayesian score.
Mahalanobis Distance: The Mahalanobis distance (MD) is the distance to the center of the training data. The larger the MD, the less trustworthy the prediction.
Mahalanobis Distance p-value: The p-value gives the fraction of training data with an MD greater than or equal to the one for the given sample, assuming normally distributed data. The smaller the p-value, the less trustworthy the prediciton. For highly non-normal X properties (e.g., fingerprints), the MD p-value is wildly inaccurate.

Structural Similar Compounds

Name	Streptozocin	Famotidine	Tetracycline
Structure	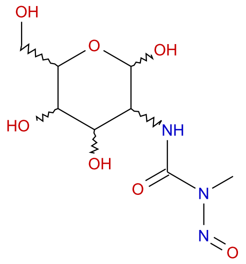	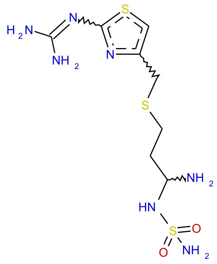	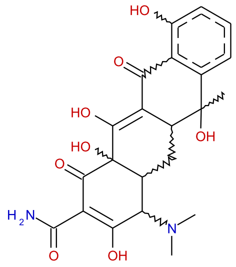
Actual Endpoint	Carcinogen	Non-Carcinogen	Non-Carcinogen
Predicted Endpoint	Carcinogen	Non-Carcinogen	Non-Carcinogen
Distance	0.789	0.850	0.856
Reference	US FDA (Centre for Drug Eval.& Res./Off. Testing & Res.) Sept. 1997	US FDA (Centre for Drug Eval.& Res./Off. Testing & Res.) Sept. 1997	US FDA (Centre for Drug Eval.& Res./Off. Testing & Res.) Sept. 1997

Model Applicability

Unknown features are fingerprint features in the query molecule, but not found or appearing too infrequently in the training set.

1. All properties and OPS components are within expected ranges.

Feature Contribution

Top features for positive contribution

Fingerprint	Bit/Smiles	Feature Structure	Score	Carcinogen in training set
SCFP_6	-1029620989	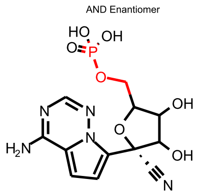 [*]COP(=[*])([*])[*]	0.712	3 out of 3

SCFP_6	1245795878	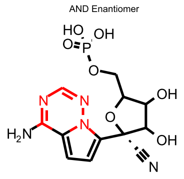 [*][c]1:[*]:n(:[*]):n :[cH]:n:1	0.603	2 out of 2
SCFP_6	149212520	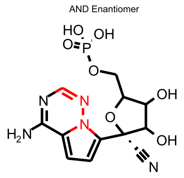 [*]:[cH]:n:n(:[*]):[*]	0.543	9 out of 15

Top Features for negative contribution

Fingerprint	Bit/Smiles	Feature Structure	Score	Carcinogen in training set
SCFP_6	-1019297400	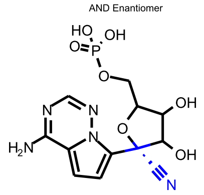 [*]C([*])([*])C#N	-0.674	0 out of 3
SCFP_6	194135988	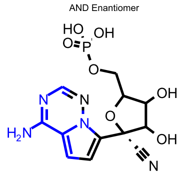 N[c]1:n:[cH]:[*]:n2:[*]:[*]:[cH]:[c]:1:2	-0.278	0 out of 1
SCFP_6	-424515134	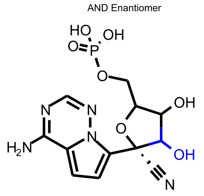 [*]C([*])O	-0.157	30 out of 110

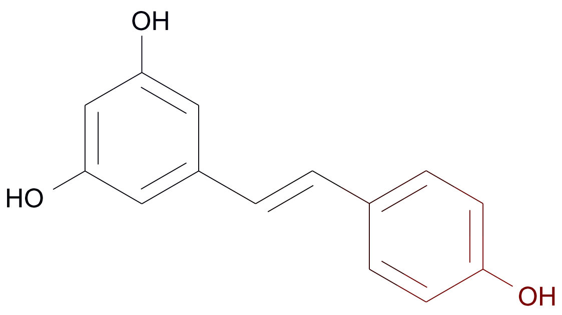

$C_{14}H_{12}O_3$
Molecular Weight: 228.243
ALogP: 3.09
Rotatable Bonds: 2
Acceptors: 3
Donors: 3

Model Prediction

Prediction: Multiple-Carcinogen
Probability: 0.569
Enrichment: 1.37
Bayesian Score: 1.7
Mahalanobis Distance: 7.8
Mahalanobis Distance p-value: 0.771

Prediction: Positive if the Bayesian score is above the estimated best cutoff value from minimizing the false positive and false negative rate.
Probability: The esimated probability that the sample is in the positive category. This assumes that the Bayesian score follows a normal distribution and is different from the prediction using a cutoff.
Enrichment: An estimate of enrichment, that is, the increased likelihood (versus random) of this sample being in the category.
Bayesian Score: The standard Laplacian-modified Bayesian score.
Mahalanobis Distance: The Mahalanobis distance (MD) is the distance to the center of the training data. The larger the MD, the less trustworthy the prediction.
Mahalanobis Distance p-value: The p-value gives the fraction of training data with an MD greater than or equal to the one for the given sample, assuming normally distributed data. The smaller the p-value, the less trustworthy the prediciton. For highly non-normal X properties (e.g., fingerprints), the MD p-value is wildly inaccurate.

Structural Similar Compounds

Name	Danthron	Oxazepam	Phenolphthalein
Structure	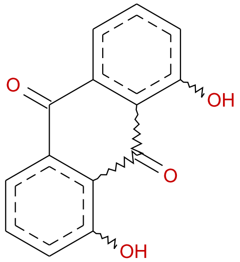	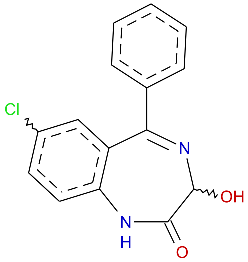	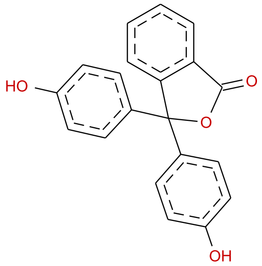
Actual Endpoint	Single-Carcinogen	Single-Carcinogen	Multiple-Carcinogen
Predicted Endpoint	Single-Carcinogen	Single-Carcinogen	Multiple-Carcinogen
Distance	0.684	0.690	0.694
Reference	US FDA (Centre for Drug Eval.& Res./Off. Testing & Res.) Sept. 1997	US FDA (Centre for Drug Eval.& Res./Off. Testing & Res.) Sept. 1997	US FDA (Centre for Drug Eval.& Res./Off. Testing & Res.) Sept. 1997

Model Applicability

Unknown features are fingerprint features in the query molecule, but not found or appearing too infrequently in the training set.

1. All properties and OPS components are within expected ranges.

Feature Contribution

Top features for positive contribution

Fingerprint	Bit/Smiles	Feature Structure	Score	Multiple-Carcinogen in training set
SCFP_8	1958008606	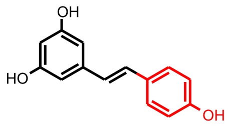 [*][c]1:[cH]:[cH]:[c](O):[cH]:[cH]:1	0.712	4 out of 4

SCFP_8	611156666	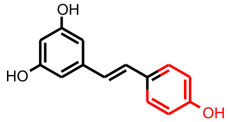 O[c]1:[cH]:[cH]:[*]:[cH]:[cH]:1	0.635	5 out of 6
SCFP_8	1112262477	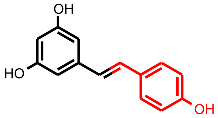 [*]=C[c]1:[cH]:[cH]:[c](O):[cH]:[cH]:1	0.383	1 out of 1
**Top Features for negative contribution**				
**Fingerprint**	**Bit/Smiles**	**Feature Structure**	**Score**	**Multiple-Carcinogen in training set**
SCFP_8	130348166	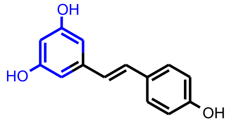 O[c]1:[cH]:[*]:[cH]:[c](O):[cH]:1	-0.31	0 out of 1
SCFP_8	367120510	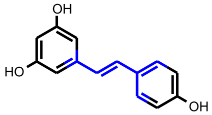 [*]:[cH]:[c](\C=C\[c] (:[*]):[*]):[cH]:[*]	-0.31	0 out of 1
SCFP_8	-2056718782	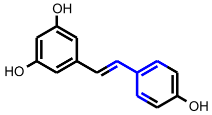 [*]=C[c](:[cH]:[*]):[cH]:[*]	-0.29	6 out of 23

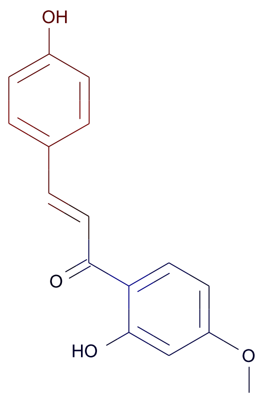

$C_{16}H_{14}O_4$
Molecular Weight: 270.28
ALogP: 3.201
Rotatable Bonds: 4
Acceptors: 4
Donors: 2

Model Prediction

Prediction: Multiple-Carcinogen
Probability: 0.569
Enrichment: 1.37
Bayesian Score: 0.976
Mahalanobis Distance: 11.3
Mahalanobis Distance p-value: 0.0144

Prediction: Positive if the Bayesian score is above the estimated best cutoff value from minimizing the false positive and false negative rate.
Probability: The esimated probability that the sample is in the positive category. This assumes that the Bayesian score follows a normal distribution and is different from the prediction using a cutoff.
Enrichment: An estimate of enrichment, that is, the increased likelihood (versus random) of this sample being in the category.
Bayesian Score: The standard Laplacian-modified Bayesian score.
Mahalanobis Distance: The Mahalanobis distance (MD) is the distance to the center of the training data. The larger the MD, the less trustworthy the prediction.
Mahalanobis Distance p-value: The p-value gives the fraction of training data with an MD greater than or equal to the one for the given sample, assuming normally distributed data. The smaller the p-value, the less trustworthy the prediciton. For highly non-normal X properties (e.g., fingerprints), the MD p-value is wildly inaccurate.

Structural Similar Compounds

Name	Cytembena	Phenolphthalein	Bunolol
Structure	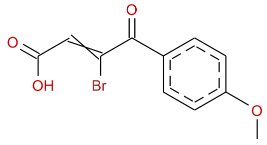	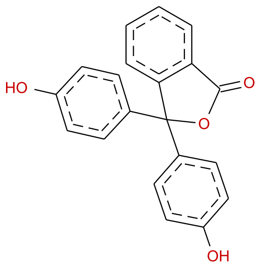	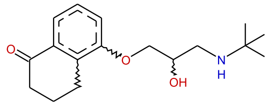
Actual Endpoint	Single-Carcinogen	Multiple-Carcinogen	Single-Carcinogen
Predicted Endpoint	Single-Carcinogen	Multiple-Carcinogen	Single-Carcinogen
Distance	0.578	0.586	0.616
Reference	US FDA (Centre for Drug Eval.& Res./Off. Testing & Res.) Sept. 1997	US FDA (Centre for Drug Eval.& Res./Off. Testing & Res.) Sept. 1997	US FDA (Centre for Drug Eval.& Res./Off. Testing & Res.) Sept. 1997

Model Applicability

Unknown features are fingerprint features in the query molecule, but not found or appearing too infrequently in the training set.

1. All properties and OPS components are within expected ranges.

Feature Contribution

Top features for positive contribution

Fingerprint	Bit/Smiles	Feature Structure	Score	Multiple-Carcinogen in training set
SCFP_8	1958008606	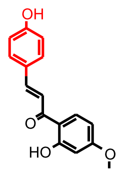 [*][c]1:[cH]:[cH]:[c](O):[cH]:[cH]:1	0.712	4 out of 4

SCFP_8	611156666	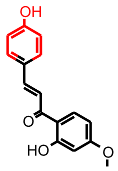 O[c]1:[cH]:[cH]:[*]:[cH]:[cH]:1	0.635	5 out of 6
SCFP_8	-1977229858	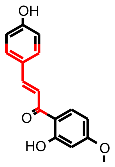 [*]C(=[*])\C=C\[c](:[cH]:[*]):[cH]:[*]	0.553	2 out of 2

Top Features for negative contribution

Fingerprint	Bit/Smiles	Feature Structure	Score	Multiple-Carcinogen in training set
SCFP_8	860600739	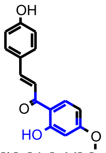 [*]C(=[*])[c]1:[cH]:[*]:[c]([*]):[cH]:[c]:1O	-0.737	0 out of 3
SCFP_8	-1980302127	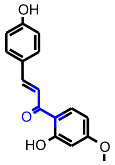 [*]=CC(=O)[c](:[*]):[*]	-0.737	0 out of 3
SCFP_8	90200732	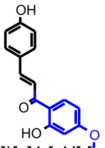 [*]C(=[*])[c]1:[cH]:[cH]:[c](OC):[cH]:[c]:1[*]	-0.546	0 out of 2

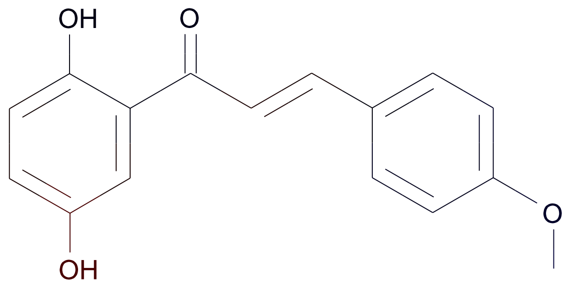

$C_{16}H_{14}O_4$
Molecular Weight: 270.28
ALogP: 3.201
Rotatable Bonds: 4
Acceptors: 4
Donors: 2

Structural Similar Compounds

Name	Cytembena	Phenolphthalein	Bunolol
Structure	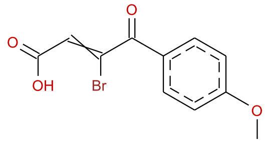	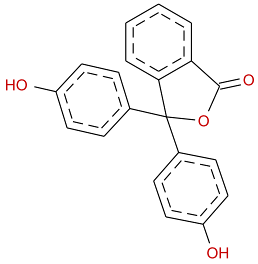	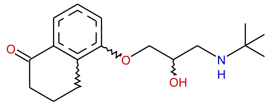
Actual Endpoint	Single-Carcinogen	Multiple-Carcinogen	Single-Carcinogen
Predicted Endpoint	Single-Carcinogen	Multiple-Carcinogen	Single-Carcinogen
Distance	0.569	0.594	0.616
Reference	US FDA (Centre for Drug Eval.& Res./Off. Testing & Res.) Sept. 1997	US FDA (Centre for Drug Eval.& Res./Off. Testing & Res.) Sept. 1997	US FDA (Centre for Drug Eval.& Res./Off. Testing & Res.) Sept. 1997

Model Prediction

Prediction: Single-Carcinogen
Probability: 0.566
Enrichment: 1.37
Bayesian Score: 0.411
Mahalanobis Distance: 11.3
Mahalanobis Distance p-value: 0.0144

Prediction: Positive if the Bayesian score is above the estimated best cutoff value from minimizing the false positive and false negative rate.
Probability: The esimated probability that the sample is in the positive category. This assumes that the Bayesian score follows a normal distribution and is different from the prediction using a cutoff.
Enrichment: An estimate of enrichment, that is, the increased likelihood (versus random) of this sample being in the category.
Bayesian Score: The standard Laplacian-modified Bayesian score.
Mahalanobis Distance: The Mahalanobis distance (MD) is the distance to the center of the training data. The larger the MD, the less trustworthy the prediction.
Mahalanobis Distance p-value: The p-value gives the fraction of training data with an MD greater than or equal to the one for the given sample, assuming normally distributed data. The smaller the p-value, the less trustworthy the prediciton. For highly non-normal X properties (e.g., fingerprints), the MD p-value is wildly inaccurate.

Model Applicability

Unknown features are fingerprint features in the query molecule, but not found or appearing too infrequently in the training set.

1. All properties and OPS components are within expected ranges.

Feature Contribution

Top features for positive contribution

Fingerprint	Bit/Smiles	Feature Structure	Score	Multiple-Carcinogen in training set
SCFP_8	611156666	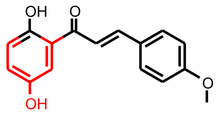 O[c]1:[cH]:[cH]:[*]:[cH]:[cH]:1	0.635	5 out of 6

SCFP_8	-1977229858	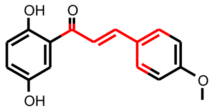<br>[*]C(=[*])\C=C\[c](:[cH]:[*]):[cH]:[*]	0.553	2 out of 2
SCFP_8	1741463142	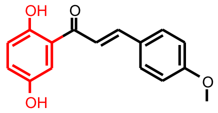<br>[*][c]1:[cH]:[c](O):[cH]:[cH]:[c]:1O	0.383	1 out of 1
**Top Features for negative contribution**				
**Fingerprint**	**Bit/Smiles**	**Feature Structure**	**Score**	**Multiple-Carcinogen in training set**
SCFP_8	860600739	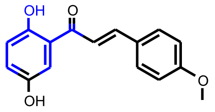<br>[*]C(=[*])[c]1:[cH]:[*]:[c]([*]):[cH]:[c] :1O	-0.737	0 out of 3
SCFP_8	-1980302127	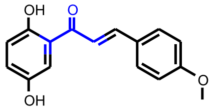<br>[*]=CC(=O)[c](:[*]):[*]	-0.737	0 out of 3
SCFP_8	90200732	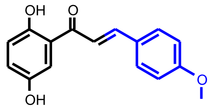<br>[*]C(=[*])[c]1:[cH]:[cH]:[c](OC):[cH]:[c] :1[*]	-0.546	0 out of 2

remdesivir

TOPKAT_Rat_Male_FDA_Single_vs_Multiple

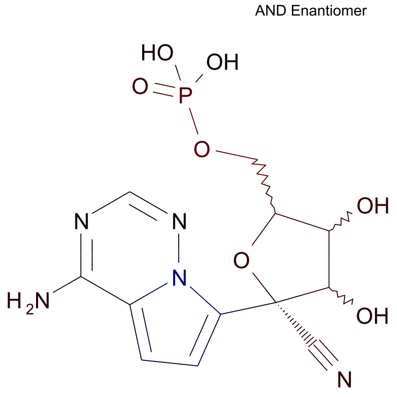

$C_{12}H_{14}N_5O_7P$
Molecular Weight: 371.24262
ALogP: -1.502
Rotatable Bonds: 4
Acceptors: 11
Donors: 5

Model Prediction

Prediction: Multiple-Carcinogen
Probability: 0.556
Enrichment: 1.34
Bayesian Score: 3.52
Mahalanobis Distance: 14
Mahalanobis Distance p-value: 8.72e-005

Prediction: Positive if the Bayesian score is above the estimated best cutoff value from minimizing the false positive and false negative rate.
Probability: The esimated probability that the sample is in the positive category. This assumes that the Bayesian score follows a normal distribution and is different from the prediction using a cutoff.
Enrichment: An estimate of enrichment, that is, the increased likelihood (versus random) of this sample being in the category.
Bayesian Score: The standard Laplacian-modified Bayesian score.
Mahalanobis Distance: The Mahalanobis distance (MD) is the distance to the center of the training data. The larger the MD, the less trustworthy the prediction.
Mahalanobis Distance p-value: The p-value gives the fraction of training data with an MD greater than or equal to the one for the given sample, assuming normally distributed data. The smaller the p-value, the less trustworthy the prediciton. For highly non-normal X properties (e.g., fingerprints), the MD p-value is wildly inaccurate.

Structural Similar Compounds

Name	Streptozocin	Minocycline	Ribavirin
Structure	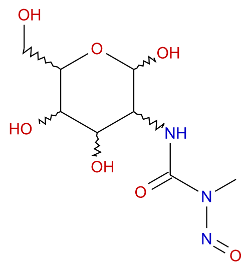	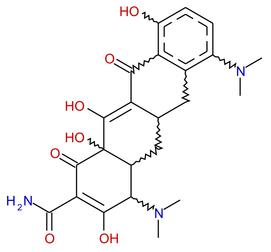	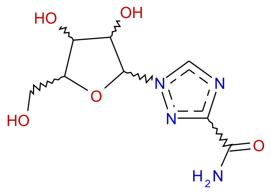
Actual Endpoint	Multiple-Carcinogen	Single-Carcinogen	Multiple-Carcinogen
Predicted Endpoint	Multiple-Carcinogen	Single-Carcinogen	Multiple-Carcinogen
Distance	0.817	0.908	0.929
Reference	US FDA (Centre for Drug Eval.& Res./Off. Testing & Res.) Sept. 1997	US FDA (Centre for Drug Eval.& Res./Off. Testing & Res.) Sept. 1997	US FDA (Centre for Drug Eval.& Res./Off. Testing & Res.) Sept. 1997

Model Applicability

Unknown features are fingerprint features in the query molecule, but not found or appearing too infrequently in the training set.

1. Num_H_Acceptors out of range. Value: 11. Training min, max, mean, SD: 0, 9, 3.8906, 2.196.

Feature Contribution

Top features for positive contribution

Fingerprint	Bit/Smiles	Feature Structure	Score	Multiple-Carcinogen in training set
SCFP_8	-1029620989	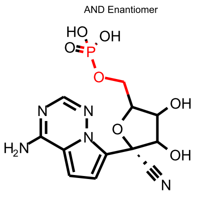 [*]COP(=[*])([*])[*]	0.649	3 out of 3

SCFP_8	2	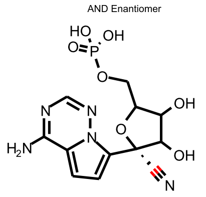 [*]C#[*]	0.584	6 out of 8
SCFP_8	-1486266146	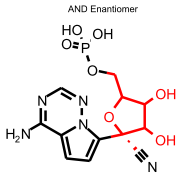 [*]CC1OC([*])([*])C(O)C1O	0.553	2 out of 2
**Top Features for negative contribution**				
**Fingerprint**	**Bit/Smiles**	**Feature Structure**	**Score**	**Multiple-Carcinogen in training set**
SCFP_8	-1381862798	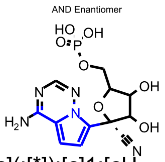 [*][c](:[*]):[c]1:[cH]:[cH]:[c]([*]):n:1: [*]	-0.572	1 out of 7
SCFP_8	1245795878	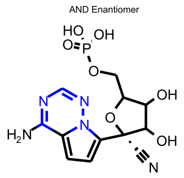 [*][c]1:[*]:n(:[*]):n :[cH]:n:1	-0.546	0 out of 2
SCFP_8	-1375522316	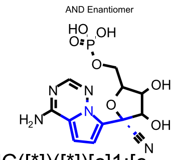 [*]C([*])([*])[c]1:[c H]:[cH]:[c](:[*]):n: 1:[*]	-0.546	0 out of 2

remdesivir

TOPKAT_Skin_Irritancy_Mild_vs_Moderate_Severe

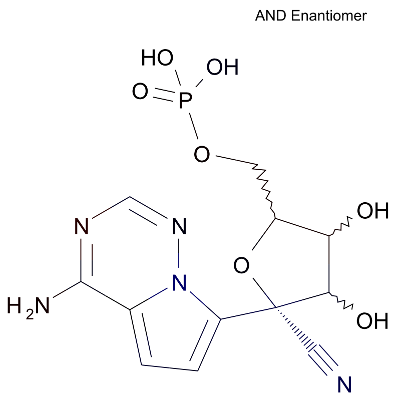

$C_{12}H_{14}N_5O_7P$

Molecular Weight: 371.24262

ALogP: -1.502

Rotatable Bonds: 4

Acceptors: 11

Donors: 5

Structural Similar Compounds

Name	1,3,6-Naphthalenetrisulfonic acid, 7-amino-	2,7-Anthracenedisulfonic acid, 9,10-dihydro-4,5-diamino-9,10-dioxo- 1-hydroxy-, disodium salt	1,5-Naphthalenedisulfonic acid, 2-amino-
Structure	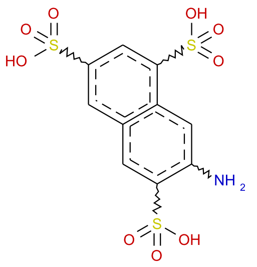	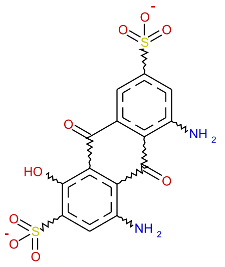	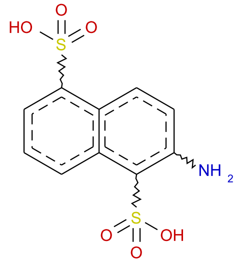
Actual Endpoint	Mild	Mild	Mild
Predicted Endpoint	Mild	Mild	Mild
Distance	0.759	1.033	1.137
Reference	85JCAE "Prehled Prumyslove Toxikologie; Organicke Latky," Marhold, J., Prague , Czechoslovakia, Avicenum, 1986 Volume(issue)/page/year: -,1058,1986	28ZPAK "Sbornik Vysledku Toxixologickeho Vysetreni Latek A Pripravku," Marhold d, J.V., Institut Pro Vychovu Vedoucicn Pracovniku Chemickeho Prumyclu Praha, Cz echoslovakia, 1972 Volume(issue)/page/year: -,239,1	85JCAE "Prehled Prumyslove Toxikologie; Organicke Latky," Marhold, J., Prague , Czechoslovakia, Avicenum, 1986 Volume(issue)/page/year: -,1058,1986

Model Prediction

Prediction: Mild

Probability: 0.0911

Enrichment: 0.247

Bayesian Score: -8.73

Mahalanobis Distance: 13.5

Mahalanobis Distance p-value: 1.21e-009

Prediction: Positive if the Bayesian score is above the estimated best cutoff value from minimizing the false positive and false negative rate.
Probability: The esimated probability that the sample is in the positive category. This assumes that the Bayesian score follows a normal distribution and is different from the prediction using a cutoff.
Enrichment: An estimate of enrichment, that is, the increased likelihood (versus random) of this sample being in the category.
Bayesian Score: The standard Laplacian-modified Bayesian score.
Mahalanobis Distance: The Mahalanobis distance (MD) is the distance to the center of the training data. The larger the MD, the less trustworthy the prediction.
Mahalanobis Distance p-value: The p-value gives the fraction of training data with an MD greater than or equal to the one for the given sample, assuming normally distributed data. The smaller the p-value, the less trustworthy the prediciton. For highly non-normal X properties (e.g., fingerprints), the MD p-value is wildly inaccurate.

Model Applicability

Unknown features are fingerprint features in the query molecule, but not found or appearing too infrequently in the training set.

1. All properties and OPS components are within expected ranges.
2. Unknown FCFP_2 feature: 472180098: [*]OP(=O)(O)O
3. Unknown FCFP_2 feature: -332197802: [*][c]1:[*]:[*]:[c](:[*]):n:1:n:[*]

Feature Contribution

Top features for positive contribution

Fingerprint	Bit/Smiles	Feature Structure	Score	Moderate_Severe in training set

FCFP_12	-1151884458	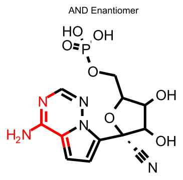 [*]:n:[c](N):[c](:[*]):[*]	0.385	1 out of 1
FCFP_12	76292238	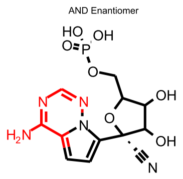 [*]:[c]1:[*]:n:[cH]:n:[c]:1N	0.385	1 out of 1
FCFP_12	-124685461	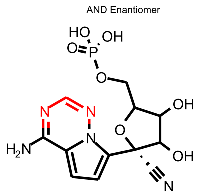 [*]:n:[cH]:n:[*]	0.206	2 out of 4

Top Features for negative contribution

Fingerprint	Bit/Smiles	Feature Structure	Score	Moderate_Severe in training set
FCFP_12	4427049	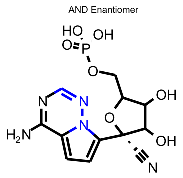 [*]:[cH]:n:n(:[*]):[*]	-0.893	0 out of 4
FCFP_12	-1277879912	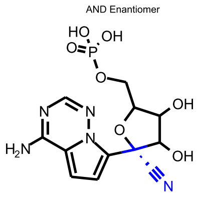 [*]C([*])([*])C#N	-0.548	5 out of 26

FCFP_12	-836603894	AND Enantiomer 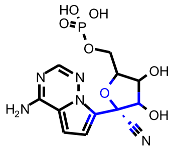 [*]C1[*][*]O[C@]1(C#[*])[c](:[*]):[*]	-0.543	0 out of 2

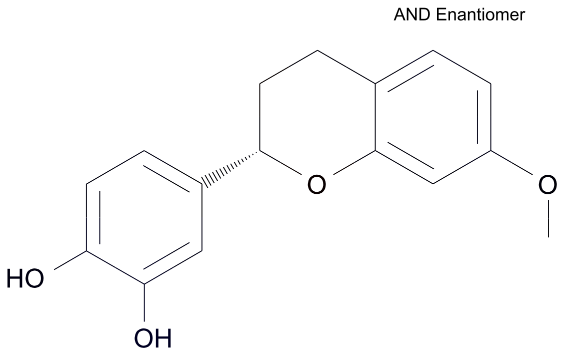

$C_{16}H_{16}O_4$
Molecular Weight: 272.296
ALogP: 3.456
Rotatable Bonds: 2
Acceptors: 4
Donors: 2

Model Prediction

Prediction: Non-Irritant
Probability: 0.953
Enrichment: 1.04
Bayesian Score: -1.78
Mahalanobis Distance: 7.65
Mahalanobis Distance p-value: 0.956

Prediction: Positive if the Bayesian score is above the estimated best cutoff value from minimizing the false positive and false negative rate.
Probability: The esimated probability that the sample is in the positive category. This assumes that the Bayesian score follows a normal distribution and is different from the prediction using a cutoff.
Enrichment: An estimate of enrichment, that is, the increased likelihood (versus random) of this sample being in the category.
Bayesian Score: The standard Laplacian-modified Bayesian score.
Mahalanobis Distance: The Mahalanobis distance (MD) is the distance to the center of the training data. The larger the MD, the less trustworthy the prediction.
Mahalanobis Distance p-value: The p-value gives the fraction of training data with an MD greater than or equal to the one for the given sample, assuming normally distributed data. The smaller the p-value, the less trustworthy the prediciton. For highly non-normal X properties (e.g., fingerprints), the MD p-value is wildly inaccurate.

Structural Similar Compounds

Name	Benzoin, oxime	Benzophenone, 2,4-dihydroxy-	Phenol, 4,4'-thiodi-
Structure	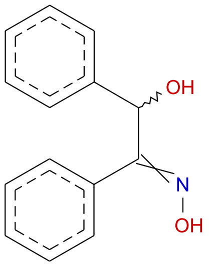	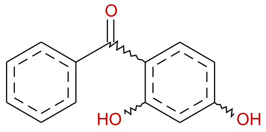	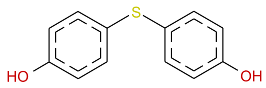
Actual Endpoint	Non-Irritant	Non-Irritant	Irritant
Predicted Endpoint	Non-Irritant	Non-Irritant	Non-Irritant
Distance	0.622	0.622	0.632
Reference	28ZPAK -,111,72	28ZPAK -,101,72	BIOFX* BIOFAX Industrial Bio-Test Laboratories, Inc., Data Sheets. (1810 Fro ntage Rd., Northbrook, IL 60062) Volume(issue)/page/year: A408,1971

Model Applicability

Unknown features are fingerprint features in the query molecule, but not found or appearing too infrequently in the training set.

1. All properties and OPS components are within expected ranges.

Feature Contribution

Top features for positive contribution

Fingerprint	Bit/Smiles	Feature Structure	Score	Irritant in training set
FCFP_12	1186333723	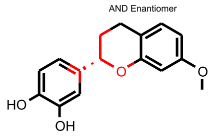 [*]C[C@H](O[*])[c](:[*]):[*]	0.0703	4 out of 4

FCFP_12	1679744180	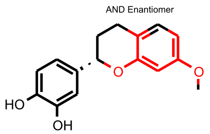<br>[*]O[c]1:[cH]:[*]:[c]([*]):[c](O[*]):[cH]:1	0.0703	4 out of 4
FCFP_12	1916525245	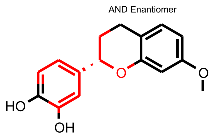<br>[*]C[C@H](O[*])[c]1:[cH]:[cH]:[*]:[c]([*]):[cH]:1	0.0658	3 out of 3

Top Features for negative contribution

Fingerprint	Bit/Smiles	Feature Structure	Score	Irritant in training set
FCFP_12	949015626	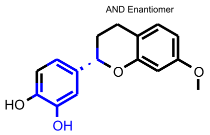<br>[*]C([*])[c]1:[cH]:[*]:[c]([*]):[c](O):[cH]:1	-0.222	2 out of 3
FCFP_12	-204034463	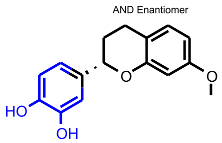<br>O[c]1:[cH]:[*]:[cH]:[cH]:[c]:1O	-0.222	2 out of 3
FCFP_12	7	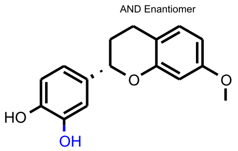<br>[*]O	-0.118	104 out of 128

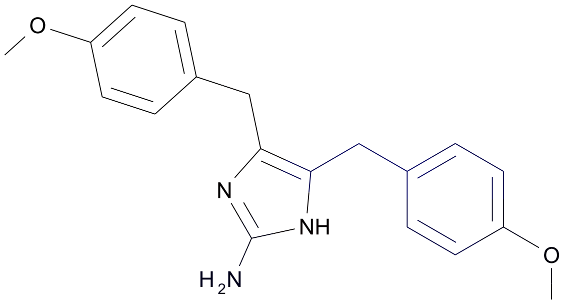

$C_{19}H_{21}N_3O_2$
Molecular Weight: 323.389
ALogP: 3.305
Rotatable Bonds: 6
Acceptors: 4
Donors: 2

Structural Similar Compounds

Name	Disiloxane, 1,3-bis(3-aminopropyl)-1,1,3,3-tetramethyl-	5-Norbornene-2,3-dicarboxylic acid, 1,4,5,6,7,7-hexachloro-	1-Piperazineacetic acid, 4-(2-hydroxyethyl)-alpha-phenyl-, 2,6-xyly l ester, monohydrochloride
Structure	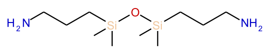	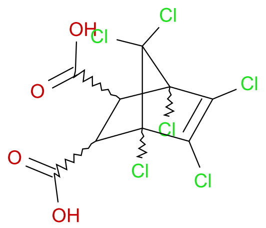	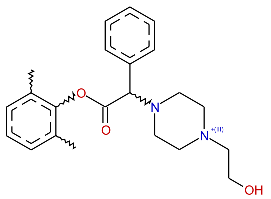
Actual Endpoint	Irritant	Irritant	Irritant
Predicted Endpoint	Irritant	Irritant	Irritant
Distance	0.706	0.731	0.732
Reference	NTIS** National Technical Information Service. (Springfield, VA 22161) Forme rly U.S. Clearinghouse for Scientific & Technical Information. Volume(issue)/pag e/year: OTS0535667	85JCAE "Prehled Prumyslove Toxikologie; Organicke Latky," Marhold, J., Prague , Czechoslovakia, Avicenum, 1986 Volume(issue)/page/year: -,581,1986	BCFAAI Bollettino Chimico Farmaceutico. (Societa Editoriale Farmaceutica, Vi a Ausonio 12, 20123 Milan, Italy) V.33- 1894- Volume(issue)/page/year: 107,3 10,1968

Model Prediction

Prediction: Non-Irritant
Probability: 0.878
Enrichment: 0.953
Bayesian Score: -2.98
Mahalanobis Distance: 6.37
Mahalanobis Distance p-value: 1

Prediction: Positive if the Bayesian score is above the estimated best cutoff value from minimizing the false positive and false negative rate.
Probability: The esimated probability that the sample is in the positive category. This assumes that the Bayesian score follows a normal distribution and is different from the prediction using a cutoff.
Enrichment: An estimate of enrichment, that is, the increased likelihood (versus random) of this sample being in the category.
Bayesian Score: The standard Laplacian-modified Bayesian score.
Mahalanobis Distance: The Mahalanobis distance (MD) is the distance to the center of the training data. The larger the MD, the less trustworthy the prediction.
Mahalanobis Distance p-value: The p-value gives the fraction of training data with an MD greater than or equal to the one for the given sample, assuming normally distributed data. The smaller the p-value, the less trustworthy the prediciton. For highly non-normal X properties (e.g., fingerprints), the MD p-value is wildly inaccurate.

Model Applicability

Unknown features are fingerprint features in the query molecule, but not found or appearing too infrequently in the training set.

1. All properties and OPS components are within expected ranges.
2. Unknown FCFP_2 feature: 203707511: [*]C[c]1:[nH]:[*]:[*]:[c]:1[*]
3. Unknown FCFP_2 feature: -1151854667: N[c]1:[nH]:[*]:[*]:n:1

Feature Contribution

Top features for positive contribution

Fingerprint	Bit/Smiles	Feature Structure	Score	Irritant in training set

FCFP_12	-1539132615	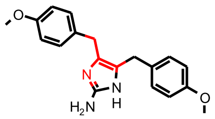 [*]C[c]1:n:[*]:[*]:[c]:1[*]	0.0795	9 out of 9
FCFP_12	19	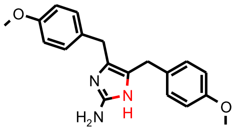 [*]:[nH]:[*]	0.0658	3 out of 3
FCFP_12	2005402822	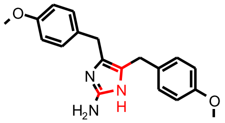 [*][c]1:[*]:[*]:[c]([*]):[nH]:1	0.0658	3 out of 3

Top Features for negative contribution

Fingerprint	Bit/Smiles	Feature Structure	Score	Irritant in training set
FCFP_12	1758843539	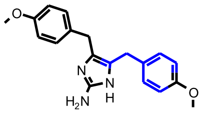 [*]:[c](:[*])C[c]1:[cH]:[cH]:[*]:[cH]:[cH]:1	-1.04	0 out of 2
FCFP_12	906530397	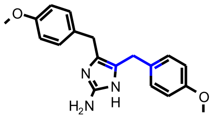 [*]:[c](:[*])C[c](:[*]):[*]	-0.627	1 out of 3

FCFP_12	1069584379	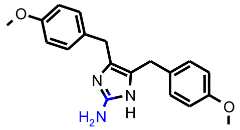<br>[*]:[c](:[*])N	-0.439	38 out of 65

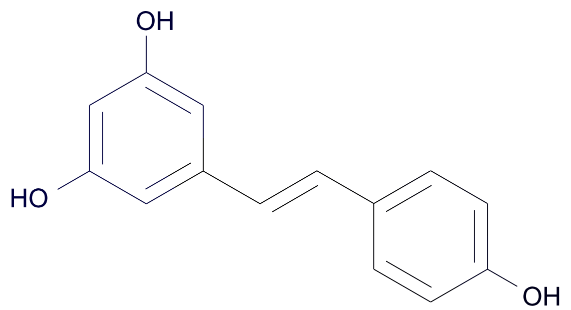

$C_{14}H_{12}O_3$
Molecular Weight: 228.243
ALogP: 3.09
Rotatable Bonds: 2
Acceptors: 3
Donors: 3

Model Prediction

Prediction: Non-Irritant
Probability: 0.937
Enrichment: 1.02
Bayesian Score: -2.2
Mahalanobis Distance: 6.26
Mahalanobis Distance p-value: 1

Prediction: Positive if the Bayesian score is above the estimated best cutoff value from minimizing the false positive and false negative rate.
Probability: The esimated probability that the sample is in the positive category. This assumes that the Bayesian score follows a normal distribution and is different from the prediction using a cutoff.
Enrichment: An estimate of enrichment, that is, the increased likelihood (versus random) of this sample being in the category.
Bayesian Score: The standard Laplacian-modified Bayesian score.
Mahalanobis Distance: The Mahalanobis distance (MD) is the distance to the center of the training data. The larger the MD, the less trustworthy the prediction.
Mahalanobis Distance p-value: The p-value gives the fraction of training data with an MD greater than or equal to the one for the given sample, assuming normally distributed data. The smaller the p-value, the less trustworthy the prediciton. For highly non-normal X properties (e.g., fingerprints), the MD p-value is wildly inaccurate.

Structural Similar Compounds

Name	Benzophenone, 2,4-dihydroxy-	Phenol, 4,4'-thiodi-	Phenol, 4,4'-isopropylidenedi-
Structure	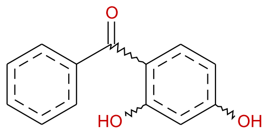	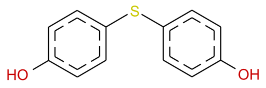	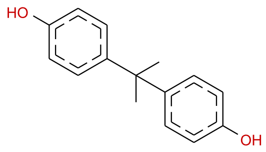
Actual Endpoint	Non-Irritant	Irritant	Irritant
Predicted Endpoint	Non-Irritant	Non-Irritant	Non-Irritant
Distance	0.599	0.634	0.657
Reference	28ZPAK -,101,72	BIOFX* BIOFAX Industrial Bio-Test Laboratories, Inc., Data Sheets. (1810 Fro ntage Rd., Northbrook, IL 60062) Volume(issue)/page/year: A408,1971	85JCAE "Prehled Prumyslove Toxikologie; Organicke Latky," Marhold, J., Prague , Czechoslovakia, Avicenum, 1986 Volume(issue)/page/year: -,238,1986

Model Applicability

Unknown features are fingerprint features in the query molecule, but not found or appearing too infrequently in the training set.

1. All properties and OPS components are within expected ranges.

Feature Contribution

Top features for positive contribution

Fingerprint	Bit/Smiles	Feature Structure	Score	Irritant in training set
FCFP_12	-1748394506	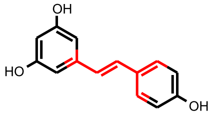 [*]:[cH]:[c](\C=C\[c](:[*]):[*]):[cH]:[*]	0.0734	5 out of 5

FCFP_12	2006871518	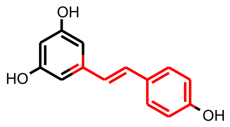 [*][c]1:[cH]:[cH]:[c](\C=C\[c](:[*]):[*]):[cH]:[cH]:1	0.0658	3 out of 3
FCFP_12	-1373956533	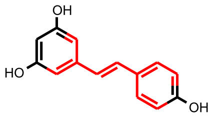 [*]:[cH]:[c](\C=C\[c]1:[cH]:[cH]:[*]:[cH]:[cH]:1):[cH]:[*]	0.0658	3 out of 3

Top Features for negative contribution

Fingerprint	Bit/Smiles	Feature Structure	Score	Irritant in training set
FCFP_12	946068634	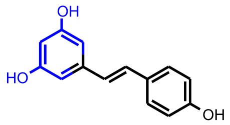 O[c]1:[cH]:[*]:[cH]:[c](O):[cH]:1	-1.04	0 out of 2
FCFP_12	949015626	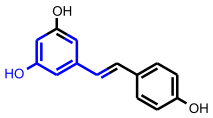 [*]C([*])[c]1:[cH]:[*]:[c]([*]):[c](O):[cH]:1	-0.222	2 out of 3
FCFP_12	7	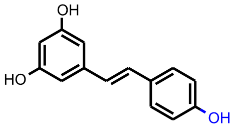 [*]O	-0.118	104 out of 128

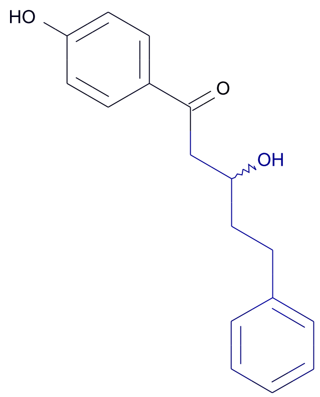

$C_{17}H_{18}O_3$

Molecular Weight: 270.323

ALogP: 3.293

Rotatable Bonds: 6

Acceptors: 3

Donors: 2

Model Prediction

Prediction: Non-Irritant

Probability: 0.51

Enrichment: 0.554

Bayesian Score: -4.52

Mahalanobis Distance: 8.56

Mahalanobis Distance p-value: 0.655

Prediction: Positive if the Bayesian score is above the estimated best cutoff value from minimizing the false positive and false negative rate.
Probability: The esimated probability that the sample is in the positive category. This assumes that the Bayesian score follows a normal distribution and is different from the prediction using a cutoff.
Enrichment: An estimate of enrichment, that is, the increased likelihood (versus random) of this sample being in the category.
Bayesian Score: The standard Laplacian-modified Bayesian score.
Mahalanobis Distance: The Mahalanobis distance (MD) is the distance to the center of the training data. The larger the MD, the less trustworthy the prediction.
Mahalanobis Distance p-value: The p-value gives the fraction of training data with an MD greater than or equal to the one for the given sample, assuming normally distributed data. The smaller the p-value, the less trustworthy the prediciton. For highly non-normal X properties (e.g., fingerprints), the MD p-value is wildly inaccurate.

Structural Similar Compounds

Name	Benzoin, oxime	Disiloxane, 1,3-bis(3-aminopropyl)-1,1,3,3-tetramethyl-	Ethyl 2-hydroxy-4-phenyl butyrate
Structure	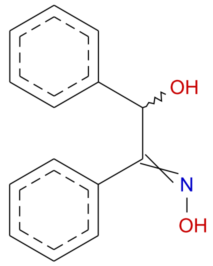	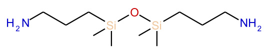	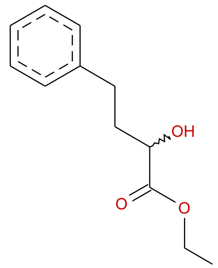
Actual Endpoint	Non-Irritant	Irritant	Non-Irritant
Predicted Endpoint	Non-Irritant	Irritant	Non-Irritant
Distance	0.581	0.585	0.623
Reference	28ZPAK -,111,72	NTIS** National Technical Information Service. (Springfield, VA 22161) Forme rly U.S. Clearinghouse for Scientific & Technical Information. Volume(issue)/pag e/year: OTS0535667	US ARMY

Model Applicability

Unknown features are fingerprint features in the query molecule, but not found or appearing too infrequently in the training set.

1. All properties and OPS components are within expected ranges.

Feature Contribution

Top Features for negative contribution				
Fingerprint	Bit/Smiles	Feature Structure	Score	Irritant in training set

FCFP_12	1629755549	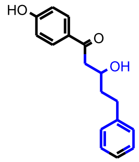 [*]CC(O)CC[c]1:[cH]:[cH]:[*]:[cH]:[cH]:1	-1.04	0 out of 2
FCFP_12	95051074	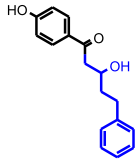 [*]CC(O)CC[c]1:[cH]:[cH]:[cH]:[cH]:[cH]:1	-1.04	0 out of 2
FCFP_12	-1442566880	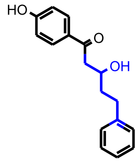 [*]CC(O)CC[c](:[*]):[*]	-0.627	1 out of 3

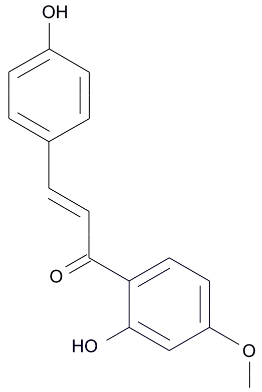

$C_{16}H_{14}O_4$
Molecular Weight: 270.28
ALogP: 3.201
Rotatable Bonds: 4
Acceptors: 4
Donors: 2

Model Prediction

Prediction: Non-Irritant
Probability: 0.958
Enrichment: 1.04
Bayesian Score: -1.63
Mahalanobis Distance: 7.52
Mahalanobis Distance p-value: 0.971

Prediction: Positive if the Bayesian score is above the estimated best cutoff value from minimizing the false positive and false negative rate.
Probability: The esimated probability that the sample is in the positive category. This assumes that the Bayesian score follows a normal distribution and is different from the prediction using a cutoff.
Enrichment: An estimate of enrichment, that is, the increased likelihood (versus random) of this sample being in the category.
Bayesian Score: The standard Laplacian-modified Bayesian score.
Mahalanobis Distance: The Mahalanobis distance (MD) is the distance to the center of the training data. The larger the MD, the less trustworthy the prediction.
Mahalanobis Distance p-value: The p-value gives the fraction of training data with an MD greater than or equal to the one for the given sample, assuming normally distributed data. The smaller the p-value, the less trustworthy the prediciton. For highly non-normal X properties (e.g., fingerprints), the MD p-value is wildly inaccurate.

Structural Similar Compounds

Name	Benzophenone, 2,4-dihydroxy-	Phenol, 4,4'-sulfonyldi-	Phenol, 4,4'-thiodi-
Structure	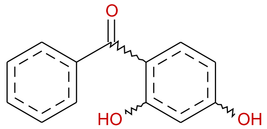	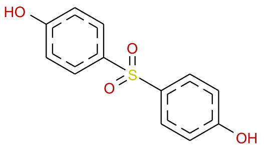	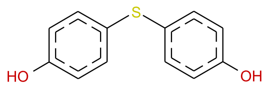
Actual Endpoint	Non-Irritant	Irritant	Irritant
Predicted Endpoint	Non-Irritant	Non-Irritant	Non-Irritant
Distance	0.602	0.616	0.617
Reference	28ZPAK -,101,72	BIOFX* BIOFAX Industrial Bio-Test Laboratories, Inc., Data Sheets. (1810 Fro ntage Rd., Northbrook, IL 60062) Volume(issue)/page/year: 601-05501,1974	BIOFX* BIOFAX Industrial Bio-Test Laboratories, Inc., Data Sheets. (1810 Fro ntage Rd., Northbrook, IL 60062) Volume(issue)/page/year: A408,1971

Model Applicability

Unknown features are fingerprint features in the query molecule, but not found or appearing too infrequently in the training set.

1. All properties and OPS components are within expected ranges.

Feature Contribution

Top features for positive contribution

Fingerprint	Bit/Smiles	Feature Structure	Score	Irritant in training set
FCFP_12	-146015125	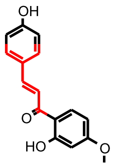 [*]C(=[*])\C=C\[c](:[cH]:[*]):[cH]:[*]	0.085	24 out of 24

FCFP_12	2011169140	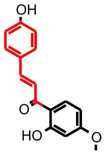 [*]C(=[*])\C=C\[c]1:[cH]:[cH]:[c]([*]):[cH]:[cH]:1	0.0829	15 out of 15
FCFP_12	451847724	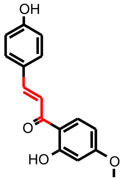 [*]\C=C\C(=[*])[*]	0.0737	270 out of 274

Top Features for negative contribution

Fingerprint	Bit/Smiles	Feature Structure	Score	Irritant in training set
FCFP_12	-1604301295	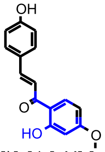 [*]C(=[*])[c]1:[cH]:[*]:[c]([*]):[cH]:[c]:1O	-0.18	22 out of 29
FCFP_12	7	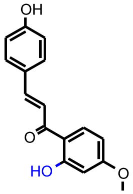 [*]O	-0.118	104 out of 128
FCFP_12	-549108873	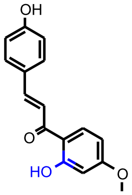 [*]:[c](:[*])O	-0.11	54 out of 66

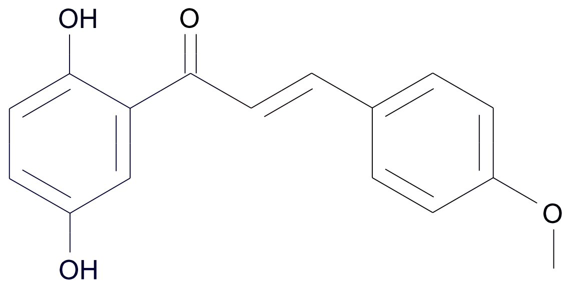

$C_{16}H_{14}O_4$
Molecular Weight: 270.28
ALogP: 3.201
Rotatable Bonds: 4
Acceptors: 4
Donors: 2

Model Prediction

Prediction: Non-Irritant
Probability: 0.945
Enrichment: 1.03
Bayesian Score: -2.02
Mahalanobis Distance: 7.52
Mahalanobis Distance p-value: 0.971

Prediction: Positive if the Bayesian score is above the estimated best cutoff value from minimizing the false positive and false negative rate.
Probability: The esimated probability that the sample is in the positive category. This assumes that the Bayesian score follows a normal distribution and is different from the prediction using a cutoff.
Enrichment: An estimate of enrichment, that is, the increased likelihood (versus random) of this sample being in the category.
Bayesian Score: The standard Laplacian-modified Bayesian score.
Mahalanobis Distance: The Mahalanobis distance (MD) is the distance to the center of the training data. The larger the MD, the less trustworthy the prediction.
Mahalanobis Distance p-value: The p-value gives the fraction of training data with an MD greater than or equal to the one for the given sample, assuming normally distributed data. The smaller the p-value, the less trustworthy the prediciton. For highly non-normal X properties (e.g., fingerprints), the MD p-value is wildly inaccurate.

Structural Similar Compounds

Name	Benzophenone, 2,4-dihydroxy-	Benzoin, oxime	Phenol, 4,4'-thiodi-
Structure	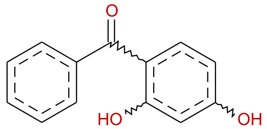	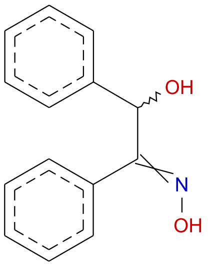	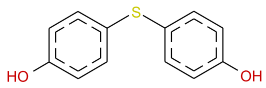
Actual Endpoint	Non-Irritant	Non-Irritant	Irritant
Predicted Endpoint	Non-Irritant	Non-Irritant	Non-Irritant
Distance	0.608	0.616	0.623
Reference	28ZPAK -,101,72	28ZPAK -,111,72	BIOFX* BIOFAX Industrial Bio-Test Laboratories, Inc., Data Sheets. (1810 Fro ntage Rd., Northbrook, IL 60062) Volume(issue)/page/year: A408,1971

Model Applicability

Unknown features are fingerprint features in the query molecule, but not found or appearing too infrequently in the training set.

1. All properties and OPS components are within expected ranges.

Feature Contribution

Top features for positive contribution

Fingerprint	Bit/Smiles	Feature Structure	Score	Irritant in training set
FCFP_12	-146015125	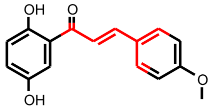 [*]C(=[*])\C=C\[c](:[cH]:[*]):[cH]:[*]	0.085	24 out of 24

FCFP_12	2011169140	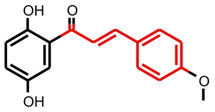 [*]C(=[*])\C=C\[c]1:[cH]:[cH]:[c]([*]):[cH]:[cH]:1	0.0829	15 out of 15
FCFP_12	451847724	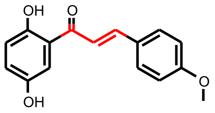 [*]\C=C\C(=[*])[*]	0.0737	270 out of 274

Top Features for negative contribution

Fingerprint	Bit/Smiles	Feature Structure	Score	Irritant in training set
FCFP_12	-305225196	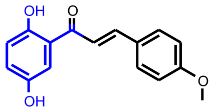 [*]C(=[*])[c]1:[cH]:[c](O):[cH]:[cH]:[c]:1O	-0.347	1 out of 2
FCFP_12	949015626	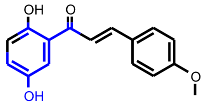 [*]C([*])[c]1:[cH]:[*]:[c]([*]):[c](O):[cH]:1	-0.222	2 out of 3
FCFP_12	-1604301295	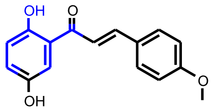 [*]C(=[*])[c]1:[cH]:[*]:[c]([*]):[cH]:[c]:1O	-0.18	22 out of 29

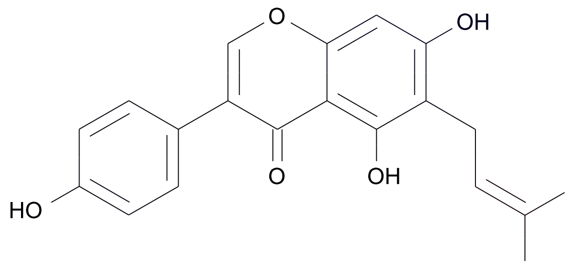

$C_{20}H_{18}O_5$
Molecular Weight: 338.354
ALogP: 3.997
Rotatable Bonds: 3
Acceptors: 5
Donors: 3

Model Prediction

Prediction: Non-Irritant
Probability: 0.97
Enrichment: 1.05
Bayesian Score: -0.996
Mahalanobis Distance: 13
Mahalanobis Distance p-value: 8.85e-008

Prediction: Positive if the Bayesian score is above the estimated best cutoff value from minimizing the false positive and false negative rate.
Probability: The esimated probability that the sample is in the positive category. This assumes that the Bayesian score follows a normal distribution and is different from the prediction using a cutoff.
Enrichment: An estimate of enrichment, that is, the increased likelihood (versus random) of this sample being in the category.
Bayesian Score: The standard Laplacian-modified Bayesian score.
Mahalanobis Distance: The Mahalanobis distance (MD) is the distance to the center of the training data. The larger the MD, the less trustworthy the prediction.
Mahalanobis Distance p-value: The p-value gives the fraction of training data with an MD greater than or equal to the one for the given sample, assuming normally distributed data. The smaller the p-value, the less trustworthy the prediciton. For highly non-normal X properties (e.g., fingerprints), the MD p-value is wildly inaccurate.

Structural Similar Compounds

Name	1-Amino-2-bromo-4-hydroxyanthraquinone	5-Norbornene-2,3-dicarboxylic acid, 1,4,5,6,7,7-hexachloro-	Phenol, 4,4'-sulfonyldi-
Structure	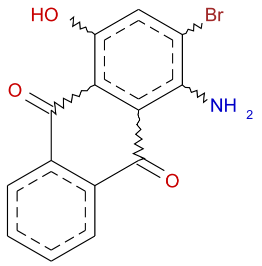	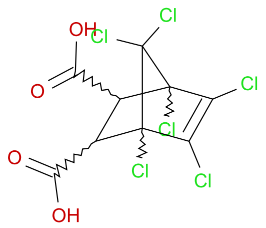	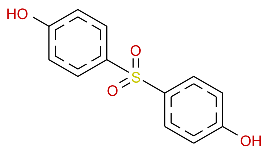
Actual Endpoint	Non-Irritant	Irritant	Irritant
Predicted Endpoint	Non-Irritant	Irritant	Non-Irritant
Distance	0.761	0.775	0.776
Reference	28ZPAK -,83,72	85JCAE "Prehled Prumyslove Toxikologie; Organicke Latky," Marhold, J., Prague , Czechoslovakia, Avicenum, 1986 Volume(issue)/page/year: -,581,1986	BIOFX* BIOFAX Industrial Bio-Test Laboratories, Inc., Data Sheets. (1810 Fro ntage Rd., Northbrook, IL 60062) Volume(issue)/page/year: 601-05501,1974

Model Applicability

Unknown features are fingerprint features in the query molecule, but not found or appearing too infrequently in the training set.

1. All properties and OPS components are within expected ranges.

Feature Contribution

Top features for positive contribution				
Fingerprint	Bit/Smiles	Feature Structure	Score	Irritant in training set

FCFP_12	-1582522951	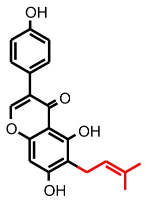 [*]CC=C(C)C	0.0868	48 out of 48
FCFP_12	451877515	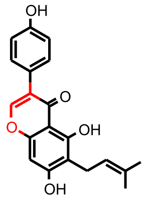 [*]OC=C([*])[*]	0.0821	13 out of 13
FCFP_12	436886043	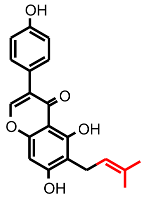 [*]C=C(C)C	0.0804	129 out of 130

Top Features for negative contribution

Fingerprint	Bit/Smiles	Feature Structure	Score	Irritant in training set
FCFP_12	-1604301295	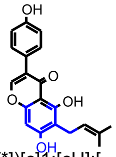 [*]C(=[*])[c]1:[cH]:[*]:[c]([*]):[cH]:[c]:1O	-0.18	22 out of 29
FCFP_12	7	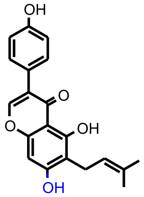 [*]O	-0.118	104 out of 128

FCFP_12	74595001	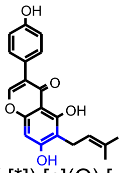 [*][c](:[*]):[c](O):[cH]:[*]	-0.11	54 out of 66

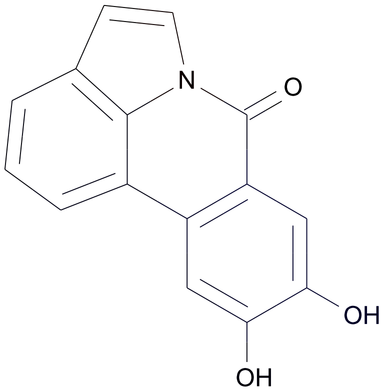

$C_{15}H_9NO_3$
Molecular Weight: 251.237
ALogP: 2.982
Rotatable Bonds: 0
Acceptors: 3
Donors: 2

Structural Similar Compounds

Name	Benzophenone, 2,4-dihydroxy-	Phenol, 4,4'-thiodi-	Anthraquinone, 1,4-dihydroxy-
Structure	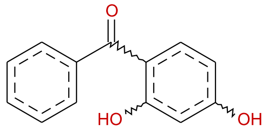	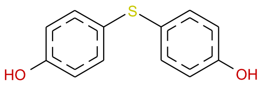	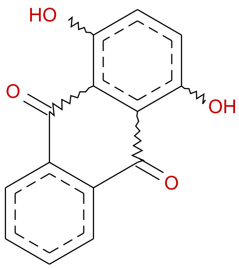
Actual Endpoint	Non-Irritant	Irritant	Non-Irritant
Predicted Endpoint	Non-Irritant	Non-Irritant	Non-Irritant
Distance	0.565	0.591	0.596
Reference	28ZPAK -,101,72	BIOFX* BIOFAX Industrial Bio-Test Laboratories, Inc., Data Sheets. (1810 Fro ntage Rd., Northbrook, IL 60062) Volume(issue)/page/year: A408,1971	28ZPAK -,102,72

Model Prediction

Prediction: Non-Irritant
Probability: 0.952
Enrichment: 1.03
Bayesian Score: -1.82
Mahalanobis Distance: 5.5
Mahalanobis Distance p-value: 1

Prediction: Positive if the Bayesian score is above the estimated best cutoff value from minimizing the false positive and false negative rate.
Probability: The esimated probability that the sample is in the positive category. This assumes that the Bayesian score follows a normal distribution and is different from the prediction using a cutoff.
Enrichment: An estimate of enrichment, that is, the increased likelihood (versus random) of this sample being in the category.
Bayesian Score: The standard Laplacian-modified Bayesian score.
Mahalanobis Distance: The Mahalanobis distance (MD) is the distance to the center of the training data. The larger the MD, the less trustworthy the prediction.
Mahalanobis Distance p-value: The p-value gives the fraction of training data with an MD greater than or equal to the one for the given sample, assuming normally distributed data. The smaller the p-value, the less trustworthy the prediciton. For highly non-normal X properties (e.g., fingerprints), the MD p-value is wildly inaccurate.

Model Applicability

Unknown features are fingerprint features in the query molecule, but not found or appearing too infrequently in the training set.

1. All properties and OPS components are within expected ranges.
2. Unknown FCFP_2 feature: -1549639687: [*]:n(:[*])C(=O)[c](:[*]):[*]

Feature Contribution

Top features for positive contribution				
Fingerprint	Bit/Smiles	Feature Structure	Score	Irritant in training set
FCFP_12	-124655670	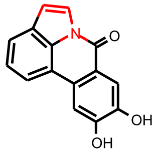 [*]n1:[*]:[*]:[cH]:[cH]:1	0.0821	13 out of 13

FCFP_12	1804743636	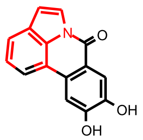 [*]n1:[cH]:[cH]:[c]2:[cH]:[cH]:[*]:[c]([*]):[c]:1:2	0.0658	3 out of 3
FCFP_12	546102568	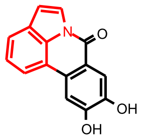 [*]n1:[cH]:[cH]:[c]2:[cH]:[cH]:[cH]:[c]([*]):[c]:1:2	0.0583	2 out of 2
**Top Features for negative contribution**				
**Fingerprint**	**Bit/Smiles**	**Feature Structure**	**Score**	**Irritant in training set**
FCFP_12	-204034463	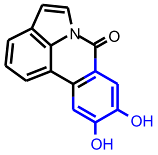 O[c]1:[cH]:[*]:[cH]:[cH]:[c]:1O	-0.222	2 out of 3
FCFP_12	949015626	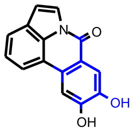 [*]C([*])[c]1:[cH]:[*]:[c]([*]):[c](O):[cH]:1	-0.222	2 out of 3
FCFP_12	900733322	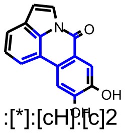 [*][c]1:[*]:[cH]:[c]2C(=[*])[*]:[c](:[*]):[c](:[cH]:[*])[c]:2:[cH]:1	-0.153	3 out of 4

remdesivir

TOPKAT_Skin_Irritancy_None_vs_Irritant

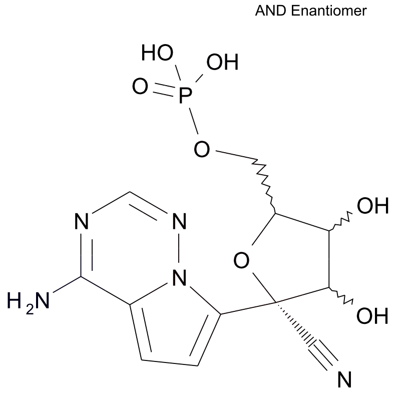

$C_{12}H_{14}N_5O_7P$
Molecular Weight: 371.24262
ALogP: -1.502
Rotatable Bonds: 4
Acceptors: 11
Donors: 5

Model Prediction

Prediction: Irritant
Probability: 0.976
Enrichment: 1.06
Bayesian Score: -0.492
Mahalanobis Distance: 13.2
Mahalanobis Distance p-value: 3.18e-008

Prediction: Positive if the Bayesian score is above the estimated best cutoff value from minimizing the false positive and false negative rate.
Probability: The esimated probability that the sample is in the positive category. This assumes that the Bayesian score follows a normal distribution and is different from the prediction using a cutoff.
Enrichment: An estimate of enrichment, that is, the increased likelihood (versus random) of this sample being in the category.
Bayesian Score: The standard Laplacian-modified Bayesian score.
Mahalanobis Distance: The Mahalanobis distance (MD) is the distance to the center of the training data. The larger the MD, the less trustworthy the prediction.
Mahalanobis Distance p-value: The p-value gives the fraction of training data with an MD greater than or equal to the one for the given sample, assuming normally distributed data. The smaller the p-value, the less trustworthy the prediciton. For highly non-normal X properties (e.g., fingerprints), the MD p-value is wildly inaccurate.

Structural Similar Compounds

Name	1,3,6-Naphthalenetrisulfonic acid, 7-amino-	2,2'-Benzidine disulfonic acid	2,7-Anthracenedisulfonic acid, 9,10-dihydro-4,5-diamino-9,10-dioxo- 1-hydroxy-, disodium salt
Structure	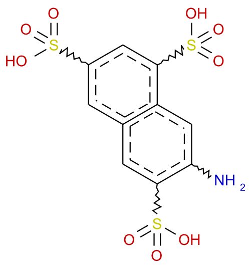	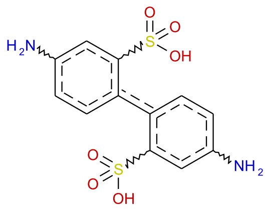	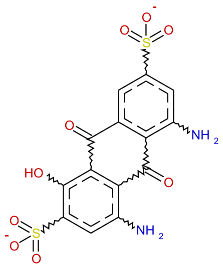
Actual Endpoint	Irritant	Non-Irritant	Irritant
Predicted Endpoint	Non-Irritant	Non-Irritant	Non-Irritant
Distance	0.755	0.896	1.025
Reference	85JCAE "Prehled Prumyslove Toxikologie; Organicke Latky," Marhold, J., Prague , Czechoslovakia, Avicenum, 1986 Volume(issue)/page/year: -,1058,1986	28ZPAK -,191,72	28ZPAK "Sbornik Vysledku Toxixologickeho Vysetreni Latek A Pripravku," Marhol d, J.V., Institut Pro Vychovu Vedoucicn Pracovniku Chemickeho Prumyclu Praha, Cz echoslovakia, 1972 Volume(issue)/page/year: -,239,1

Model Applicability

Unknown features are fingerprint features in the query molecule, but not found or appearing too infrequently in the training set.

1. All properties and OPS components are within expected ranges.
2. Unknown FCFP_2 feature: 472180098: [*]OP(=O)(O)O
3. Unknown FCFP_2 feature: -332197802: [*][c]1:[*]:[*]:[c](:[*]):n:1:n:[*]

Feature Contribution

Top features for positive contribution				
**Fingerprint**	**Bit/Smiles**	**Feature Structure**	**Score**	**Irritant in training set**

FCFP_12	654335567	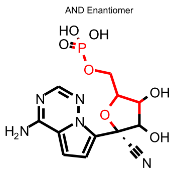 [*]C1[*][*]OC1COP(=[*])([*])[*]	0.0856	29 out of 29
FCFP_12	-1539132615	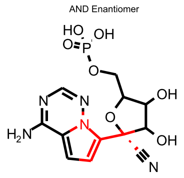 [*]C[c]1:n:[*]:[*]:[c]:1[*]	0.0795	9 out of 9
FCFP_12	-1280036918	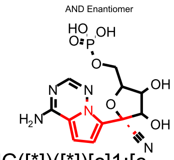 [*]C([*])([*])[c]1:[c H]:[cH]:[c](:[*]):n: 1:[*]	0.0772	7 out of 7

Top Features for negative contribution

Fingerprint	Bit/Smiles	Feature Structure	Score	Irritant in training set
FCFP_12	1069584379	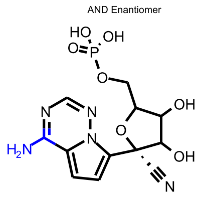 [*]:[c](:[*])N	-0.439	38 out of 65
FCFP_12	1618154665	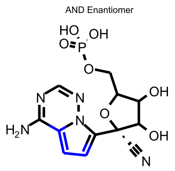 [*][c](:[*]):[cH]:[cH]:[*]	-0.0845	412 out of 490

FCFP_12	16	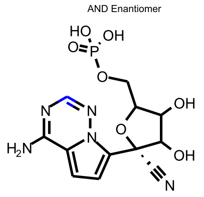<br>[*][c](:[*]):[*]	-0.0843	423 out of 503

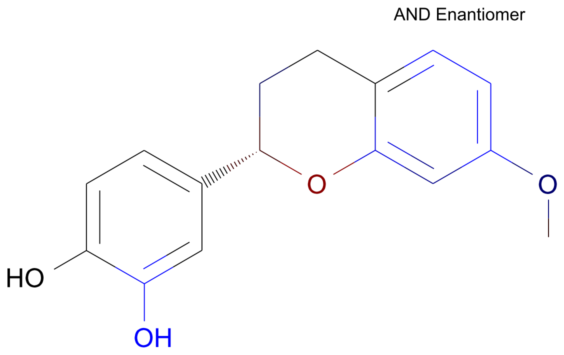

$C_{16}H_{16}O_4$
Molecular Weight: 272.296
ALogP: 3.456
Rotatable Bonds: 2
Acceptors: 4
Donors: 2

Model Prediction

Prediction: 63
Unit: mg/kg_body_weight/day
Mahalanobis Distance: 10.6
Mahalanobis Distance p-value: 0.00528

Mahalanobis Distance: The Mahalanobis distance (MD) is a generalization of the Euclidean distance that accounts for correlations among the X properties. It is calculated as the distance to the center of the training data. The larger the MD, the less trustworthy the prediction.
Mahalanobis Distance p-value: The p-value gives the fraction of training data with an MD greater than or equal to the one for the given sample, assuming normally distributed data. The smaller the p-value, the less trustworthy the prediciton. For highly non-normal X properties (e.g., fingerprints), the MD p-value is wildly inaccurate.

Structural Similar Compounds

Name	693	Oxazepam	44
Structure	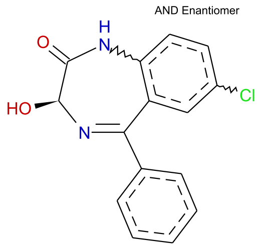	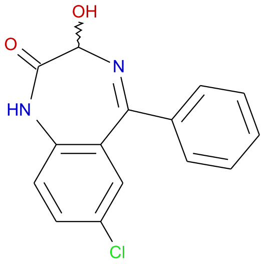	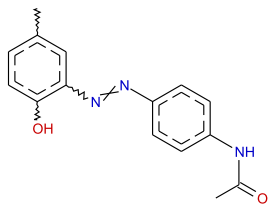
Actual Endpoint (-log C)	3.90356	3.90356	2.42163
Predicted Endpoint (-log C)	3.39677	3.39677	2.85113
Distance	0.565	0.565	0.586
Reference	CPDB	CPDB	CPDB

Model Applicability

Unknown features are fingerprint features in the query molecule, but not found or appearing too infrequently in the training set.

1. All properties and OPS components are within expected ranges.

Feature Contribution

Top features for positive contribution			
Fingerprint	Bit/Smiles	Feature Structure	Score
ECFP_6	683445015	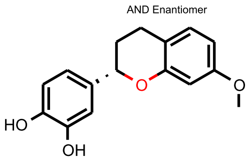 [*]O[*]	0.136

ECFP_6	-176455838	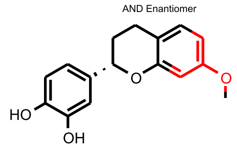<br>[*]O[c](:[cH]:[*]):[cH]:[*]	0.0818
ECFP_6	-167460056	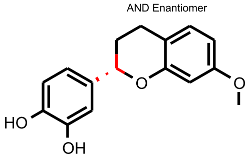<br>[*]C([*])[*]	0.0596

Top Features for negative contribution

**Fingerprint**	**Bit/Smiles**	**Feature Structure**	**Score**
ECFP_6	2019062761	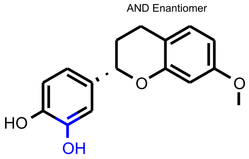<br>[*]:[c](:[*])O	-0.258
ECFP_6	1996767644	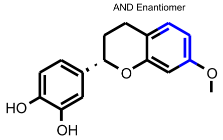<br>[*][c](:[*]):[cH]:[cH]:[*]	-0.251
ECFP_6	642810091	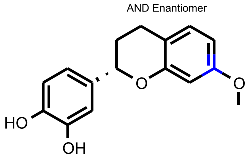<br>[*][c](:[*]):[*]	-0.247

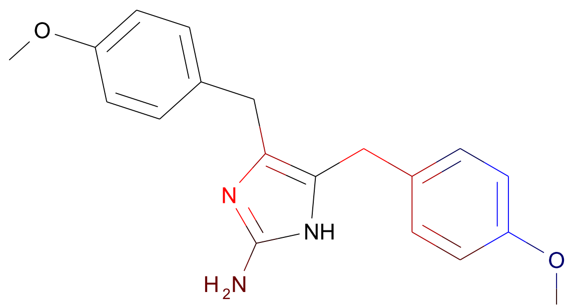

$C_{19}H_{21}N_3O_2$
Molecular Weight: 323.389
ALogP: 3.305
Rotatable Bonds: 6
Acceptors: 4
Donors: 2

Model Prediction

Prediction: 4.02
Unit: mg/kg_body_weight/day
Mahalanobis Distance: 11.9
Mahalanobis Distance p-value: 3.08e-005

Mahalanobis Distance: The Mahalanobis distance (MD) is a generalization of the Euclidean distance that accounts for correlations among the X properties. It is calculated as the distance to the center of the training data. The larger the MD, the less trustworthy the prediction.
Mahalanobis Distance p-value: The p-value gives the fraction of training data with an MD greater than or equal to the one for the given sample, assuming normally distributed data. The smaller the p-value, the less trustworthy the prediciton. For highly non-normal X properties (e.g., fingerprints), the MD p-value is wildly inaccurate.

Structural Similar Compounds

Name	455	3,3´-Dimethoxybenzidine.2HCl	Phenolphthalein
Structure	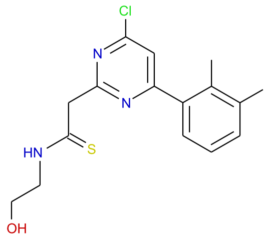	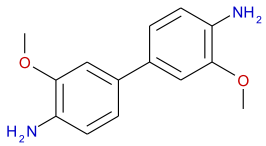	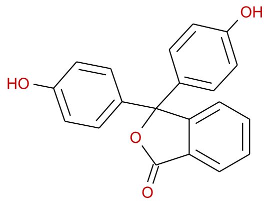
Actual Endpoint (-log C)	3.87681	3.51985	2.43468
Predicted Endpoint (-log C)	3.77582	3.20081	3.66084
Distance	0.687	0.706	0.709
Reference	CPDB	CPDB	CPDB

Model Applicability

Unknown features are fingerprint features in the query molecule, but not found or appearing too infrequently in the training set.

1. OPS PC13 out of range. Value: -3.294. Training min, max, SD, explained variance: -3.068, 3.6909, 1.329, 0.0220.
2. Unknown ECFP_2 feature: -746759483: [*]C[c]1:[nH]:[*]:[*]:[c]:1[*]
3. Unknown ECFP_2 feature: -435942924: [*]C[c]1:n:[*]:[*]:[c]:1[*]

Feature Contribution

Top features for positive contribution

Fingerprint	Bit/Smiles	Feature Structure	Score
ECFP_6	655739385	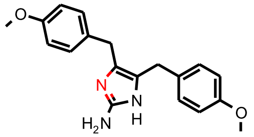 [*]:n:[*]	0.229

ECFP_6	1572579716	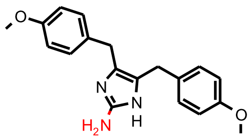 [*]N	0.225
ECFP_6	1559650422	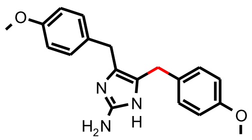 [*]C[*]	0.203

Top Features for negative contribution

Fingerprint	Bit/Smiles	Feature Structure	Score
ECFP_6	1996767644	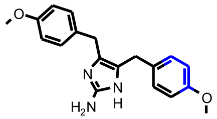 [*][c](:[*]):[cH]:[cH]:[*]	-0.251
ECFP_6	642810091	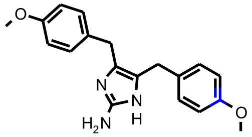 [*][c](:[*]):[*]	-0.247
ECFP_6	-182236392	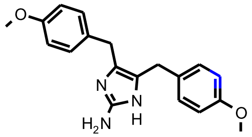 [*]:[cH]:[*]	-0.232

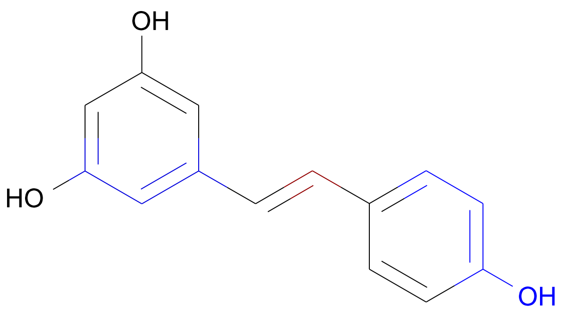

$C_{14}H_{12}O_3$
Molecular Weight: 228.243
ALogP: 3.09
Rotatable Bonds: 2
Acceptors: 3
Donors: 3

Model Prediction

Prediction: 87.5
Unit: mg/kg_body_weight/day
Mahalanobis Distance: 7.62
Mahalanobis Distance p-value: 0.901

Mahalanobis Distance: The Mahalanobis distance (MD) is a generalization of the Euclidean distance that accounts for correlations among the X properties. It is calculated as the distance to the center of the training data. The larger the MD, the less trustworthy the prediction.
Mahalanobis Distance p-value: The p-value gives the fraction of training data with an MD greater than or equal to the one for the given sample, assuming normally distributed data. The smaller the p-value, the less trustworthy the prediciton. For highly non-normal X properties (e.g., fingerprints), the MD p-value is wildly inaccurate.

Structural Similar Compounds

Name	691	4,4´-Oxydianiline	4,4´-Thiodianiline
Structure	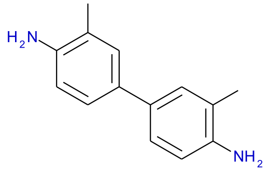	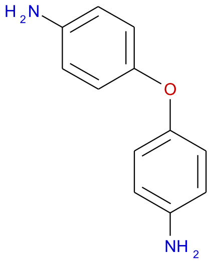	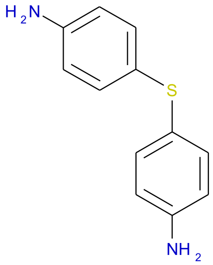
Actual Endpoint (-log C)	3.87056	3.7752	3.81392
Predicted Endpoint (-log C)	3.41789	3.57646	3.72747
Distance	0.609	0.618	0.619
Reference	CPDB	CPDB	CPDB

Model Applicability

Unknown features are fingerprint features in the query molecule, but not found or appearing too infrequently in the training set.

1. All properties and OPS components are within expected ranges.

Feature Contribution

Top features for positive contribution

Fingerprint	Bit/Smiles	Feature Structure	Score
ECFP_6	-1925046727	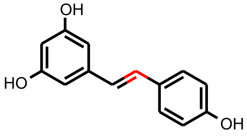 [*]C=[*]	0.145

Top Features for negative contribution

Fingerprint	Bit/Smiles	Feature Structure	Score

ECFP_6	2019062761	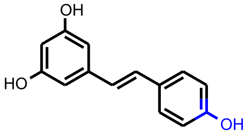 [*]:[c](:[*])O	-0.258
ECFP_6	1996767644	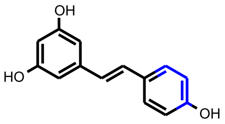 [*][c](:[*]):[cH]:[cH<br>]:[*]	-0.251
ECFP_6	642810091	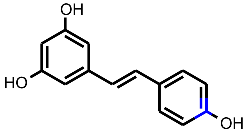 [*][c](:[*]):[*]	-0.247

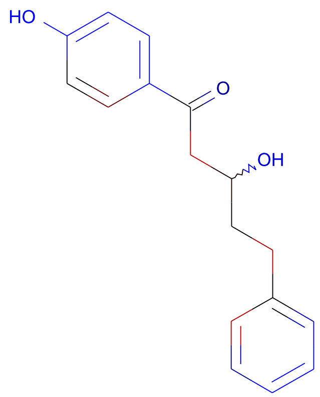

$C_{17}H_{18}O_3$
Molecular Weight: 270.323
ALogP: 3.293
Rotatable Bonds: 6
Acceptors: 3
Donors: 2

Model Prediction

Prediction: 185
Unit: mg/kg_body_weight/day
Mahalanobis Distance: 11
Mahalanobis Distance p-value: 0.00131

Mahalanobis Distance: The Mahalanobis distance (MD) is a generalization of the Euclidean distance that accounts for correlations among the X properties. It is calculated as the distance to the center of the training data. The larger the MD, the less trustworthy the prediction.
Mahalanobis Distance p-value: The p-value gives the fraction of training data with an MD greater than or equal to the one for the given sample, assuming normally distributed data. The smaller the p-value, the less trustworthy the prediciton. For highly non-normal X properties (e.g., fingerprints), the MD p-value is wildly inaccurate.

Structural Similar Compounds

Name	5	Diethylstilbestrol	Cinnamyl anthranilate
Structure	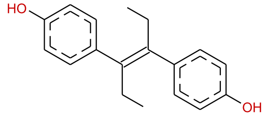	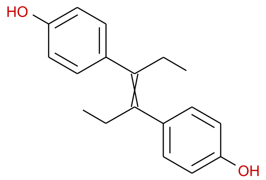	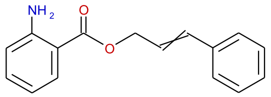
Actual Endpoint (-log C)	6.85816	6.83653	1.99201
Predicted Endpoint (-log C)	3.82521	3.82521	3.01089
Distance	0.565	0.565	0.570
Reference	CPDB	CPDB	CPDB

Model Applicability

Unknown features are fingerprint features in the query molecule, but not found or appearing too infrequently in the training set.

1. All properties and OPS components are within expected ranges.
2. Unknown ECFP_2 feature: -2143661067: [*]CC(=O)[c](:[*]):[*]

Feature Contribution

Top features for positive contribution			
**Fingerprint**	**Bit/Smiles**	**Feature Structure**	**Score**
ECFP_6	1559650422	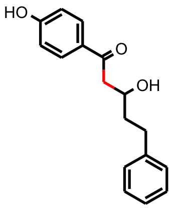 [*]C[*]	0.203

ECFP_6	-2024255407	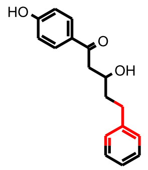 [*]C[c](:[cH]:[*]):[cH]:[*]	0.172
ECFP_6	-175146122	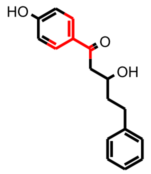 [*]C(=[*])[c](:[cH]:[*]):[cH]:[*]	0.107

Top Features for negative contribution

Fingerprint	Bit/Smiles	Feature Structure	Score
ECFP_6	2019062761	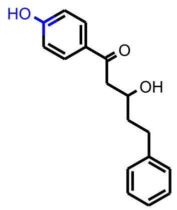 [*]:[c](:[*])O	-0.258
ECFP_6	1996767644	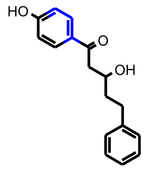 [*][c](:[*]):[cH]:[cH]:[*]	-0.251
ECFP_6	642810091	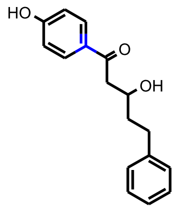 [*][c](:[*]):[*]	-0.247

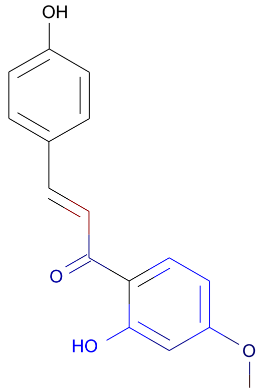

$C_{16}H_{14}O_4$
Molecular Weight: 270.28
ALogP: 3.201
Rotatable Bonds: 4
Acceptors: 4
Donors: 2

Model Prediction

Prediction: 260
Unit: mg/kg_body_weight/day
Mahalanobis Distance: 10.8
Mahalanobis Distance p-value: 0.0026

Mahalanobis Distance: The Mahalanobis distance (MD) is a generalization of the Euclidean distance that accounts for correlations among the X properties. It is calculated as the distance to the center of the training data. The larger the MD, the less trustworthy the prediction.
Mahalanobis Distance p-value: The p-value gives the fraction of training data with an MD greater than or equal to the one for the given sample, assuming normally distributed data. The smaller the p-value, the less trustworthy the prediciton. For highly non-normal X properties (e.g., fingerprints), the MD p-value is wildly inaccurate.

Structural Similar Compounds

Name	44	3,3´-Dimethoxybenzidine.2HCl	422
Structure	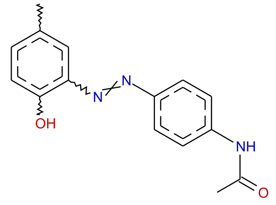	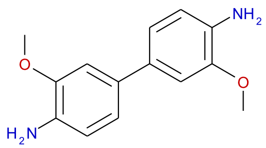	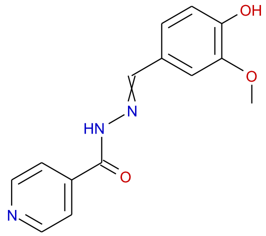
Actual Endpoint (-log C)	2.42163	3.51985	3.99565
Predicted Endpoint (-log C)	2.85113	3.20081	3.22211
Distance	0.497	0.555	0.562
Reference	CPDB	CPDB	CPDB

Model Applicability

Unknown features are fingerprint features in the query molecule, but not found or appearing too infrequently in the training set.

1. All properties and OPS components are within expected ranges.
2. Unknown ECFP_2 feature: 1430764055: [*]=CC(=O)[c](:[*]):[*]

Feature Contribution

Top features for positive contribution			
**Fingerprint**	**Bit/Smiles**	**Feature Structure**	**Score**
ECFP_6	-1925046727	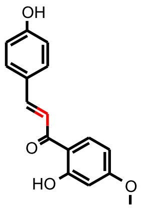 [*]C=[*]	0.145

ECFP_6	-176455838	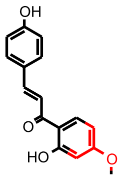 [*]O[c](:[cH]:[*]):[cH]:[*]	0.0818
ECFP_6	734603939	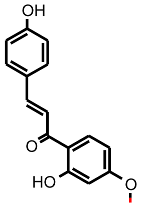 [*]C	0.0424
**Top Features for negative contribution**			
**Fingerprint**	**Bit/Smiles**	**Feature Structure**	**Score**
ECFP_6	2019062761	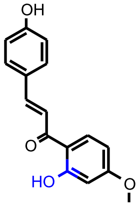 [*]:[c](:[*])O	-0.258
ECFP_6	1996767644	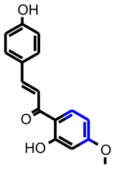 [*][c](:[*]):[cH]:[cH]:[*]	-0.251
ECFP_6	642810091	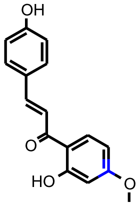 [*][c](:[*]):[*]	-0.247

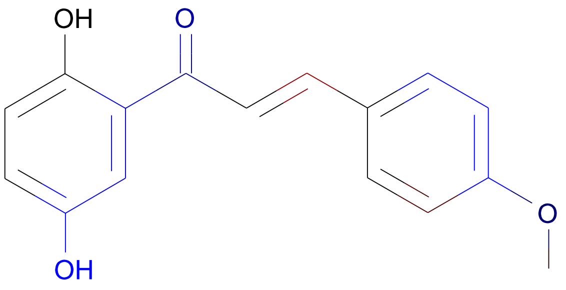

$C_{16}H_{14}O_4$
Molecular Weight: 270.28
ALogP: 3.201
Rotatable Bonds: 4
Acceptors: 4
Donors: 2

Structural Similar Compounds

Name	44	3,3´-Dimethoxybenzidine.2HCl	422
Structure	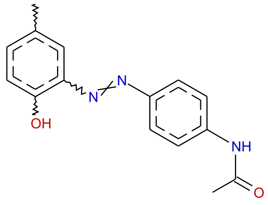	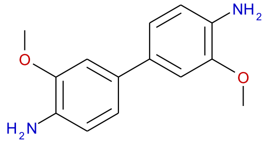	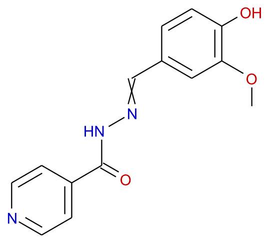
Actual Endpoint (-log C)	2.42163	3.51985	3.99565
Predicted Endpoint (-log C)	2.85113	3.20081	3.22211
Distance	0.491	0.556	0.556
Reference	CPDB	CPDB	CPDB

Model Prediction

Prediction: 260
Unit: mg/kg_body_weight/day
Mahalanobis Distance: 10.8
Mahalanobis Distance p-value: 0.0026

Mahalanobis Distance: The Mahalanobis distance (MD) is a generalization of the Euclidean distance that accounts for correlations among the X properties. It is calculated as the distance to the center of the training data. The larger the MD, the less trustworthy the prediction.
Mahalanobis Distance p-value: The p-value gives the fraction of training data with an MD greater than or equal to the one for the given sample, assuming normally distributed data. The smaller the p-value, the less trustworthy the prediciton. For highly non-normal X properties (e.g., fingerprints), the MD p-value is wildly inaccurate.

Model Applicability

Unknown features are fingerprint features in the query molecule, but not found or appearing too infrequently in the training set.

1. All properties and OPS components are within expected ranges.
2. Unknown ECFP_2 feature: 1430764055: [*]=CC(=O)[c](:[*]):[*]

Feature Contribution

Top features for positive contribution

Fingerprint	Bit/Smiles	Feature Structure	Score
ECFP_6	-1925046727	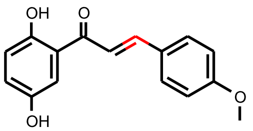 [*]C=[*]	0.145

ECFP_6	-176455838	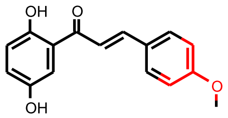 [*]O[c](:[cH]:[*]):[cH]:[*]	0.0818
ECFP_6	734603939	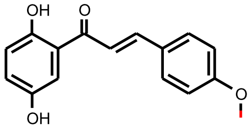 [*]C	0.0424

Top Features for negative contribution

Fingerprint	Bit/Smiles	Feature Structure	Score
ECFP_6	2019062761	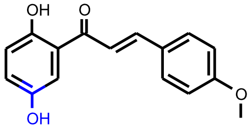 [*]:[c](:[*])O	-0.258
ECFP_6	1996767644	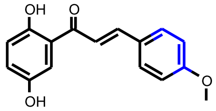 [*][c](:[*]):[cH]:[cH]:[*]	-0.251
ECFP_6	642810091	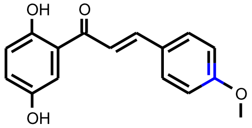 [*][c](:[*]):[*]	-0.247

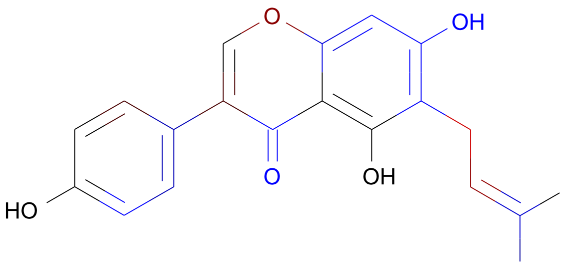

$C_{20}H_{18}O_5$
Molecular Weight: 338.354
ALogP: 3.997
Rotatable Bonds: 3
Acceptors: 5
Donors: 3

Model Prediction

Prediction: 42.6
Unit: mg/kg_body_weight/day
Mahalanobis Distance: 12.4
Mahalanobis Distance p-value: 3.37e-006

Mahalanobis Distance: The Mahalanobis distance (MD) is a generalization of the Euclidean distance that accounts for correlations among the X properties. It is calculated as the distance to the center of the training data. The larger the MD, the less trustworthy the prediction.
Mahalanobis Distance p-value: The p-value gives the fraction of training data with an MD greater than or equal to the one for the given sample, assuming normally distributed data. The smaller the p-value, the less trustworthy the prediciton. For highly non-normal X properties (e.g., fingerprints), the MD p-value is wildly inaccurate.

Structural Similar Compounds

Name	542	Ochratoxin A	44
Structure	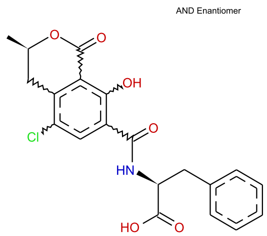	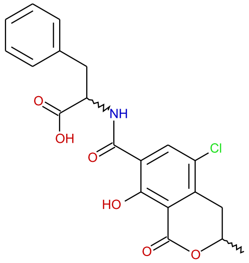	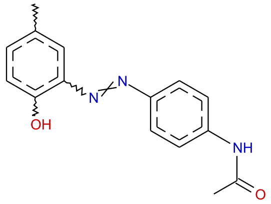
Actual Endpoint (-log C)	4.79932	4.79932	2.42163
Predicted Endpoint (-log C)	3.6353	3.6353	2.85113
Distance	0.626	0.626	0.668
Reference	CPDB	CPDB	CPDB

Model Applicability

Unknown features are fingerprint features in the query molecule, but not found or appearing too infrequently in the training set.

1. OPS PC26 out of range. Value: 3.5057. Training min, max, SD, explained variance: -2.9667, 3.5042, 1.009, 0.0127.
2. Unknown ECFP_2 feature: 1717082529: [*]\C=C(/C(=[*])[*])\[c](:[*]):[*]

Feature Contribution

Top features for positive contribution			
Fingerprint	Bit/Smiles	Feature Structure	Score
ECFP_6	1559650422	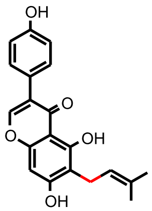 [*]C[*]	0.203

ECFP_6	-1925046727	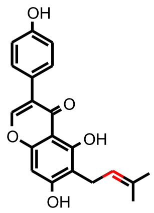 [*]C=[*]	0.145
ECFP_6	683445015	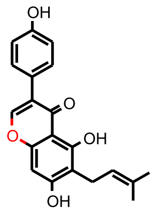 [*]O[*]	0.136

Top Features for negative contribution

Fingerprint	Bit/Smiles	Feature Structure	Score
ECFP_6	2106656448	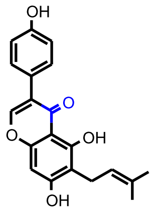 [*]C(=O)[*]	-0.275
ECFP_6	2019062761	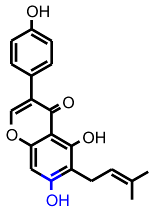 [*]:[c](:[*])O	-0.258
ECFP_6	1996767644	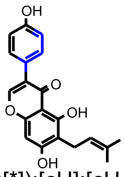 [*][c](:[*]):[cH]:[cH]:[*]	-0.251

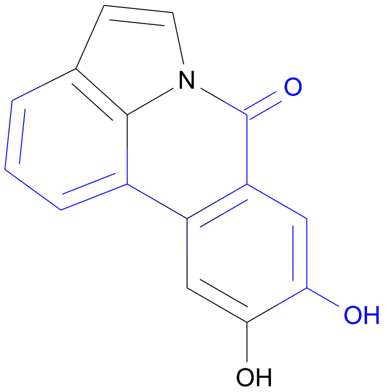

$C_{15}H_9NO_3$
Molecular Weight: 251.237
ALogP: 2.982
Rotatable Bonds: 0
Acceptors: 3
Donors: 2

Model Prediction

Prediction: 142
Unit: mg/kg_body_weight/day
Mahalanobis Distance: 9.39
Mahalanobis Distance p-value: 0.146

Mahalanobis Distance: The Mahalanobis distance (MD) is a generalization of the Euclidean distance that accounts for correlations among the X properties. It is calculated as the distance to the center of the training data. The larger the MD, the less trustworthy the prediction.
Mahalanobis Distance p-value: The p-value gives the fraction of training data with an MD greater than or equal to the one for the given sample, assuming normally distributed data. The smaller the p-value, the less trustworthy the prediciton. For highly non-normal X properties (e.g., fingerprints), the MD p-value is wildly inaccurate.

Structural Similar Compounds

Name	Phenolphthalein	Chrysazin	429
Structure	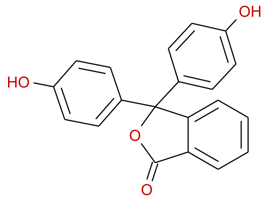	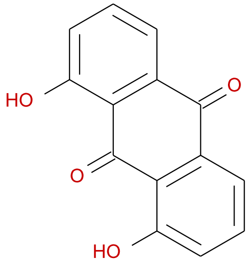	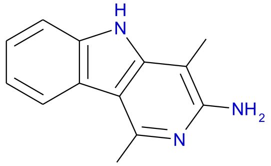
Actual Endpoint (-log C)	2.43468	3.0774	3.71523
Predicted Endpoint (-log C)	3.66084	3.07832	4.22904
Distance	0.595	0.619	0.628
Reference	CPDB	CPDB	CPDB

Model Applicability

Unknown features are fingerprint features in the query molecule, but not found or appearing too infrequently in the training set.

1. All properties and OPS components are within expected ranges.
2. Unknown ECFP_2 feature: 1312166648: [*]C(=[*])n1:[c](:[*]):[*]:[*]:c:1
3. Unknown ECFP_2 feature: 1444648700: [*]:n(:[*])C(=O)[c](:[*]):[*]

Feature Contribution

Top Features for negative contribution

Fingerprint	Bit/Smiles	Feature Structure	Score
ECFP_6	2106656448	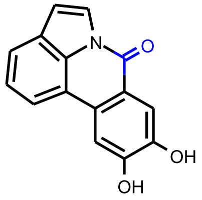 [*]C(=O)[*]	-0.275

ECFP_6	2019062761	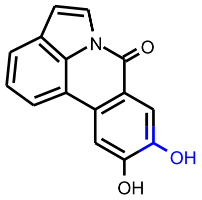 [*]:[c](:[*])O	-0.258
ECFP_6	1996767644	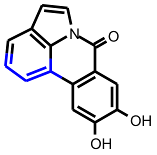 [*][c](:[*]):[cH]:[cH]:[*]	-0.251

remdesivir

TOPKAT_Carcinogenic_Potency_TD50_Mouse

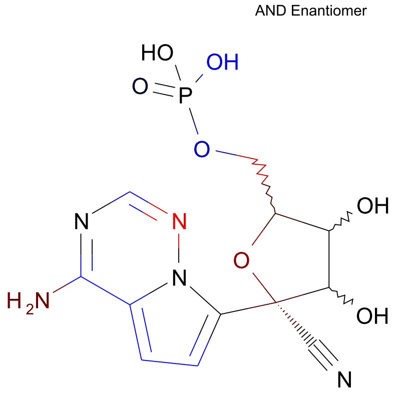

$C_{12}H_{14}N_5O_7P$

Molecular Weight: 371.24262

ALogP: -1.502

Rotatable Bonds: 4

Acceptors: 11

Donors: 5

Model Prediction

Prediction: 9.25

Unit: mg/kg_body_weight/day

Mahalanobis Distance: 14

Mahalanobis Distance p-value: 2.59e-010

Mahalanobis Distance: The Mahalanobis distance (MD) is a generalization of the Euclidean distance that accounts for correlations among the X properties. It is calculated as the distance to the center of the training data. The larger the MD, the less trustworthy the prediction.
Mahalanobis Distance p-value: The p-value gives the fraction of training data with an MD greater than or equal to the one for the given sample, assuming normally distributed data. The smaller the p-value, the less trustworthy the prediciton. For highly non-normal X properties (e.g., fingerprints), the MD p-value is wildly inaccurate.

Structural Similar Compounds

Name	377	(N-6)-(Methylnitroso)adenosine	338
Structure	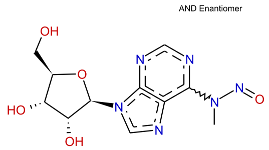	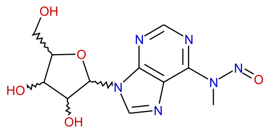	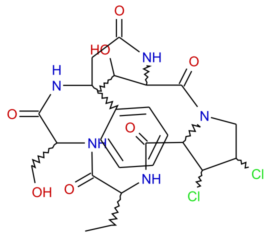
Actual Endpoint (-log C)	4.22928	4.22928	4.39533
Predicted Endpoint (-log C)	5.36013	5.36013	4.31268
Distance	0.852	0.852	0.919
Reference	CPDB	CPDB	CPDB

Model Applicability

Unknown features are fingerprint features in the query molecule, but not found or appearing too infrequently in the training set.

1. All properties and OPS components are within expected ranges.
2. Unknown ECFP_2 feature: 1126642748: [*]OP(=O)(O)O
3. Unknown ECFP_2 feature: 2024329577: [*]P(=[*])([*])O
4. Unknown ECFP_2 feature: -194719409: [*]C1[*][*]C([*])([*])O1
5. Unknown ECFP_2 feature: 1258791451: [*]C1[*][*]O[C@]1(C#[*])[c](:[*]):[*]
6. Unknown ECFP_2 feature: -264833661: [*]C([*])([*])C#N
7. Unknown ECFP_2 feature: -1507082173: [*][c]1:[*]:[*]:[c](:[*]):n:1:n:[*]
8. Unknown ECFP_2 feature: -676555381: [*]:[cH]:n:n(:[*]):[*]
9. Unknown ECFP_2 feature: -66263742: [*]C([*])([*])[c]1:[cH]:[*]:[*]:n:1:[*]

Feature Contribution

Top features for positive contribution

Fingerprint	Bit/Smiles	Feature Structure	Score

ECFP_6	655739385	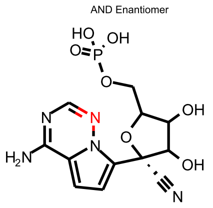 [*]:n:[*]	0.229
ECFP_6	1572579716	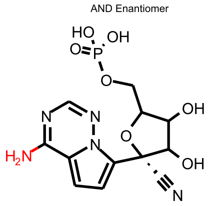 [*]N	0.225
ECFP_6	1559650422	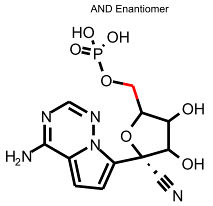 [*]C[*]	0.203

Top Features for negative contribution

Fingerprint	Bit/Smiles	Feature Structure	Score
ECFP_6	1996767644	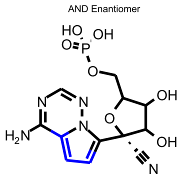 [*][c](:[*]):[cH]:[cH]:[*]	-0.251
ECFP_6	642810091	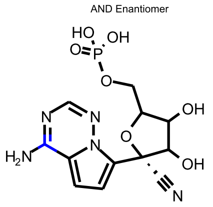 [*][c](:[*]):[*]	-0.247

ECFP_6	-182236392	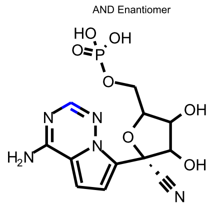 [*]:[cH]:[*]	-0.232

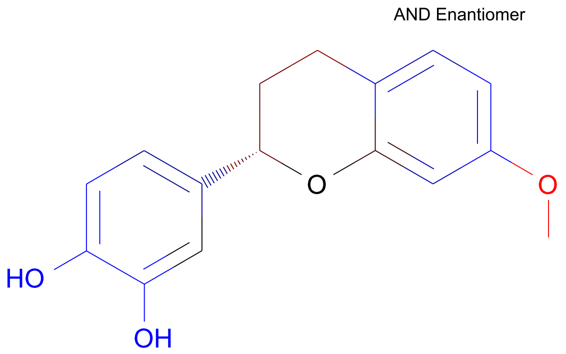

$C_{16}H_{16}O_4$
Molecular Weight: 272.296
ALogP: 3.456
Rotatable Bonds: 2
Acceptors: 4
Donors: 2

Model Prediction

Prediction: 9.18
Unit: mg/kg_body_weight/day
Mahalanobis Distance: 10.9
Mahalanobis Distance p-value: 0.0229

Mahalanobis Distance: The Mahalanobis distance (MD) is a generalization of the Euclidean distance that accounts for correlations among the X properties. It is calculated as the distance to the center of the training data. The larger the MD, the less trustworthy the prediction.
Mahalanobis Distance p-value: The p-value gives the fraction of training data with an MD greater than or equal to the one for the given sample, assuming normally distributed data. The smaller the p-value, the less trustworthy the prediciton. For highly non-normal X properties (e.g., fingerprints), the MD p-value is wildly inaccurate.

Structural Similar Compounds

Name	44	Chrysazin	Nafenopin s
Structure	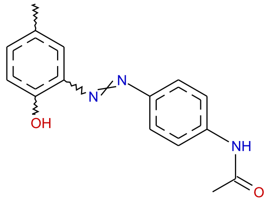	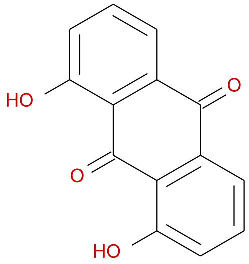	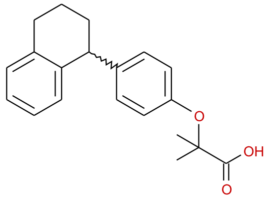
Actual Endpoint (-log C)	2.85045	2.99143	4.45051
Predicted Endpoint (-log C)	2.7768	3.29868	3.8403
Distance	0.545	0.562	0.598
Reference	CPDB	CPDB	CPDB

Model Applicability

Unknown features are fingerprint features in the query molecule, but not found or appearing too infrequently in the training set.

1. All properties and OPS components are within expected ranges.

Feature Contribution

Top features for positive contribution			
**Fingerprint**	**Bit/Smiles**	**Feature Structure**	**Score**
FCFP_6	136627117	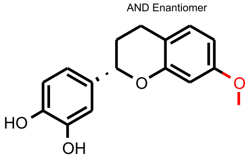 [*]OC	0.69

FCFP_6	1	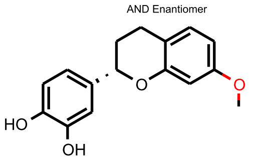<br>[*]O[*]	0.234
FCFP_6	203677720	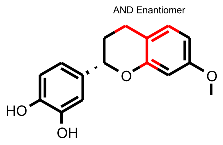<br>[*]C[c](:[cH]:[*]):[c<br>]([*]):[*]	0.137

Top Features for negative contribution

Fingerprint	Bit/Smiles	Feature Structure	Score
FCFP_6	7	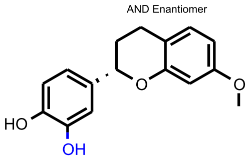<br>[*]O	-0.372
FCFP_6	16	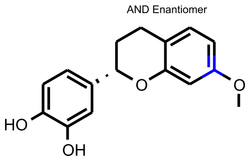<br>[*][c](:[*]):[*]	-0.354
FCFP_6	74595001	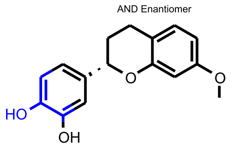<br>[*][c](:[*]):[c](O):[<br>cH]:[*]	-0.267

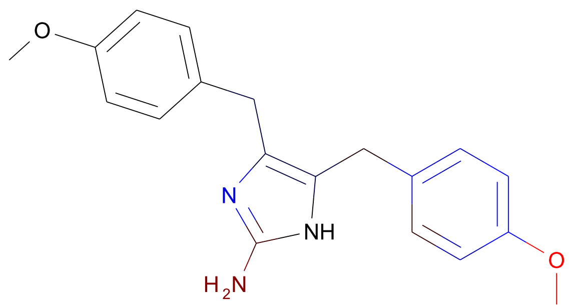

$C_{19}H_{21}N_3O_2$
Molecular Weight: 323.389
ALogP: 3.305
Rotatable Bonds: 6
Acceptors: 4
Donors: 2

Model Prediction

Prediction: 3.94
Unit: mg/kg_body_weight/day
Mahalanobis Distance: 13.3
Mahalanobis Distance p-value: 1.3e-006

Mahalanobis Distance: The Mahalanobis distance (MD) is a generalization of the Euclidean distance that accounts for correlations among the X properties. It is calculated as the distance to the center of the training data. The larger the MD, the less trustworthy the prediction.
Mahalanobis Distance p-value: The p-value gives the fraction of training data with an MD greater than or equal to the one for the given sample, assuming normally distributed data. The smaller the p-value, the less trustworthy the prediciton. For highly non-normal X properties (e.g., fingerprints), the MD p-value is wildly inaccurate.

Structural Similar Compounds

Name	Omeprazole	796	Indomethacin
Structure	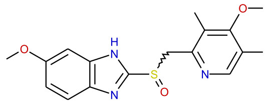	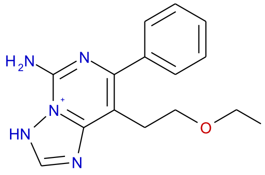	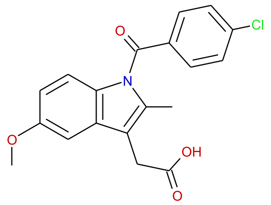
Actual Endpoint (-log C)	3.4628	2.71505	5.49293
Predicted Endpoint (-log C)	4.7324	4.45918	4.9569
Distance	0.564	0.568	0.579
Reference	CPDB	CPDB	CPDB

Model Applicability

Unknown features are fingerprint features in the query molecule, but not found or appearing too infrequently in the training set.

1. All properties and OPS components are within expected ranges.

Feature Contribution

Top features for positive contribution

Fingerprint	Bit/Smiles	Feature Structure	Score
FCFP_6	136627117	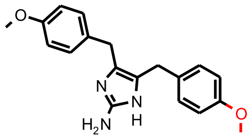 [*]OC	0.69

FCFP_6	1	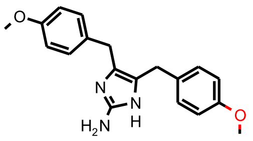 [*]O[*]	0.234
FCFP_6	203677720	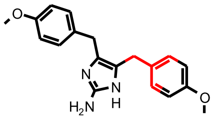 [*]C[c](:[cH]:[*]):[c]([*]):[*]	0.137
**Top Features for negative contribution**			
**Fingerprint**	**Bit/Smiles**	**Feature Structure**	**Score**
FCFP_6	16	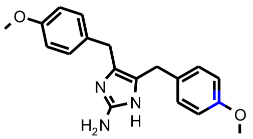 [*][c](:[*]):[*]	-0.354
FCFP_6	1674451008	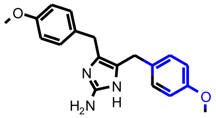 [*]O[c]1:[cH]:[*]:[c]([*]):[cH]:[cH]:1	-0.233
FCFP_6	17	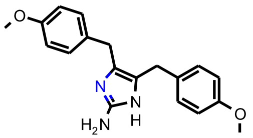 [*]:n:[*]	-0.149

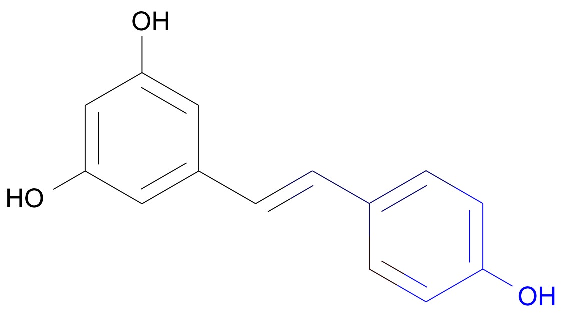

$C_{14}H_{12}O_3$
Molecular Weight: 228.243
ALogP: 3.09
Rotatable Bonds: 2
Acceptors: 3
Donors: 3

Model Prediction

Prediction: 112
Unit: mg/kg_body_weight/day
Mahalanobis Distance: 6.98
Mahalanobis Distance p-value: 1

Mahalanobis Distance: The Mahalanobis distance (MD) is a generalization of the Euclidean distance that accounts for correlations among the X properties. It is calculated as the distance to the center of the training data. The larger the MD, the less trustworthy the prediction.
Mahalanobis Distance p-value: The p-value gives the fraction of training data with an MD greater than or equal to the one for the given sample, assuming normally distributed data. The smaller the p-value, the less trustworthy the prediciton. For highly non-normal X properties (e.g., fingerprints), the MD p-value is wildly inaccurate.

Structural Similar Compounds

Name	Diethylstilbestrol	5	Caffeic acid
Structure	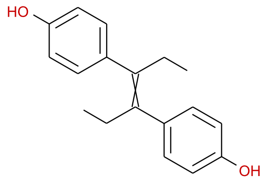	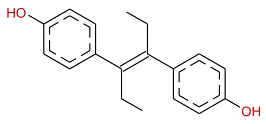	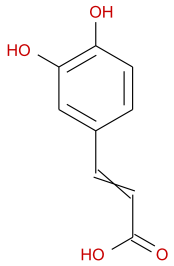
Actual Endpoint (-log C)	6.0804	6.0804	2.7829
Predicted Endpoint (-log C)	3.53032	3.53032	2.77347
Distance	0.550	0.550	0.563
Reference	CPDB	CPDB	CPDB

Model Applicability

Unknown features are fingerprint features in the query molecule, but not found or appearing too infrequently in the training set.

1. All properties and OPS components are within expected ranges.

Feature Contribution

Top features for positive contribution

Fingerprint	Bit/Smiles	Feature Structure	Score
FCFP_6	203677720	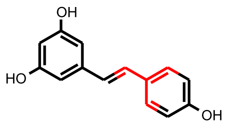 [*]C[c](:[cH]:[*]):[c]([*]):[*]	0.137

Top Features for negative contribution

Fingerprint	Bit/Smiles	Feature Structure	Score

FCFP_6	7	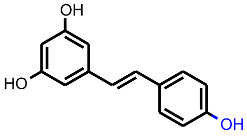 [*]O	-0.372
FCFP_6	16	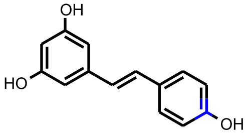 [*][c](:[*]):[*]	-0.354
FCFP_6	74595001	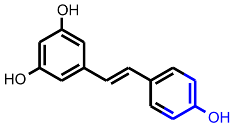 [*][c](:[*]):[c](O):[cH]:[*]	-0.267

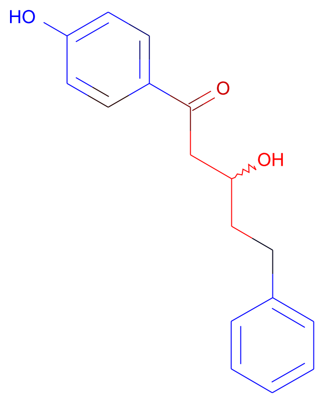

$C_{17}H_{18}O_3$
Molecular Weight: 270.323
ALogP: 3.293
Rotatable Bonds: 6
Acceptors: 3
Donors: 2

Structural Similar Compounds

Name	Diethylstilbestrol	5	Cinnamyl anthranilate
Structure	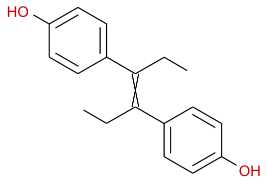	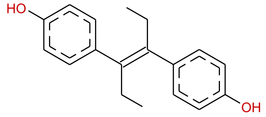	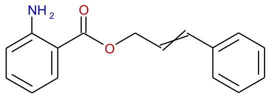
Actual Endpoint (-log C)	6.0804	6.0804	1.32084
Predicted Endpoint (-log C)	3.53032	3.53032	2.6022
Distance	0.536	0.536	0.548
Reference	CPDB	CPDB	CPDB

Model Applicability

Unknown features are fingerprint features in the query molecule, but not found or appearing too infrequently in the training set.

1. OPS PC10 out of range. Value: 6.4011. Training min, max, SD, explained variance: -5.5482, 6.0958, 1.698, 0.0277.

Model Prediction

Prediction: 39.9
Unit: mg/kg_body_weight/day
Mahalanobis Distance: 11.5
Mahalanobis Distance p-value: 0.00323

Mahalanobis Distance: The Mahalanobis distance (MD) is a generalization of the Euclidean distance that accounts for correlations among the X properties. It is calculated as the distance to the center of the training data. The larger the MD, the less trustworthy the prediction.
Mahalanobis Distance p-value: The p-value gives the fraction of training data with an MD greater than or equal to the one for the given sample, assuming normally distributed data. The smaller the p-value, the less trustworthy the prediciton. For highly non-normal X properties (e.g., fingerprints), the MD p-value is wildly inaccurate.

Feature Contribution

Top features for positive contribution

Fingerprint	Bit/Smiles	Feature Structure	Score
FCFP_6	-1043250487	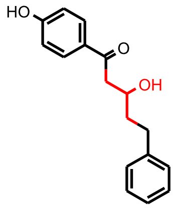 [*]CC(O)C[*]	1.15

FCFP_6	1	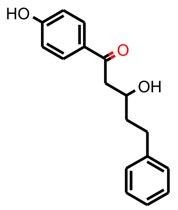 [*]O[*]	0.234
FCFP_6	203677720	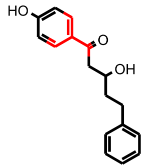 [*]C[c](:[cH]:[*]):[c]([*]):[*]	0.137
**Top Features for negative contribution**			
**Fingerprint**	**Bit/Smiles**	**Feature Structure**	**Score**
FCFP_6	991735244	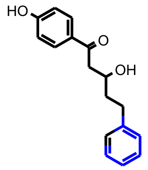 [*][c]1:[*]:[cH]:[cH]:[cH]:[cH]:1	-0.422
FCFP_6	-2093839777	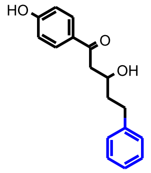 [*][c]1:[cH]:[cH]:[cH]:[cH]:[cH]:1	-0.378
FCFP_6	7	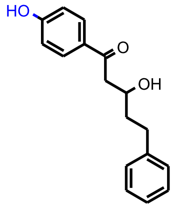 [*]O	-0.372

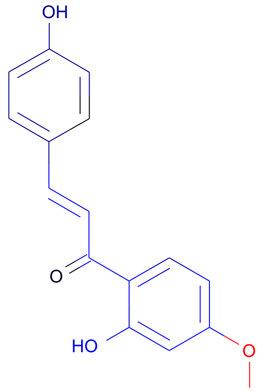

$C_{16}H_{14}O_4$
Molecular Weight: 270.28
ALogP: 3.201
Rotatable Bonds: 4
Acceptors: 4
Donors: 2

Model Prediction

Prediction: 112
Unit: mg/kg_body_weight/day
Mahalanobis Distance: 9.41
Mahalanobis Distance p-value: 0.469

Mahalanobis Distance: The Mahalanobis distance (MD) is a generalization of the Euclidean distance that accounts for correlations among the X properties. It is calculated as the distance to the center of the training data. The larger the MD, the less trustworthy the prediction.
Mahalanobis Distance p-value: The p-value gives the fraction of training data with an MD greater than or equal to the one for the given sample, assuming normally distributed data. The smaller the p-value, the less trustworthy the prediciton. For highly non-normal X properties (e.g., fingerprints), the MD p-value is wildly inaccurate.

Structural Similar Compounds

Name	44	3,3´-Dimethoxybenzidine.2HCl	2,5-Dimethoxy-4´-aminostilbene
Structure	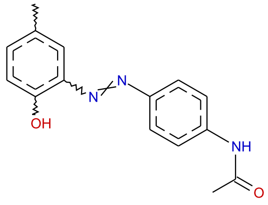	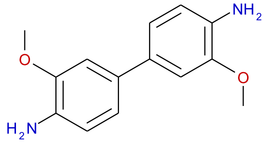	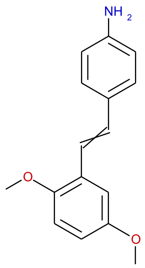
Actual Endpoint (-log C)	2.85045	5.37087	5.54914
Predicted Endpoint (-log C)	2.7768	4.89028	4.63197
Distance	0.477	0.539	0.549
Reference	CPDB	CPDB	CPDB

Model Applicability

Unknown features are fingerprint features in the query molecule, but not found or appearing too infrequently in the training set.

1. All properties and OPS components are within expected ranges.

Feature Contribution

Top features for positive contribution

Fingerprint	Bit/Smiles	Feature Structure	Score
FCFP_6	136627117	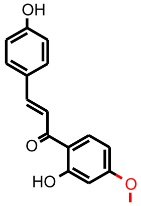 [*]OC	0.69

FCFP_6	1	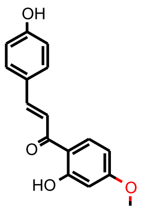 [*]O[*]	0.234
FCFP_6	203677720	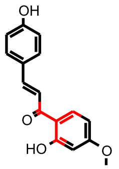 [*]C[c](:[cH]:[*]):[c]([*]):[*]	0.137
**Top Features for negative contribution**			
**Fingerprint**	**Bit/Smiles**	**Feature Structure**	**Score**
FCFP_6	451847724	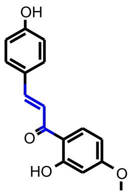 [*]\C=C\C(=[*])[*]	-0.436
FCFP_6	7	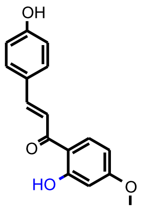 [*]O	-0.372
FCFP_6	16	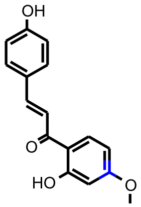 [*][c](:[*]):[*]	-0.354

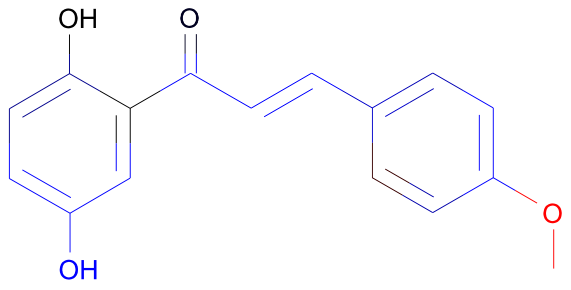

$C_{16}H_{14}O_4$
Molecular Weight: 270.28
ALogP: 3.201
Rotatable Bonds: 4
Acceptors: 4
Donors: 2

Model Prediction

Prediction: 112
Unit: mg/kg_body_weight/day
Mahalanobis Distance: 9.41
Mahalanobis Distance p-value: 0.469

Mahalanobis Distance: The Mahalanobis distance (MD) is a generalization of the Euclidean distance that accounts for correlations among the X properties. It is calculated as the distance to the center of the training data. The larger the MD, the less trustworthy the prediction.
Mahalanobis Distance p-value: The p-value gives the fraction of training data with an MD greater than or equal to the one for the given sample, assuming normally distributed data. The smaller the p-value, the less trustworthy the prediciton. For highly non-normal X properties (e.g., fingerprints), the MD p-value is wildly inaccurate.

Structural Similar Compounds

Name	44	3,3´-Dimethoxybenzidine.2HCl	2,5-Dimethoxy-4´-aminostilbene
Structure	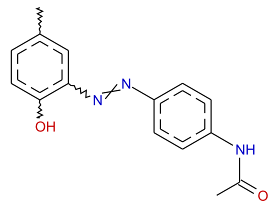	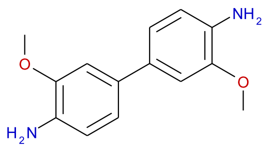	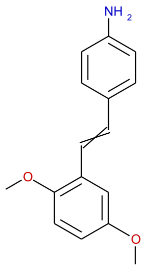
Actual Endpoint (-log C)	2.85045	5.37087	5.54914
Predicted Endpoint (-log C)	2.7768	4.89028	4.63197
Distance	0.479	0.540	0.551
Reference	CPDB	CPDB	CPDB

Model Applicability

Unknown features are fingerprint features in the query molecule, but not found or appearing too infrequently in the training set.

1. All properties and OPS components are within expected ranges.

Feature Contribution

Top features for positive contribution			
**Fingerprint**	**Bit/Smiles**	**Feature Structure**	**Score**
FCFP_6	136627117	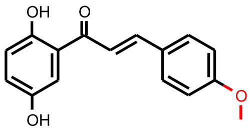 [*]OC	0.69

FCFP_6	1	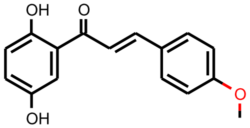 [*]O[*]	0.234
FCFP_6	203677720	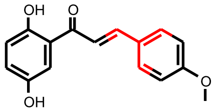 [*]C[c](:[cH]:[*]):[c]([*]):[*]	0.137
**Top Features for negative contribution**			
**Fingerprint**	**Bit/Smiles**	**Feature Structure**	**Score**
FCFP_6	451847724	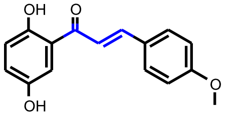 [*]\C=C\C(=[*])[*]	-0.436
FCFP_6	7	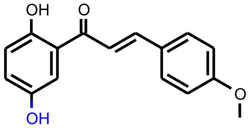 [*]O	-0.372
FCFP_6	16	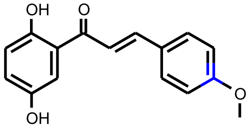 [*][c](:[*]):[*]	-0.354

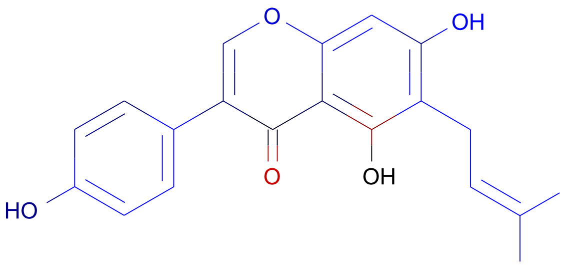

$C_{20}H_{18}O_5$
Molecular Weight: 338.354
ALogP: 3.997
Rotatable Bonds: 3
Acceptors: 5
Donors: 3

Model Prediction

Prediction: 323
Unit: mg/kg_body_weight/day
Mahalanobis Distance: 12.7
Mahalanobis Distance p-value: 2.6e-005

Mahalanobis Distance: The Mahalanobis distance (MD) is a generalization of the Euclidean distance that accounts for correlations among the X properties. It is calculated as the distance to the center of the training data. The larger the MD, the less trustworthy the prediction.
Mahalanobis Distance p-value: The p-value gives the fraction of training data with an MD greater than or equal to the one for the given sample, assuming normally distributed data. The smaller the p-value, the less trustworthy the prediciton. For highly non-normal X properties (e.g., fingerprints), the MD p-value is wildly inaccurate.

Structural Similar Compounds

Name	542	Ochratoxin A	44
Structure	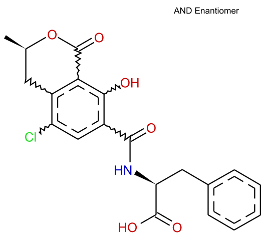	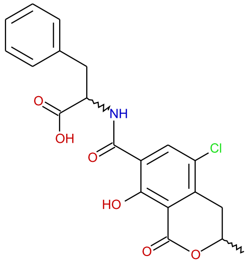	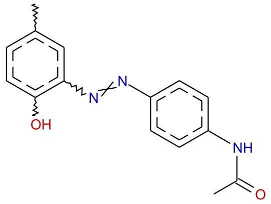
Actual Endpoint (-log C)	6.59334	6.47264	2.85045
Predicted Endpoint (-log C)	5.06501	5.06501	2.7768
Distance	0.618	0.618	0.632
Reference	CPDB	CPDB	CPDB

Model Applicability

Unknown features are fingerprint features in the query molecule, but not found or appearing too infrequently in the training set.

1. All properties and OPS components are within expected ranges.

Feature Contribution

Top features for positive contribution

Fingerprint	Bit/Smiles	Feature Structure	Score
FCFP_6	1	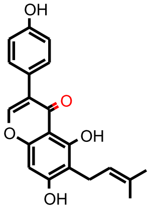 [*]O[*]	0.234

FCFP_6	203677720	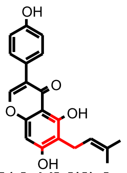 [*]C[c](:[cH]:[*]):[c]([*]):[*]	0.137
FCFP_6	136597326	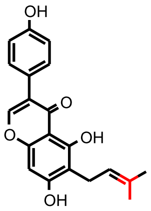 [*]C(=[*])C	0.0695

Top Features for negative contribution

Fingerprint	Bit/Smiles	Feature Structure	Score
FCFP_6	451847724	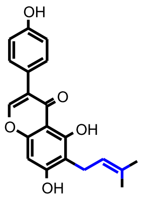 [*]\C=C\C(=[*])[*]	-0.436
FCFP_6	436886043	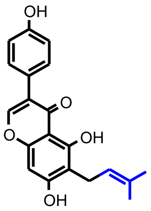 [*]C=C(C)C	-0.383
FCFP_6	7	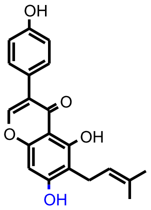 [*]O	-0.372

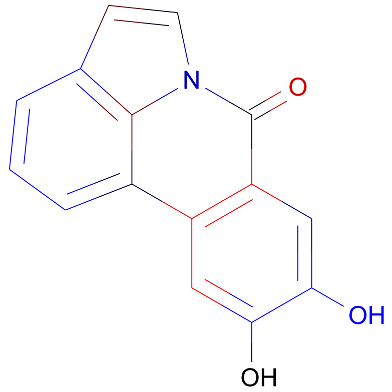

$C_{15}H_9NO_3$
Molecular Weight: 251.237
ALogP: 2.982
Rotatable Bonds: 0
Acceptors: 3
Donors: 2

Model Prediction

Prediction: 14
Unit: mg/kg_body_weight/day
Mahalanobis Distance: 16.9
Mahalanobis Distance p-value: 2.51e-017

Mahalanobis Distance: The Mahalanobis distance (MD) is a generalization of the Euclidean distance that accounts for correlations among the X properties. It is calculated as the distance to the center of the training data. The larger the MD, the less trustworthy the prediction.
Mahalanobis Distance p-value: The p-value gives the fraction of training data with an MD greater than or equal to the one for the given sample, assuming normally distributed data. The smaller the p-value, the less trustworthy the prediciton. For highly non-normal X properties (e.g., fingerprints), the MD p-value is wildly inaccurate.

Structural Similar Compounds

Name	Phenolphthalein	429	Chrysazin
Structure	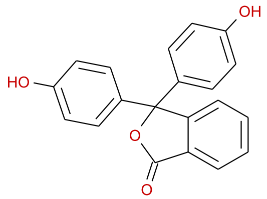	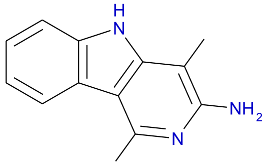	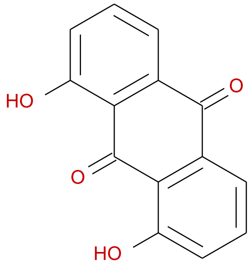
Actual Endpoint (-log C)	2.54766	5.56515	2.99143
Predicted Endpoint (-log C)	3.7508	4.07595	3.29868
Distance	0.588	0.593	0.599
Reference	CPDB	CPDB	CPDB

Model Applicability

Unknown features are fingerprint features in the query molecule, but not found or appearing too infrequently in the training set.

1. All properties and OPS components are within expected ranges.

Feature Contribution

Top features for positive contribution

Fingerprint	Bit/Smiles	Feature Structure	Score
FCFP_6	-1861645784	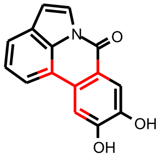 [*][c](:[*]):[c](:[cH]:[*])[c](:[*]):[*]	0.359

FCFP_6	1	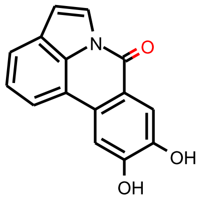 [*]O[*]	0.234
FCFP_6	203677720	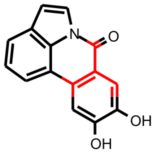 [*]C[c](:[cH]:[*]):[c]([*]):[*]	0.137
**Top Features for negative contribution**			
**Fingerprint**	**Bit/Smiles**	**Feature Structure**	**Score**
FCFP_6	991735244	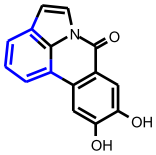 [*][c]1:[*]:[cH]:[cH] :[cH]:[cH]:1	-0.422
FCFP_6	7	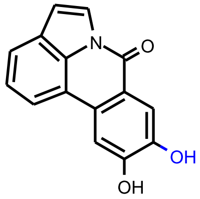 [*]O	-0.372
FCFP_6	16	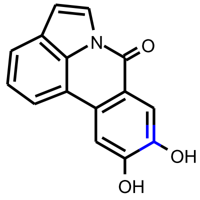 [*][c](:[*]):[*]	-0.354

remdesivir

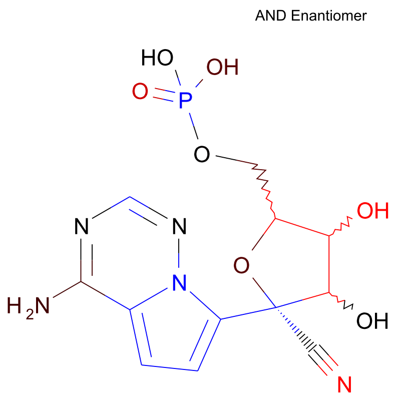

$C_{12}H_{14}N_5O_7P$
Molecular Weight: 371.24262
ALogP: -1.502
Rotatable Bonds: 4
Acceptors: 11
Donors: 5

Model Prediction

Prediction: 1.01
Unit: mg/kg_body_weight/day
Mahalanobis Distance: 16.2
Mahalanobis Distance p-value: 4.38e-015

Mahalanobis Distance: The Mahalanobis distance (MD) is a generalization of the Euclidean distance that accounts for correlations among the X properties. It is calculated as the distance to the center of the training data. The larger the MD, the less trustworthy the prediction.
Mahalanobis Distance p-value: The p-value gives the fraction of training data with an MD greater than or equal to the one for the given sample, assuming normally distributed data. The smaller the p-value, the less trustworthy the prediciton. For highly non-normal X properties (e.g., fingerprints), the MD p-value is wildly inaccurate.

Structural Similar Compounds

Name	b-Thioguanine deoxyriboside	Hexamethylmelamine	604
Structure	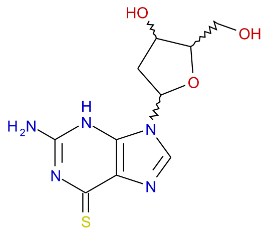	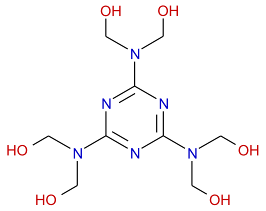	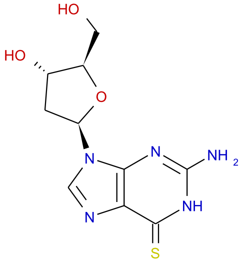
Actual Endpoint (-log C)	5.13004	4.47751	5.13004
Predicted Endpoint (-log C)	4.82552	3.76275	4.96687
Distance	0.805	0.832	0.835
Reference	CPDB	CPDB	CPDB

Model Applicability

Unknown features are fingerprint features in the query molecule, but not found or appearing too infrequently in the training set.

1. All properties and OPS components are within expected ranges.
2. Unknown FCFP_2 feature: 472180098: [*]OP(=O)(O)O
3. Unknown FCFP_2 feature: -836603894: [*]C1[*][*]O[C@]1(C#[*])[c](:[*]):[*]

Feature Contribution

Top features for positive contribution

Fingerprint	Bit/Smiles	Feature Structure	Score
FCFP_6	-1043250487	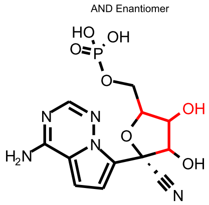 [*]CC(O)C[*]	1.15

FCFP_6	9	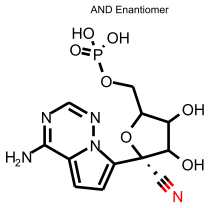 [*]#N	0.385
FCFP_6	1	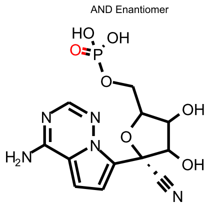 [*]O[*]	0.234

Top Features for negative contribution			
**Fingerprint**	**Bit/Smiles**	**Feature Structure**	**Score**
FCFP_6	-1280036918	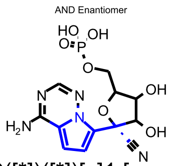 [*]C([*])([*])[c]1:[cH]:[cH]:[c](:[*]):n:1:[*]	-0.363
FCFP_6	16	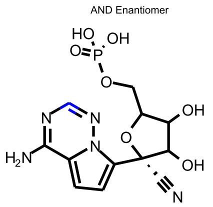 [*][c](:[*]):[*]	-0.354
FCFP_6	17	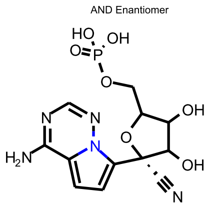 [*]:n:[*]	-0.149

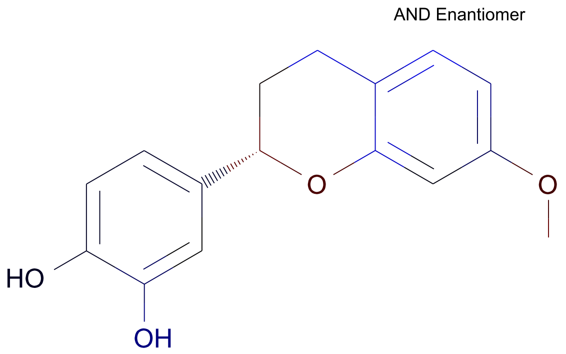

$C_{16}H_{16}O_4$
Molecular Weight: 272.296
ALogP: 3.456
Rotatable Bonds: 2
Acceptors: 4
Donors: 2

Model Prediction

Prediction: 0.0522
Unit: g/kg_body_weight
Mahalanobis Distance: 23.4
Mahalanobis Distance p-value: 9.11e-013

Mahalanobis Distance: The Mahalanobis distance (MD) is a generalization of the Euclidean distance that accounts for correlations among the X properties. It is calculated as the distance to the center of the training data. The larger the MD, the less trustworthy the prediction.
Mahalanobis Distance p-value: The p-value gives the fraction of training data with an MD greater than or equal to the one for the given sample, assuming normally distributed data. The smaller the p-value, the less trustworthy the prediciton. For highly non-normal X properties (e.g., fingerprints), the MD p-value is wildly inaccurate.

Structural Similar Compounds

Name	HC BLUE 1	LORAZEPAM	ETODOLAC
Structure	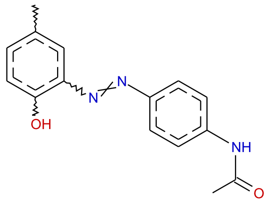	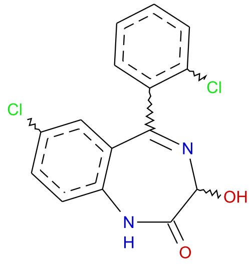	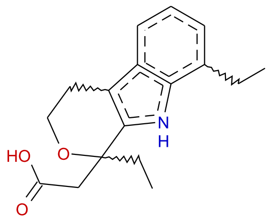
Actual Endpoint (-log C)	3.0323	3.7286	4.9813
Predicted Endpoint (-log C)	2.7171	2.8638	4.39289
Distance	0.477	0.524	0.528
Reference	NTP REPORT # 222	NDA-17794	NDA-18922

Model Applicability

Unknown features are fingerprint features in the query molecule, but not found or appearing too infrequently in the training set.

1. All properties and OPS components are within expected ranges.
2. Unknown ECFP_6 feature: 1307307440: [*]:[c](:[*])OC
3. Unknown ECFP_6 feature: 1334973442: [*]C[c](:[cH]:[*]):[c]([*]):[*]
4. Unknown ECFP_6 feature: -570915357: [*]O[c](:[cH]:[*]):[c]([*]):[*]
5. Unknown ECFP_6 feature: -555098937: [*]C([*])O[c](:[*]):[*]
6. Unknown ECFP_6 feature: -856154029: [*]C[C@H](O[*])[c](:[*]):[*]
7. Unknown ECFP_6 feature: 51876938: [*]CC[c](:[*]):[*]
8. Unknown ECFP_6 feature: -175882072: [*]C([*])[c](:[cH]:[*]):[cH]:[*]
9. Unknown ECFP_6 feature: 2019062761: [*]:[c](:[*])O

Feature Contribution

Top features for positive contribution

Fingerprint	Bit/Smiles	Feature Structure	Score

ECFP_6	-167460056	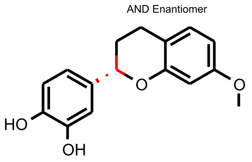<br>[*]C([*])[*]	0.136
ECFP_6	-176455838	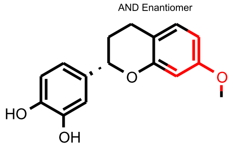<br>[*]O[c](:[cH]:[*]):[cH]:[*]	0.106
ECFP_6	683445015	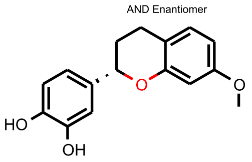<br>[*]O[*]	0.0734
**Top Features for negative contribution**			
**Fingerprint**	**Bit/Smiles**	**Feature Structure**	**Score**
FCFP_6	1	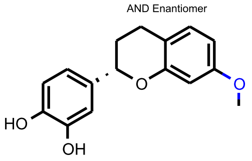<br>[*]O[*]	-0.102
FCFP_6	-453677277	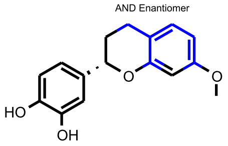<br>[*]C[c]1:[cH]:[cH]:[c](([*]):[*]:[c]:1[*]	-0.0906

FCFP_6	203677720	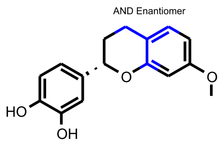<br>[*]C[c](:[cH]:[*]):[c<br>]([*]):[*]	-0.0713

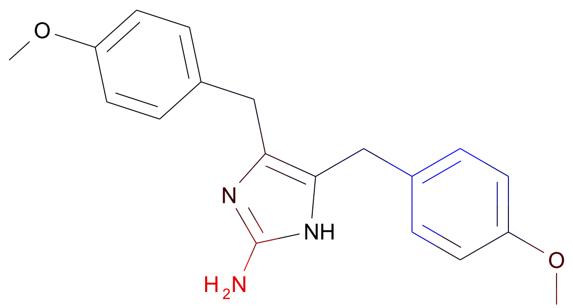

$C_{19}H_{21}N_3O_2$
Molecular Weight: 323.389
ALogP: 3.305
Rotatable Bonds: 6
Acceptors: 4
Donors: 2

Structural Similar Compounds

Name	ISOXABEN	ETODOLAC	3;3'-DIMETHOXYBENZIDINE .2HCL
Structure	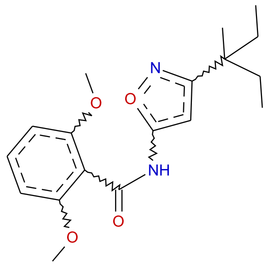	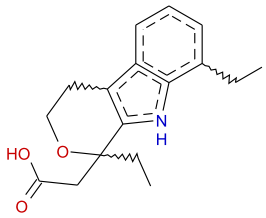	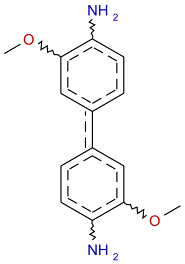
Actual Endpoint (-log C)	3.81665	4.9813	4.79463
Predicted Endpoint (-log C)	4.42315	4.39289	3.61371
Distance	0.592	0.625	0.654
Reference	EPA COVER SHEET 0339;881201;(1)	NDA-18922	NTP REPORT # 372

Model Prediction

Prediction: 0.0147
Unit: g/kg_body_weight
Mahalanobis Distance: 28.6
Mahalanobis Distance p-value: 3.96e-022

Mahalanobis Distance: The Mahalanobis distance (MD) is a generalization of the Euclidean distance that accounts for correlations among the X properties. It is calculated as the distance to the center of the training data. The larger the MD, the less trustworthy the prediction.
Mahalanobis Distance p-value: The p-value gives the fraction of training data with an MD greater than or equal to the one for the given sample, assuming normally distributed data. The smaller the p-value, the less trustworthy the prediciton. For highly non-normal X properties (e.g., fingerprints), the MD p-value is wildly inaccurate.

Model Applicability

Unknown features are fingerprint features in the query molecule, but not found or appearing too infrequently in the training set.

1. All properties and OPS components are within expected ranges.
2. Unknown FCFP_2 feature: -1151854667: N[c]1:[nH]:[*]:[*]:n:1
3. Unknown ECFP_6 feature: -152683720: [*]:[nH]:[*]
4. Unknown ECFP_6 feature: 1307307440: [*]:[c](:[*])OC
5. Unknown ECFP_6 feature: 765434811: [*]:[c](:[*])C[c](:[*]):[*]
6. Unknown ECFP_6 feature: -746759483: [*]C[c]1:[nH]:[*]:[*]:[c]:1[*]
7. Unknown ECFP_6 feature: 558201926: [*][c]1:[*]:[*]:[c]([*]):[nH]:1
8. Unknown ECFP_6 feature: -2046255371: N[c]1:[nH]:[*]:[*]:n:1
9. Unknown ECFP_6 feature: -435942924: [*]C[c]1:n:[*]:[*]:[c]:1[*]
10. Unknown ECFP_6 feature: -938530932: [*]:[c](:[*])N

Feature Contribution

Top features for positive contribution			
Fingerprint	Bit/Smiles	Feature Structure	Score

ECFP_6	1559650422	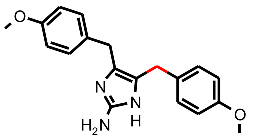<br>[*]C[*]	0.129
ECFP_6	-176455838	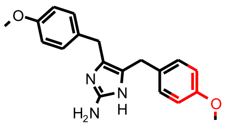<br>[*]O[c](:[cH]:[*]):[c<br>H]:[*]	0.106
FCFP_6	3	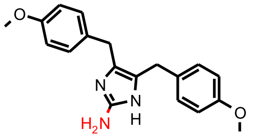<br>[*]N	0.0924
**Top Features for negative contribution**			
**Fingerprint**	**Bit/Smiles**	**Feature Structure**	**Score**
FCFP_6	1	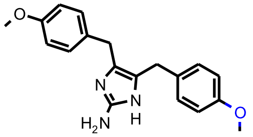<br>[*]O[*]	-0.102
FCFP_6	-453677277	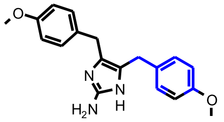<br>[*]C[c]1:[cH]:[cH]:[c<br>]([*]):[*]:[c]:1[*]	-0.0906

FCFP_6	203677720	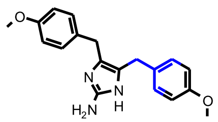 [*]C[c](:[cH]:[*]):[c]([*]):[*]	-0.0713

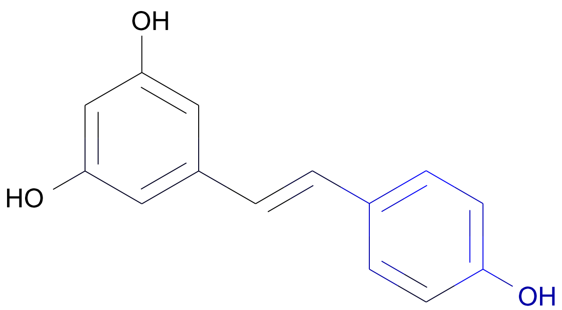

$C_{14}H_{12}O_3$
Molecular Weight: 228.243
ALogP: 3.09
Rotatable Bonds: 2
Acceptors: 3
Donors: 3

Structural Similar Compounds

Name	BISPHENOL A	4;4'-OXYDIANILINE	HC BLUE 1
Structure	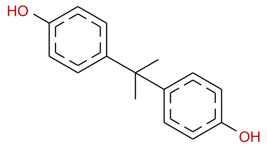	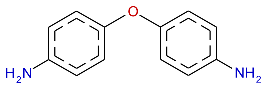	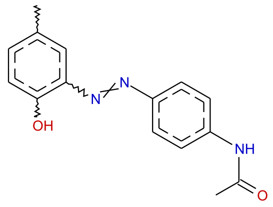
Actual Endpoint (-log C)	3.6595	4.3016	3.0323
Predicted Endpoint (-log C)	3.33364	3.80404	2.7171
Distance	0.482	0.573	0.583
Reference	EPA COVER SHEET 0356;890901;(1)	NTP REPORT # 205	NTP REPORT # 222

Model Prediction

Prediction: 0.089
Unit: g/kg_body_weight
Mahalanobis Distance: 16.8
Mahalanobis Distance p-value: 0.00506

Mahalanobis Distance: The Mahalanobis distance (MD) is a generalization of the Euclidean distance that accounts for correlations among the X properties. It is calculated as the distance to the center of the training data. The larger the MD, the less trustworthy the prediction.
Mahalanobis Distance p-value: The p-value gives the fraction of training data with an MD greater than or equal to the one for the given sample, assuming normally distributed data. The smaller the p-value, the less trustworthy the prediciton. For highly non-normal X properties (e.g., fingerprints), the MD p-value is wildly inaccurate.

Model Applicability

Unknown features are fingerprint features in the query molecule, but not found or appearing too infrequently in the training set.

1. All properties and OPS components are within expected ranges.
2. Unknown ECFP_6 feature: 2019062761: [*]:[c](:[*])O
3. Unknown ECFP_6 feature: -177786161: [*]:[cH]:[c](O):[cH]:[*]
4. Unknown ECFP_6 feature: -176483725: [*]=C[c](:[cH]:[*]):[cH]:[*]
5. Unknown ECFP_6 feature: -1831055759: [*]\C=C\[c](:[*]):[*]

Feature Contribution

Top features for positive contribution			
**Fingerprint**	**Bit/Smiles**	**Feature Structure**	**Score**
ECFP_6	-1925046727	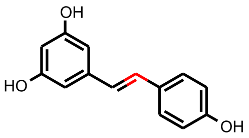 [*]C=[*]	0.0915

ECFP_6	642810091	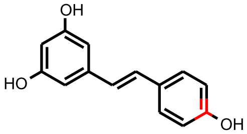<br>[*][c](:[*]):[*]	0.0424
FCFP_6	0	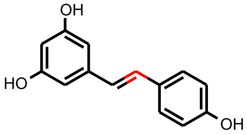<br>[*]C	0.0177
**Top Features for negative contribution**			
**Fingerprint**	**Bit/Smiles**	**Feature Structure**	**Score**
FCFP_6	-453677277	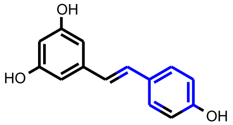<br>[*]C[c]1:[cH]:[cH]:[c]([*]):[*]:[c]:1[*]	-0.0906
FCFP_6	203677720	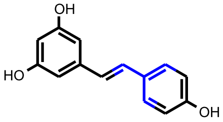<br>[*]C[c](:[cH]:[*]):[c]([*]):[*]	-0.0713
FCFP_6	7	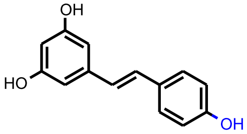<br>[*]O	-0.0664

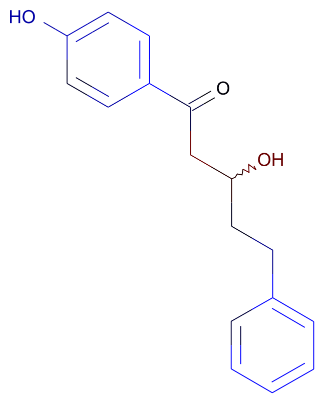

$C_{17}H_{18}O_3$
Molecular Weight: 270.323
ALogP: 3.293
Rotatable Bonds: 6
Acceptors: 3
Donors: 2

Model Prediction

Prediction: 0.173
Unit: g/kg_body_weight
Mahalanobis Distance: 23.7
Mahalanobis Distance p-value: 3.4e-013

Mahalanobis Distance: The Mahalanobis distance (MD) is a generalization of the Euclidean distance that accounts for correlations among the X properties. It is calculated as the distance to the center of the training data. The larger the MD, the less trustworthy the prediction.
Mahalanobis Distance p-value: The p-value gives the fraction of training data with an MD greater than or equal to the one for the given sample, assuming normally distributed data. The smaller the p-value, the less trustworthy the prediciton. For highly non-normal X properties (e.g., fingerprints), the MD p-value is wildly inaccurate.

Structural Similar Compounds

Name	ETODOLAC	DICHLOFENAC.NA	KETOPROFEN
Structure	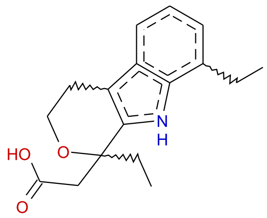	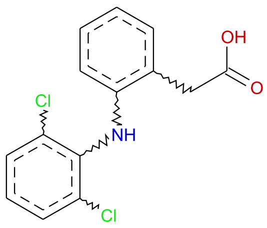	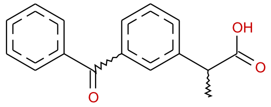
Actual Endpoint (-log C)	4.9813	5.47151	4.9282
Predicted Endpoint (-log C)	4.39289	3.9421	3.01758
Distance	0.504	0.523	0.526
Reference	NDA-18922	NDA-19201	NDA-18754

Model Applicability

Unknown features are fingerprint features in the query molecule, but not found or appearing too infrequently in the training set.

1. All properties and OPS components are within expected ranges.
2. Unknown ECFP_6 feature: 2023785560: [*]C([*])O
3. Unknown ECFP_6 feature: 196083830: [*]CC(O)C[*]
4. Unknown ECFP_6 feature: -1310859884: [*]C([*])CC(=[*])[*]
5. Unknown ECFP_6 feature: -2143661067: [*]CC(=O)[c](:[*]):[*]
6. Unknown ECFP_6 feature: -175146122: [*]C(=[*])[c](:[cH]:[*]):[cH]:[*]
7. Unknown ECFP_6 feature: -177786161: [*]:[cH]:[c](O):[cH]:[*]
8. Unknown ECFP_6 feature: 2019062761: [*]:[c](:[*])O
9. Unknown ECFP_6 feature: -1790802833: [*]CCC([*])[*]
10. Unknown ECFP_6 feature: -1795525632: [*]CC[c](:[*]):[*]
11. Unknown ECFP_6 feature: 1997021792: [*]:[cH]:[cH]:[cH]:[*]

Feature Contribution

Top features for positive contribution			
Fingerprint	Bit/Smiles	Feature Structure	Score

ECFP_6	1559650422	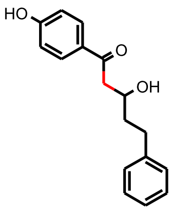 [*]C[*]	0.129
FCFP_6	3	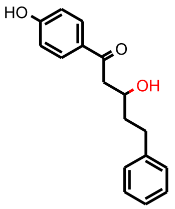 [*]N	0.0924
FCFP_6	-2093839777	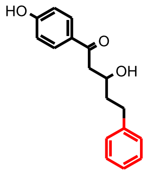 [*][c]1:[cH]:[cH]:[cH]:[cH]:[cH]:1	0.078
**Top Features for negative contribution**			
**Fingerprint**	**Bit/Smiles**	**Feature Structure**	**Score**
FCFP_6	991735244	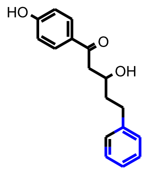 [*][c]1:[*]:[cH]:[cH] :[cH]:[cH]:1	-0.134
ECFP_6	1564392544	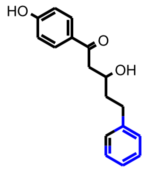 [*][c]1:[*]:[cH]:[cH] :[cH]:[cH]:1	-0.133

FCFP_6	1	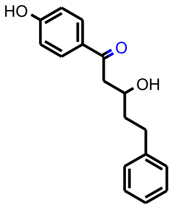<br>[*]O[*]	-0.102

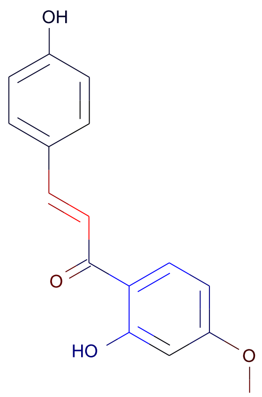

$C_{16}H_{14}O_4$
Molecular Weight: 270.28
ALogP: 3.201
Rotatable Bonds: 4
Acceptors: 4
Donors: 2

Structural Similar Compounds

Name	HC BLUE 1	ETODOLAC	3;3'-DIMETHOXYBENZIDINE .2HCL
Structure	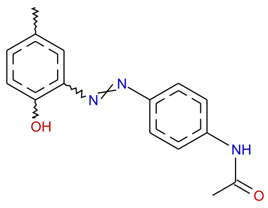	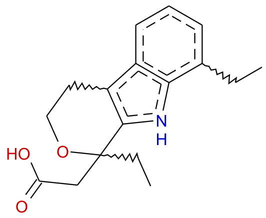	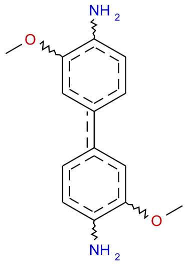
Actual Endpoint (-log C)	3.0323	4.9813	4.79463
Predicted Endpoint (-log C)	2.7171	4.39289	3.61371
Distance	0.439	0.506	0.510
Reference	NTP REPORT # 222	NDA-18922	NTP REPORT # 372

Model Prediction

Prediction: 0.0605
Unit: g/kg_body_weight
Mahalanobis Distance: 17.7
Mahalanobis Distance p-value: 0.00046

Mahalanobis Distance: The Mahalanobis distance (MD) is a generalization of the Euclidean distance that accounts for correlations among the X properties. It is calculated as the distance to the center of the training data. The larger the MD, the less trustworthy the prediction.
Mahalanobis Distance p-value: The p-value gives the fraction of training data with an MD greater than or equal to the one for the given sample, assuming normally distributed data. The smaller the p-value, the less trustworthy the prediciton. For highly non-normal X properties (e.g., fingerprints), the MD p-value is wildly inaccurate.

Model Applicability

Unknown features are fingerprint features in the query molecule, but not found or appearing too infrequently in the training set.

1. All properties and OPS components are within expected ranges.
2. Unknown ECFP_6 feature: 1307307440: [*]:[c](:[*])OC
3. Unknown ECFP_6 feature: 2019062761: [*]:[c](:[*])O
4. Unknown ECFP_6 feature: 1430764055: [*]=CC(=O)[c](:[*]):[*]
5. Unknown ECFP_6 feature: -470416293: [*]\C=C\C(=[*])[*]
6. Unknown ECFP_6 feature: -1831055759: [*]\C=C\[c](:[*]):[*]
7. Unknown ECFP_6 feature: -176483725: [*]=C[c](:[cH]:[*]):[cH]:[*]
8. Unknown ECFP_6 feature: -177786161: [*]:[cH]:[c](O):[cH]:[*]

Feature Contribution

Top features for positive contribution

Fingerprint	Bit/Smiles	Feature Structure	Score

FCFP_6	451847724	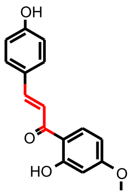<br>[*]\C=C\C(=[*])[*]	0.16
ECFP_6	-176455838	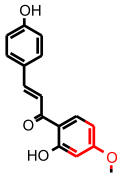<br>[*]O[c](:[cH]:[*]):[cH]:[*]	0.106
ECFP_6	-1925046727	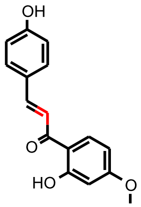<br>[*]C=[*]	0.0915
**Top Features for negative contribution**			
**Fingerprint**	**Bit/Smiles**	**Feature Structure**	**Score**
ECFP_6	1337040050	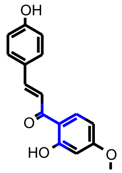<br>[*]C(=[*])[c](:[cH]:[*]):[c]([*]):[*]	-0.158
FCFP_6	1	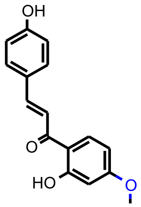<br>[*]O[*]	-0.102

FCFP_6	-453677277	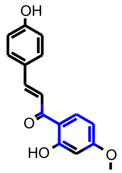<br>[*]C[c]1:[cH]:[cH]:[c<br>]([*]):[*]:[c]:1[*]	-0.0906

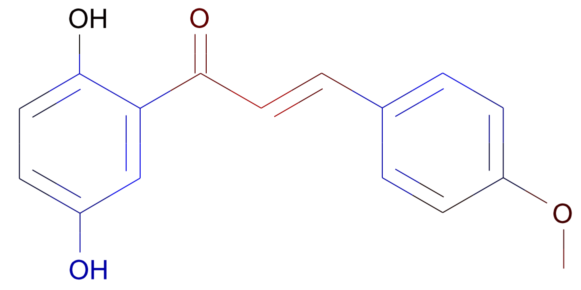

$C_{16}H_{14}O_4$
Molecular Weight: 270.28
ALogP: 3.201
Rotatable Bonds: 4
Acceptors: 4
Donors: 2

Model Prediction

Prediction: 0.0605
Unit: g/kg_body_weight
Mahalanobis Distance: 17.7
Mahalanobis Distance p-value: 0.00046

Mahalanobis Distance: The Mahalanobis distance (MD) is a generalization of the Euclidean distance that accounts for correlations among the X properties. It is calculated as the distance to the center of the training data. The larger the MD, the less trustworthy the prediction.
Mahalanobis Distance p-value: The p-value gives the fraction of training data with an MD greater than or equal to the one for the given sample, assuming normally distributed data. The smaller the p-value, the less trustworthy the prediciton. For highly non-normal X properties (e.g., fingerprints), the MD p-value is wildly inaccurate.

Structural Similar Compounds

Name	HC BLUE 1	ETODOLAC	3;3'-DIMETHOXYBENZIDINE .2HCL
Structure	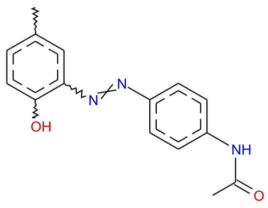	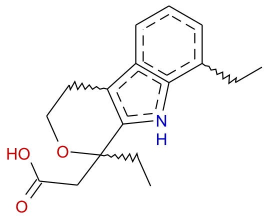	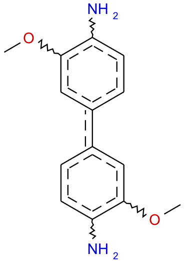
Actual Endpoint (-log C)	3.0323	4.9813	4.79463
Predicted Endpoint (-log C)	2.7171	4.39289	3.61371
Distance	0.437	0.506	0.510
Reference	NTP REPORT # 222	NDA-18922	NTP REPORT # 372

Model Applicability

Unknown features are fingerprint features in the query molecule, but not found or appearing too infrequently in the training set.

1. All properties and OPS components are within expected ranges.
2. Unknown ECFP_6 feature: 1307307440: [*]:[c](:[*])OC
3. Unknown ECFP_6 feature: -176483725: [*]=C[c](:[cH]:[*]):[cH]:[*]
4. Unknown ECFP_6 feature: -1831055759: [*]\C=C\[c](:[*]):[*]
5. Unknown ECFP_6 feature: -470416293: [*]\C=C\C(=[*])[*]
6. Unknown ECFP_6 feature: 1430764055: [*]=CC(=O)[c](:[*]):[*]
7. Unknown ECFP_6 feature: -177786161: [*]:[cH]:[c](O):[cH]:[*]
8. Unknown ECFP_6 feature: 2019062761: [*]:[c](:[*])O

Feature Contribution

Top features for positive contribution			
**Fingerprint**	**Bit/Smiles**	**Feature Structure**	**Score**

FCFP_6	451847724	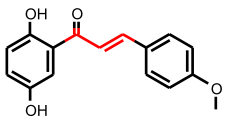 [*]\C=C\C(=[*])[*]	0.16
ECFP_6	-176455838	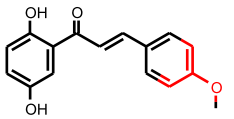 [*]O[c](:[cH]:[*]):[cH]:[*]	0.106
ECFP_6	-1925046727	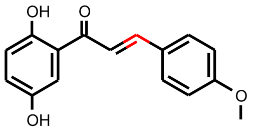 [*]C=[*]	0.0915

Top Features for negative contribution

Fingerprint	Bit/Smiles	Feature Structure	Score
ECFP_6	1337040050	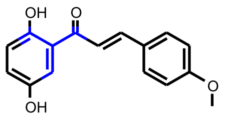 [*]C(=[*])[c](:[cH]:[*]):[c]([*]):[*]	-0.158
FCFP_6	1	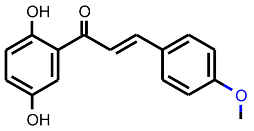 [*]O[*]	-0.102

FCFP_6	-453677277	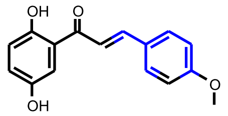<br>[*]C[c]1:[cH]:[cH]:[c<br>]([*]):[*]:[c]:1[*]	-0.0906

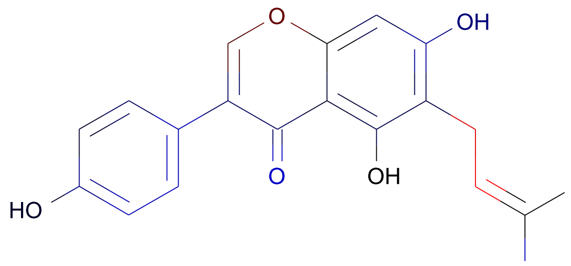

$C_{20}H_{18}O_5$
Molecular Weight: 338.354
ALogP: 3.997
Rotatable Bonds: 3
Acceptors: 5
Donors: 3

Model Prediction

Prediction: 0.053
Unit: g/kg_body_weight
Mahalanobis Distance: 25.3
Mahalanobis Distance p-value: 4.07e-016

Mahalanobis Distance: The Mahalanobis distance (MD) is a generalization of the Euclidean distance that accounts for correlations among the X properties. It is calculated as the distance to the center of the training data. The larger the MD, the less trustworthy the prediction.
Mahalanobis Distance p-value: The p-value gives the fraction of training data with an MD greater than or equal to the one for the given sample, assuming normally distributed data. The smaller the p-value, the less trustworthy the prediciton. For highly non-normal X properties (e.g., fingerprints), the MD p-value is wildly inaccurate.

Structural Similar Compounds

Name	ZERANOL	HC BLUE 1	ZEARALENONE
Structure	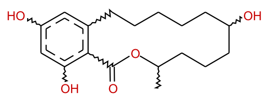	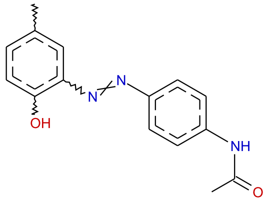	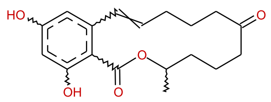
Actual Endpoint (-log C)	4.20737	3.0323	5.40602
Predicted Endpoint (-log C)	3.4948	2.7171	3.57081
Distance	0.601	0.618	0.635
Reference	REGULAT. TOXICOL. PHARMACOL. 1983; 3: 9-	NTP REPORT # 222	NTP REPORT # 235

Model Applicability

Unknown features are fingerprint features in the query molecule, but not found or appearing too infrequently in the training set.

1. OPS PC30 out of range. Value: 5.0451. Training min, max, SD, explained variance: -3.8594, 4.2863, 1.27, 0.0071.
2. Unknown ECFP_6 feature: -1774681326: [*]C=C(C)C
3. Unknown ECFP_6 feature: -98561723: [*]CC=C([*])[*]
4. Unknown ECFP_6 feature: 770519970: [*]=CC[c](:[*]):[*]
5. Unknown ECFP_6 feature: -512323383: [*]C[c](:[c]([*]):[*]):[c]([*]):[*]
6. Unknown ECFP_6 feature: 2019062761: [*]:[c](:[*])O
7. Unknown ECFP_6 feature: -570915357: [*]O[c](:[cH]:[*]):[c]([*]):[*]
8. Unknown ECFP_6 feature: -813997308: [*]C(=[*])[c](:[c]([*]):[*]):[c]([*]):[*]
9. Unknown ECFP_6 feature: -1660913849: [*][c](:[*]):[c](O):[c]([*]):[*]
10. Unknown ECFP_6 feature: 1717462980: [*]C(=[*])C(=O)[c](:[*]):[*]
11. Unknown ECFP_6 feature: 1717082529: [*]\C=C(/C(=[*])[*])\[c](:[*]):[*]
12. Unknown ECFP_6 feature: 471124258: [*]OC=C([*])[*]
13. Unknown ECFP_6 feature: -554736825: [*]=CO[c](:[*]):[*]
14. Unknown ECFP_6 feature: -181568884: [*]C(=[*])[c](:[cH]:[*]):[cH]:[*]
15. Unknown ECFP_6 feature: -177786161: [*]:[cH]:[c](O):[cH]:[*]

Feature Contribution

Top features for positive contribution

Fingerprint	Bit/Smiles	Feature Structure	Score
FCFP_6	451847724	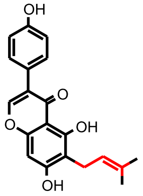 [*]\C=C\C(=[*])[*]	0.16
ECFP_6	1559650422	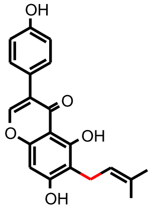 [*]C[*]	0.129
ECFP_6	-1925046727	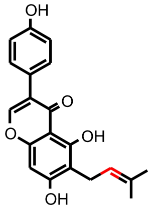 [*]C=[*]	0.0915

Top Features for negative contribution

Fingerprint	Bit/Smiles	Feature Structure	Score
ECFP_6	2106656448	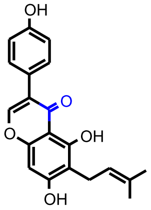 [*]C(=O)[*]	-0.11

FCFP_6	1	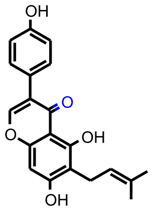<br>[*]O[*]	-0.102
FCFP_6	-453677277	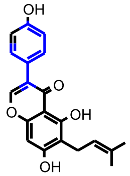<br>[*]C[c]1:[cH]:[cH]:[c<br>]([*]):[*]:[c]:1[*]	-0.0906

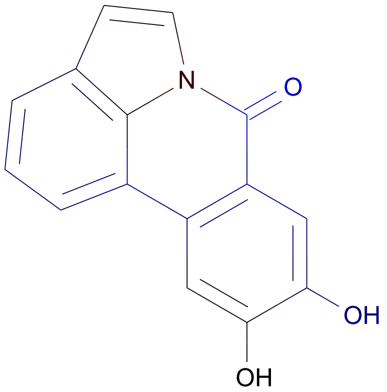

$C_{15}H_9NO_3$
Molecular Weight: 251.237
ALogP: 2.982
Rotatable Bonds: 0
Acceptors: 3
Donors: 2

Model Prediction

Prediction: 0.103
Unit: g/kg_body_weight
Mahalanobis Distance: 25.2
Mahalanobis Distance p-value: 5.41e-016

Mahalanobis Distance: The Mahalanobis distance (MD) is a generalization of the Euclidean distance that accounts for correlations among the X properties. It is calculated as the distance to the center of the training data. The larger the MD, the less trustworthy the prediction.
Mahalanobis Distance p-value: The p-value gives the fraction of training data with an MD greater than or equal to the one for the given sample, assuming normally distributed data. The smaller the p-value, the less trustworthy the prediciton. For highly non-normal X properties (e.g., fingerprints), the MD p-value is wildly inaccurate.

Structural Similar Compounds

Name	PROFLAVINE	LORAZEPAM	1-AMINO-2-METHYLANTHRAQUINONE
Structure	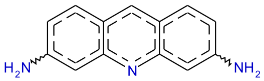	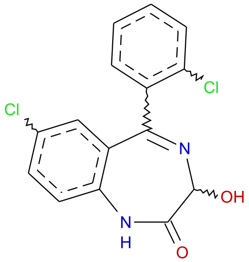	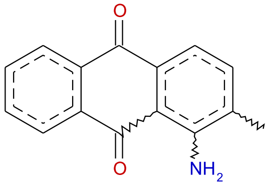
Actual Endpoint (-log C)	4.14458	3.7286	3.6763
Predicted Endpoint (-log C)	4.05203	2.8638	3.47653
Distance	0.532	0.600	0.614
Reference	NTP 5 58	NDA-17794	NTP 111 A-4

Model Applicability

Unknown features are fingerprint features in the query molecule, but not found or appearing too infrequently in the training set.

1. All properties and OPS components are within expected ranges.
2. Unknown FCFP_2 feature: -1549639687: [*]:n(:[*])C(=O)[c](:[*]):[*]
3. Unknown ECFP_6 feature: 672362763: [*]n(:[*]):[*]
4. Unknown ECFP_6 feature: 2019062761: [*]:[c](:[*])O
5. Unknown ECFP_6 feature: 1997021792: [*]:[cH]:[cH]:[cH]:[*]
6. Unknown ECFP_6 feature: -178525456: [*]:[cH]:[c]1:[cH]:[*]:[*]:[c]:1:[*]
7. Unknown ECFP_6 feature: -1660898726: [*]n1:[*]:[*]:[c](:[*]):[c]:1:[c]([*]):[*]
8. Unknown ECFP_6 feature: 1312166648: [*]C(=[*])n1:[cH]:[*]:[*]:[c]:1:[*]
9. Unknown ECFP_6 feature: 1444648700: [*]:n(:[*])C(=O)[c](:[*]):[*]
10. Unknown ECFP_6 feature: 1997522062: [*]n1:[*]:[*]:[cH]:[cH]:1

Feature Contribution

Top features for positive contribution			
Fingerprint	Bit/Smiles	Feature Structure	Score

ECFP_6	2007300961	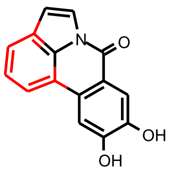<br>[*][c]1:[*]:[c](:[*]) :[cH]:[cH]:[cH]:1	0.0564
ECFP_6	642810091	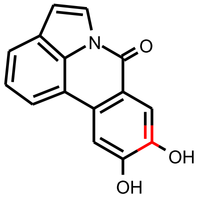<br>[*][c](:[*]):[*]	0.0424
FCFP_6	17	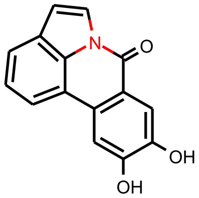<br>[*]:n:[*]	0.0417
**Top Features for negative contribution**			
**Fingerprint**	**Bit/Smiles**	**Feature Structure**	**Score**
FCFP_6	991735244	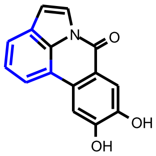<br>[*][c]1:[*]:[cH]:[cH] :[cH]:[cH]:1	-0.134
ECFP_6	2106656448	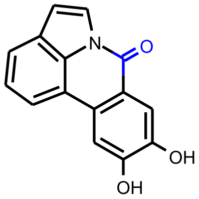<br>[*]C(=O)[*]	-0.11

FCFP_6	1	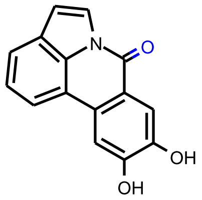<br>[*]O[*]	-0.102

remdesivir

TOPKAT_Chronic_LOAEL

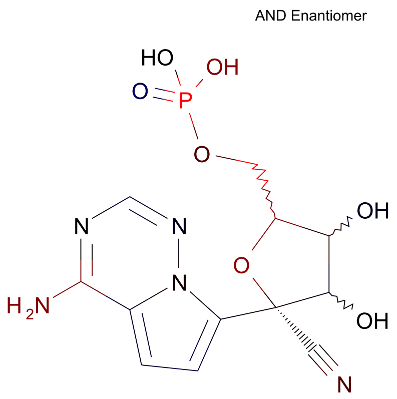

$C_{12}H_{14}N_5O_7P$
Molecular Weight: 371.24262
ALogP: -1.502
Rotatable Bonds: 4
Acceptors: 11
Donors: 5

Structural Similar Compounds

Name	TETRACYCLINE .HCL	4;4'-DIAMINO-2;2'-STILBENEDIS	OXYTETRACYCLINE .HCL
Structure	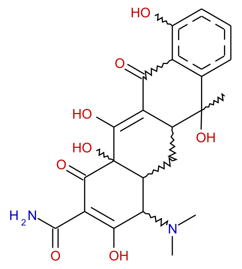	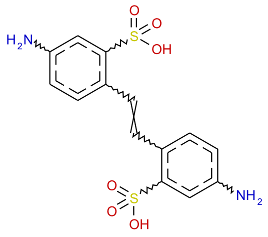	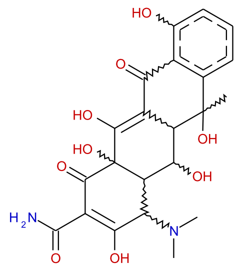
Actual Endpoint (-log C)	2.85193	2.47175	2.56626
Predicted Endpoint (-log C)	3.94748	3.53715	3.75581
Distance	0.746	0.746	0.802
Reference	NTP REPORT # 344	NTP 412 82	NTP REPORT # 315

Model Prediction

Prediction: 0.00379
Unit: g/kg_body_weight
Mahalanobis Distance: 47.7
Mahalanobis Distance p-value: 2.93e-054

Mahalanobis Distance: The Mahalanobis distance (MD) is a generalization of the Euclidean distance that accounts for correlations among the X properties. It is calculated as the distance to the center of the training data. The larger the MD, the less trustworthy the prediction.
Mahalanobis Distance p-value: The p-value gives the fraction of training data with an MD greater than or equal to the one for the given sample, assuming normally distributed data. The smaller the p-value, the less trustworthy the prediciton. For highly non-normal X properties (e.g., fingerprints), the MD p-value is wildly inaccurate.

Model Applicability

Unknown features are fingerprint features in the query molecule, but not found or appearing too infrequently in the training set.

1. All properties and OPS components are within expected ranges.
2. Unknown FCFP_2 feature: 472180098: [*]OP(=O)(O)O
3. Unknown FCFP_2 feature: -332197802: [*][c]1:[*]:[*]:[c](:[*]):n:1:n:[*]
4. Unknown ECFP_6 feature: -1114776580: [*]C#[*]
5. Unknown ECFP_6 feature: -1101847286: [*]#N
6. Unknown ECFP_6 feature: 672362763: [*]n(:[*]):[*]
7. Unknown ECFP_6 feature: 1126642748: [*]OP(=O)(O)O
8. Unknown ECFP_6 feature: 2100964382: [*]P(=O)([*])[*]
9. Unknown ECFP_6 feature: 2024329577: [*]P(=[*])([*])O
10. Unknown ECFP_6 feature: -1250439909: [*]COP(=[*])([*])[*]
11. Unknown ECFP_6 feature: -1687549011: [*]OCC([*])[*]
12. Unknown ECFP_6 feature: -194719409: [*]C1[*][*]C([*])([*])O1
13. Unknown ECFP_6 feature: -553149446: [*]CC1O[*][*]C1[*]
14. Unknown ECFP_6 feature: 305695353: [*]C1[*][*]C([*])C1O
15. Unknown ECFP_6 feature: -521596699: [*]C1[*][*]C([*])([*])C1O
16. Unknown ECFP_6 feature: 1258791451: [*]C1[*][*]O[C@]1(C#[*])[c](:[*]):[*]
17. Unknown ECFP_6 feature: 2024749573: [*]C([*])O
18. Unknown ECFP_6 feature: -264833661: [*]C([*])([*])C#N
19. Unknown ECFP_6 feature: 1412053881: [*]C#N

20. Unknown ECFP_6 feature: -1507082173: [*][c]1:[*]:[*]:[c](:[*]):n:1:n:[*]
21. Unknown ECFP_6 feature: -676555381: [*]:[cH]:n:n(:[*]):[*]
22. Unknown ECFP_6 feature: -710237522: [*]:n:[cH]:n:[*]
23. Unknown ECFP_6 feature: -677309799: [*][c](:[*]):n:[cH]:[*]
24. Unknown ECFP_6 feature: -1734834311: [*]:n:[c](N):[c](:[*]):[*]
25. Unknown ECFP_6 feature: 1334415134: [*][c](:[*]):[c]1:[cH]:[*]:[*]:n:1:[*]
26. Unknown ECFP_6 feature: -66263742: [*]C([*])([*])[c]1:[cH]:[*]:[*]:n:1:[*]
27. Unknown ECFP_6 feature: -938530932: [*]:[c](:[*])N

Feature Contribution

Top features for positive contribution			
**Fingerprint**	**Bit/Smiles**	**Feature Structure**	**Score**
ECFP_6	-167460056	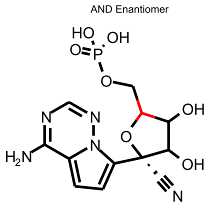 [*]C([*])[*]	0.136
FCFP_6	-1143715940	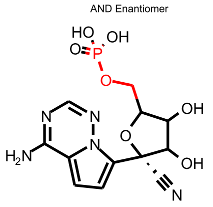 [*]COP(=[*])([*])[*]	0.13
ECFP_6	1559650422	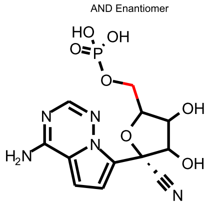 [*]C[*]	0.129

Top Features for negative contribution			
**Fingerprint**	**Bit/Smiles**	**Feature Structure**	**Score**

FCFP_6	1	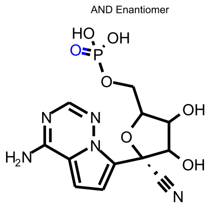 [*]O[*]	-0.102
ECFP_6	1996767644	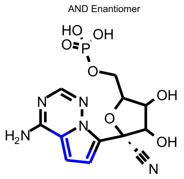 [*][c](:[*]):[cH]:[cH]:[*]	-0.0497
FCFP_6	16	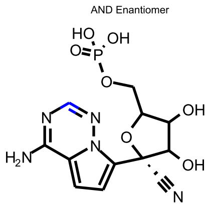 [*][c](:[*]):[*]	-0.0462

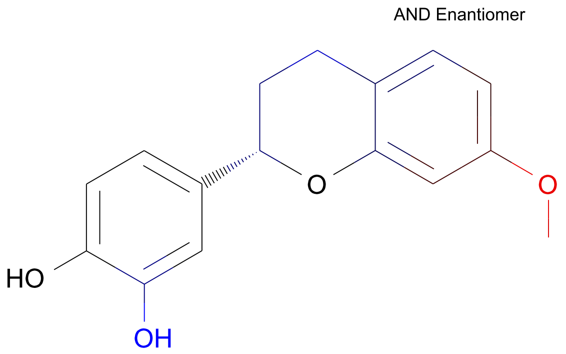

$C_{16}H_{16}O_4$

Molecular Weight: 272.296

ALogP: 3.456

Rotatable Bonds: 2

Acceptors: 4

Donors: 2

Structural Similar Compounds

Name	DISPERSE YELLOW 3	BISPHENOL	BENZIDINE,3,3'-DIMETHOXY-
Structure	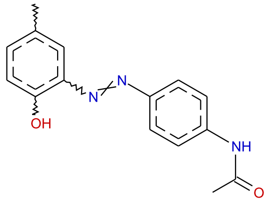	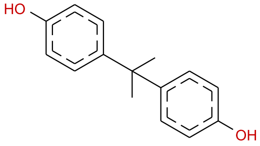	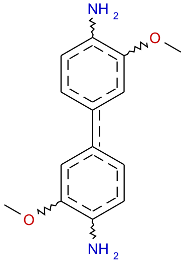
Actual Endpoint (-log C)	2.77703	3.40425	4.06569
Predicted Endpoint (-log C)	2.80195	2.7079	3.57405
Distance	0.389	0.481	0.500
Reference	NCI/NTP TR-222	NCI/NTP TR-215	NCI/NTP Report 10, Nov. 1987

Model Prediction

Prediction: 0.285

Unit: g/kg_body_weight

Mahalanobis Distance: 6.52

Mahalanobis Distance p-value: 0.387

Mahalanobis Distance: The Mahalanobis distance (MD) is a generalization of the Euclidean distance that accounts for correlations among the X properties. It is calculated as the distance to the center of the training data. The larger the MD, the less trustworthy the prediction.
Mahalanobis Distance p-value: The p-value gives the fraction of training data with an MD greater than or equal to the one for the given sample, assuming normally distributed data. The smaller the p-value, the less trustworthy the prediciton. For highly non-normal X properties (e.g., fingerprints), the MD p-value is wildly inaccurate.

Model Applicability

Unknown features are fingerprint features in the query molecule, but not found or appearing too infrequently in the training set.

1. All properties and OPS components are within expected ranges.
2. Unknown FCFP_2 feature: 1186333723: [*]C[C@H](O[*])[c](:[*]):[*]

Feature Contribution

Top features for positive contribution			
Fingerprint	Bit/Smiles	Feature Structure	Score
FCFP_2	136627117	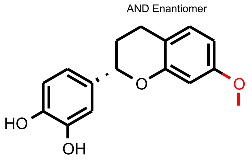 [*]OC	0.173

FCFP_2	1036089772	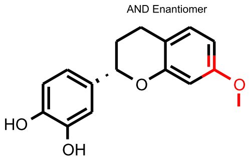<br>[*]:[c](:[*])OC	0.0749
FCFP_2	332760439	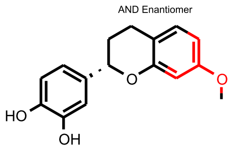<br>[*]O[c](:[cH]:[*]):[c<br>H]:[*]	0.0611
**Top Features for negative contribution**			
**Fingerprint**	**Bit/Smiles**	**Feature Structure**	**Score**
FCFP_2	7	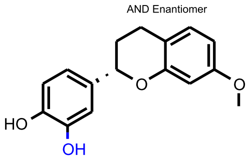<br>[*]O	-0.214
FCFP_2	-549108873	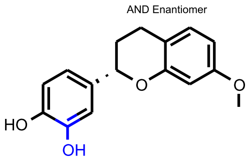<br>[*]:[c](:[*])O	-0.127
FCFP_2	-1272798659	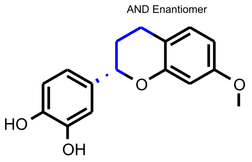<br>[*]CCC([*])[*]	-0.111

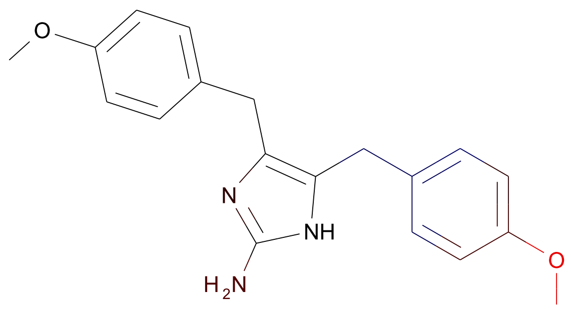

$C_{19}H_{21}N_3O_2$
Molecular Weight: 323.389
ALogP: 3.305
Rotatable Bonds: 6
Acceptors: 4
Donors: 2

Model Prediction

Prediction: 0.0863
Unit: g/kg_body_weight
Mahalanobis Distance: 8.81
Mahalanobis Distance p-value: 0.00255

Mahalanobis Distance: The Mahalanobis distance (MD) is a generalization of the Euclidean distance that accounts for correlations among the X properties. It is calculated as the distance to the center of the training data. The larger the MD, the less trustworthy the prediction.
Mahalanobis Distance p-value: The p-value gives the fraction of training data with an MD greater than or equal to the one for the given sample, assuming normally distributed data. The smaller the p-value, the less trustworthy the prediciton. For highly non-normal X properties (e.g., fingerprints), the MD p-value is wildly inaccurate.

Structural Similar Compounds

Name	BENZIDINE,3,3'-DIMETHOXY-	PHENOLPHTHALEIN	DISPERSE YELLOW 3
Structure	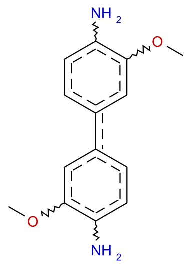	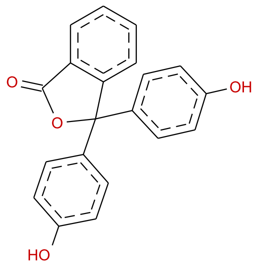	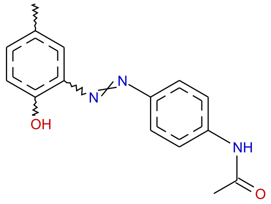
Actual Endpoint (-log C)	4.06569	2.20184	2.77703
Predicted Endpoint (-log C)	3.57405	2.8857	2.80195
Distance	0.605	0.632	0.658
Reference	NCI/NTP Report 10, Nov. 1987	NCI/NTP TR-465	NCI/NTP TR-222

Model Applicability

Unknown features are fingerprint features in the query molecule, but not found or appearing too infrequently in the training set.

1. All properties and OPS components are within expected ranges.
2. Unknown FCFP_2 feature: -1151854667: N[c]1:[nH]:[*]:[*]:n:1

Feature Contribution

Top features for positive contribution			
**Fingerprint**	**Bit/Smiles**	**Feature Structure**	**Score**
FCFP_2	136627117	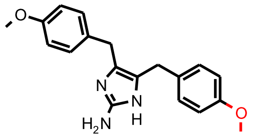 [*]OC	0.173

FCFP_2	1036089772	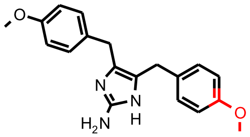 [*]:[c](:[*])OC	0.0749
FCFP_2	3	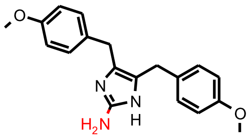 [*]N	0.0737
**Top Features for negative contribution**			
**Fingerprint**	**Bit/Smiles**	**Feature Structure**	**Score**
FCFP_2	203677720	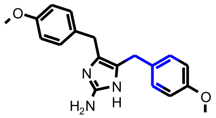 [*]C[c](:[cH]:[*]):[c]([*]):[*]	-0.0829
FCFP_2	1	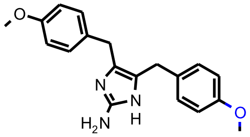 [*]O[*]	-0.0796
FCFP_2	16	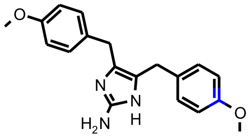 [*][c](:[*]):[*]	-0.0512

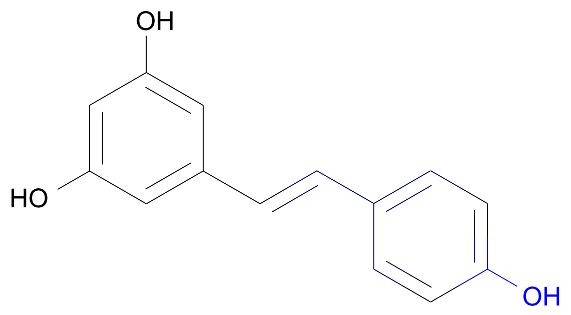

$C_{14}H_{12}O_3$
Molecular Weight: 228.243
ALogP: 3.09
Rotatable Bonds: 2
Acceptors: 3
Donors: 3

Model Prediction

Prediction: 0.69
Unit: g/kg_body_weight
Mahalanobis Distance: 4.94
Mahalanobis Distance p-value: 0.965

Mahalanobis Distance: The Mahalanobis distance (MD) is a generalization of the Euclidean distance that accounts for correlations among the X properties. It is calculated as the distance to the center of the training data. The larger the MD, the less trustworthy the prediction.
Mahalanobis Distance p-value: The p-value gives the fraction of training data with an MD greater than or equal to the one for the given sample, assuming normally distributed data. The smaller the p-value, the less trustworthy the prediciton. For highly non-normal X properties (e.g., fingerprints), the MD p-value is wildly inaccurate.

Structural Similar Compounds

Name	BISPHENOL	t-BUTYLHYDROQUINONE	DISPERSE YELLOW 3
Structure	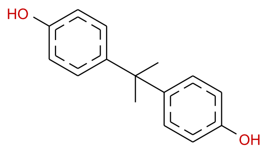	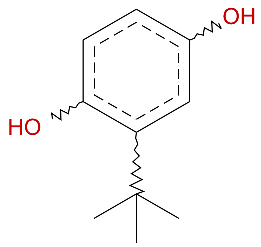	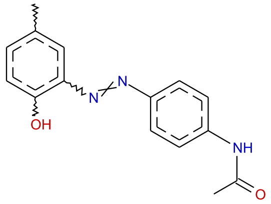
Actual Endpoint (-log C)	3.40425	2.44253	2.77703
Predicted Endpoint (-log C)	2.7079	2.62758	2.80195
Distance	0.370	0.471	0.509
Reference	NCI/NTP TR-215	NCI/NTP TR-459	NCI/NTP TR-222

Model Applicability

Unknown features are fingerprint features in the query molecule, but not found or appearing too infrequently in the training set.

1. All properties and OPS components are within expected ranges.

Feature Contribution

Top Features for negative contribution

Fingerprint	Bit/Smiles	Feature Structure	Score
FCFP_2	7	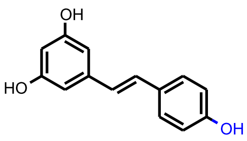 [*]O	-0.214

FCFP_2	-549108873	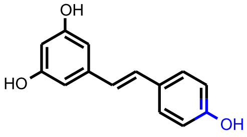 [*]:[c](:[*])O	-0.127
FCFP_2	203677720	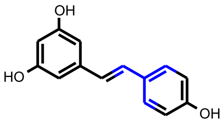 [*]C[c](:[cH]:[*]):[c]([*]):[*]	-0.0829

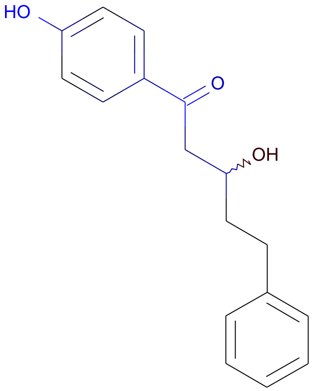

$C_{17}H_{18}O_3$
Molecular Weight: 270.323
ALogP: 3.293
Rotatable Bonds: 6
Acceptors: 3
Donors: 2

Model Prediction

Prediction: 0.829
Unit: g/kg_body_weight
Mahalanobis Distance: 7.28
Mahalanobis Distance p-value: 0.119

Mahalanobis Distance: The Mahalanobis distance (MD) is a generalization of the Euclidean distance that accounts for correlations among the X properties. It is calculated as the distance to the center of the training data. The larger the MD, the less trustworthy the prediction.
Mahalanobis Distance p-value: The p-value gives the fraction of training data with an MD greater than or equal to the one for the given sample, assuming normally distributed data. The smaller the p-value, the less trustworthy the prediciton. For highly non-normal X properties (e.g., fingerprints), the MD p-value is wildly inaccurate.

Structural Similar Compounds

Name	DISPERSE YELLOW 3	CINNAMYL ANTHRANILATE	BENZOIN
Structure	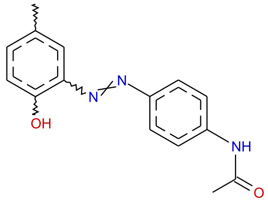	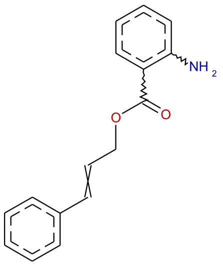	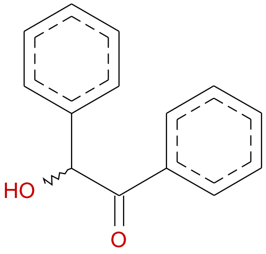
Actual Endpoint (-log C)	2.77703	2.2733	4.27569
Predicted Endpoint (-log C)	2.80195	3.25473	3.12678
Distance	0.472	0.507	0.511
Reference	NCI/NTP TR-222	NCI/NTP TR-196	NCI/NTP TR-204

Model Applicability

Unknown features are fingerprint features in the query molecule, but not found or appearing too infrequently in the training set.

1. All properties and OPS components are within expected ranges.

Feature Contribution

Top features for positive contribution

Fingerprint	Bit/Smiles	Feature Structure	Score
FCFP_2	3	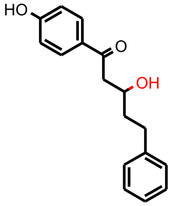 [*]N	0.0737

Top Features for negative contribution

Fingerprint	Bit/Smiles	Feature Structure	Score

FCFP_2	7	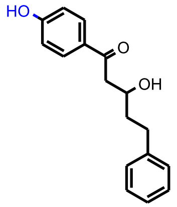<br>[*]O	-0.214
FCFP_2	-549108873	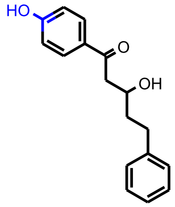<br>[*]:[c](:[*])O	-0.127
FCFP_2	-1272798659	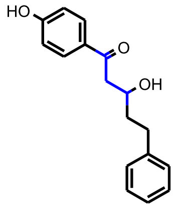<br>[*]CCC([*])[*]	-0.111

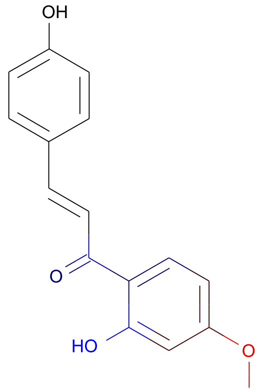

$C_{16}H_{14}O_4$

Molecular Weight: 270.28

ALogP: 3.201

Rotatable Bonds: 4

Acceptors: 4

Donors: 2

Model Prediction

Prediction: 0.32

Unit: g/kg_body_weight

Mahalanobis Distance: 5.5

Mahalanobis Distance p-value: 0.848

Mahalanobis Distance: The Mahalanobis distance (MD) is a generalization of the Euclidean distance that accounts for correlations among the X properties. It is calculated as the distance to the center of the training data. The larger the MD, the less trustworthy the prediction.
Mahalanobis Distance p-value: The p-value gives the fraction of training data with an MD greater than or equal to the one for the given sample, assuming normally distributed data. The smaller the p-value, the less trustworthy the prediciton. For highly non-normal X properties (e.g., fingerprints), the MD p-value is wildly inaccurate.

Structural Similar Compounds

Name	DISPERSE YELLOW 3	BENZIDINE,3,3'-DIMETHOXY-	CINNAMYL ANTHRANILATE
Structure	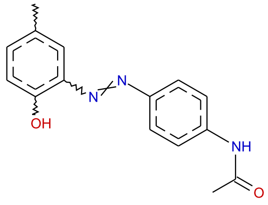	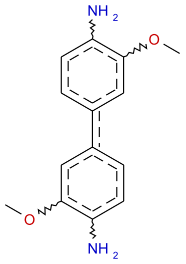	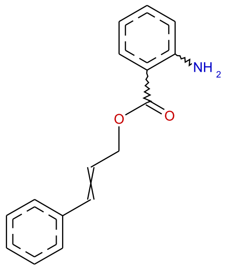
Actual Endpoint (-log C)	2.77703	4.06569	2.2733
Predicted Endpoint (-log C)	2.80195	3.57405	3.25473
Distance	0.348	0.463	0.506
Reference	NCI/NTP TR-222	NCI/NTP Report 10, Nov. 1987	NCI/NTP TR-196

Model Applicability

Unknown features are fingerprint features in the query molecule, but not found or appearing too infrequently in the training set.

1. All properties and OPS components are within expected ranges.

Feature Contribution

Top features for positive contribution

Fingerprint	Bit/Smiles	Feature Structure	Score
FCFP_2	136627117	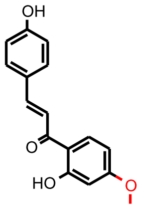 [*]OC	0.173

FCFP_2	1036089772	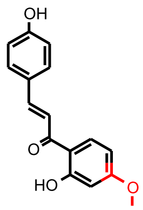 [*]:[c](:[*])OC	0.0749
FCFP_2	332760439	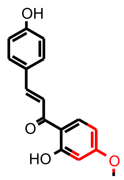 [*]O[c](:[cH]:[*]):[cH]:[*]	0.0611

Top Features for negative contribution

Fingerprint	Bit/Smiles	Feature Structure	Score
FCFP_2	7	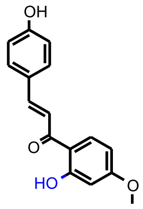 [*]O	-0.214
FCFP_2	-549108873	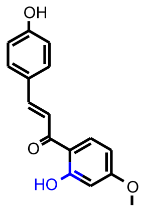 [*]:[c](:[*])O	-0.127
FCFP_2	1872154524	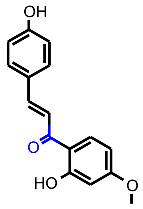 [*]C(=O)[*]	-0.105

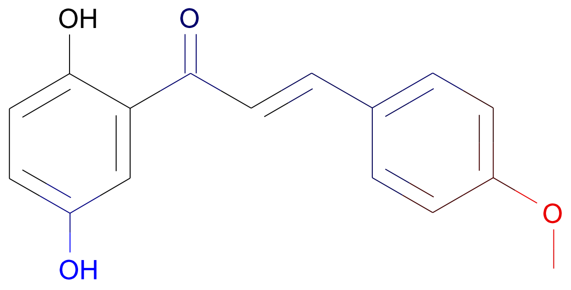

$C_{16}H_{14}O_4$
Molecular Weight: 270.28
ALogP: 3.201
Rotatable Bonds: 4
Acceptors: 4
Donors: 2

Model Prediction

Prediction: 0.32
Unit: g/kg_body_weight
Mahalanobis Distance: 5.5
Mahalanobis Distance p-value: 0.848

Mahalanobis Distance: The Mahalanobis distance (MD) is a generalization of the Euclidean distance that accounts for correlations among the X properties. It is calculated as the distance to the center of the training data. The larger the MD, the less trustworthy the prediction.
Mahalanobis Distance p-value: The p-value gives the fraction of training data with an MD greater than or equal to the one for the given sample, assuming normally distributed data. The smaller the p-value, the less trustworthy the prediciton. For highly non-normal X properties (e.g., fingerprints), the MD p-value is wildly inaccurate.

Structural Similar Compounds

Name	DISPERSE YELLOW 3	BENZIDINE,3,3'-DIMETHOXY-	CINNAMYL ANTHRANILATE
Structure	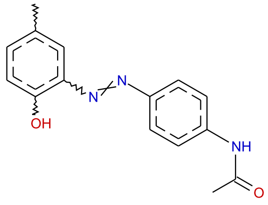	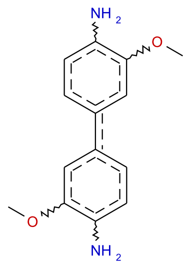	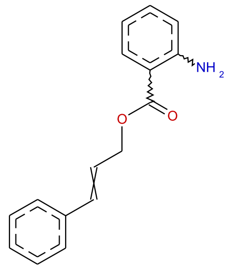
Actual Endpoint (-log C)	2.77703	4.06569	2.2733
Predicted Endpoint (-log C)	2.80195	3.57405	3.25473
Distance	0.348	0.463	0.506
Reference	NCI/NTP TR-222	NCI/NTP Report 10, Nov. 1987	NCI/NTP TR-196

Model Applicability

Unknown features are fingerprint features in the query molecule, but not found or appearing too infrequently in the training set.

1. All properties and OPS components are within expected ranges.

Feature Contribution

Top features for positive contribution			
Fingerprint	Bit/Smiles	Feature Structure	Score
FCFP_2	136627117	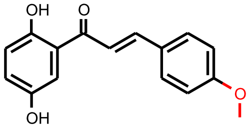 [*]OC	0.173

FCFP_2	1036089772	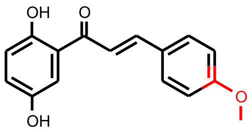 [*]:[c](:[*])OC	0.0749
FCFP_2	332760439	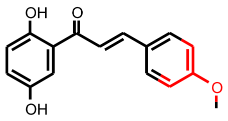 [*]O[c](:[cH]:[*]):[c H]:[*]	0.0611
**Top Features for negative contribution**			
**Fingerprint**	**Bit/Smiles**	**Feature Structure**	**Score**
FCFP_2	7	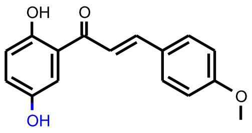 [*]O	-0.214
FCFP_2	-549108873	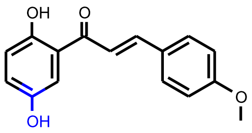 [*]:[c](:[*])O	-0.127
FCFP_2	1872154524	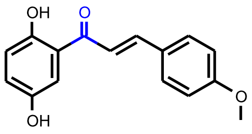 [*]C(=O)[*]	-0.105

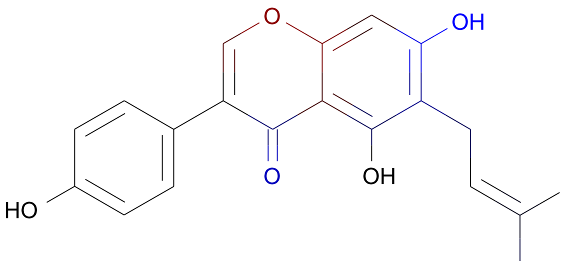

$C_{20}H_{18}O_5$
Molecular Weight: 338.354
ALogP: 3.997
Rotatable Bonds: 3
Acceptors: 5
Donors: 3

Model Prediction

Prediction: 0.525
Unit: g/kg_body_weight
Mahalanobis Distance: 6.34
Mahalanobis Distance p-value: 0.474

Mahalanobis Distance: The Mahalanobis distance (MD) is a generalization of the Euclidean distance that accounts for correlations among the X properties. It is calculated as the distance to the center of the training data. The larger the MD, the less trustworthy the prediction.
Mahalanobis Distance p-value: The p-value gives the fraction of training data with an MD greater than or equal to the one for the given sample, assuming normally distributed data. The smaller the p-value, the less trustworthy the prediciton. For highly non-normal X properties (e.g., fingerprints), the MD p-value is wildly inaccurate.

Structural Similar Compounds

Name	DISPERSE YELLOW 3	PHENOLPHTHALEIN	C.I.BASIC RED 9
Structure	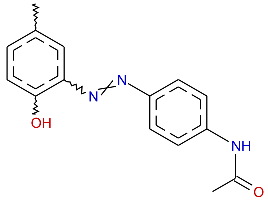	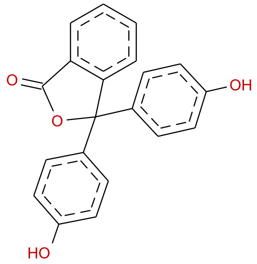	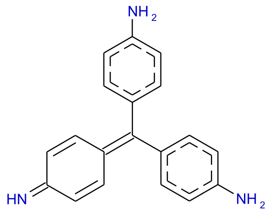
Actual Endpoint (-log C)	2.77703	2.20184	3.4456
Predicted Endpoint (-log C)	2.80195	2.8857	3.33206
Distance	0.515	0.590	0.602
Reference	NCI/NTP TR-222	NCI/NTP TR-465	NCI/NTP TR-285

Model Applicability

Unknown features are fingerprint features in the query molecule, but not found or appearing too infrequently in the training set.

1. All properties and OPS components are within expected ranges.

Feature Contribution

Top features for positive contribution			
**Fingerprint**	**Bit/Smiles**	**Feature Structure**	**Score**
FCFP_2	1036089772	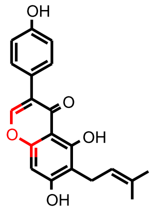 [*]:[c](:[*])OC	0.0749

FCFP_2	332760439	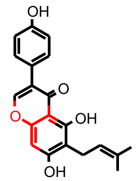<br>[*]O[c](:[cH]:[*]):[cH]:[*]	0.0611

Top Features for negative contribution

**Fingerprint**	**Bit/Smiles**	**Feature Structure**	**Score**
FCFP_2	7	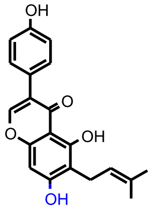<br>[*]O	-0.214
FCFP_2	-549108873	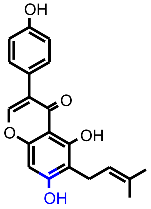<br>[*]:[c](:[*])O	-0.127
FCFP_2	1872154524	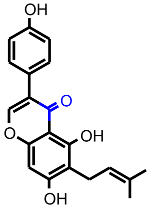<br>[*]C(=O)[*]	-0.105

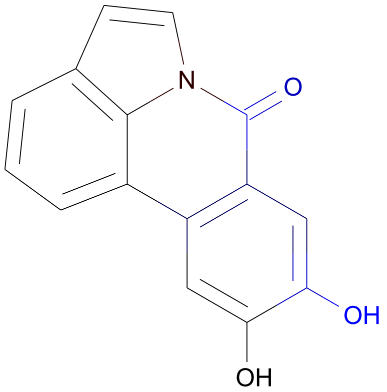

$C_{15}H_9NO_3$
Molecular Weight: 251.237
ALogP: 2.982
Rotatable Bonds: 0
Acceptors: 3
Donors: 2

Model Prediction

Prediction: 0.453
Unit: g/kg_body_weight
Mahalanobis Distance: 6.41
Mahalanobis Distance p-value: 0.438

Mahalanobis Distance: The Mahalanobis distance (MD) is a generalization of the Euclidean distance that accounts for correlations among the X properties. It is calculated as the distance to the center of the training data. The larger the MD, the less trustworthy the prediction.
Mahalanobis Distance p-value: The p-value gives the fraction of training data with an MD greater than or equal to the one for the given sample, assuming normally distributed data. The smaller the p-value, the less trustworthy the prediciton. For highly non-normal X properties (e.g., fingerprints), the MD p-value is wildly inaccurate.

Structural Similar Compounds

Name	PHENOLPHTHALEIN	DISPERSE YELLOW 3	D&C YELLOW NO. 11
Structure	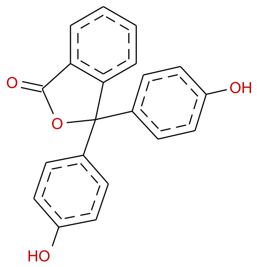	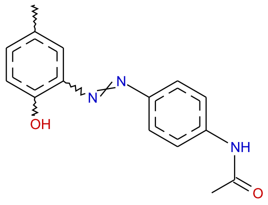	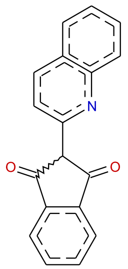
Actual Endpoint (-log C)	2.20184	2.77703	4.03869
Predicted Endpoint (-log C)	2.8857	2.80195	3.54593
Distance	0.464	0.606	0.606
Reference	NCI/NTP TR-465	NCI/NTP TR-222	NCI/NTP TR-463

Model Applicability

Unknown features are fingerprint features in the query molecule, but not found or appearing too infrequently in the training set.

1. All properties and OPS components are within expected ranges.
2. Unknown FCFP_2 feature: -306856457: [*]C(=[*])n1:[c](:[*]):[*]:[*]:c:1
3. Unknown FCFP_2 feature: -1549639687: [*]:n(:[*])C(=O)[c](:[*]):[*]

Feature Contribution

Top features for positive contribution

Fingerprint	Bit/Smiles	Feature Structure	Score
FCFP_2	17	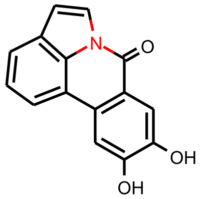 [*]:n:[*]	0.0441

Top Features for negative contribution

Fingerprint	Bit/Smiles	Feature Structure	Score

FCFP_2	7	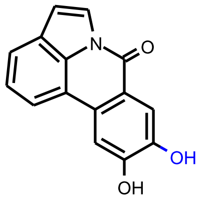 [*]O	-0.214
FCFP_2	-549108873	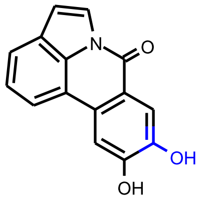 [*]:[c](:[*])O	-0.127
FCFP_2	1872154524	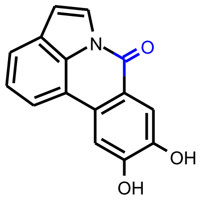 [*]C(=O)[*]	-0.105

remdesivir

TOPKAT_Rat_Maximum_Tolerated_Dose_Feed

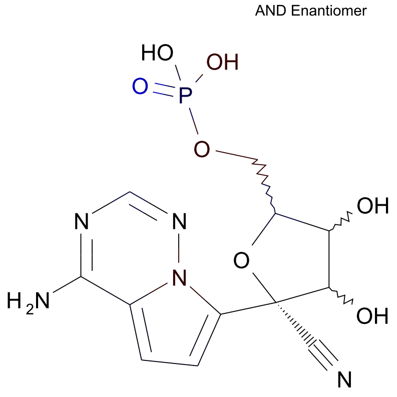

$C_{12}H_{14}N_5O_7P$

Molecular Weight: 371.24262

ALogP: -1.502

Rotatable Bonds: 4

Acceptors: 11

Donors: 5

Model Prediction

Prediction: 0.235

Unit: g/kg_body_weight

Mahalanobis Distance: 9.52

Mahalanobis Distance p-value: 0.000247

Mahalanobis Distance: The Mahalanobis distance (MD) is a generalization of the Euclidean distance that accounts for correlations among the X properties. It is calculated as the distance to the center of the training data. The larger the MD, the less trustworthy the prediction.

Mahalanobis Distance p-value: The p-value gives the fraction of training data with an MD greater than or equal to the one for the given sample, assuming normally distributed data. The smaller the p-value, the less trustworthy the prediciton. For highly non-normal X properties (e.g., fingerprints), the MD p-value is wildly inaccurate.

Structural Similar Compounds

Name	4,4'-DIAMINO-2,2'-STILBENEDISULFONIC ACID.2NaSALT	OXYTETRACYCLINE	50%1,4,5,8-TETRAAMINOANTHRAQUIONONE + DERIVATIVES
Structure	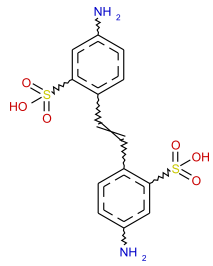	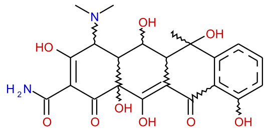	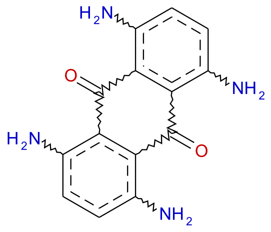
Actual Endpoint (-log C)	2.50759	2.36214	3.0764
Predicted Endpoint (-log C)	3.26068	2.77834	3.08142
Distance	0.743	0.818	0.989
Reference	NCI/NTP TR-412	NCI/NTP TR-315	NCI/NTP TR-299

Model Applicability

Unknown features are fingerprint features in the query molecule, but not found or appearing too infrequently in the training set.

1. Molecular_PolarSurfaceArea out of range. Value: 206.26. Training min, max, mean, SD: 0, 201.84, 63.052, 40.7.
2. Unknown FCFP_2 feature: 472180098: [*]OP(=O)(O)O
3. Unknown FCFP_2 feature: -836603894: [*]C1[*][*]O[C@]1(C#[*])[c](:[*]):[*]
4. Unknown FCFP_2 feature: -1277879912: [*]C([*])([*])C#N
5. Unknown FCFP_2 feature: -332197802: [*][c]1:[*]:[*]:[c](:[*]):n:1:n:[*]
6. Unknown FCFP_2 feature: -124685461: [*]:n:[cH]:n:[*]

Feature Contribution

Top features for positive contribution			
Fingerprint	Bit/Smiles	Feature Structure	Score

FCFP_2	-1143715940	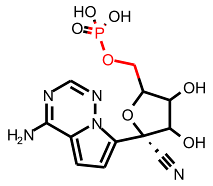 [*]COP(=[*])([*])[*]	0.095
FCFP_2	3	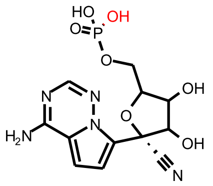 [*]N	0.0737
FCFP_2	17	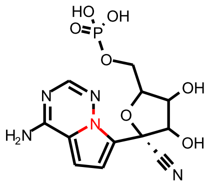 [*]:n:[*]	0.0441
**Top Features for negative contribution**			
**Fingerprint**	**Bit/Smiles**	**Feature Structure**	**Score**
FCFP_2	1872154524	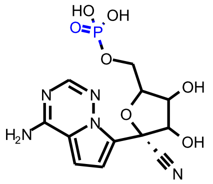 [*]C(=O)[*]	-0.105
FCFP_2	1	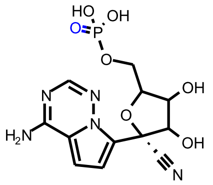 [*]O[*]	-0.0796

FCFP_2	16	AND Enantiomer<br>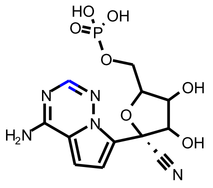<br>[*][c](:[*]):[*]	-0.0512

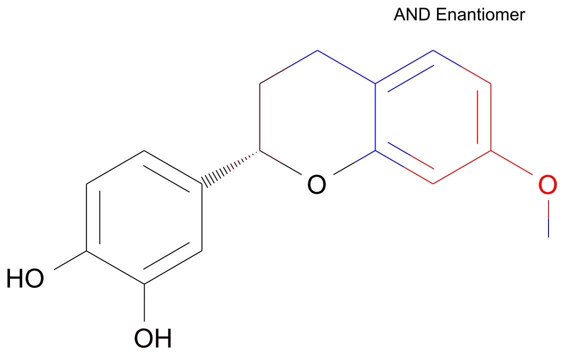

$C_{16}H_{16}O_4$
Molecular Weight: 272.296
ALogP: 3.456
Rotatable Bonds: 2
Acceptors: 4
Donors: 2

Model Prediction

Prediction: 0.000942
Unit: g/kg_body_weight
Mahalanobis Distance: 8.39
Mahalanobis Distance p-value: 0.000714

Mahalanobis Distance: The Mahalanobis distance (MD) is a generalization of the Euclidean distance that accounts for correlations among the X properties. It is calculated as the distance to the center of the training data. The larger the MD, the less trustworthy the prediction.
Mahalanobis Distance p-value: The p-value gives the fraction of training data with an MD greater than or equal to the one for the given sample, assuming normally distributed data. The smaller the p-value, the less trustworthy the prediciton. For highly non-normal X properties (e.g., fingerprints), the MD p-value is wildly inaccurate.

Structural Similar Compounds

Name	4-HEXYLRESORCINOL	1-TRANS-DELTA(9)-TETRAHYDROCANNABINOL	o-BENZYL-p-CHLOROPHENOL
Structure	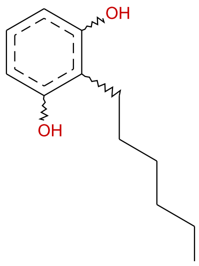	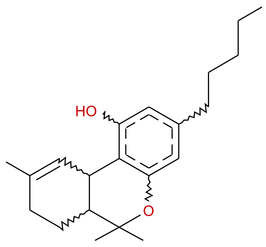	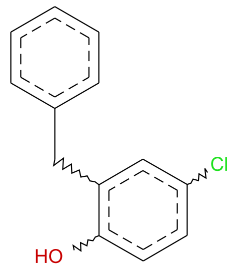
Actual Endpoint (-log C)	3.1915	3.79861	3.26063
Predicted Endpoint (-log C)	2.16134	4.44032	3.64448
Distance	0.639	0.756	0.795
Reference	NCI/NTP TR-330	NCI/NTP TR-446	NCI/NTP TR-424

Model Applicability

Unknown features are fingerprint features in the query molecule, but not found or appearing too infrequently in the training set.

1. OPS PC9 out of range. Value: 4.2189. Training min, max, SD, explained variance: -2.7086, 2.9267, 1.019, 0.0321.

Feature Contribution

Top features for positive contribution

Fingerprint	Bit/Smiles	Feature Structure	Score
FCFP_2	332760439	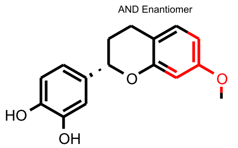 [*]O[c](:[cH]:[*]):[cH]:[*]	0.672

FCFP_2	1	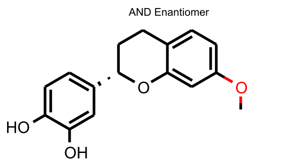<br>[*]O[*]	0.511
FCFP_2	-1272798659	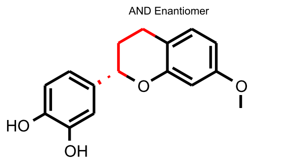<br>[*]CCC([*])[*]	0.0703
**Top Features for negative contribution**			
**Fingerprint**	**Bit/Smiles**	**Feature Structure**	**Score**
FCFP_2	203677720	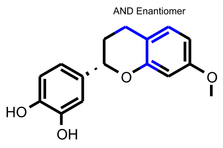<br>[*]C[c](:[cH]:[*]):[c]([*]):[*]	-0.406
FCFP_2	0	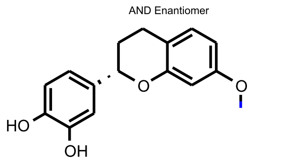<br>[*]C	-0.29

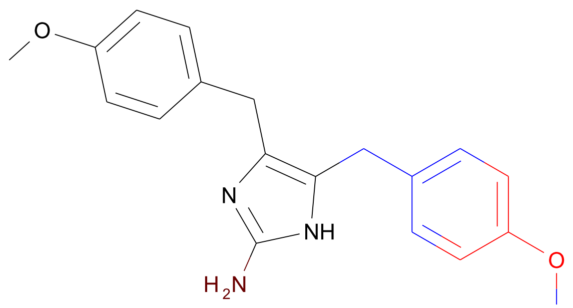

$C_{19}H_{21}N_3O_2$
Molecular Weight: 323.389
ALogP: 3.305
Rotatable Bonds: 6
Acceptors: 4
Donors: 2

Model Prediction

Prediction: 0.00611
Unit: g/kg_body_weight
Mahalanobis Distance: 7.81
Mahalanobis Distance p-value: 0.00344

Mahalanobis Distance: The Mahalanobis distance (MD) is a generalization of the Euclidean distance that accounts for correlations among the X properties. It is calculated as the distance to the center of the training data. The larger the MD, the less trustworthy the prediction.
Mahalanobis Distance p-value: The p-value gives the fraction of training data with an MD greater than or equal to the one for the given sample, assuming normally distributed data. The smaller the p-value, the less trustworthy the prediciton. For highly non-normal X properties (e.g., fingerprints), the MD p-value is wildly inaccurate.

Structural Similar Compounds

Name	SULFISOOXAZOLE	OCHRATOXIN	8-METHOXYPSORALEN
Structure	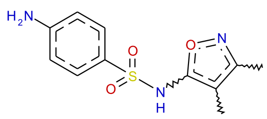	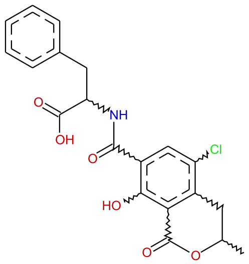	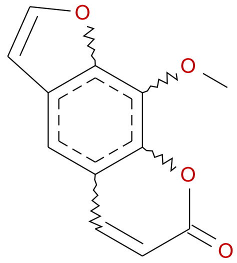
Actual Endpoint (-log C)	2.82494	6.28396	3.45978
Predicted Endpoint (-log C)	3.0705	5.12358	4.14745
Distance	0.831	0.956	0.982
Reference	NCI/NTP TR-138	NCI/NTP TR-358	NCI/NTP TR-359

Model Applicability

Unknown features are fingerprint features in the query molecule, but not found or appearing too infrequently in the training set.

1. Num_AromaticRings out of range. Value: 3. Training min, max, mean, SD: 0, 2, 0.5625, 0.693.
2. OPS PC6 out of range. Value: -3.283. Training min, max, SD, explained variance: -2.4321, 2.9885, 1.256, 0.0488.
3. Unknown FCFP_2 feature: 19: [*]:[nH]:[*]
4. Unknown FCFP_2 feature: 203707511: [*]C[c]1:[nH]:[*]:[*]:[c]:1[*]
5. Unknown FCFP_2 feature: 2005402822: [*][c]1:[*]:[*]:[c]([*]):[nH]:1
6. Unknown FCFP_2 feature: -1151854667: N[c]1:[nH]:[*]:[*]:n:1

Feature Contribution

Top features for positive contribution			
**Fingerprint**	**Bit/Smiles**	**Feature Structure**	**Score**
FCFP_2	332760439	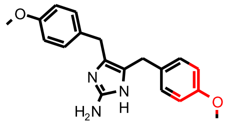 [*]O[c](:[cH]:[*]):[cH]:[*]	0.672

FCFP_2	1	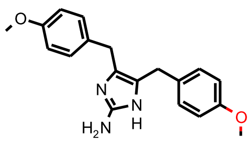 [*]O[*]	0.511
FCFP_2	3	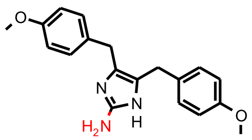 [*]N	0.104
**Top Features for negative contribution**			
**Fingerprint**	**Bit/Smiles**	**Feature Structure**	**Score**
FCFP_2	203677720	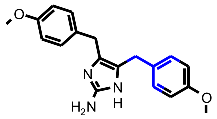 [*]C[c](:[cH]:[*]):[c]([*]):[*]	-0.406
FCFP_2	0	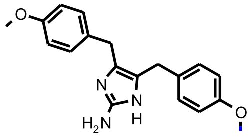 [*]C	-0.29

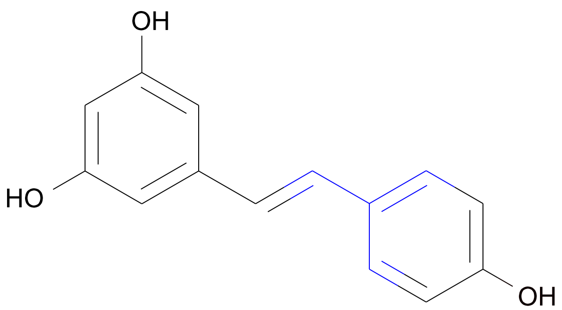

$C_{14}H_{12}O_3$
Molecular Weight: 228.243
ALogP: 3.09
Rotatable Bonds: 2
Acceptors: 3
Donors: 3

Model Prediction

Prediction: 0.102
Unit: g/kg_body_weight
Mahalanobis Distance: 8.92
Mahalanobis Distance p-value: 0.000155

Mahalanobis Distance: The Mahalanobis distance (MD) is a generalization of the Euclidean distance that accounts for correlations among the X properties. It is calculated as the distance to the center of the training data. The larger the MD, the less trustworthy the prediction.
Mahalanobis Distance p-value: The p-value gives the fraction of training data with an MD greater than or equal to the one for the given sample, assuming normally distributed data. The smaller the p-value, the less trustworthy the prediciton. For highly non-normal X properties (e.g., fingerprints), the MD p-value is wildly inaccurate.

Structural Similar Compounds

Name	4-HEXYLRESORCINOL	RESORCINOL	HYDROQUINONE
Structure	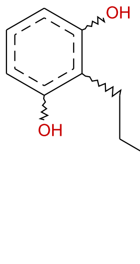	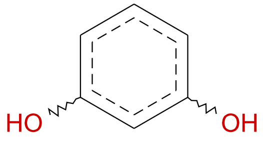	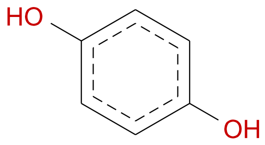
Actual Endpoint (-log C)	3.1915	2.99262	3.34286
Predicted Endpoint (-log C)	2.16134	3.36172	3.36172
Distance	0.632	0.699	0.699
Reference	NCI/NTP TR-330	NCI/NTP TR-403	NCI/NTP TR-366

Model Applicability

Unknown features are fingerprint features in the query molecule, but not found or appearing too infrequently in the training set.

1. All properties and OPS components are within expected ranges.

Feature Contribution

Top features for positive contribution

Fingerprint	Bit/Smiles	Feature Structure	Score
FCFP_2	7	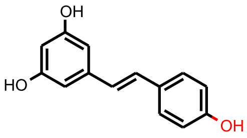 [*]O	0.0144

FCFP_2	74595001	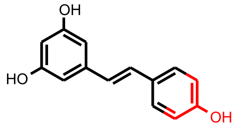 [*][c](:[*]):[c](O):[cH]:[*]	0.000246

Top Features for negative contribution			
**Fingerprint**	**Bit/Smiles**	**Feature Structure**	**Score**
FCFP_2	203677720	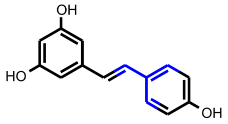 [*]C[c](:[cH]:[*]):[c]([*]):[*]	-0.406
FCFP_2	0	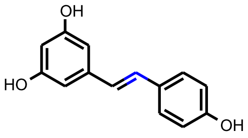 [*]C	-0.29

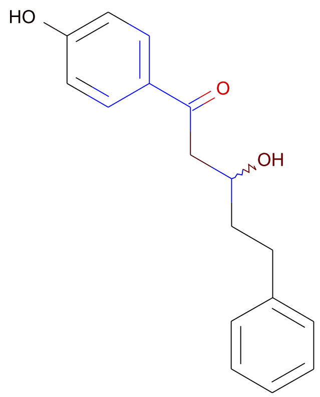

$C_{17}H_{18}O_3$
Molecular Weight: 270.323
ALogP: 3.293
Rotatable Bonds: 6
Acceptors: 3
Donors: 2

Model Prediction

Prediction: 0.21
Unit: g/kg_body_weight
Mahalanobis Distance: 9.54
Mahalanobis Distance p-value: 2.4e-005

Mahalanobis Distance: The Mahalanobis distance (MD) is a generalization of the Euclidean distance that accounts for correlations among the X properties. It is calculated as the distance to the center of the training data. The larger the MD, the less trustworthy the prediction.
Mahalanobis Distance p-value: The p-value gives the fraction of training data with an MD greater than or equal to the one for the given sample, assuming normally distributed data. The smaller the p-value, the less trustworthy the prediciton. For highly non-normal X properties (e.g., fingerprints), the MD p-value is wildly inaccurate.

Structural Similar Compounds

Name	4-HEXYLRESORCINOL	PROBENECID	o-BENZYL-p-CHLOROPHENOL
Structure	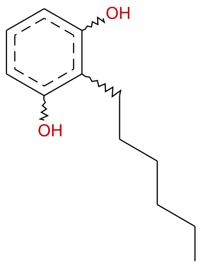	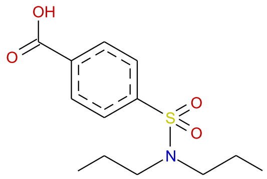	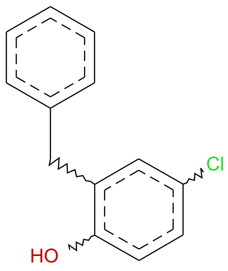
Actual Endpoint (-log C)	3.1915	2.85333	3.26063
Predicted Endpoint (-log C)	2.16134	2.4258	3.64448
Distance	0.581	0.726	0.828
Reference	NCI/NTP TR-330	NCI/NTP TR-395	NCI/NTP TR-424

Model Applicability

Unknown features are fingerprint features in the query molecule, but not found or appearing too infrequently in the training set.

1. All properties and OPS components are within expected ranges.
2. Unknown FCFP_2 feature: -1549192822: [*]CC(=O)[c](:[*]):[*]

Feature Contribution

Top features for positive contribution			
**Fingerprint**	**Bit/Smiles**	**Feature Structure**	**Score**
FCFP_2	1	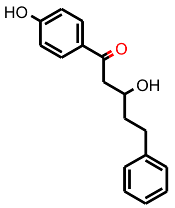 [*]O[*]	0.511

FCFP_2	3	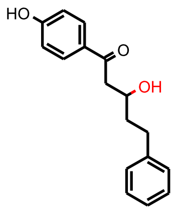 [*]N	0.104
FCFP_2	-1272798659	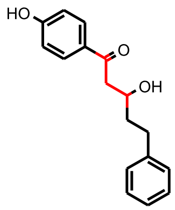 [*]CCC([*])[*]	0.0703

Top Features for negative contribution

**Fingerprint**	**Bit/Smiles**	**Feature Structure**	**Score**
FCFP_2	203677720	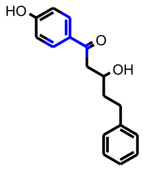 [*]C[c](:[cH]:[*]):[c]([*]):[*]	-0.406
FCFP_2	1872154524	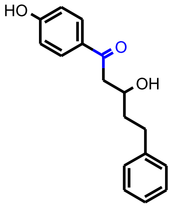 [*]C(=O)[*]	-0.307
FCFP_2	0	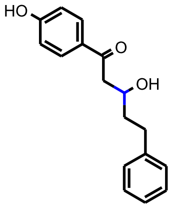 [*]C	-0.29

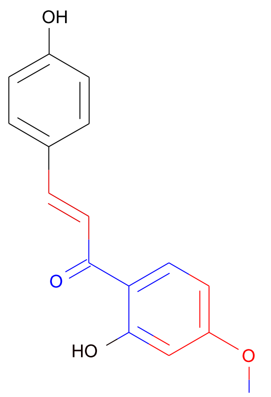

$C_{16}H_{14}O_4$
Molecular Weight: 270.28
ALogP: 3.201
Rotatable Bonds: 4
Acceptors: 4
Donors: 2

Model Prediction

Prediction: 0.00472
Unit: g/kg_body_weight
Mahalanobis Distance: 8.37
Mahalanobis Distance p-value: 0.000762

Mahalanobis Distance: The Mahalanobis distance (MD) is a generalization of the Euclidean distance that accounts for correlations among the X properties. It is calculated as the distance to the center of the training data. The larger the MD, the less trustworthy the prediction.
Mahalanobis Distance p-value: The p-value gives the fraction of training data with an MD greater than or equal to the one for the given sample, assuming normally distributed data. The smaller the p-value, the less trustworthy the prediciton. For highly non-normal X properties (e.g., fingerprints), the MD p-value is wildly inaccurate.

Structural Similar Compounds

Name	4-HEXYLRESORCINOL	PROBENECID	SULFISOOXAZOLE
Structure	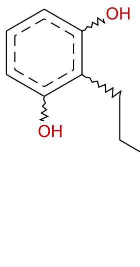	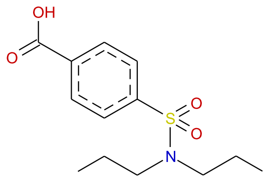	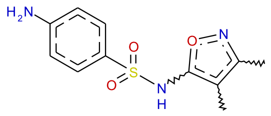
Actual Endpoint (-log C)	3.1915	2.85333	2.82494
Predicted Endpoint (-log C)	2.16134	2.4258	3.0705
Distance	0.734	0.760	0.771
Reference	NCI/NTP TR-330	NCI/NTP TR-395	NCI/NTP TR-138

Model Applicability

Unknown features are fingerprint features in the query molecule, but not found or appearing too infrequently in the training set.

1. OPS PC9 out of range. Value: 5.0866. Training min, max, SD, explained variance: -2.7086, 2.9267, 1.019, 0.0321.
2. Unknown FCFP_2 feature: -1549192822: [*]CC(=O)[c](:[*]):[*]

Feature Contribution

Top features for positive contribution

Fingerprint	Bit/Smiles	Feature Structure	Score
FCFP_2	332760439	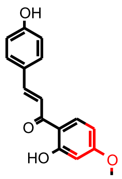 [*]O[c](:[cH]:[*]):[cH]:[*]	0.672

FCFP_2	1	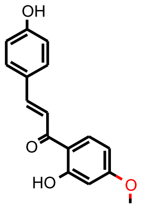 [*]O[*]	0.511
FCFP_2	451847724	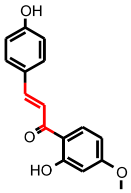 [*]\C=C\C(=[*])[*]	0.225

Top Features for negative contribution

Fingerprint	Bit/Smiles	Feature Structure	Score
FCFP_2	203677720	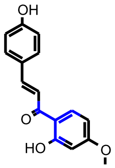 [*]C[c](:[cH]:[*]):[c]([*]):[*]	-0.406
FCFP_2	1872154524	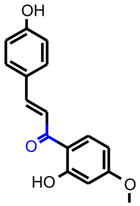 [*]C(=O)[*]	-0.307
FCFP_2	0	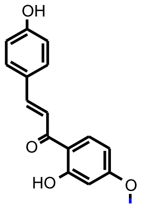 [*]C	-0.29

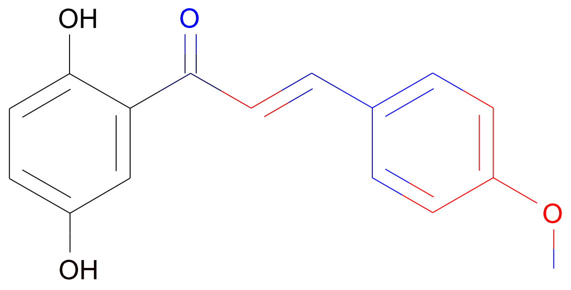

$C_{16}H_{14}O_4$
Molecular Weight: 270.28
ALogP: 3.201
Rotatable Bonds: 4
Acceptors: 4
Donors: 2

Model Prediction

Prediction: 0.00472
Unit: g/kg_body_weight
Mahalanobis Distance: 8.37
Mahalanobis Distance p-value: 0.000762

Mahalanobis Distance: The Mahalanobis distance (MD) is a generalization of the Euclidean distance that accounts for correlations among the X properties. It is calculated as the distance to the center of the training data. The larger the MD, the less trustworthy the prediction.
Mahalanobis Distance p-value: The p-value gives the fraction of training data with an MD greater than or equal to the one for the given sample, assuming normally distributed data. The smaller the p-value, the less trustworthy the prediciton. For highly non-normal X properties (e.g., fingerprints), the MD p-value is wildly inaccurate.

Structural Similar Compounds

Name	4-HEXYLRESORCINOL	PROBENECID	SULFISOOXAZOLE
Structure	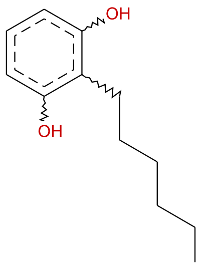	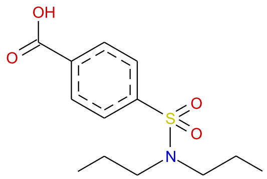	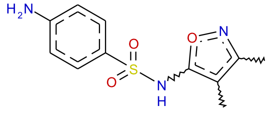
Actual Endpoint (-log C)	3.1915	2.85333	2.82494
Predicted Endpoint (-log C)	2.16134	2.4258	3.0705
Distance	0.734	0.760	0.771
Reference	NCI/NTP TR-330	NCI/NTP TR-395	NCI/NTP TR-138

Model Applicability

Unknown features are fingerprint features in the query molecule, but not found or appearing too infrequently in the training set.

1. OPS PC9 out of range. Value: 5.0866. Training min, max, SD, explained variance: -2.7086, 2.9267, 1.019, 0.0321.
2. Unknown FCFP_2 feature: -1549192822: [*]CC(=O)[c](:[*]):[*]

Feature Contribution

Top features for positive contribution

Fingerprint	Bit/Smiles	Feature Structure	Score
FCFP_2	332760439	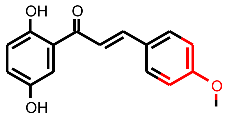 [*]O[c](:[cH]:[*]):[cH]:[*]	0.672

FCFP_2	1	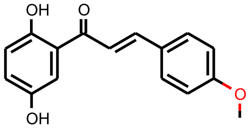 [*]O[*]	0.511
FCFP_2	451847724	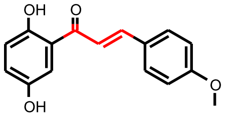 [*]\C=C\C(=[*])[*]	0.225
**Top Features for negative contribution**			
**Fingerprint**	**Bit/Smiles**	**Feature Structure**	**Score**
FCFP_2	203677720	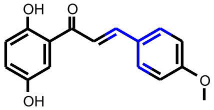 [*]C[c](:[cH]:[*]):[c]([*]):[*]	-0.406
FCFP_2	1872154524	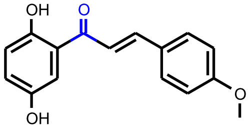 [*]C(=O)[*]	-0.307
FCFP_2	0	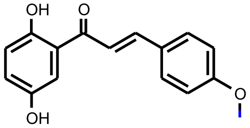 [*]C	-0.29

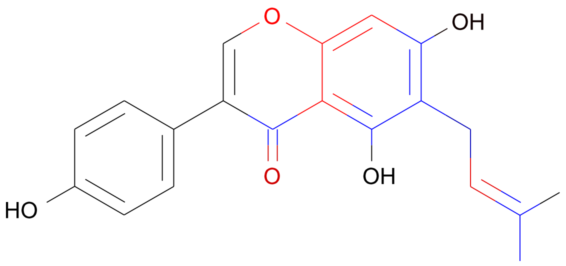

$C_{20}H_{18}O_5$
Molecular Weight: 338.354
ALogP: 3.997
Rotatable Bonds: 3
Acceptors: 5
Donors: 3

Model Prediction

Prediction: 0.00262
Unit: g/kg_body_weight
Mahalanobis Distance: 10.9
Mahalanobis Distance p-value: 3.42e-007

Mahalanobis Distance: The Mahalanobis distance (MD) is a generalization of the Euclidean distance that accounts for correlations among the X properties. It is calculated as the distance to the center of the training data. The larger the MD, the less trustworthy the prediction.
Mahalanobis Distance p-value: The p-value gives the fraction of training data with an MD greater than or equal to the one for the given sample, assuming normally distributed data. The smaller the p-value, the less trustworthy the prediciton. For highly non-normal X properties (e.g., fingerprints), the MD p-value is wildly inaccurate.

Structural Similar Compounds

Name	OCHRATOXIN	SULFISOOXAZOLE	PENICILLIN VK
Structure	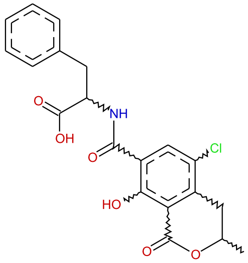	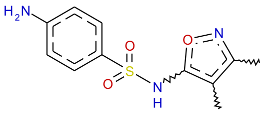	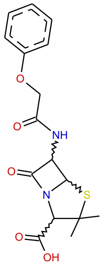
Actual Endpoint (-log C)	6.28396	2.82494	2.54455
Predicted Endpoint (-log C)	5.12358	3.0705	3.9702
Distance	0.608	0.851	0.895
Reference	NCI/NTP TR-358	NCI/NTP TR-138	NCI/NTP TR-336

Model Applicability

Unknown features are fingerprint features in the query molecule, but not found or appearing too infrequently in the training set.

1. All properties and OPS components are within expected ranges.
2. Unknown FCFP_2 feature: -1549192822: [*]CC(=O)[c](:[*]):[*]
3. Unknown FCFP_2 feature: -1678275541: [*]\C=C(/C(=[*])[*])\[c](:[*]):[*]

Feature Contribution

Top features for positive contribution

Fingerprint	Bit/Smiles	Feature Structure	Score
FCFP_2	332760439	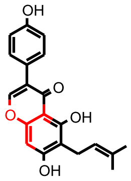 [*]O[c](:[cH]:[*]):[cH]:[*]	0.672

FCFP_2	1	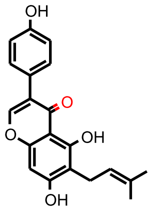 [*]O[*]	0.511
FCFP_2	451847724	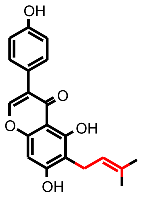 [*]\C=C\C(=[*])[*]	0.225
**Top Features for negative contribution**			
**Fingerprint**	**Bit/Smiles**	**Feature Structure**	**Score**
FCFP_2	136597326	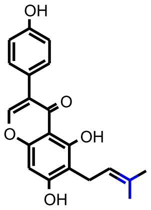 [*]C(=[*])C	-0.489
FCFP_2	203677720	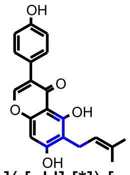 [*]C[c](:[cH]:[*]):[c]([*]):[*]	-0.406
FCFP_2	1872154524	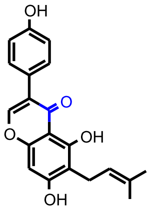 [*]C(=O)[*]	-0.307

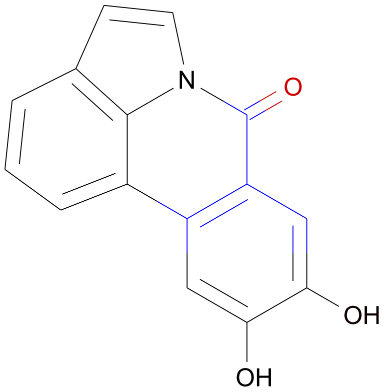

$C_{15}H_9NO_3$
Molecular Weight: 251.237
ALogP: 2.982
Rotatable Bonds: 0
Acceptors: 3
Donors: 2

Model Prediction

Prediction: 0.0412
Unit: g/kg_body_weight
Mahalanobis Distance: 9.34
Mahalanobis Distance p-value: 4.42e-005

Mahalanobis Distance: The Mahalanobis distance (MD) is a generalization of the Euclidean distance that accounts for correlations among the X properties. It is calculated as the distance to the center of the training data. The larger the MD, the less trustworthy the prediction.
Mahalanobis Distance p-value: The p-value gives the fraction of training data with an MD greater than or equal to the one for the given sample, assuming normally distributed data. The smaller the p-value, the less trustworthy the prediciton. For highly non-normal X properties (e.g., fingerprints), the MD p-value is wildly inaccurate.

Structural Similar Compounds

Name	2-MERCAPTOBENZOTHIAZOLE	8-METHOXYPSORALEN	SULFISOOXAZOLE
Structure	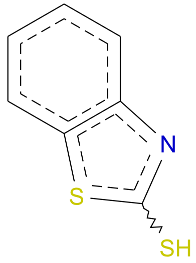	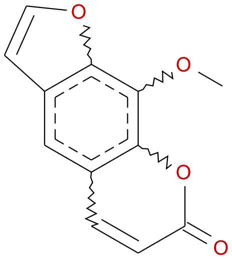	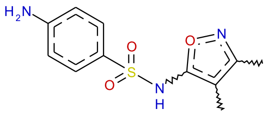
Actual Endpoint (-log C)	2.34829	3.45978	2.82494
Predicted Endpoint (-log C)	3.82125	4.14745	3.0705
Distance	0.775	0.834	0.864
Reference	NCI/NTP TR-332	NCI/NTP TR-359	NCI/NTP TR-138

Model Applicability

Unknown features are fingerprint features in the query molecule, but not found or appearing too infrequently in the training set.

1. Num_AromaticRings out of range. Value: 3. Training min, max, mean, SD: 0, 2, 0.5625, 0.693.
2. Unknown FCFP_2 feature: -1861645784: [*][c](:[*]):[c]([c](:[*]):[*]):c:[*]
3. Unknown FCFP_2 feature: -306856457: [*]C(=[*])n1:[c](:[*]):[*]:[*]:c:1
4. Unknown FCFP_2 feature: -1549639687: [*]:n(:[*])C(=O)[c](:[*]):[*]

Feature Contribution

Top features for positive contribution

Fingerprint	Bit/Smiles	Feature Structure	Score
FCFP_2	1	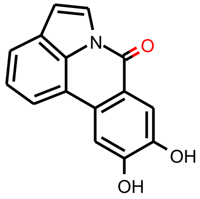 [*]O[*]	0.511

FCFP_2	7	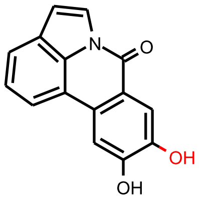 [*]O	0.0144
FCFP_2	74595001	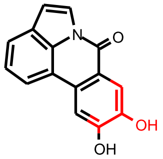 [*][c](:[*]):[c](O):[cH]:[*]	0.000246
**Top Features for negative contribution**			
**Fingerprint**	**Bit/Smiles**	**Feature Structure**	**Score**
FCFP_2	203677720	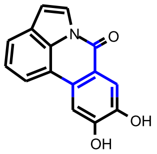 [*]C[c](:[cH]:[*]):[c]([*]):[*]	-0.406
FCFP_2	1872154524	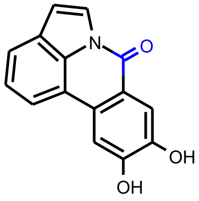 [*]C(=O)[*]	-0.307
FCFP_2	0	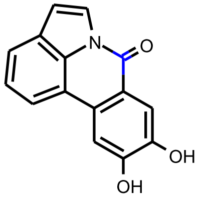 [*]C	-0.29

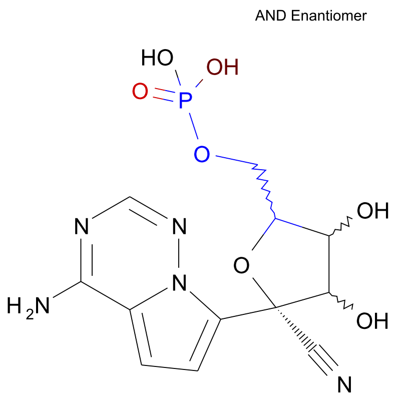

$C_{12}H_{14}N_5O_7P$

Molecular Weight: 371.24262

ALogP: -1.502

Rotatable Bonds: 4

Acceptors: 11

Donors: 5

Structural Similar Compounds

Name	AMPICILLIN TRIHYDRATE	OCHRATOXIN	PENICILLIN VK
Structure	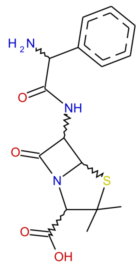	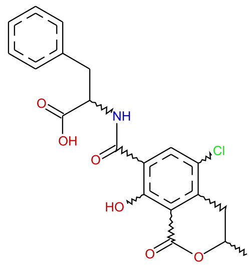	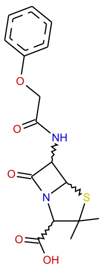
Actual Endpoint (-log C)	2.36724	6.28396	2.54455
Predicted Endpoint (-log C)	2.27651	5.12358	3.9702
Distance	1.255	1.482	1.498
Reference	NCI/NTP TR-318	NCI/NTP TR-358	NCI/NTP TR-336

Model Prediction

Prediction: 0.000298

Unit: g/kg_body_weight

Mahalanobis Distance: 17.2

Mahalanobis Distance p-value: 5.05e-016

Mahalanobis Distance: The Mahalanobis distance (MD) is a generalization of the Euclidean distance that accounts for correlations among the X properties. It is calculated as the distance to the center of the training data. The larger the MD, the less trustworthy the prediction.
Mahalanobis Distance p-value: The p-value gives the fraction of training data with an MD greater than or equal to the one for the given sample, assuming normally distributed data. The smaller the p-value, the less trustworthy the prediciton. For highly non-normal X properties (e.g., fingerprints), the MD p-value is wildly inaccurate.

Model Applicability

Unknown features are fingerprint features in the query molecule, but not found or appearing too infrequently in the training set.

1. Num_H_Donors out of range. Value: 5. Training min, max, mean, SD: 0, 3, 0.4375, 0.8311.
2. Num_H_Acceptors out of range. Value: 11. Training min, max, mean, SD: 0, 6, 1.6146, 1.644.
3. Molecular_PolarSASA out of range. Value: 321.97. Training min, max, mean, SD: 0, 223.97, 50.816, 55.15.
4. Molecular_PolarSurfaceArea out of range. Value: 206.26. Training min, max, mean, SD: 0, 138.03, 28.978, 32.1.
5. OPS PC1 out of range. Value: 9.0116. Training min, max, SD, explained variance: -4.0008, 7.9165, 2.861, 0.2531.
6. OPS PC5 out of range. Value: -4.1876. Training min, max, SD, explained variance: -3.4, 4.1587, 1.489, 0.0686.
7. OPS PC9 out of range. Value: -2.7276. Training min, max, SD, explained variance: -2.7086, 2.9267, 1.019, 0.0321.
8. Unknown FCFP_2 feature: 472180098: [*]OP(=O)(O)O
9. Unknown FCFP_2 feature: -836603894: [*]C1[*][*]O[C@]1(C#[*])[c](:[*]):[*]
10. Unknown FCFP_2 feature: -1277879912: [*]C([*])([*])C#N
11. Unknown FCFP_2 feature: -1362791977: [*]C#N
12. Unknown FCFP_2 feature: -332197802: [*][c]1:[*]:[*]:[c](:[*]):n:1:n:[*]
13. Unknown FCFP_2 feature: -124685461: [*]:n:[cH]:n:[*]

Feature Contribution

Top features for positive contribution			
**Fingerprint**	**Bit/Smiles**	**Feature Structure**	**Score**
FCFP_2	1	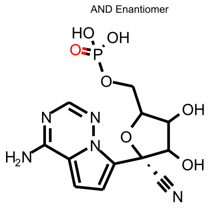 [*]O[*]	0.511
FCFP_2	3	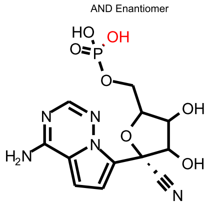 [*]N	0.104

Top Features for negative contribution			
**Fingerprint**	**Bit/Smiles**	**Feature Structure**	**Score**
FCFP_2	1872154524	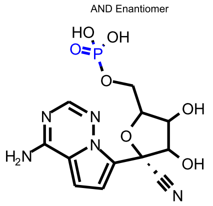 [*]C(=O)[*]	-0.307
FCFP_2	0	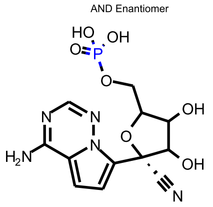 [*]C	-0.29

FCFP_2	-1272768868	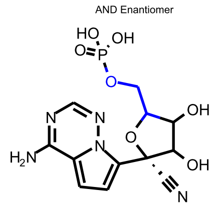 [*]OCC([*])[*]	-0.271

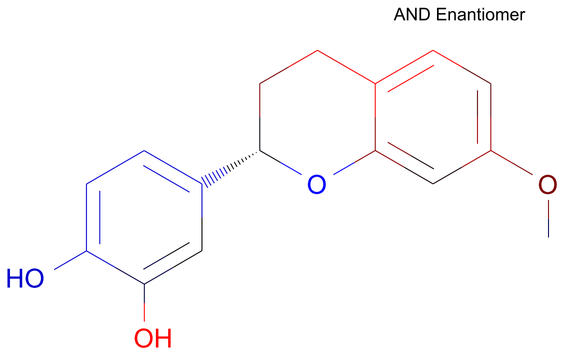

$C_{16}H_{16}O_4$
Molecular Weight: 272.296
ALogP: 3.456
Rotatable Bonds: 2
Acceptors: 4
Donors: 2

Model Prediction

Prediction: 0.441
Unit: g/kg_body_weight
Mahalanobis Distance: 16
Mahalanobis Distance p-value: 0.298

Mahalanobis Distance: The Mahalanobis distance (MD) is a generalization of the Euclidean distance that accounts for correlations among the X properties. It is calculated as the distance to the center of the training data. The larger the MD, the less trustworthy the prediction.
Mahalanobis Distance p-value: The p-value gives the fraction of training data with an MD greater than or equal to the one for the given sample, assuming normally distributed data. The smaller the p-value, the less trustworthy the prediciton. For highly non-normal X properties (e.g., fingerprints), the MD p-value is wildly inaccurate.

Structural Similar Compounds

Name	DIFLUNISAL	BUTANTRONE	PHENOL; 4;4'-THIODI-
Structure	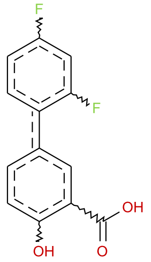	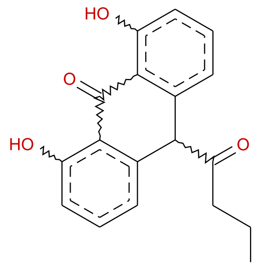	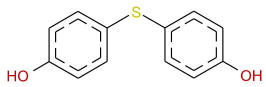
Actual Endpoint (-log C)	2.805	2.273	1.812
Predicted Endpoint (-log C)	2.41811	1.9286	2.50274
Distance	0.488	0.520	0.536
Reference	IYKEDH 15;688;84	ARTODN 59;180;86	BIOFX* A408;71

Model Applicability

Unknown features are fingerprint features in the query molecule, but not found or appearing too infrequently in the training set.

1. All properties and OPS components are within expected ranges.
2. Unknown FCFP_6 feature: 16: [*][c](:[*]):[*]
3. Unknown FCFP_6 feature: 1618154665: [*][c](:[*]):[cH]:[cH]:[*]
4. Unknown FCFP_6 feature: 1186333723: [*]C[C@H](O[*])[c](:[*]):[*]
5. Unknown FCFP_6 feature: 74595001: [*][c](:[*]):[c](O):[cH]:[*]
6. Unknown FCFP_6 feature: -549108873: [*]:[c](:[*])O

Feature Contribution

Top features for positive contribution			
**Fingerprint**	**Bit/Smiles**	**Feature Structure**	**Score**
ECFP_6	642810091	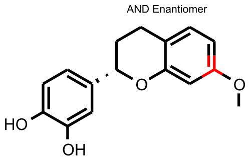 [*][c](:[*]):[*]	0.281

FCFP_6	136627117	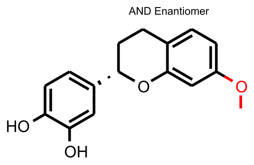 [*]OC	0.17
ECFP_6	1334973442	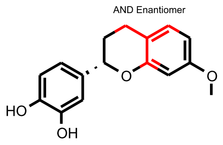 [*]C[c](:[cH]:[*]):[c]([*]):[*]	0.15

Top Features for negative contribution

Fingerprint	Bit/Smiles	Feature Structure	Score
ECFP_6	683445015	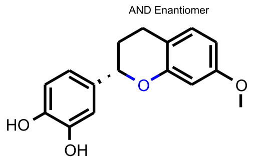 [*]O[*]	-0.266
ECFP_6	-176455838	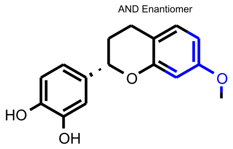 [*]O[c](:[cH]:[*]):[c H]:[*]	-0.257
FCFP_6	946589555	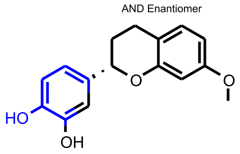 [*][c]1:[*]:[c]([*]): [c](O):[cH]:[cH]:1	-0.204

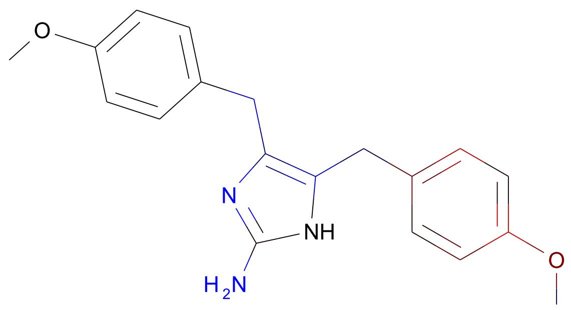

$C_{19}H_{21}N_3O_2$
Molecular Weight: 323.389
ALogP: 3.305
Rotatable Bonds: 6
Acceptors: 4
Donors: 2

Structural Similar Compounds

Name	FLUBENDAZOLE	CARBAMIC ACID; N-(5-BENZOYLBENZIMIDAZOL-2-YL)-; METHYL ESTER	ACRIDINE; 9-[3-(DIMETHYLAMINO)PROPYLAMINO]-1-NITRO-
Structure	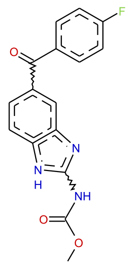	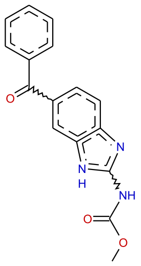	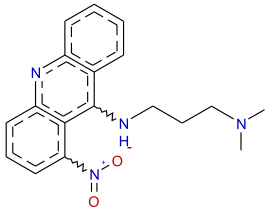
Actual Endpoint (-log C)	2.088	2.617	4.101
Predicted Endpoint (-log C)	2.69288	2.2368	3.3633
Distance	0.481	0.484	0.605
Reference	YRTMA6 9;11;78	IYKEDH 19;735;88	MMDPA6 8;252;76

Model Prediction

Prediction: 2.44
Unit: g/kg_body_weight
Mahalanobis Distance: 19.7
Mahalanobis Distance p-value: 5.75e-008

Mahalanobis Distance: The Mahalanobis distance (MD) is a generalization of the Euclidean distance that accounts for correlations among the X properties. It is calculated as the distance to the center of the training data. The larger the MD, the less trustworthy the prediction.
Mahalanobis Distance p-value: The p-value gives the fraction of training data with an MD greater than or equal to the one for the given sample, assuming normally distributed data. The smaller the p-value, the less trustworthy the prediciton. For highly non-normal X properties (e.g., fingerprints), the MD p-value is wildly inaccurate.

Model Applicability

Unknown features are fingerprint features in the query molecule, but not found or appearing too infrequently in the training set.

1. All properties and OPS components are within expected ranges.
2. Unknown ECFP_2 feature: -746759483: [*]C[c]1:[nH]:[*]:[*]:[c]:1[*]
3. Unknown ECFP_2 feature: -2046255371: N[c]1:[nH]:[*]:[*]:n:1
4. Unknown FCFP_6 feature: 16: [*][c](:[*]):[*]
5. Unknown FCFP_6 feature: 19: [*]:[nH]:[*]
6. Unknown FCFP_6 feature: 1618154665: [*][c](:[*]):[cH]:[cH]:[*]
7. Unknown FCFP_6 feature: 906530397: [*]:[c](:[*])C[c](:[*]):[*]
8. Unknown FCFP_6 feature: 203707511: [*]C[c]1:[nH]:[*]:[*]:[c]:1[*]
9. Unknown FCFP_6 feature: 2005402822: [*][c]1:[*]:[*]:[c]([*]):[nH]:1
10. Unknown FCFP_6 feature: -1151854667: N[c]1:[nH]:[*]:[*]:n:1
11. Unknown FCFP_6 feature: 1747237384: [*][c]1:[*]:[*]:[c]([*]):n:1
12. Unknown FCFP_6 feature: 1069584379: [*]:[c](:[*])N

Feature Contribution

Top features for positive contribution			
Fingerprint	Bit/Smiles	Feature Structure	Score

ECFP_6	642810091	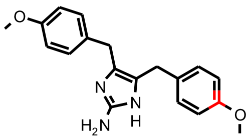 [*][c](:[*]):[*]	0.281
FCFP_6	136627117	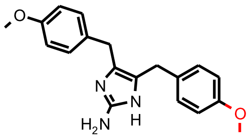 [*]OC	0.17
ECFP_6	-1059365320	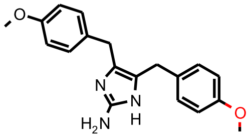 [*]O[*]	0.0976

Top Features for negative contribution

Fingerprint	Bit/Smiles	Feature Structure	Score
ECFP_6	-176455838	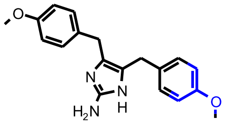 [*]O[c](:[cH]:[*]):[cH]:[*]	-0.257
ECFP_6	655739385	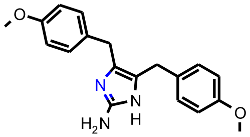 [*]:n:[*]	-0.239

ECFP_6	734603939	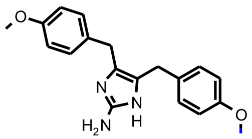 [*]C	-0.201

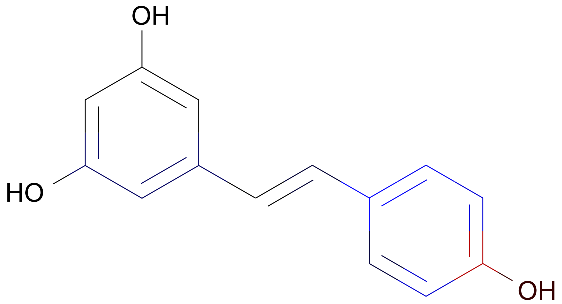

$C_{14}H_{12}O_3$
Molecular Weight: 228.243
ALogP: 3.09
Rotatable Bonds: 2
Acceptors: 3
Donors: 3

Model Prediction

Prediction: 2.48
Unit: g/kg_body_weight
Mahalanobis Distance: 13.9
Mahalanobis Distance p-value: 0.993

Mahalanobis Distance: The Mahalanobis distance (MD) is a generalization of the Euclidean distance that accounts for correlations among the X properties. It is calculated as the distance to the center of the training data. The larger the MD, the less trustworthy the prediction.
Mahalanobis Distance p-value: The p-value gives the fraction of training data with an MD greater than or equal to the one for the given sample, assuming normally distributed data. The smaller the p-value, the less trustworthy the prediciton. For highly non-normal X properties (e.g., fingerprints), the MD p-value is wildly inaccurate.

Structural Similar Compounds

Name	PHENOL; 4;4'-THIODI-	4;4'-DIHYDROXYDIPHENYLMETHANE	DIFLUNISAL
Structure	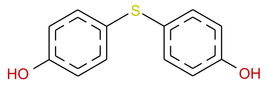	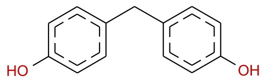	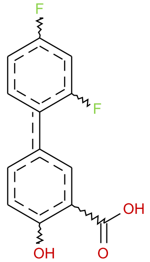
Actual Endpoint (-log C)	1.812	1.607	2.805
Predicted Endpoint (-log C)	2.50274	1.8824	2.41811
Distance	0.497	0.522	0.540
Reference	BIOFX* A408;71	AIHAAP 23;95;62	IYKEDH 15;688;84

Model Applicability

Unknown features are fingerprint features in the query molecule, but not found or appearing too infrequently in the training set.

1. All properties and OPS components are within expected ranges.
2. Unknown FCFP_6 feature: 16: [*][c](:[*]):[*]
3. Unknown FCFP_6 feature: -549108873: [*]:[c](:[*])O
4. Unknown FCFP_6 feature: 74595001: [*][c](:[*]):[c](O):[cH]:[*]
5. Unknown FCFP_6 feature: 1618154665: [*][c](:[*]):[cH]:[cH]:[*]
6. Unknown FCFP_6 feature: 451371068: [*]\C=C\[c](:[*]):[*]

Feature Contribution

Top features for positive contribution

Fingerprint	Bit/Smiles	Feature Structure	Score

ECFP_6	642810091	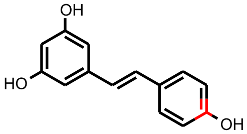 [*][c](:[*]):[*]	0.281
ECFP_6	2019062761	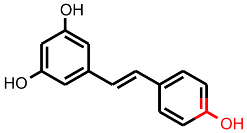 [*]:[c](:[*])O	0.138
FCFP_6	7	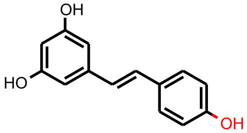 [*]O	0.0882

Top Features for negative contribution

Fingerprint	Bit/Smiles	Feature Structure	Score
FCFP_6	946589555	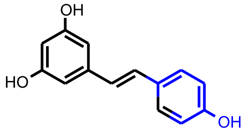 [*][c]1:[*]:[c]([*]):[c](O):[cH]:[cH]:1	-0.204
ECFP_6	-786013480	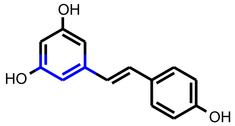 [*][c](:[*]):[cH]:[c]([*]):[*]	-0.0951

FCFP_6	0	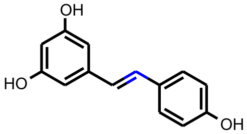 [*]C	-0.0791

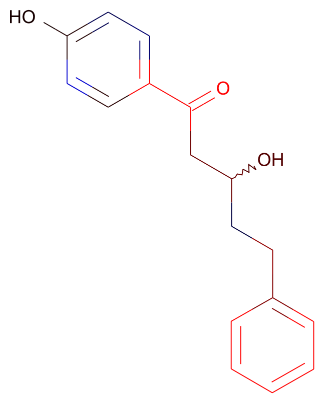

$C_{17}H_{18}O_3$
Molecular Weight: 270.323
ALogP: 3.293
Rotatable Bonds: 6
Acceptors: 3
Donors: 2

Structural Similar Compounds

Name	FENBUFEN	PRODOLIC ACID	BENZAMIDE; 2-(.beta.-HYDROXYETHOXY)-N-METHYL-5-PHENYL-
Structure	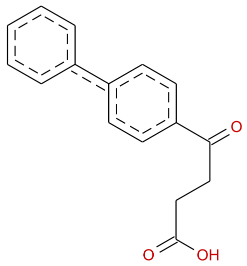	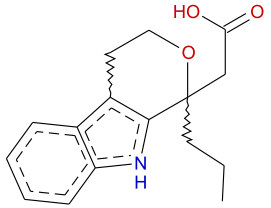	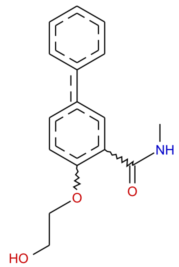
Actual Endpoint (-log C)	3.104	2.284	2.002
Predicted Endpoint (-log C)	2.29981	3.15318	2.05377
Distance	0.495	0.499	0.514
Reference	ARZNAD 30;721;80	AGACBH 4;370;74	JPETAB 108;450;53

Model Prediction

Prediction: 0.646
Unit: g/kg_body_weight
Mahalanobis Distance: 17.5
Mahalanobis Distance p-value: 0.0062

Mahalanobis Distance: The Mahalanobis distance (MD) is a generalization of the Euclidean distance that accounts for correlations among the X properties. It is calculated as the distance to the center of the training data. The larger the MD, the less trustworthy the prediction.
Mahalanobis Distance p-value: The p-value gives the fraction of training data with an MD greater than or equal to the one for the given sample, assuming normally distributed data. The smaller the p-value, the less trustworthy the prediciton. For highly non-normal X properties (e.g., fingerprints), the MD p-value is wildly inaccurate.

Model Applicability

Unknown features are fingerprint features in the query molecule, but not found or appearing too infrequently in the training set.

1. All properties and OPS components are within expected ranges.
2. Unknown FCFP_6 feature: 16: [*][c](:[*]):[*]
3. Unknown FCFP_6 feature: 1618154665: [*][c](:[*]):[cH]:[cH]:[*]
4. Unknown FCFP_6 feature: 74595001: [*][c](:[*]):[c](O):[cH]:[*]
5. Unknown FCFP_6 feature: -549108873: [*]:[c](:[*])O

Feature Contribution

Top features for positive contribution

Fingerprint	Bit/Smiles	Feature Structure	Score
ECFP_6	642810091	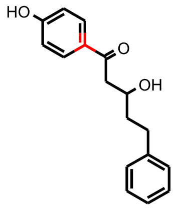 [*][c](:[*]):[*]	0.281

ECFP_6	1571214559	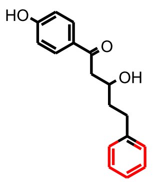 [*]1:[cH]:[cH]:[cH]:[cH]:[cH]:1	0.19
FCFP_6	-1549192822	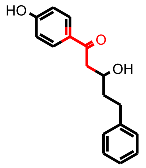 [*]CC(=O)[c](:[*]):[*]	0.168

Top Features for negative contribution

Fingerprint	Bit/Smiles	Feature Structure	Score
FCFP_6	946589555	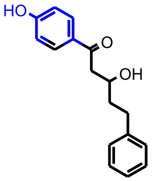 [*][c]1:[*]:[c]([*]):[c](O):[cH]:[cH]:1	-0.204
ECFP_6	-1795525632	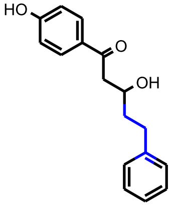 [*]CC[c](:[*]):[*]	-0.176
FCFP_6	3	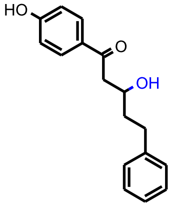 [*]N	-0.107

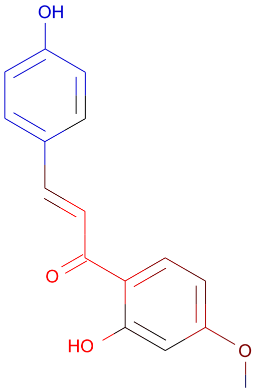

$C_{16}H_{14}O_4$
Molecular Weight: 270.28
ALogP: 3.201
Rotatable Bonds: 4
Acceptors: 4
Donors: 2

Structural Similar Compounds

Name	BUTANTRONE	NIFLUMIC ACID	CARBANILIC ACID; m-HYDROXY-; METHYL ESTER; m-METHYLCARBANILATE
Structure	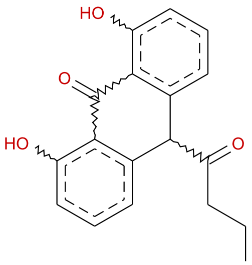	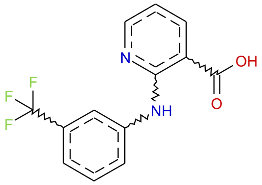	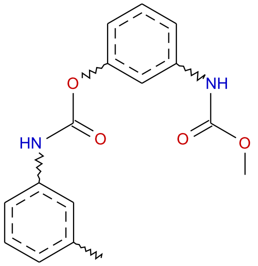
Actual Endpoint (-log C)	2.273	3.053	1.876
Predicted Endpoint (-log C)	1.9286	2.74769	1.81916
Distance	0.442	0.464	0.466
Reference	ARTODN 59;180;86	JMCMAR 16;780;73	GISAAA 49(4);16;84

Model Prediction

Prediction: 1.01
Unit: g/kg_body_weight
Mahalanobis Distance: 12.7
Mahalanobis Distance p-value: 1

Mahalanobis Distance: The Mahalanobis distance (MD) is a generalization of the Euclidean distance that accounts for correlations among the X properties. It is calculated as the distance to the center of the training data. The larger the MD, the less trustworthy the prediction.
Mahalanobis Distance p-value: The p-value gives the fraction of training data with an MD greater than or equal to the one for the given sample, assuming normally distributed data. The smaller the p-value, the less trustworthy the prediciton. For highly non-normal X properties (e.g., fingerprints), the MD p-value is wildly inaccurate.

Model Applicability

Unknown features are fingerprint features in the query molecule, but not found or appearing too infrequently in the training set.

1. All properties and OPS components are within expected ranges.
2. Unknown FCFP_6 feature: 16: [*][c](:[*]):[*]
3. Unknown FCFP_6 feature: 1618154665: [*][c](:[*]):[cH]:[cH]:[*]
4. Unknown FCFP_6 feature: 74595001: [*][c](:[*]):[c](O):[cH]:[*]
5. Unknown FCFP_6 feature: -549108873: [*]:[c](:[*])O
6. Unknown FCFP_6 feature: 451371068: [*]\C=C\[c](:[*]):[*]

Feature Contribution

Top features for positive contribution			
Fingerprint	Bit/Smiles	Feature Structure	Score

ECFP_6	642810091	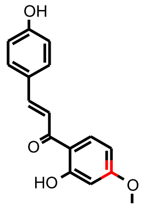 [*][c](:[*]):[*]	0.281
FCFP_6	136627117	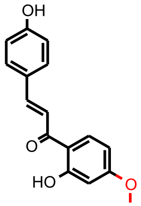 [*]OC	0.17
FCFP_6	-1549192822	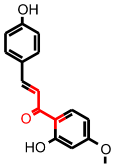 [*]CC(=O)[c](:[*]):[*]	0.168
**Top Features for negative contribution**			
**Fingerprint**	**Bit/Smiles**	**Feature Structure**	**Score**
ECFP_6	-176455838	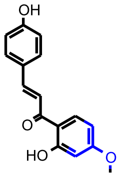 [*]O[c](:[cH]:[*]):[cH]:[*]	-0.257
FCFP_6	946589555	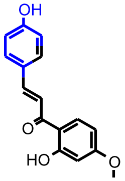 [*][c]1:[*]:[c]([*]):[c](O):[cH]:[cH]:1	-0.204

ECFP_6	734603939	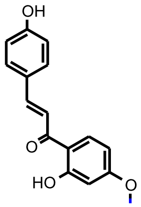 [*]C	-0.201

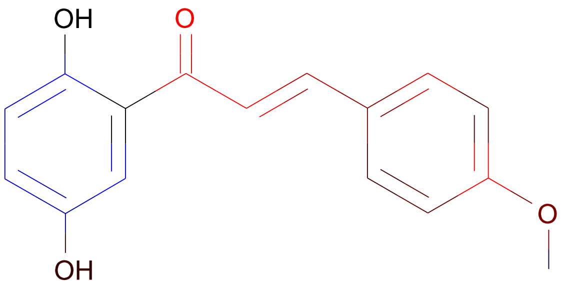

$C_{16}H_{14}O_4$

Molecular Weight: 270.28

ALogP: 3.201

Rotatable Bonds: 4

Acceptors: 4

Donors: 2

Structural Similar Compounds

Name	BUTANTRONE	NIFLUMIC ACID	CARBANILIC ACID; m-HYDROXY-; METHYL ESTER; m-METHYLCARBANILATE
Structure	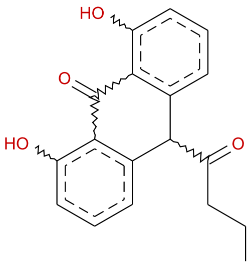	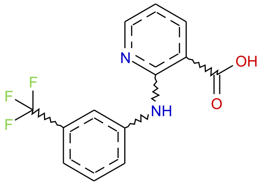	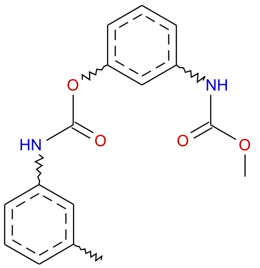
Actual Endpoint (-log C)	2.273	3.053	1.876
Predicted Endpoint (-log C)	1.9286	2.74769	1.81916
Distance	0.442	0.465	0.466
Reference	ARTODN 59;180;86	JMCMAR 16;780;73	GISAAA 49(4);16;84

Model Prediction

Prediction: 1.01

Unit: g/kg_body_weight

Mahalanobis Distance: 12.7

Mahalanobis Distance p-value: 1

Mahalanobis Distance: The Mahalanobis distance (MD) is a generalization of the Euclidean distance that accounts for correlations among the X properties. It is calculated as the distance to the center of the training data. The larger the MD, the less trustworthy the prediction.
Mahalanobis Distance p-value: The p-value gives the fraction of training data with an MD greater than or equal to the one for the given sample, assuming normally distributed data. The smaller the p-value, the less trustworthy the prediciton. For highly non-normal X properties (e.g., fingerprints), the MD p-value is wildly inaccurate.

Model Applicability

Unknown features are fingerprint features in the query molecule, but not found or appearing too infrequently in the training set.

1. All properties and OPS components are within expected ranges.
2. Unknown FCFP_6 feature: 16: [*][c](:[*]):[*]
3. Unknown FCFP_6 feature: 1618154665: [*][c](:[*]):[cH]:[cH]:[*]
4. Unknown FCFP_6 feature: 451371068: [*]\C=C\[c](:[*]):[*]
5. Unknown FCFP_6 feature: 74595001: [*][c](:[*]):[c](O):[cH]:[*]
6. Unknown FCFP_6 feature: -549108873: [*]:[c](:[*])O

Feature Contribution

Top features for positive contribution

Fingerprint	Bit/Smiles	Feature Structure	Score

ECFP_6	642810091	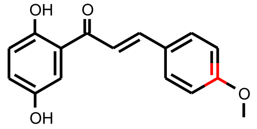 [*][c](:[*]):[*]	0.281
FCFP_6	136627117	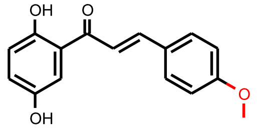 [*]OC	0.17
FCFP_6	-1549192822	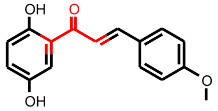 [*]CC(=O)[c](:[*]):[*]	0.168
**Top Features for negative contribution**			
**Fingerprint**	**Bit/Smiles**	**Feature Structure**	**Score**
ECFP_6	-176455838	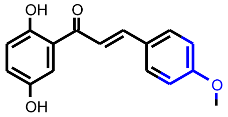 [*]O[c](:[cH]:[*]):[c H]:[*]	-0.257
FCFP_6	946589555	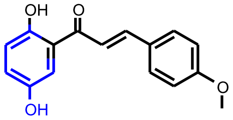 [*][c]1:[*]:[c]([*]): [c](O):[cH]:[cH]:1	-0.204

ECFP_6	734603939	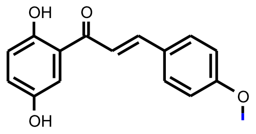 [*]C	-0.201

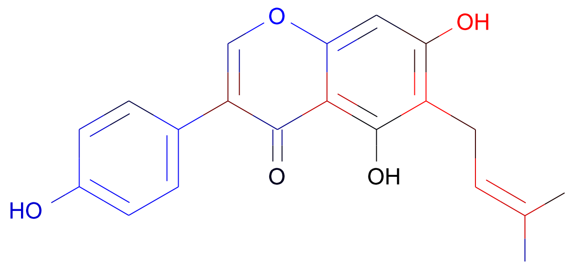

$C_{20}H_{18}O_5$
Molecular Weight: 338.354
ALogP: 3.997
Rotatable Bonds: 3
Acceptors: 5
Donors: 3

Model Prediction

Prediction: 0.962
Unit: g/kg_body_weight
Mahalanobis Distance: 18.3
Mahalanobis Distance p-value: 0.000146

Mahalanobis Distance: The Mahalanobis distance (MD) is a generalization of the Euclidean distance that accounts for correlations among the X properties. It is calculated as the distance to the center of the training data. The larger the MD, the less trustworthy the prediction.
Mahalanobis Distance p-value: The p-value gives the fraction of training data with an MD greater than or equal to the one for the given sample, assuming normally distributed data. The smaller the p-value, the less trustworthy the prediciton. For highly non-normal X properties (e.g., fingerprints), the MD p-value is wildly inaccurate.

Structural Similar Compounds

Name	FLUORESCEINE; SODIUM SALT (Na STRIPPED)	DICOUMAROL	BUTANTRONE
Structure	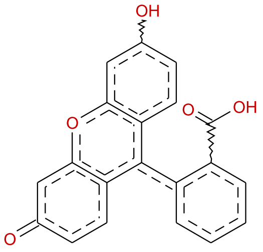	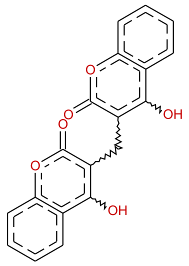	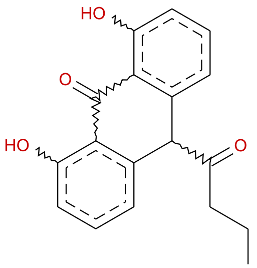
Actual Endpoint (-log C)	1.694	3.129	2.273
Predicted Endpoint (-log C)	2.71831	2.86156	1.9286
Distance	0.529	0.568	0.588
Reference	JOPRAJ 48;228;77	SMWOAS 83;471;53	ARTODN 59;180;86

Model Applicability

Unknown features are fingerprint features in the query molecule, but not found or appearing too infrequently in the training set.

1. All properties and OPS components are within expected ranges.
2. Unknown ECFP_2 feature: 1717082529: [*]\C=C(/C(=[*])[*])\[c](:[*]):[*]
3. Unknown FCFP_6 feature: 16: [*][c](:[*]):[*]
4. Unknown FCFP_6 feature: 74595001: [*][c](:[*]):[c](O):[cH]:[*]
5. Unknown FCFP_6 feature: -549108873: [*]:[c](:[*])O
6. Unknown FCFP_6 feature: 1618154665: [*][c](:[*]):[cH]:[cH]:[*]
7. Unknown FCFP_6 feature: -1678275541: [*]\C=C(/C(=[*])[*])\[c](:[*]):[*]

Feature Contribution

Top features for positive contribution

Fingerprint	Bit/Smiles	Feature Structure	Score

ECFP_6	642810091	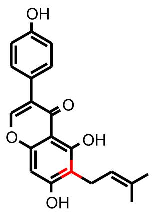<br>[*][c](:[*]):[*]	0.281
FCFP_6	-1549192822	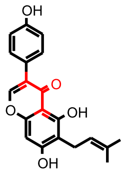<br>[*]CC(=O)[c](:[*]):[*<br>]	0.168
ECFP_6	-1074141656	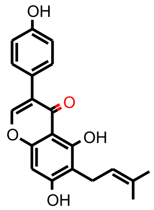<br>[*]=O	0.142
**Top Features for negative contribution**			
**Fingerprint**	**Bit/Smiles**	**Feature Structure**	**Score**
ECFP_6	2106656448	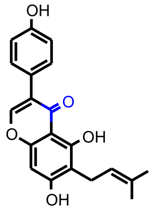<br>[*]C(=O)[*]	-0.352
ECFP_6	683445015	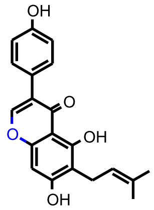<br>[*]O[*]	-0.266

FCFP_6	946589555	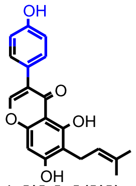 [*][c]1:[*]:[c]([*]):[c](O):[cH]:[cH]:1	-0.204

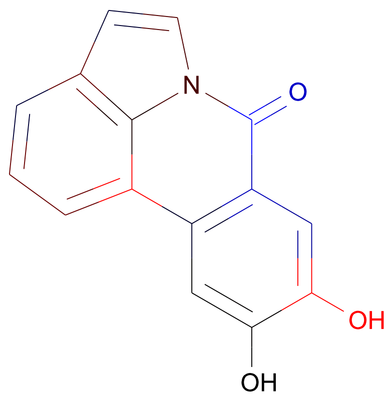

$C_{15}H_9NO_3$
Molecular Weight: 251.237
ALogP: 2.982
Rotatable Bonds: 0
Acceptors: 3
Donors: 2

Model Prediction

Prediction: 0.788
Unit: g/kg_body_weight
Mahalanobis Distance: 18.1
Mahalanobis Distance p-value: 0.000545

Mahalanobis Distance: The Mahalanobis distance (MD) is a generalization of the Euclidean distance that accounts for correlations among the X properties. It is calculated as the distance to the center of the training data. The larger the MD, the less trustworthy the prediction.
Mahalanobis Distance p-value: The p-value gives the fraction of training data with an MD greater than or equal to the one for the given sample, assuming normally distributed data. The smaller the p-value, the less trustworthy the prediciton. For highly non-normal X properties (e.g., fingerprints), the MD p-value is wildly inaccurate.

Structural Similar Compounds

Name	NAPTALAM	TRP-P-1	DITHRANOL
Structure	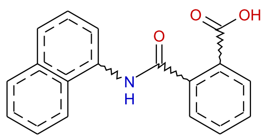	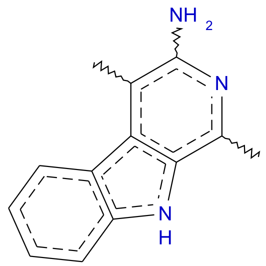	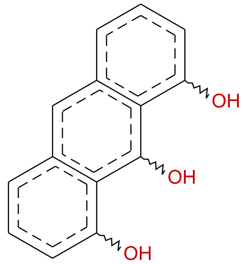
Actual Endpoint (-log C)	1.551	3.325	1.847
Predicted Endpoint (-log C)	1.89036	2.34981	2.28476
Distance	0.544	0.551	0.558
Reference	FMCHA2 -;C206;89	PPTCBY 9;159;79	ARTODN 59;180;86

Model Applicability

Unknown features are fingerprint features in the query molecule, but not found or appearing too infrequently in the training set.

1. All properties and OPS components are within expected ranges.
2. Unknown ECFP_2 feature: 1312166648: [*]C(=[*])n1:[c](:[*]):[*]:[*]:c:1
3. Unknown FCFP_6 feature: 16: [*][c](:[*]):[*]
4. Unknown FCFP_6 feature: -549108873: [*]:[c](:[*])O
5. Unknown FCFP_6 feature: 74595001: [*][c](:[*]):[c](O):[cH]:[*]
6. Unknown FCFP_6 feature: 1618154665: [*][c](:[*]):[cH]:[cH]:[*]
7. Unknown FCFP_6 feature: -1861645784: [*][c](:[*]):[c](:[cH]:[*])[c](:[*]):[*]
8. Unknown FCFP_6 feature: -306856457: [*]C(=[*])n1:[cH]:[*]:[*]:[c]:1:[*]
9. Unknown FCFP_6 feature: -1549639687: [*]:n(:[*])C(=O)[c](:[*]):[*]

Feature Contribution

Top features for positive contribution

Fingerprint	Bit/Smiles	Feature Structure	Score

ECFP_6	642810091	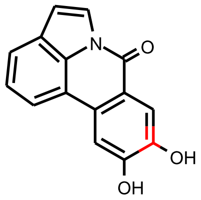 [*][c](:[*]):[*]	0.281
ECFP_6	-1074141656	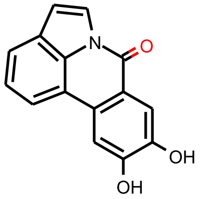 [*]=O	0.142
ECFP_6	2019062761	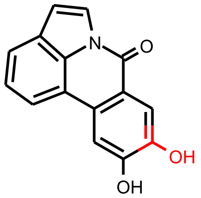 [*]:[c](:[*])O	0.138

Top Features for negative contribution

Fingerprint	Bit/Smiles	Feature Structure	Score
ECFP_6	2106656448	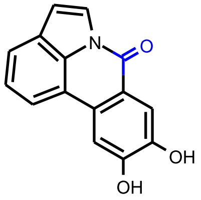 [*]C(=O)[*]	-0.352
ECFP_6	-178525456	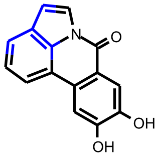 [*]:[cH]:[c]1:[cH]:[*]:[*]:[c]:1:[*]	-0.157

ECFP_6	-786013480	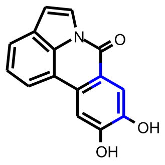 [*][c](:[*]):[cH]:[c]([*]):[*]	-0.0951

remdesivir

TOPKAT_Rat_Oral_LD50

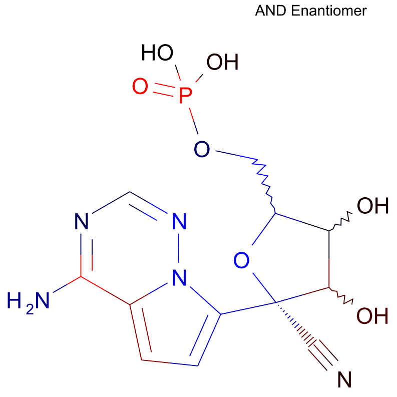

$C_{12}H_{14}N_5O_7P$
Molecular Weight: 371.24262
ALogP: -1.502
Rotatable Bonds: 4
Acceptors: 11
Donors: 5

Structural Similar Compounds

Name	5'-ADENYLIC ACID; POTASSIUM SALT (K STRIPPED)	INOSINATE; DISODIUM SALT (Na STRIPPED)	INOSINE-5'-PHOSPHORIC ACID
Structure	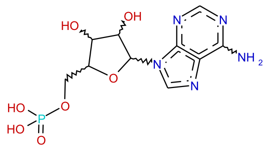	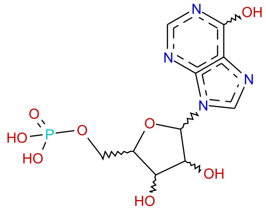	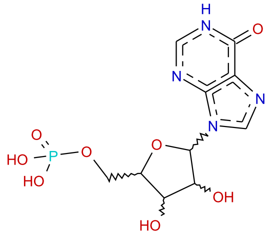
Actual Endpoint (-log C)	1.49	1.34	1.338
Predicted Endpoint (-log C)	2.45569	2.92201	1.35922
Distance	0.361	0.428	0.592
Reference	OYYAA2 4;689;70	AJINO* -;-;73	ARTODN 47;77;81

Model Prediction

Prediction: 0.309
Unit: g/kg_body_weight
Mahalanobis Distance: 29.4
Mahalanobis Distance p-value: 1.72e-059

Mahalanobis Distance: The Mahalanobis distance (MD) is a generalization of the Euclidean distance that accounts for correlations among the X properties. It is calculated as the distance to the center of the training data. The larger the MD, the less trustworthy the prediction.
Mahalanobis Distance p-value: The p-value gives the fraction of training data with an MD greater than or equal to the one for the given sample, assuming normally distributed data. The smaller the p-value, the less trustworthy the prediciton. For highly non-normal X properties (e.g., fingerprints), the MD p-value is wildly inaccurate.

Model Applicability

Unknown features are fingerprint features in the query molecule, but not found or appearing too infrequently in the training set.

1. OPS PC10 out of range. Value: 15.526. Training min, max, SD, explained variance: -6.0395, 14.892, 2.468, 0.0220.
2. Unknown ECFP_2 feature: 1258791451: [*]C1[*][*]O[C@]1(C#[*])[c](:[*]):[*]
3. Unknown ECFP_2 feature: -264833661: [*]C([*])([*])C#N
4. Unknown ECFP_2 feature: -66263742: [*]C([*])([*])[c]1:[cH]:[*]:[*]:n:1:[*]
5. Unknown FCFP_6 feature: 16: [*][c](:[*]):[*]
6. Unknown FCFP_6 feature: 472180098: [*]OP(=O)(O)O
7. Unknown FCFP_6 feature: -836603894: [*]C1[*][*]O[C@]1(C#[*])[c](:[*]):[*]
8. Unknown FCFP_6 feature: -332197802: [*][c]1:[*]:[*]:[c](:[*]):n:1:n:[*]
9. Unknown FCFP_6 feature: 4427049: [*]:[cH]:n:n(:[*]):[*]
10. Unknown FCFP_6 feature: -124685461: [*]:n:[cH]:n:[*]
11. Unknown FCFP_6 feature: 1747237384: [*][c]1:[*]:[*]:[c]([*]):n:1
12. Unknown FCFP_6 feature: -1151884458: [*]:n:[c](N):[c](:[*]):[*]
13. Unknown FCFP_6 feature: 1618154665: [*][c](:[*]):[cH]:[cH]:[*]
14. Unknown FCFP_6 feature: 1069584379: [*]:[c](:[*])N

Feature Contribution

Top features for positive contribution

Fingerprint	Bit/Smiles	Feature Structure	Score
ECFP_6	642810091	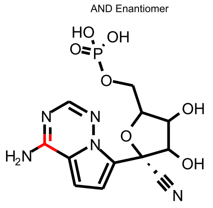 [*][c](:[*]):[*]	0.281
ECFP_6	-826638028	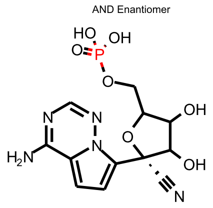 [*]P(=[*])([*])[*]	0.225
ECFP_6	2100964382	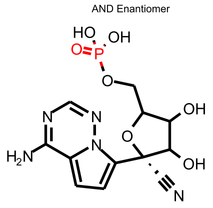 [*]P(=O)([*])[*]	0.166

Top Features for negative contribution

Fingerprint	Bit/Smiles	Feature Structure	Score
ECFP_6	683445015	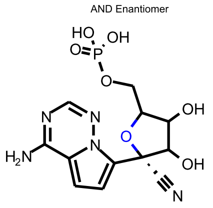 [*]O[*]	-0.266

ECFP_6	655739385	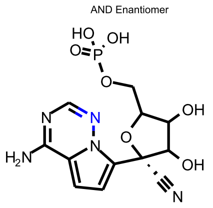 [*]:n:[*]	-0.239
FCFP_6	-1539132615	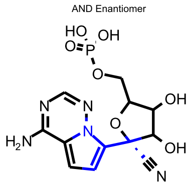 [*]C[c]1:n:[*]:[*]:[c]:1[*]	-0.2
